# The G Protein–Coupled Receptor Subset of the Chicken Genome

**DOI:** 10.1371/journal.pcbi.0020054

**Published:** 2006-06-02

**Authors:** Malin C Lagerström, Anders R Hellström, David E Gloriam, Thomas P Larsson, Helgi B Schiöth, Robert Fredriksson

**Affiliations:** Department of Neuroscience, Uppsala University, Uppsala, Sweden; University of California San Diego, United States of America

## Abstract

G protein–coupled receptors (GPCRs) are one of the largest families of proteins, and here we scan the recently sequenced chicken genome for GPCRs. We use a homology-based approach, utilizing comparisons with all human GPCRs, to detect and verify chicken GPCRs from translated genomic alignments and Genscan predictions. We present 557 manually curated sequences for GPCRs from the chicken genome, of which 455 were previously not annotated. More than 60% of the chicken Genscan gene predictions with a human ortholog needed curation, which drastically changed the average percentage identity between the human–chicken orthologous pairs (from 56.3% to 72.9%). Of the non-olfactory chicken GPCRs, 79% had a one-to-one orthologous relationship to a human GPCR. The *Frizzled, Secretin,* and subgroups of the *Rhodopsin* families have high proportions of orthologous pairs, although the percentage of amino acid identity varies. Other groups show large differences, such as the *Adhesion* family and GPCRs that bind exogenous ligands. The chicken has only three bitter *Taste 2* receptors, and it also lacks an ortholog to human TAS1R2 (one of three GPCRs in the human genome in the *Taste 1* receptor family [TAS1R]), implying that the chicken's ability and mode of detecting both bitter and sweet taste may differ from the human's. The chicken genome contains at least 229 olfactory receptors, and the majority of these (218) originate from a chicken-specific expansion. To our knowledge, this dataset of chicken GPCRs is the largest curated dataset from a single gene family from a non-mammalian vertebrate. Both the updated human GPCR dataset, as well the chicken GPCR dataset, are available for download.

## Synopsis

Man and chicken are very different, but how is that difference related to our respective gene repertoire? The authors studied the family of G protein–coupled receptors (GPCRs), which in man contains about 791 proteins. These are found in the cell membrane, where they recognize substances, thereby functioning as mediators of signals across the cellular membrane. GPCRs respond to physiologically important substances such as hormones and neurotransmitters. In this paper, the publicly available genomic sequence from the domestic chicken is used to identify the entire repertoire of GPCRs in this species. The authors found 557 GPCRs and compared the chicken and human receptors; they concluded that out of the 328 chicken receptors that are not involved in olfaction, more than 250 have a corresponding human receptor. The majority of the differences between the chicken and man are within three groups of GPCRs—the receptors for olfaction, bitter taste, and the receptors involved in the immune system. The chicken GPCR sequences obtained here will be useful for identification of GPCRs in other species that are more distantly related to man, such as fish or insects. The domestic chicken represents the leading experimental model among the avian species and also serves as an important source of food worldwide.

## Introduction

Several vertebrate genomes have been sequenced since the release of the first draft sequence of the human genome in 2001 [1,2], but the first project to fill the large evolutionary gap between mammals and fish was the completion of the genome of the red jungle fowl, Gallus gallus, released in December 2004 [3]. Besides bridging the gap between mammals and other vertebrates, the chicken represents the leading experimental model among the avian species and serves also as an important source of food worldwide.

The initial chicken genome annotation suggested between 20,000 and 23,000 protein-coding genes [3], which is similar to what has been estimated for the human genome [4,5]. It is, however, well known that automatic annotations of new genomes are error-prone, and tremendous work is left in annotation of the different protein families. The prediction of coding sequences of multi-exon genes is complicated, and one of the best programs, Genscan, has a sensitivity and specificity of about 90% for detecting exons, meaning that the average gene with about ten exons is very likely to have at least one exon wrongly predicted. Moreover, it has been estimated that the number of exons that have both splice sites correctly predicted by Genscan is as low as only 19% [6]. This shows that the likelihood of correctly predicting multi-exon genes is fairly low. This has, of course, a substantial impact on subsequent analysis such as phylogeny and calculations of evolutionary distances, when gene predictions are used instead of curated full-length genes.

The superfamily of G protein–coupled receptors (GPCRs) is one of the largest families of proteins in the human genome [1,2] and probably also in most other vertebrate species [7]. The GPCR family has approximately 800 members in man, and these participate in numerous important physiological functions and are also targets for many therapeutic drugs. Their natural ligands are particularly diverse including ions, organic odorants, amines, peptides, proteins, lipids, nucleotides, and photons, which are all able to activate GPCRs. The name GPCR indicates that these receptors interact with G-proteins, but the main common characteristic of GPCRs are seven stretches of about 20–35 consecutive amino acid residues that show a high degree of hydrophobicity and represent α-helixes that span the plasma membrane.

The complete repertoire of GPCRs has been analyzed for several vertebrate genomes such as the human [8,9], mouse [8], mosquito [10], and pufferfish [11] genomes. The numbers of GPCRs in gene-prediction datasets from 13 eukaryotic genomes, not including the chicken genome, was also recently investigated [7]. Most of the genome-wide analyses have, however, been performed on raw gene predictions which make reliable phylogenetic analyses impossible. Currently, only the repertoire of GPCRs in the human [8,9] and mouse [8] genomes has been analyzed using curated-sequence datasets. Both physiological and structural features have been used to classify GPCRs [12–14]. These classification systems were constructed before the completion of human and other vertebrate genomes and hence did not classify atypical receptors not yet identified, such as most of the *Adhesion* (long N-termini–transmembrane-7 [LN-TM7]) family receptors [15] and the bitter-taste receptors. In addition, these classification systems did not phylogenetically subdivide the large rhodopsin family, which has approximately 660 members in humans, into groups. Recently, we performed large-scale systematic phylogenetic analyses, including the majority of the GPCRs in the human genome [9]. This provided us with the GRAFS system showing five main families of GPCRs named *Glutamate* (G), *Rhodopsin* (R), *Adhesion* (A), *Frizzled/Taste2* (F), and *Secretin* (S). Moreover, we subdivided the large *Rhodopsin* family into four groups; α, β, γ, and δ. The grouping was performed with strict phylogenetic criteria where some atypical human receptors were placed into a group designated *Other.*

In this paper, we scan the recently sequenced chicken genome for GPCRs, using a multitude of methods to obtain a nearly complete set of chicken GPCRs. We manually edit and verify, i.e., curate, the coding regions of each of the GPCRs (557 in total), to provide the first high-quality collection of GPCR sequences from the full genome of a non-mammalian species. We perform maximum-likelihood phylogenetic analysis on these chicken GPCRs together with 750 human GPCRs, which is an updated version of the dataset used previously ([9]; unpublished data), which comprises the entire human GPCR family. We present a strategy of detecting and verifying genes from a genomic assembly and evaluate the accuracy of Genscan predictions in relation to our homology-based gene-prediction approach.

## Results

### Identification and Verification of Chicken GPCRs

**Figure 1:**
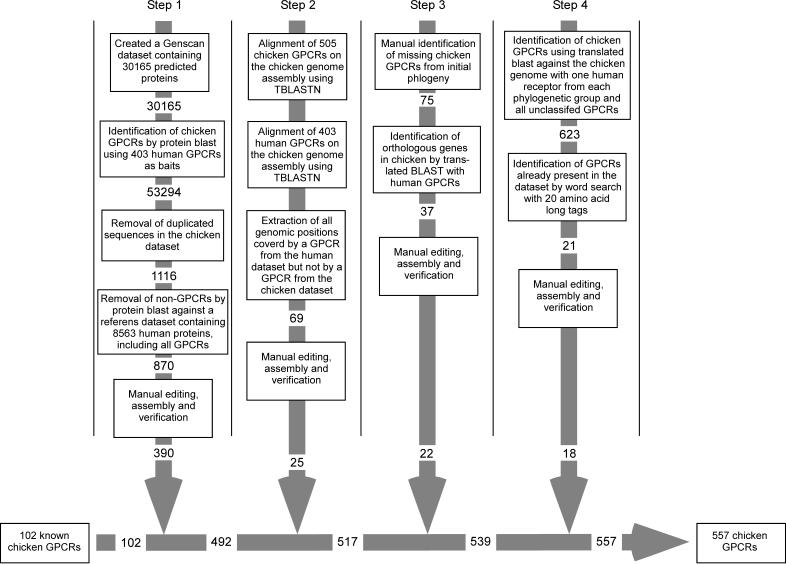
Flowchart Describing the Sequence Analysis Strategy Used in This Work Briefly, in the first step, a Genscan dataset was created from the Ensembl February 2004 assembly of the chicken genome. These 30,165 predicted proteins were then searched against a human reference set using BLAST, and 53,294 proteins were selected as possible GPCRs. After removal of multiple hits, 1,116 potential proteins remained. After elimination of non-GPCRs, all 870 GPCR-like sequences were manually inspected and corrected, pseudogenes were removed, and multiple hits representing the same protein were merged. With the completion of step 1, the sequences of 390 new chicken GPCRs were identified. In step 2, a set of 505 putative chicken GPCRs were aligned together with a human reference set to the chicken genome. All sites with a human hit, but without a chicken hit, were extracted and manually processed. Step 2 identified 25 possible chicken GPCRs. In step 3, an initial phylogenetic analysis was performed to identify possible missing orthologs. These human receptor proteins were searched against the chicken genome. All hits with an *E*-value of better than *E* = 1e−6 were compared to all collected chicken GPCRs. A total of 22 new chicken GPCRs were identified in this way after manual assembly and verification. In the fourth and final step, 18 additional chicken GPCR-like sequences were identified using crude searches against the chicken genome with a selection of human GPCRs as baits. All hits with an *E*-value of better than *E* = 0.1 were manually compared with all previously identified chicken GPCRs. In total, 455 new potential GPCR-like sequences were identified using this approach.

Initially, 102 known chicken GPCRs from the nr (http://www.ncbi.nlm.nih.gov/BLAST/Blast.cgi) database at NCBI were identified using BLASTP with all human GPCRs as baits [9]. In Figure 1, we describe a four-step process of identifying an additional 455 GPCRs from the chicken genome. In step 1, we created a Genscan dataset from the Ensembl February 2004 assembly of the chicken genome (http://www.ensembl.org). This resulted in 30,165 Genscan predictions, and we used all human GPCRs as baits in BLASTP searches to obtain, in total, 53,294 hits. After removal of multiple hits, 1,116 putative chicken GPCRs remained which, after removal of non-GPCRs using BLASTP, was reduced to 870. Finally, all these 870 GPCR-like sequences were manually inspected and corrected, pseudogenes were removed, and multiple hits representing the same protein were merged. The final result of step 1 was the identification of 390 new chicken GPCR sequences.

Furthermore, three additional steps were taken to identify GPCR sequences that were not represented among the Genscan predictions. In step 2, we aligned all human GPCRs as well as 505 putative chicken GPCRs against the chicken genome using TBLASTN. All genomic positions that were hit by a human GPCR, but not by a chicken GPCR, were manually inspected for additional new chicken GPCRs. Only the best hit from each search was considered. Step 2 identified 25 new chicken GPCRs.

In step 3, we first produced neighbor-joining phylogenetic trees for all chicken and human GPCRs. Subsequently, in all cases where a one-to-one orthologous relationship between a human and a chicken sequence was not present, we manually identified all human sequences that did lack an orthologous chicken sequence and used these to search the chicken genome using TBLASTN. All genomic positions containing a human hit but not a chicken GPCR were then manually inspected for additional new chicken GPCRs. This resulted in the identification of 22 additional chicken GPCRs.

In step 4, we used a strategy similar to that used in step 2, but here we used a limited number of GPCRs as baits and inspected all genomic positions, not only the top scoring, for additional new chicken GPCRs. This step identified 18 new chicken GPCRs. In total, 455 new chicken GPCRs were identified. The chicken GPCR dataset was divided into *Adhesion, Frizzled, Glutamate, Secretin, Rhodopsin,* and *Taste 2* families based on the human dataset. The large *Rhodopsin* family was further subdivided into α, β, γ, δ, and *Olfactory.* Table S1 describes the step in which receptors from the different families were identified.

### The Accuracy of Genscan Predictions

It has to be noted that in this process one crucial, but tedious, step was the manual editing/assembly/verification step. Here, wrongly predicted regions were excluded, new splice sites were selected to correct frame-shifted regions of the protein, and the most likely start codon was selected based on alignment with the human orthologous protein. In addition, missing regions were identified by the use of translated alignments against the genomic regions in those cases where those missing sequences were expected to be found from comparison with the human genome. To illustrate the importance of this process, we performed pairwise alignments between the corrected protein and the initial Genscan prediction for all non-olfactory sequences with a human ortholog from step 1.

**Figure 2:**
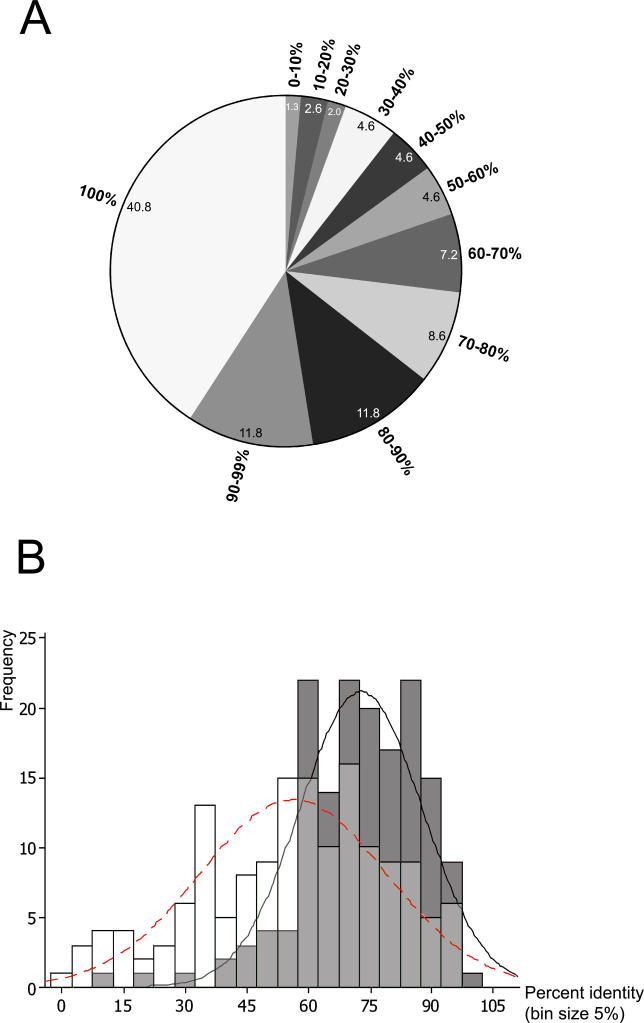
Comparison between Curated and Non-Curated Chicken GPCR Sequences (A) The chart describes the percentage identity between the original Genscan prediction and the manually curated version of the chicken proteins for 158 sequence pairs. The segment labeled 100% contains those proteins that were correctly predicted by Genscan, while the segment labeled 0%–10% contains those pairs that had almost no correctly predicted material. (B) A histogram describing the percentage identity between 158 human–chicken orthologous pairs as identified from the phylogenetic trees. The solid line and the grey bars represent the comparison between the manually edited chicken proteins and the human orthologs, while the dotted line and the white bars represent the comparison between the human proteins and the non-edited Genscan predictions. The mean percentage identities are 72.9% (standard deviation 14.9) and 56.3% (standard deviation 22.7) for the comparison with the edited and non-edited chicken sequences, respectively. The datasets fit a normal distribution with *p* = 0.04 and *p* = 0.08, respectively, using the Kolmogorov–Smirnov test (MiniTab). The lines in the graphs are fitted assuming normal distribution.

From each of these alignments, the percentage identity between the sequences was calculated, using a scale where 100 means completely identical and 0 means no identity. The result of this comparison can be seen in Figure 2A. It is evident that less than 41% of the sequences are correctly predicted. The mean percentage identity between the Genscan predictions and each corresponding protein in the curated dataset is 80.7% (median 58.6) with a large standard deviation of 25.3. It has to be noted here that flanking regions that cannot be aligned outside of the TM regions in the predictions were excluded from the comparison. If these were included, the difference would be even larger. One important point is that the percentage error in the Genscan prediction could, at the nucleotide level, be lower. For example, a wrongly predicted splice site could introduce a frame-shift that would have a significant impact on the predicted protein, although the number of wrongly predicted bases could be small.

When comparing species, one factor that is often discussed is the sequence identity of orthologous genes as this gives a measure of the evolutionary distance between the two species. Figure 2B illustrates the difference in calculating percentage identity between the chicken and human genomes using 158 manually edited proteins, based only on the TM regions, or the corresponding unedited protein predictions, similar to what was done on gene predictions from various types of proteins by Hillier et al. (see Figure 6 in Hillier et al. [3]). Figure 2B shows a histogram with a bin size of 5 with the percentage identity, calculated with infoalign [16], on the *y*-axis and with frequency on the *x*-axis. It is obvious from Figure 2B that the difference is remarkable; the mean value for the sequence identity using the edited proteins (solid line and grey bars) is 72.9% (standard deviation 14.9), while the unedited proteins (dotted line and white bars) gives a mean value of 56.4% (standard deviation 22.7). The amino acid identity, calculated from global alignments, varies between the families of GPCRs. Of the 158 one-to-one orthologous pairs of human and curated chicken GPCRs, the percentage amino acid identity for the different families are 68.8 *(Adhesion);* 81.4 *(Frizzled);* 71.6 *(Glutamate);* 73.8 (*Rhodopsin* α); 77.3 (*Rhodopsin* β); 69.2 (*Rhodopsin* γ); 73.8 (*Rhodopsin* δ-excluding olfactory receptors); and 72.1 *(Secretin).*

### Phylogenetic Analysis

**Figure 3:**
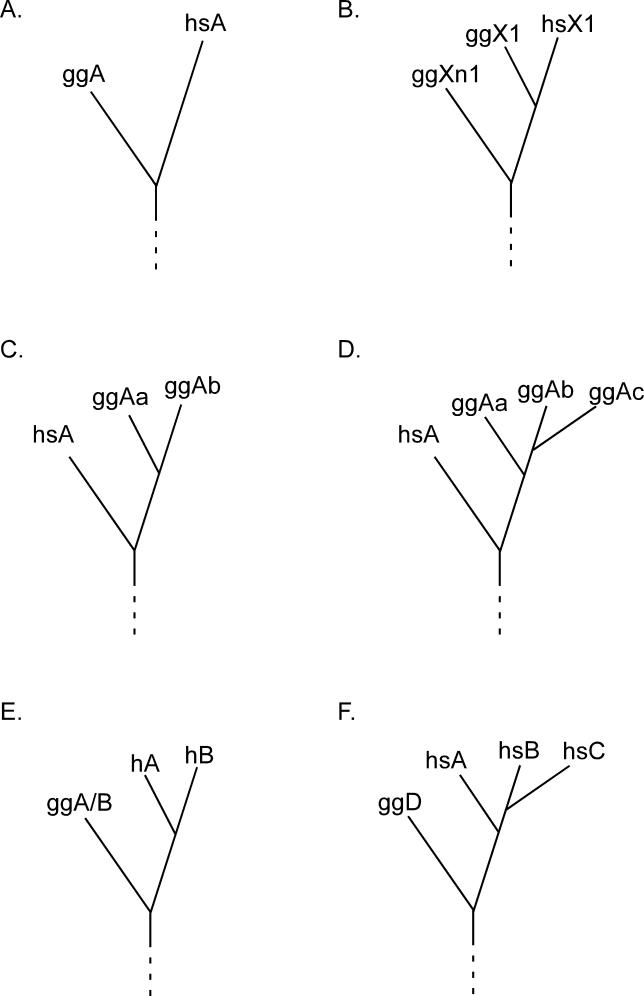
The Nomenclature Definitions That We Used to Classify the Various Outcomes of the Phylogenetic Relationships between the Chicken and Human GPCRs (A) Orthologs. The chicken sequence will inherit the human sequence name with “gg” *(G. gallus)* as prefix (according to the guidelines of CHICKBASE hosted at the Roslin Institute). (B) One orthologous pair in receptor family X together with a missing human ortholog. The chicken sequence will inherit the receptor family name “X” together with the appendix “n1” (novel 1); for example, see Figure 5A ggGPR119n1. (C) Gene duplication in the chicken genome/gene loss in the human genome. The chicken sequences will inherit the human sequence name. The two chicken sequences will be discriminated by “a, b” appendix; for example, see Figure 5A ggADORA2Ba and ggADORA2Bb. (D) Gene expansion in the chicken genome/gene loss in the human genome (*n* > 2). The chicken sequences will inherit the name of the closest human sequence. The chicken sequences will be discriminated by appendix “a, b, c …”; for example, see Figure 5D ggGPR43n1a–1h. (E) Gene duplication in the human genome/gene loss in the chicken genome. The chicken sequence will inherit a combination of the two human sequence names; for example, see Figure 4A ggGPR111/115. (F) Gene expansion in the human genome/gene loss in the chicken genome (*n* > 2). The chicken gene will be given a novel name associated with the closest human receptor family; for example, see Figure 5D ggMRGn1.

**Figure 4:**
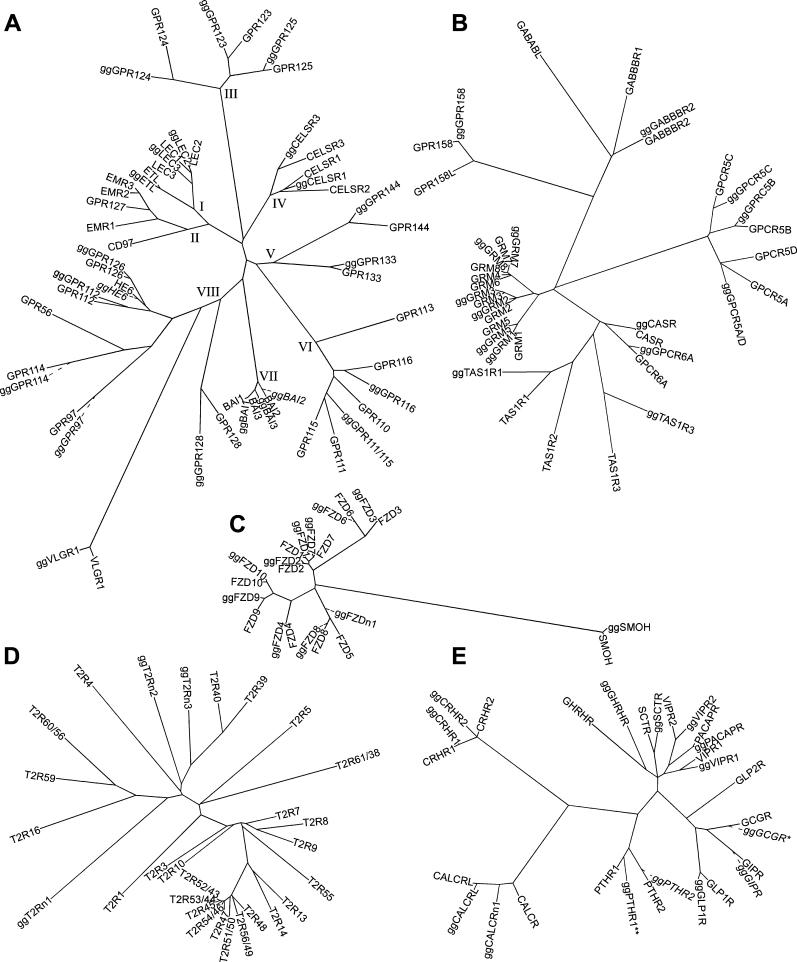
Phylogenetic Relationship between Human and Chicken GPCR Sequences Phylogenetic analysis was performed by first calculating neighbor-joining trees (except for Figure 4A where a maximum-parsimony topology was used) with 100 bootstrap replicas for each of the ten groups described in Table 1 and then mapping maximum-likelihood branch lengths onto the topology using TreePuzzle. The trees were visualized in TreeView [94]. Dotted lines represent the position of a receptor protein with a partial TM region. These positions are based on a separate calculation. (A) The *Adhesion* receptor family. I–VIII represents the different groups of the *Adhesion* family [15]. (B) The *Glutamate* receptor family. (C) FZD. (D) TAS2R. (E) The *Secretin* receptor family. A single asterisk indicates that the position is based on sequence alignment with the human GCGR. Only a fragment of the N-terminus was found. A double asterisk indicates possible pseudogene.

**Figure 5:**
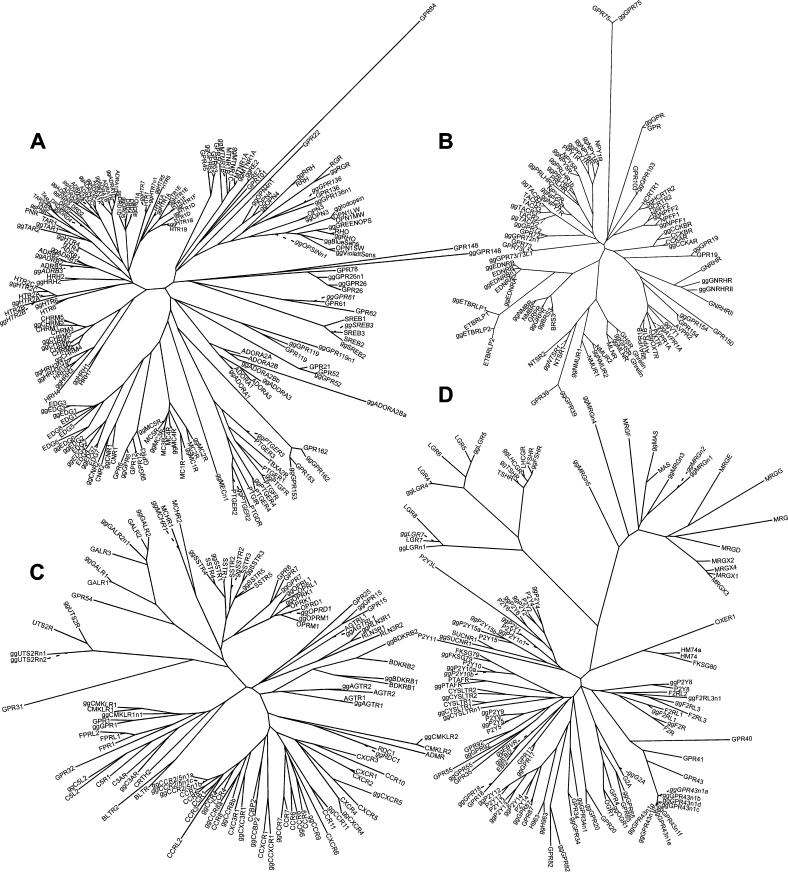
Phylogenetic Relationship between Human and Chicken GPCR Sequences Phylogenetic analysis was performed by first calculating neighbor-joining trees with 100 bootstrap replicas for each of the ten groups described in Table 1, and then mapping maximum-likelihood branch lengths onto the neighbor-joining topology using TreePuzzle. The trees were visualized in TreeView [94]. Dotted lines represent the position of a receptor protein with a partial TM region. These positions are based on a separate calculation. The *Rhodopsin* family receptors are shown. (A) The α-group of *Rhodopsin* family receptors. (B) The β-group of *Rhodopsin* family receptors. (C) The γ-group of *Rhodopsin* receptor family. (D) The δ-group of *Rhodopsin* receptor family.

Phylogenetic analysis was performed by first calculating neighbor-joining trees for each of the ten groups described in Table 1 (all except *Other*), and then mapping maximum-likelihood branch lengths onto the neighbor-joining topology using TreePuzzle. The topology for the *Adhesion* tree was calculated using maximium parsimony. The naming of the chicken receptors follows the guidelines of CHICKBASE hosted at the Roslin Institute (http://www.thearkdb.org) (see Figure 3). The definitions described in Figure 3 were used to classify the various possibilities of the phylogenetic relationships between the chicken and human GPCRs. The nomenclature for the human receptors follows, with a few exceptions, the guidelines from the International Union of Pharmacology Committee on Receptor Nomenclature and Drug Classification (NC-IUPHAR) [17]. The phylogenetic results are presented in Figures 4 and 5, while the large *Olfactory* tree is available as Dataset S1.

**Table 1:**
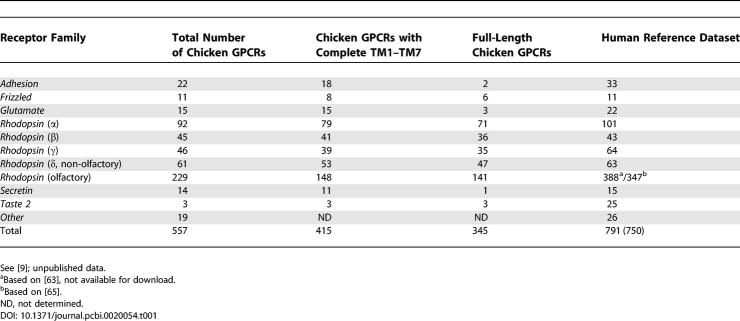
Summary of the Chicken GPCR Dataset in Relation to the Human Reference Dataset

In addition to the ten subgroups presented in the phylogenetic trees, 19 chicken GPCRs could not be classified into any of these subgroups. This is similar to the case with the human GPCRs, where 26 receptors could not be placed into any of the phylogenetic groups, and these are therefore placed into a group designated *Other.* It is important to note that the receptors in this group are, in general, not related to each other, although a few of these do have high sequence identity to one other receptor from the *Other* group. To assess possible sequence relationships between these and the human GPCRs, a BLAST database was built from the protein sequences of all the human GPCRs, and the 19 chicken GPCRs were compared against the database using BLASTP. In Table 2, we present the results from this analysis. It is interesting to note that one chicken GPCR, here designated ggNOVEL26, appears to lack clear similarity to any human GPCR using BLASTP alignments (cut off *E*-value > 10). We chose to include this clearly atypical GPCR as it shows similarity to the 7tm_1 model, the model derived from the *Rhodopsin* family of GPCRs, using the online version of RPS-BLAST. In step 4 (Figure 1), we use all atypical human GPCRs in low-stringency BLAST searches; the results of these low-stringency BLAST searches suggest that the majority of atypical GPCRs in chicken have been detected. We also searched with sequences from non-mammalian GPCR families. These were the cAMP-binding GPCRs from slime molds, the chemosensory GPCRs from nematodes, and the gustatory GPCRs from insects. We did, however, not find any GPCR from these families in the chicken.

**Table 2:**
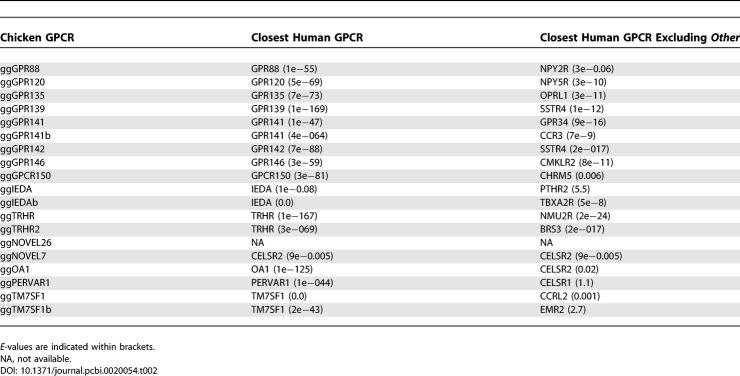
The Results of Pairwise Comparison of the Chicken GPCRs from the *Other* Group with the Human Dataset

Figure 4 describes the repertoire of chicken and human GPCRs in the *Adhesion* (Figure 4A), *Glutamate* (Figure 4B), *Frizzled* (Figure 4C), *Taste 2* (Figure 4D), and *Secretin* (Figure 4E) receptor families. In the phylogenetic tree (Figure 4A), the 22 chicken *Adhesion* GPCRs in the Ensembl February 2004 assembly of the chicken genome were compared to the 33 human *Adhesion* GPCRs (Table 1; [15]). None of the 22 chicken *Adhesion* GPCRs has previously been reported. Overall, there are 21 cases of one-to-one orthologous relationships between human and chicken *Adhesion* GPCRs, while 12 human *Adhesion* GPCRs lack a chicken ortholog. The adhesion family can be divided into groups I–VIII based on phylogeny [15]. Group I, which contains the lectomedin receptors (LEC1–LEC3) and the EGF-TM7-latrophilin–related protein (ETL) receptor, is relatively well conserved in the chicken, and only the LEC1 receptor is missing. Group II in the human consists of CD97 and four EGF-like modules containing mucin-like receptor proteins (EMR1–4) [15]. This group does not have any chicken orthologs. Since CD97 is present in the teleost Takifugu rubripes (unpublished data), this receptor appears to be have been lost in the lineage leading to the chicken, while the EMRs have probably expanded in mammals.

In group IV, the ortholog to human cadherin EGF LAG seven-pass G-type receptor 2 (CELSR2) is missing, while groups III and V are identical between the species regarding gene content. Group VI apparently has expanded in mammals or, more specifically, in humans. Chicken ggGPR111/115 may be the common ancestor of human GPR111 and GPR115 based on chromosomal localization and phylogenetic relationship. GPR111 and GPR115 are positioned in close proximity on human Chromosome 6p12.3 and may therefore be the result of a local duplication from the common ancestor GPR111/GPR115, still present as a single copy in the chicken. In group VII, there is a one-to-one relationship between the human and the chicken, while group VIII lacks a chicken ortholog to human GPR56.

Figure 4B describes the repertoire of chicken *Glutamate* family receptors. The chicken genome holds 15 *Glutamate* family members compared to the 22 human receptors [18]. Only one of the 15 chicken *Glutamate* family GPCRs has previously been reported in Genbank. The eight human metabotropic glutamate receptors (GRMs) can be divided into three different classes (type I–III) based on structural similarities, intracellular signaling, and pharmacological profile [19,20]. The GRMs are well conserved in the chicken compared to the human; only two human type III GRMs—GRM4 and GRM6—lack a chicken ortholog.

The *Taste 1* receptor family (TAS1R) consists in the human genome of three different GPCRs—TAS1R1, TAS1R2, and TAS1R3—which function as protomers in heterodimeric complexes [21–23]. Intriguingly, chickens appear to lack an ortholog to human TAS1R2. We also searched the chicken EST databases (http://www.chick.umist.ac.uk) for mRNA sequences representing the TAS1R2 receptor, but were unable to identify any sequence representing this receptor. The signaling combination TAS1R1/TAS1R3 is responsible for the l-glutamate taste (umami), whereas TAS1R2/TAS1R3 detects sweeteners [22,24]. The loss of TAS1R2 in the chicken was confirmed while this manuscript was under preparation [25].

The human genome contains a group of retinoic acid–inducible genes (RAIGs) or G protein–coupled receptor family C (GPRC) *GPRC5A–5D* [26–29]. The genes for these orphan receptors are well conserved in the chicken, although human GPRC5A and GPRC5D may represent a duplication in mammals of *GPRC5A/5D* that we found in chicken. This scenario is also strengthened by their localization in close proximity on the human Chromosome 12p13.2–p13.1, which is a syntenic region to chicken Chromosome 1 where *ggGPRC5A/D* is positioned [30].

The chicken appears to lack the ortholog for the human GABABR (gamma aminobutyric acid–binding receptor) 1. Studies have shown that human GABABR2 is unable to function without the GABABR1 unit, which is responsible for the ligand binding [28,31,32]. No EST sequences could be found for the GABABR1 receptor in the chicken (http://www.chick.umist.ac.uk); however, a search with the human GABABR1 revealed a short stretch of 23 identical amino acids matching an unlocalized chicken scaffold (chrUn: 96630977–96631045), which may represent a part of a GABABR1 ortholog, partially missing in this assembly. If this is not the case, GABABR receptors in the chicken could function in a very different mode compared with GABABR receptors in mammals, which would be interesting to explore further.

The *Frizzled* receptor family (FZD; Figure 4C) contains 11 members in the chicken as well as in the human. Of these, nine chicken receptor proteins were previously known from the literature. The family displays an almost 100% ortholog conservation between the human and the chicken. Only the human frizzled 5 (FZD5) lacks a clear chicken ortholog. However, chicken ggFZDn1 could have a different location in the phylogenetic tree if the sequence was full-length. The fact that the FZD is so well conserved in the chicken may be explained by the family's important role in basal cell functions such as controlling cell fate, proliferation, and polarity during metazoan development [33,34].

The human genome contains 25 functional human *Taste 2* receptors (T2Rs) responsible for detecting bitter-tasting compounds in addition to ten pseudo genes [35]. However, the chicken genome contains only three T2Rs (Figure 4D). Most of the human T2R genes are localized in clusters on Chromosomes 7q31 and 12p13 [35–40], while the three chicken T2R genes are not clustered. The identification of three T2R genes in the chicken was confirmed during the preparation of this manuscript [3,25].

Figure 4E describes the relationship between human and chicken *Secretin* family receptors. This family, like the *Frizzled* family, is distinguished by the high degree of one-to-one orthologs. The chicken genome contains 14 *Secretin* family receptors, while the human genome holds 15. Of these chicken genes, only four were previously known. The phylogeny shows that the ggCALCRn1 does not represent the chicken version of the human CALCR (the calcitonin receptor). It is, however, notable that these receptors are localized in syntenic regions (ggCALCRn1 on GG02; CALCR on Hsa7q21.3) which could indicate a close evolutionary relationship between the genes. Chickens appear to lack a counterpart to human GLP2R (glucagon-like peptide receptor 2), and the chicken *ggPTHR1* may be a pseudogene because the fourth exon is interrupted by a stop codon; no EST sequences could be found for chicken *ggPTHR1* that would contradict this hypothesis (http://www.chick.umist.ac.uk).

The *Rhodopsin* family of GPCRs has previously been divided into α-, β-, γ-, and δ-groups based on phylogeny [9]. The α-group consists of serotonin, dopamine, adrenergic, muscarinic, histamine, and trace amine receptors (TARs); endothelial differentiation G protein–coupled receptors (EDGRs); melanocortin, prostaglandin, and opsin receptors; and several orphan GPCRs. In humans, this group contains a total of 101 receptors, while we found 92 in the chicken genome (Figure 5A; Table 1). Several subgroups within the α-group are very well conserved. The melanocortin and EDGR have one-to-one orthologous pairs, while the prostaglandin receptor family (PTGxR) lacks a chicken ortholog for the human tromboxan receptor (TBXA2R), the prostaglandin E receptor 1, subtype EP1 (PTGER1), the prostacyclin receptor (PTGIR), and the prostaglandin D2 receptor (PTGDR).

The adenosine receptors (ADORAs) are relatively well conserved; however, human ADORA2B appears to be represented by two subtypes in the chicken, giving rise to ggADORA2Ba and ggADORA2Bb, where ggADORA2Ba appears to be evolving rapidly. All four chicken ADORAs have previously been cloned. In the databases, two unique sequences are named adenosine receptor 2B and, to avoid confusion, we have renamed those ggADORA2Ba and ggADORA2Bb according to our phylogeny. The chicken histamine receptor family (HRH) consists of ggHRH1, ggHRH2, and three human HRH3-like subtypes: ggHRH3, ggHRH3n1a, and ggHRH3n1b (Figure 5A). ggHRH3 is the ortholog of human HRH3, while the genes for ggHRH3n1a and ggHRH3n1b may represent a late local duplication in the chicken based on their close chromosomal localization on GG02. The chicken genome lacks the ortholog for the human muscarinic receptor 1 (CHRM1), while the adrenergic receptor family displays a one-to-one orthologous relationship between human and chicken receptors. This is also the case for the serotonergic receptor family, except for an extra copy of a HTR7-like subtype, ggHTR7n1, which possibly represents a subtype that was lost in humans.

The chicken genome contains five visual pigment genes, the rod pigment (rhodopsin) [41] and the four cone pigments—iodopsin (red) [42], blue-sensitive opsin, violet-sensitive opsin, and green-sensitive opsin [43]. As previously described, the human *OPN1LW* (red) and *OPN1MW* (green) are the result of a local duplication on the X chromosome [44]. Besides these already known genes, we identified a novel opsin-related gene in the chicken: *ggOPSINn1* localized basally in the opsin-cluster (Figure 5A). The ggOPN4n1 was previously called putative photopigment melanopsin but, based on the phylogenetic relationship within the melanopsin family (the OPN4s), we renamed it.

The TAR family has been subjected to different evolutionary events in different species; for example, several subtype-specific expansions both in zebrafish and rodent genomes. The human genome contains TAR1, TAR3, TAR4, TAR5, and PNR, while TAR2 is a pseudogene [45]. However, the TAR2 subtype seems to be present as a functional receptor both in rodents and chickens (Figure 5A) [45].

The β-group in the *Rhodopsin* family consists of 43 human receptors (unpublished data) and 45 potential chicken receptors (Table 1; Figure 5B). This group contains mainly peptide-binding receptors [9] such as the neuropeptide FF, neuropeptide Y, ghrelin, orexin, and cholecystokinin receptors, the neurotensin receptors (NTSRs), and the prolactin-releasing hormone receptors (PRLHRs). We expanded this group with 20 new chicken GPCRs, including orthologs for human NTSR1, tachykinin 2 and tachykinin 3 receptors (TACR2 and TACR3), the motilin receptor, and the neuromedin U subtype 1 and neuromedin U subtype 2 receptors (NMUR1 and NMUR2) (Figure 5B). The β-group contains as many as 37 one-to-one orthologous pairs between the human and chicken. However, the chicken genome appears to be missing an ortholog to the human NTSR2, while two NTSR subtypes are present in both human, mouse, rat, and bullfrog *(Rana ridibunda)* [46–48].

The chicken genome contains six neuropeptide Y receptor (NPYR) subtypes compared to four subtypes in human [49,50]. Besides NPY1R, NPY2R, NPY4R (PPYR1), and NPY5R—which both genomes contain—the chicken genome also contains NPY6R, homologous to human pseudogene *npy6r,* and NPY7R, homologous to zebrafish Npy7r [50,51]. NPY7R is most closely related to NPY2R, based on phylogeny, and appears to be an ancient relative to this gene, which is missing in mammals. Although *npy6r* is a pseudogene in the human, as well as in many other mammals, it is present as a functional receptor in the mouse, but appears to be missing completely in the rat [52]. Previous phylogenetic analysis of PRLHRs from fish, chicken, and mammals show that these receptors group with NPYRs with high bootstrap support [53]. Based on chromosomal localization and phylogenetic relationship, it was suggested that the tetraploidization events generated four copies of the ancient PRLHR gene—PRLHR1, PRLHR1b, PRLHR2, and PRLHR2b—which were differentially retained in different genomes. The mammalian genomes appear to contain only the PRLHR1 subtype, while the chicken genome contains PRLHR1, PRLHR1b, and PRLHR2, and the teleost genome from pufferfish contains the PRLHR1b and the PRLHR2b subtypes [53]. The chicken genome is missing the ortholog for the human orexin 1 receptor (HCRTR1), while the endothelin receptor family and the orphan GPR73 contain more subtypes than do the human families.

The γ-group contains receptors such as the chemokine, opioid, galanin, and somatostatin receptors [9]. The chicken genome contains 46 potential γ-group GPCRs, while the human genome contains 64 (Table 1; unpublished data). We expanded this group with 29 novel potential chicken GPCRs (Table 1). The main differences are in the chemokine receptor family, which has expanded in mammals, and in the formyl peptide-binding receptor family, which is missing in the chicken (Figure 5C). The human chemokine receptors are classified by their ligand-binding preferences [54], thereby generating four GPCR subclasses of chemokine receptors; the CCRs, the CXCRs, XCR1, and CX3CR1 [54].

Compared to the human genome, the chicken genome apparently has lost several receptors from the CXCR subclass (Figure 5C). This may also be an effect of a mammalian expansion in this subclass, since several of these receptors have been cloned in the mouse but not yet found in fish [55,56]. However, orthologs to human CXCR1 and CXCR2 have been found in *Xenopus laevis, Oncorhynchus mykiss,* and Cyprinus carpio [57–59]. The CCR subclass has expanded in the chicken genome (Figure 5C). There exist at least three chicken orthologs to human receptors CCR2 and CCR5; ggCCR2/5n1a, CCR2/5n1b, and ggCCR5n1c. In addition human CCR8 is represented by two chicken orthologs: ggCCR8a and ggCCR8b (Figure 5C). The chicken ortholog to human GPR8 is apparently missing (Figure 5C). This receptor has, in the human genome, been shown to bind neuropeptide W [60]. Only mammalian genomes appear to contain both subtypes, while zebrafish hold two GPR7/GPR8–like sequences, which may be the result of a linage-specific duplication of a gene ancestral to mammalian GPR7 and GPR8 (unpublished data) in fish.

The somatostatin receptors 1–5 display a clear one-to-one orthologous relationship (Figure 5C). The chicken genome appears to have lost the melanin-concentrating hormone receptor (MCHR) 2; both subtypes can be found in mammals and teleosts (MCHR1 in T. rubripes and MCHR2 in Danio rerio). The urotensin II receptor (UTS2R) has an ortholog in chicken, ggUTS2R; however, two additional receptor proteins, which resembled the human UTS2R, were found—ggUTS2Rn1 and ggUTS2Rn2 (Figure 5C). ggUTS2R and ggUTS2Rn2 are located in close proximity on chicken Chromosome 18; however, based on this phylogenetic analysis and the fact that both receptors have an ortholog in the teleost Tetraodon nigroviridis (unpublished data), it is not likely that they represent a late chicken-specific gene duplication (Figure 5C).

The fourth *Rhodopsin* family group, the δ-group, contains the olfactory receptors, the nucleotide-binding receptors, the glucoprotein receptors, several orphan receptors, and the mas-related G protein–coupled receptor (MRG) cluster 9 (Figure 5D). The glycoprotein receptors and the MRGs could have been placed in the γ- or δ-group because receptors from these groups hit other receptors from the γ- and δ-group with similar BLAST score. We chose, however, to place the glycoprotein receptors and the MRGs in the δ-group, based on phylogenetic topologies obtained using maximum-parsimony analysis [9]. We expanded the chicken δ-group with 52 novel GPCRs (Table 1). The largest differences between the receptors in the δ-group in the human and chicken are three species-specific expansions (Figure 5D). The first case deals with an expansion in the chicken genome which comprises a total of eight homologs to human orphan receptor GPR43 (Figure 5D). The International Chicken Genome Sequencing Consortium identified 13 GPR43-like sequences in their initial estimation [3]. At first, we also identified more than eight GPR43-like sequences, but after searches against the most recent assembly (http://genome.ucsc.edu/cgi-bin/hgBlat?command=start), the number of sequences was reduced to eight. The differences may be due to assembly problems in the unlocalized genomic regions where the GPR43 homologs are situated and, as a result, different assemblies may give different outcomes.

The second case regards the MRG cluster which, in the human genome, contains one mas-1 oncogene receptor gene and nine MRGs [61]. The chicken genome contains only one clear ortholog to this family, ggMAS (Figure 5D). However, the chicken genome also contains five other MRG-related genes. These genes may represent chicken-specific MRGs, as phylogenetic analysis groups these receptors separately when they are analyzed together with all human MRGs and members from mouse and rat mrgA, mrgB, and mrgC receptor families (unpublished data) (for nomenclature see [61,62]).

The third case is the olfactory receptors. The human genome holds 388 functional olfactory receptors [63,64], while the chicken genome contains at least 229. A majority of the chicken olfactory receptors (*n* = 218) represent an expansion of genes similar to the human *01.01.01/OR5BF1* gene [3] (for nomenclature see [64,65]). However, the eight functional chicken olfactory genes that have been cloned so far—*COR1–6, COR7a,* and *COR7b* [66]—are all localized in close proximity on chicken Chromosome 5 and are not part of that expansion. Instead, receptors COR1–6 form a separate sub-tree close to group 11.31.01–11.31.05 of human olfactory receptors, all tightly positioned together on human Chromosome 11 (for phylogenetic tree, see Dataset S1; for nomenclature, see [65]). Based on their phylogenetic relationship, the chicken COR1–6 and human 11.31.01–11.31.05 may share a recent common ancestor. In addition to these six chicken receptors, COR7a, COR7b, and the novel receptors ggOR62, ggOR220, and ggOR221 also position outside the 01.01.01/OR5BF1 gene expansion group. COR7a, COR7b, and ggOR220 group close to human olfactory receptors 11.47.01–11.47.03, while ggOR62 and ggOR221 group close to human receptors 11.44.01 and 15.02.01, respectively. All 229 chicken olfactory receptors represent unique genomic positions in the chicken assembly because Genscan was set to exclude predicted alternative transcripts.

All 557 reported chicken receptors can be found in Dataset S2 (description) and Dataset S3 (sequences).

## Discussion

In this paper, we present a collection of 557 manually curated GPCR sequences from the chicken genome. The sequences were obtained through a four-step search procedure with a high degree of manual verification, and it is likely that this dataset contains most of the GPCR sequences present in the current assembly of the chicken genome. The aim of both the manual curation and the assembly step are to ensure that all pseudogenes, i.e., genes with coding regions interrupted by stop-codons, are excluded and that the exon–intron organizations are correct. It is well established that automatic prediction and annotation of proteins from genomic sequences is highly error-prone [6]. We show that only 62 out of the 158 non-olfactory chicken GPCRs with a clear orthologous relation to a human GPCR were correctly predicted by Genscan (41%) (Figure 2A). The manually corrected sequences differ, on average, by 19% from the original Genscan predictions. To show the significance of this fact with regard to subsequent analysis, we calculated the sequence identity between pairwise alignments of orthologous chicken and human sequence pairs for the corrected and uncorrected chicken GPCRs, and here again the difference is remarkable. We found that the average sequence identity was 56.3% between the human and non-edited chicken GPCRs, while the average sequence identity between the corresponding edited chicken GPCRs and the human sequences was 72.9% (Figure 2B). Because it is highly unlikely that Genscan errors make orthologs more similar, comparison between these numbers suggests that our manual curation has clearly improved the dataset.

It is interesting to note that in a study by Hillier et al. describing the initial annotation of the chicken genome, the overall average sequence identity between 10,094 protein sequences orthologous between the human and the chicken was found to be around 80% [3]. It has to be noted that these sequences are all computer predictions on a draft genome assembly. The percentage identity could be even higher if it was based on curated sequences using the same reasoning as above. However, the dataset used by Hillier et al. contains only “core orthologs”, i.e., sequences that are conserved between the human, chicken, and *Fugu*. It is possible that the automatic procedure used to obtain these “core” sequence predictions has enriched the dataset for highly conserved sequences.

Our results suggest that the orthologous GPCRs are, in general, less well conserved between the human and the chicken than the average protein. This is also indicated by data presented by Hillier et al. where the gene ontology (GO) category “GPCR-signaling”, containing 323 orthologous pairs, was the 16th least-conserved category at primary-sequence level, out of 20 categories from the biological process GO-tree [3]. According to the definition, this GO-class contains, apart from GPCRs, other proteins associated with the signaling cascade of GPCRs. Assuming that there are around 250 orthologous pairs representing GPCRs in this category (which is approximately what we identified), other proteins—such as G-proteins, peptides, and enzymes—constitute around 33% of the proteins in this GO category. Many of these proteins, for example the G-proteins, are generally known to be well conserved between species [67,68]. Taken together, these data suggest that GPCRs, even when confounding factors such as gene duplications, expansions, and deletions are considered, evolve more rapidly than most other protein families.

GPCRs constitute 3.2% and 5.2% of the genes in the human and mouse genomes, respectively, considering that both these genomes have about 25,000 protein-coding genes [4,5]. The main difference in the GPCR repertoire between the mouse and the human genomes can be attributed to gene expansions of olfactory receptors in mouse. The initial estimates of the chicken genome indicate that it also contains about 20,000–23,000 protein-coding genes [3], and considering the number of GPCRs that we find, the overall percentage of GPCR is lower in chickens or between 2.2% and 2.4%. This difference can be explained by the fact that chicken has a lower number of olfactory receptors. If the olfactory receptors are excluded, the overall percentage of GPCR genes is 1.65% for the human genome and between 1.32% and 1.43% for chicken. We have previously shown that the percentages of all protein predictions that are GPCR sequences are, in general, similar in different vertebrates as well as in invertebrates [7]. The only large deviation between species was found to be related to large expansions of certain GPCR families, interestingly always receptors for non-endogenous ligands. Examples are the chemosensory receptors in the nematode *(Caenorhabditis elegans),* gustatory receptors in insects, and olfactory receptors in the mouse [7]. The overall percentage of GPCRs for endogenous ligands is thus remarkably constant for all the bilateral species investigated in detail so far.

Our phylogenetic analysis with all known human and chicken GPCRs is the first detailed comparison between the repertoire of GPCRs in non-mammalian and mammalian species. This analysis shows the orthologous pairs of the chicken and human GPCRs, and we conclude that all the main groups of GPCRs, with the exception of *Taste 2* and the olfactory subset of the *Rhodopsin* γ-group are, in general, well conserved between the human and chicken. The average sequence identity between orthologous pairs of chicken and human proteins is around 73% in their TM regions, but there is a considerable variation in identity between the different families and groups of GPCRs. The *Adhesion* family displays the lowest percentage identity (68.8%) between orthologous pairs, and this could be due to the fact that the *Adhesion* GPCRs utilize the TM regions mostly as a membrane-anchor and signal-transmission unit, and not primarily for complex ligand interactions.

FZD is well conserved between the human and the chicken, regarding both repertoire and primary sequence (81.4%), which could relate to their important role in basic functions such as controlling cell fate, proliferation, and polarity during metazoan development [33,34]. In fact, FZD is the only GPCR family that is close to the figure of 80% conservation that is reported as the mean value for the entire set of orthologous pairs in the chicken and the human genomes [3]. It is also interesting to note that the proteins in the *Rhodopsin* family β-group appear to evolve more slowly (77.3%) than the other *Rhodopsin* groups (69.2%–73.8%); this could be due to the fact that all ligands in this family are peptides and that peptide ligands may require more interaction points than smaller non-peptidergic substances—which is likely to conserve the structure and thus the amino acid sequence of the *Rhodopsin* β-group receptors.

According to our phylogenetic analysis, 259 of the 557 chicken receptors have a one-to-one ortholog in the human genome. It is, however, important to note that comparison of only two genomes may provide some wrong conclusions about orthologous relationships in individual cases. This is because deletions of one member in each of two related pairs in both species (double loss) may cause topology that wrongly indicates those genes that are orthologs. We used data from the rodent or fish genomes to clarify in more detail the true phylogenetic relationship in cases where no clear one-to-one orthologous relationships were present—for example TAR2 and NPY6R, which are both pseudogenes in the human but are functional in the mouse. Overall, the orthologous pairs for many of the GPCR groups are, in general, remarkably well conserved between the human and the chicken, despite the fact that this family of proteins appears to evolve relatively fast considering the primary sequence. For example, the *Secretin* and the *Frizzled* families display a one-to-one human–chicken orthologous relationship for all but one protein in each of the two families (Figure 4C and 4E). However, the repertoire of GPCRs that contribute to the sensory systems such as smell, taste, and vision differ remarkably between the chicken and the human genomes.

Olfaction is mediated by GPCRs expressed in the olfactory epithelium, and it is one of the major neurosensory functions by which vertebrates such as humans and chickens investigate their external chemical environment [69]. The 388 functional olfactory receptors in the human genome can be divided into class I and class II based on phylogenetic criteria [63,64]. The chicken genome contains at least 229 potentially functional class II olfactory receptors, while class I receptors appear to be missing. Class I olfactory receptors are present both in teleosts and in mammals, and have long been considered to recognize water-soluble odorants, while class II receptors mediate the effects of airborne odorants [64,70]. A majority of the chicken olfactory receptors, 218 genes, represent an expansion of genes most similar to the human 01.01.01/OR5BF1 gene in class II [3] (for nomenclature, see [64,65]). It is not known which ligands these novel proteins recognize, but it is likely that these ligands are some kind of volatile airborne substance. Several studies have addressed the deficient homing ability in anosmic birds; these studies indicate that birds use the olfactory system for navigation, particularly in unknown terrain [71–73]. Birds apparently also use the olfactory system for discriminating between individuals and finding their nests, for finding food, and for avoiding toxic insects and dangerous predators [74–76]. One possibility could be that the large number of class II olfactory receptors could be involved in such functions.

The gustatory system in humans can detect and differentiate between hundreds of compounds, allowing us to avoid toxic compounds and to select nutritious food [77]. Three of the five taste modalities—sweet, bitter, salt, sour, and l-glutamate (umami)—are mediated through GPCRs. Sweet and umami are mediated by the TAS1Rs which, in humans, consist of three different GPCRs—TAS1R1, TAS1R2, and TAS1R3 [21–23]—while bitter taste is mediated by the *Taste 2* receptor family (TAS2R) [35–37]. The TAS1Rs function as protomers in heterodimeric complexes [21–23], where the dimer complex between TAS1R1 and TAS1R3 is responsible for the l-glutamate taste (umami), whereas the combination of TAS1R2 and TAS1R3 detects sweeteners [22,24]. Intriguingly, chickens appear to lack an ortholog to human TAS1R2. This may imply that the chicken's mode of detecting sweet taste differs from that of humans, since the TAS1R2 unit, which is missing in the chicken, is the interaction point for sweet-tasting compounds such as aspartame and neotame, while the TAS1R3 unit is responsible for the intracellular signaling [78]. However, sweet compounds such as lactisole, brazzein, and cyclamate have been shown to interact directly with the TAS1R3 unit [78–80], which could implicate a sweet-detecting ability despite the lack of a TAS1R2 unit.

The human genome contains 25 functional genes that code for T2Rs, which are responsible for detecting bitter-tasting compounds [35]. Intriguingly the chicken genome contains only three bitter-tasting T2Rs (Figure 4D). The closest human homolog to the novel ggT2Rn1 binds β-glucopyranosides [38], while the closest human relatives to the novel chicken genes ggT2Rn2 and ggT2Rn3 are still orphans. The large sequence diversity among the 25 human T2Rs may explain how a limited number of receptors can sense the thousands of bitter compounds that humans can detect [81], while in the chicken, the low number of T2Rs may indicate a relatively poor ability of chickens to select between bitter compounds.

Humans are trichromatic i.e., have the ability to discriminate between three different colors (wavelengths), while the chicken is tetrachromatic. The chicken genome contains five visual pigment genes; the rod pigment (rhodopsin) [41] and the four cone pigments—iodopsin (red) [42], blue-sensitive opsin, violet-sensitive opsin, and green-sensitive opsin [43]. Besides these already known genes, we have identified a novel opsin-related gene in the chicken—ggOPSINn1 localized basally in the phylogenetic opsin-cluster (Figure 5A). This gene is closely related to the two forms of vertebrate ancient opsins (short and long) previously found in the zebrafish and roach [82,83]. The long form in zebrafish has been found to function as a green-sensitive pigment, and immunoreactivity towards this splice variant has been detected in non-GABAergic horizontal cells in the zebrafish and roach retinas, and in cells surrounding the zebrafish diencephalic ventricle of thalamus, suggesting multiple roles in photosensory physiology [82,83].

In summary, we scanned the recently sequenced chicken genome for GPCRs to obtain manually edited and verified coding regions of a total of 557 GPCRs. To our knowledge, this provides the first high-quality collection of GPCR sequences from a full genome of a non-mammalian species. Our phylogenetic analysis on the curated chicken GPCRs, together with 750 human GPCRs, clarifies the differences between the GPCR repertoires that may relate to the functional differences between these two species. Our curated GPCR dataset from the chicken genome could serve as a basis for annotating this important protein family in other vertebrates, as well as in invertebrates.

## Materials and Methods

### Generation of a Genscan dataset.

A set of in silico–predicted chicken genes was obtained from the February 2004 genome assembly by the following procedure. The chromosomal files were divided into smaller files of 2 MB. We scanned each of these files with Genscan [84], using the human parameter file HumanIso.smat, because no Genscan dataset was available for download at that point. Thereafter, we gathered all the complete predictions into a unique set of predicted chicken genes.

### Identification of GPCRs from the Genscan dataset using BLASTP (step 1).

An “in house” GPCR dataset consisting of 403 human non-olfactory receptors ([9,15,85–87]; unpublished data) was searched against a database built from the chicken Genscan dataset using BLAST. All hits with an *E*-value of better than 0.1 were extracted into a temporary file. From this file, all duplicates, based on the Genscan number, were automatically removed using a custom made C++ program (available upon request). From the resulting single-copy set, the sequences that were true GPCRs were extracted by searching those against a database consisting of the entire RefSeq database [88]—all human non-olfactory GPCRs and 347 human olfactory GPCRs [65]. The chicken Genscan sequences that did not hit any of the human GPCRs among the five top hits in a BLASTP search with a cut-off at *E* = 10 were classified as non-GPCRs and removed. This resulted in 870 putative predicted chicken GPCRs. These were tentatively annotated by searching every hit against a database consisting of all human GPCRs using BLASTP with a cut-off at *E* = 10, with subsequent naming according to the most significant human hit with a gg (for G. gallus) prefix. During manual editing, we found that many chicken GPCRs had to be built from several (sometimes more than 30) different predictions, which each contained only a small part of the final chicken GPCR sequence. After manual editing, 390 chicken GPCR sequences remained.

### Identification of GPCRs from the chicken genome using TBLASTN (step 2).

A set of chicken GPCRs that were not found in the Genscan dataset was obtained in the following way. The genomic position was identified for 505 putative chicken GPCRs by aligning its sequence to the genome assembly with BLAT 3.0 [89] and defining the highest scoring alignment as the position for each gene. In a similar way, best-in-chicken genome positions were identified for each human GPCR sequence using translated BLAST (TBLASTN) with a cut-off at 1e−6 [90]. The set of putative new chicken GPCRs was then identified as being the positions in the chicken genome that had an alignment with a human GPCR that was not overlapped by any chicken GPCR. The genomic material aligning to the human GPCR was downloaded, and a final version of each of the chicken GPCRs was manually assembled and edited.

### Identification of missing GPCRs from initial phylogenetic analysis (step 3).

An initial phylogenetic analysis was performed as described below, with the longest possible version of all sequences from each family and group, using both neighbor-joining and maximum-parsimony analysis. From these trees, all cases of missing orthologous GPCRs in the chicken, compared with the human, dataset were identified. This resulted in a dataset of human GPCRs consisting of eight *Glutamate,* 24 *Rhodopsin* (α), seven *Rhodopsin* (β), 21 *Rhodopsin* (γ), 14 *Rhodopsin* (δ), and three *Secretin* GPCRs. This dataset, consisting of, in total, 77 human GPCRs, was searched against the sequence of the entire chicken genome using TBLASTN. All hits with an *E*-value of better than *E* = 1e−6 were manually compared against the chicken GPCRs that had so far been collected. All new sequences were collected and subjected to manual assembly and verification.

### Identification of residual GPCRs from the chicken genome (step 4).

A set of human GPCRs consisting of one human sequence from each of the *Secretin, Glutamate, Adhesion, Taste2,* and *Frizzled* families, together with one sequence from each of the 13 subgroups of the *Rhodopsin* family [9] were defined. These were combined with all human sequences from the *Other* group into a dataset of 47 human GPCR sequences. This dataset was searched against the sequence of the entire chicken genome using TBLASTN. All hits with an *E*-value of better than *E* = 0.1 were manually compared against the chicken GPCRs that were collected so far by literal word searches using a bash script. Stretches of 20 amino acids from each hit were used. All new sequences were collected and subjected to manual assembly and verification.

### Manual curation of chicken GPCRs.

All tentative chicken GPCRs were manually assembled, corrected, and verified. This was done using EditSeq and MegAlign from the DNASTAR package (DNASTAR, Madison, Wisconsin, United States), EMBOSS [16], ClustalW [91], and the web-based services BLAST [90] and BLAT [89]. For BLAST and BLAT, we also used standalone versions with local databases with these tools. All chicken proteins were edited under the following assumptions. (1) All splice sites are of the canonical (GT-AG) type. (2) The position of the splice sites, and hence the organization of exons and introns, are in general conserved between chicken and human orthologs. (3) When, in a small region, there were several possible splice sites that fulfill points 1 and 2, the one that gave an amino acid alignment most similar to the human ortholog was chosen. (4) The end of each predicted coding region was chosen as the first stop codon in the correct frame in the last exon. (5) The start codon was chosen as the methionine in the correct frame in the first exon that gave the best alignment to the human ortholog. In a few cases, an exon–intron boundary is clearly different between chicken and human and, in these cases, we attempted to identify that exon by translated alignments between the human ortholog and the smallest possible region in the chicken genome that could contain that exon. All genes were corrected and assembled at the DNA level, and in the final step were translated into an amino acid sequence to ensure that the correct reading frame is maintained along the entire coding region.

### Semi-automatic verification of the dataset.

To ensure that all sequences identified in this process were truly identical to the chicken genome, all sequences were aligned against the genome using the Windows version of BLAT 3.0 [89]. The resulting psl-file was parsed using a JAVA program, and only the highest scoring alignment was kept. This table was inspected manually, and all alignments with less than 100% identity were identified and manually inspected in more detail. The majority of these turned out to be alignment errors produced by BLAT, something that occurs relatively frequently [92]. The other sequences were corrected unless (1) the sequence was known before, in which case the GenBank sequence (http://www.ncbi.nlm.nih.gov) was used, or (2) it appeared to be a polymorphic site that changes between assembly versions of the chicken genome.

### Phylogenetic analysis.

The chicken GPCRs were first divided into families and groups by BLAST searches with the sequence against the human GPCR dataset. The accession numbers for all human sequences used for the phylogenetic analysis can be found in Dataset S4. The olfactory receptor sequences are as described originally [65], and hence the sequences in the NCBI database varies for some, as noted in Dataset S4. The sequences were categorized based on the family identity of the first five human hits. A sequence was placed in *Other* if the sequence hit two different families or groups. The edited and verified chicken and human GPCRs from each group were combined into a FASTA file and aligned using the UNIX version of ClustalW 1.82 [91]. The default alignment parameters were applied. The alignment was bootstrapped 100 times using SEQBOOT from the Win32 version of the Phylip 3.6 package [93], and the same bootstrapped alignment was used for all subsequent calculations. For neighbor-joining trees, protein distances were calculated on the bootstrapped alignments using PROTDIST from the Win32 version of the Phylip 3.6 package to obtain, in total, 100 distance matrixes. The Jones–Taylor–Thornton matrix was used. Trees were calculated on the distance matrixes using NEIGHBOR from the Win32 version of the Phylip 3.6 package, resulting in 100 trees. Majority-rule consensus trees were constructed using CONSENSE from the Win32 version of the Phylip 3.5 package. The trees were plotted using TreeView [94].

Maximum-parsimony trees were calculated from the same bootstrapped alignment as used for distance trees with PROTPARS from the Win32 version of the Phylip 3.6 package. The trees were un-rooted and calculated using ordinary parsimony, and the topologies were obtained using the built-in tree-search procedure. Consensus trees were calculated and plotted as described above. For the maximum-likelihood trees, the topology obtained from the maximum-parsimony or neighbor-joining trees was used as a user-defined tree in TreePuzzle [95], and clock-like branch lengths were estimated in TreePuzzle using the following parameters. *Type of analysis:* Tree reconstruction; *Tree-search procedure:* User-defined trees; *Compute clock-like branch lengths:* Yes; *Location of root:* Best Place (automatic search); *Parameter estimates:* Exact (slow); *Parameter-estimation uses:* 1st input tree; *Type of sequence input data:* Amino acids; *Model of substitution:* VT (Mueller–Vingron Model of Substitution, 2000); *Amino acid frequencies:* Estimate from dataset; *Model of rate heterogeneity:* Mixed (one invariable plus eight Gamma rates); *Fraction of invariable sites:* Estimate from dataset; *Gamma distribution parameter alpha:* Estimate from dataset; *Number of Gamma rate categories:* eight.

### Global pairwise alignments.

Global pairwise alignments for calculation of percentage identity between two sequences were constructed and scored automatically using a bash-script that utilized ClustalW [91] as alignment engine and infoalign from the EMBOSS 2.8.0 package [16] for scoring, i.e., calculating the percentage of identical amino acids. All statistical analysis was performed using MiniTab (http://www.minitab.com). Graphs were plotted using Microsoft Excel (http://www.microsoft.com) and MiniTab.

## Supporting Information

Dataset S1 The Olfactory Phylogenetic Tree in Standard Newick Format (12 KB TXT)

Dataset S2 Table Indicating Completeness and Protein Name of All Chicken GPCR Identified in This Study. Classification and Step in which They Were Identified in the Search Procedure is Included (87 KB PDF)

Dataset S3 Amino Acid Sequences of All Chicken GPCRs Identified in This Study in Standard Fasta Format (132 KB PDF)

Dataset S4 Table with Accession Numbers to All Human Protein Sequences Used in This Analysis (19 KB PDF)

Table S1 Summary of the Process of Identification of Chicken GPCRs and of the Steps in which Receptors from the Respective Groups Were Identified (59 KB PDF)

## Abbreviations

ADORA: adenosine receptor
CALCR: calcitonin receptor
CHRM1: human muscarinic receptor 1
EDGR: endothelial differentiation G protein–coupled receptor
EMR: mucin-like receptor protein
ETL: EGF-TM7-latrophilin–related protein
FZD: *Frizzled* receptor family
GABABR: gamma aminobutyric acid–binding receptor
GLP2R: glucagon-like peptide receptor 2
GO: gene ontology
GPCR: G protein–coupled receptor
GPRC: G protein–coupled receptor family C
GRM: human metabotropic glutamate receptor
HCRTR1: human orexin 1 receptor
HRH: histamine receptor family
LEC: lectomedin receptor
LN: long N-termini
MCHR: melanin-concentrating hormone receptor
MRG: mas-related G protein–coupled receptor
NC-IUPHAR: International Union of Pharmacology Committee on Receptor Nomenclature and Drug Classification
NMUR1: neuromedin U subtype 1 receptor
NMUR2: neuromedin U subtype 2 receptor
NPYR: neuropeptide Y receptor
NTSR: neurotensin receptor
PRLHR: prolactin-releasing hormone receptor
PTGIR: prostacyclin receptor
PTGxR: prostaglandin receptor family
RAIG: retinoic acid–inducible gene
T2R: *Taste* 2 receptor
TACR2: tachykinin 2 receptor
TACR3: tachykinin 3 receptor
TAR: trace amine receptor
TAS1R: *Taste* 1 receptor family
TAS2R: *Taste* 2 receptor family
TBXA2R: human tromboxan receptor
TM: transmembrane
UTS2R: urotensin II receptor
