# Supplementary material for: The G Protein–Coupled Receptor Subset of the Chicken Genome
Source: PLoS Comput Biol. 2006 Jun 2;2(6):e54. doi: 10.1371/journal.pcbi.0020054 (PMC1472694; doi:10.1371/journal.pcbi.0020054)
Supplement: Supplementary file 1 [file pcbi.0020054.sd002.pdf]

Gene Name	Accesion number	Familly	Group	Found In Step	Complete TM-regions	Complete/ partial cds	Comment
ggBAI1	NA	Adhesion		1	Y	partial	missing 17:th exon (GPS-motif) due to poor genomic sequence
ggBAI2	NA	Adhesion		1	N	partial	missing extracellular region completely, missing parts of tm-regions and cytosolic part due to poor genomic sequence
ggBAI3	NA	Adhesion		1	Y	partial	missing one exon in extracellular part and one exon in cytosolic part due to poor genomic sequence
ggCELSR1	NA	Adhesion		1	Y	partial	missing exons in extracellular part and one exon in cytosolic part
ggCELSR3	NA	Adhesion		1	Y	partial	missing exons in extracellular part and one exon in cytosolic part
ggETL	NA	Adhesion		1	Y	complete	
ggGPR112	NA	Adhesion		1	Y	partial	missing extracellular region completely, missing the last two exons in cytosolic part
ggGPR114	NA	Adhesion		4	N	partial	missing parts of extracellular region, transmembrane region and the last exon in the cytosolic part
ggGPR115	NA	Adhesion		1	Y	partial	missing part of extracellular region and the last exon in cytosolic part
ggGPR116	NA	Adhesion		1	Y	partial	missing part of extracellular region and the last exon in cytosolic part
ggGPR123	NA	Adhesion		1	Y	partial	missing exon 1-11
ggGPR124	NA	Adhesion		1	Y	partial	missing exon 1-7
ggGPR125	NA	Adhesion		1	Y	partial	missing exon 1
ggGPR126	NA	Adhesion		1	Y	partial	missing start Met
ggGPR128	NA	Adhesion		4	Y	partial	missing parts of extracellular region and cytosolic region
ggGPR133	NA	Adhesion		4	Y	partial	missing part of extracellular region and part of cytosolic region
ggGPR144	NA	Adhesion		4	Y	partial	missing part of extracellular region and part of cytosolic region
ggGPR97	NA	Adhesion		4	N	partial	missing parts of extracellular region and cytosolic region
ggHE6	NA	Adhesion		2	N	partial	Missin parts of extracellular region and two internal exons
ggLEC2	NA	Adhesion		1	Y	complete	
ggLEC3	NA	Adhesion		1	Y	partial	missing exon 1 and 7
ggVLGR1	NA	Adhesion		1	Y	partial	missing exon 26-27
ggFZD1	O57328	Frizzled		Known	Y	complete	
ggFZD10	NP_989429	Frizzled		Known	Y	complete	
ggFZD2	NP_989553	Frizzled		Known	Y	complete	
ggFZD3	NA	Frizzled		1	Y	complete	
ggFZD4	NP_989430	Frizzled		Known	Y	complete	
ggFZD6	AAQ24762	Frizzled		Known	N	partial	missing part of extracellular region, transmembrane region and part of cytosolic region
ggFZD7	NP_989552	Frizzled		Known	Y	complete	
ggFZD8	NA	Frizzled		1	N	partial	missing part of extracellular region, transmembrane region and part of cytosolic region due to poor genomic sequence
ggFZD9	Q9IA02	Frizzled		Known	Y	partial	missing part of extracellular region and part of cytosolic region
ggFZDn1	BAA89403	Frizzled		Known*	N	partial	missing part of extracellular region, transmembrane region and part of cytosolic region due to poor genomic sequence
ggSMO	O42224	Frizzled		Known	Y	partial	missing part of exon 1 due to poor genomic sequence
ggCASR	NA	Glutamate		1	Y	partial	missing exon 3
ggGABBR2	NA	Glutamate		1	Y	partial	missing exon 1
ggGPRC5A/D	NA	Glutamate		2	Y	partial	Missing last two exons
ggGPR158	NA	Glutamate		2	Y	partial	missing exon 1
ggGPRC5B	NA	Glutamate		1	Y	partial	missing last exon
ggGPRC5C	NA	Glutamate		1	Y	partial	missing last two exons
ggGPRC6A	NA	Glutamate		1	Y	complete	
ggGRM1	NA	Glutamate		1	Y	partial	missing parts of extracellular region and cytosolic region
ggGRM2	NA	Glutamate		3	Y	partial	missing parts of extracellular region and cytosolic region
ggGRM3	NA	Glutamate		2	Y	partial	missing the last two exons
ggGRM5	AAK01487.1	Glutamate		Known	Y	complete	
ggGRM7	NA	Glutamate		3	Y	partial	missing exon 1 and part of exon 2
ggGRM8	NA	Glutamate		1	Y	partial	missing last exon
ggTAS1R1	NA	Glutamate		1	Y	partial	missing exon 1 and exon 2
ggTAS1R3	NA	Glutamate		1	Y	complete	
ggGPCR150	NA	Other		1	ND	NA	
ggGPR120	NA	Other		3	ND	partial	missing first and last exon
ggGPR135	NA	Other		1	ND	partial	Missing first part due to poor genomic sequence
ggGPR139	NA	Other		1	ND	complete	
ggGPR142	NA	Other		1	ND	NA	
ggGPR88	NA	Other		1	ND	NA	

ggIEDA	NP_989993.1	Other		Known	ND	NA	
ggNOVEL26	NA	Other		4	ND	NA	
ggNOVEL7	NA	Other		1	ND	NA	
ggPGR10	NA	Other		4	ND	NA	
ggTM7SF1	NP_00102623	Other		Known	ND	NA	
ggTM7SF1b	NA	Other		1	ND	NA	
ggTRHR	NP_990261.1	Other		Known	ND	complete	
ggTRHR2	NA	Other		1	ND	partial	missing part of transmembrane region and cytosolic part due to poor genomic sequence
ggOA1	NA	Other		4	ND	NA	missing last exon
ggPERVAR1	NA	Other		4	ND	partial	Missing first part due to poor genomic sequence
ggGPR141a	NA	Other		1	ND	complete	
ggGPR141b	NA	Other		1	ND	complete	
ggGPR146	NA	Other		3	ND	partial	missing part of transmembrane region due to poor genomic sequence
ggADORA1	NP_989647.1	Rhodopsin	A	Known*	Y	complete	
ggADORA2Ba	AAM46927.1	Rhodopsin	A	Known*	Y	complete	
ggADORA2Bb	NP_990418.1	Rhodopsin	A	Known	Y	complete	
ggADORA3	NP_989482.1	Rhodopsin	A	Known	Y	complete	
ggADRA1A	NA	Rhodopsin	A	1	Y	complete	
ggADRA1B	NA	Rhodopsin	A	3	Y	partial	unclear start of last exon
ggADRA1D	NA	Rhodopsin	A	1	Y	complete	
ggADRA2A	NA	Rhodopsin	A	1	N	partial	partial due to poor genomic sequence
ggADRA2B	NA	Rhodopsin	A	4	Y	complete	
ggADRA2C	NA	Rhodopsin	A	1	Y	partial	partial due to poor genomic sequence
ggADRB1	NA	Rhodopsin	A	1	Y	complete	uncertain about the extra exon in the end of the gene
ggADRB2	NA	Rhodopsin	A	3	Y	complete	
ggADRB3	NA	Rhodopsin	A	1	N	partial	missing part of extracellular region and cytosolic region
ggBlueSensitiveOpsin	NP_990848.1	Rhodopsin	A	Known	Y	complete	
ggCHRM2	A40972	Rhodopsin	A	Known	Y	complete	
ggCHRM3	NP_990730.1	Rhodopsin	A	Known	Y	complete	
ggCHRM4	P17200	Rhodopsin	A	Known	Y	complete	
ggCHRM5	AAF19027.1	Rhodopsin	A	Known	Y	complete	
ggCNR1	NA	Rhodopsin	A	1	Y	complete	
ggCNR2	NA	Rhodopsin	A	2	Y	complete	
ggDRD1A	A55886	Rhodopsin	A	Known	Y	complete	
ggDRD1D	C55886	Rhodopsin	A	Known	Y	complete	
ggDRD2	NA	Rhodopsin	A	3	Y	complete	
ggDRD4	NA	Rhodopsin	A	1	N	partial	missing part of extracellular region, transmembrane region and part of cytosolic region
ggDRD5	NA	Rhodopsin	A	1	Y	partial	missing start Met due to poor sequence
ggEDG1	NA	Rhodopsin	A	1	Y	complete	
ggEDG2	NA	Rhodopsin	A	1	Y	complete	
ggEDG3	NA	Rhodopsin	A	1	Y	complete	
ggEDG4	NA	Rhodopsin	A	1	N	partial	partial due to poor genomic sequence
ggEDG6	NA	Rhodopsin	A	1	Y	complete	
ggEDG7	NA	Rhodopsin	A	1	Y	complete	
ggGPR119n1	NA	Rhodopsin	A	1	Y	complete	
ggGPR12	NA	Rhodopsin	A	2	Y	complete	
ggGPR136	NA	Rhodopsin	A	1	N	partial	missing part of transmembrane region due to poor genomic sequence
ggGPR136n1	NA	Rhodopsin	A	1	Y	complete	
ggGPR148	NA	Rhodopsin	A	1	Y	complete	
ggGPR153	NA	Rhodopsin	A	2	Y	partial	missing last two exons
ggGPR162	NA	Rhodopsin	A	2	Y	complete	
ggGPR26	NA	Rhodopsin	A	1	Y	complete	
ggGPR26n1	NA	Rhodopsin	A	1	Y	complete	
ggGPR52	NA	Rhodopsin	A	1	Y	complete	
ggGPR6	NA	Rhodopsin	A	1	Y	partial	missing start Met
ggGPR61	NA	Rhodopsin	A	1	N	partial	missing N-term due to Ns
ggGPR63	NA	Rhodopsin	A	1	Y	complete	

ggMECn1	NA	Rhodopsin	A	1	Y	complete	
ggGREENOPSIN	NP_990821.1	Rhodopsin	A	Known	Y	complete	
ggHRH1	NA	Rhodopsin	A	3	Y	complete	
ggHRH2	NA	Rhodopsin	A	1	Y	complete	
ggHRH3	NA	Rhodopsin	A	1	Y	complete	
ggHRH3n1a	NA	Rhodopsin	A	1	Y	complete	
ggHRH3n1b	NA	Rhodopsin	A	1	Y	complete	
ggHTR1A	NA	Rhodopsin	A	1	Y	complete	
ggHTR1B	NA	Rhodopsin	A	1	Y	complete	
ggHTR1D	NA	Rhodopsin	A	4	Y	complete	
ggHTR1E	NA	Rhodopsin	A	1	Y	complete	
ggHTR1F	NA	Rhodopsin	A	3	Y	complete	
ggHTR2A	NA	Rhodopsin	A	3	Y	complete	
ggHTR2B	AAF20211.1	Rhodopsin	A	Known	N	partial	
ggHTR2C	NA	Rhodopsin	A	1	Y	complete	
ggHTR4	NA	Rhodopsin	A	1	Y	partial	missing first and last exon
ggHTR5	NA	Rhodopsin	A	1	Y	complete	
ggHTR6	NA	Rhodopsin	A	1	Y	complete	
ggHTR7	NA	Rhodopsin	A	1	N	partial	missing first Met due to poor genomic sequence
ggHTR7n1	NA	Rhodopsin	A	1	Y	partial	missing first Met due to poor genomic sequence
ggIodopsin	NP_990771.1	Rhodopsin	A	Known	Y	complete	
ggMC1R	AAO69658.1	Rhodopsin	A	Known	Y	complete	
ggMC2R	BAA24002.1	Rhodopsin	A	Known	Y	complete	
ggMC3R	BAA32555.1	Rhodopsin	A	Known	Y	complete	
ggMC4R	BAA25252.1	Rhodopsin	A	Known	Y	complete	
ggMC5R	BAA25640.1	Rhodopsin	A	Known	Y	complete	
ggMTN1A	NP_990693.1	Rhodopsin	A	Known	Y	complete	more similar to human MTN1A than ggMTN1A is.
ggMTN1C	NP_990692.1	Rhodopsin	A	Known	Y	complete	Alt. MTN1C
ggMTN1B	P51050	Rhodopsin	A	Known	N	partial	
ggOPN3	NA	Rhodopsin	A	1	Y	complete	
ggOPN4	NA	Rhodopsin	A	1	Y	partial	missing exon 1
ggOPN4n1	NP_989956.1	Rhodopsin	A	Known	Y	complete	Alt.putative photopigment melanopsin
ggOPSINn1	NA	Rhodopsin	A	1	N	partial	saknar exon1, exon3 och slutet.
ggPNR	NA	Rhodopsin	A	1	Y	complete	
ggPTGER2	NA	Rhodopsin	A	1	N	partial	short sequence due to poor genomic sequence
ggPTGER3	NA	Rhodopsin	A	1	N	partial	
ggPTGER4	NA	Rhodopsin	A	1	Y	complete	lack human specific insertion in exon 1
ggPTGFR	NA	Rhodopsin	A	1	Y	complete	
ggRE2	NA	Rhodopsin	A	2	Y	complete	
ggRGR	NA	Rhodopsin	A	1	Y	complete	
ggRHO	BAA00610.1	Rhodopsin	A	Known	Y	complete	
ggRRH	NA	Rhodopsin	A	1	Y	complete	
ggSREB2	NA	Rhodopsin	A	2	Y	complete	
ggSREB3	NA	Rhodopsin	A	2	N	partial	Missing large part of the gene due to Ns
ggTAR1	NA	Rhodopsin	A	1	Y	complete	
ggTAR2	NA	Rhodopsin	A	4	Y	complete	
ggVioletSensitiveConeOpsin	NP_990769.1	Rhodopsin	A	Known	Y	complete	
ggGPR119	NA	Rhodopsin	A	1	Y	complete	
ggAVPR1A	NA	Rhodopsin	B	3	N	partial	Missing first part due to poor genomic sequence
ggAVPR1B	AAG17937.2	Rhodopsin	B	Known	Y	complete	
ggBRS3	NP_989737.1|	Rhodopsin	B	Known*	Y	complete	
ggCCKAR	NA	Rhodopsin	B	1	Y	complete	
ggCCKBR	CAD33800.1	Rhodopsin	B	Known	Y	complete	
ggEDNRA	NP_989450.1	Rhodopsin	B	Known	Y	complete	
ggEDNRB	NP_989451.1	Rhodopsin	B	Known	Y	complete	
ggEDNRBn1	AAM74023.1	Rhodopsin	B	Known	Y	complete	
ggETBRLP1	NA	Rhodopsin	B	2	Y	partial	Missing first part due to poor genomic sequence

ggETBRLP2	NA	Rhodopsin	B	1	Y	complete	
ggGHSR_ghrelin	NP_989725.1	Rhodopsin	B	Known*	Y	complete	
ggGNRHRII	NP_00101262	Rhodopsin	B	Known	Y	complete	
ggGNRHR	NP_989984.1	Rhodopsin	B	Known	Y	complete	
ggGPR	NA	Rhodopsin	B	1	Y	complete	
ggGPR103	NA	Rhodopsin	B	1	Y	partial	missing exon 1
ggGPR154	NA	Rhodopsin	B	1	Y	complete	
ggGPR19	NA	Rhodopsin	B	2	Y	complete	
ggGPR39	NA	Rhodopsin	B	1	Y	complete	
ggGPR72	NA	Rhodopsin	B	2	Y	complete	
ggGPR72n1	NA	Rhodopsin	B	1	Y	complete	
ggGPR73/73L1	NA	Rhodopsin	B	1	Y	complete	
ggGPR75	NA	Rhodopsin	B	1	Y	partial	missing part of extracellular region due to poor genomic sequence
ggGRPR	NP_989738.1	Rhodopsin	B	Known	Y	complete	
ggHCRTR2	NP_00101975	Rhodopsin	B	Known	Y	complete	
ggMLNR	NA	Rhodopsin	B	2	Y	complete	one big deletion compared to human sequence homolog
ggNMBR	NA	Rhodopsin	B	1	Y	complete	
ggNMUR1	NA	Rhodopsin	B	1	Y	complete	
ggNMUR2	NA	Rhodopsin	B	2	Y	complete	
ggNPFF1	NP_989693.1	Rhodopsin	B	Known	Y	partial	missing first part of extracellular region
ggNPFF2	BAE17048.1	Rhodopsin	B	Known	Y	partial	missing exon 1
ggNPY1R	AAK83557.1	Rhodopsin	B	Known	Y	complete	
ggNPY2R	Q9DDN6	Rhodopsin	B	Known	Y	complete	
ggNPY5R	AAK83556.1	Rhodopsin	B	Known	Y	complete	
ggNPY6R	*	Rhodopsin	B	Known	Y	complete	probable orthologe to human pseudogene y6
ggNPY7R	*	Rhodopsin	B	Known	Y	complete	probable orthologe to fish Y7
ggNTSR1	NA	Rhodopsin	B	1	N	partial	missing exon 1
ggOXTR	NP_00102674	Rhodopsin	B	Known	N	partial	Missing met, pseudogene or bad genomic sequence, missing exon 2.
ggPPY1R	AAL84161.1	Rhodopsin	B	Known	Y	complete	
ggPRLHR1	BK005177	Rhodopsin	B	Known	Y	complete	
ggPRLHR1B	BK005491	Rhodopsin	B	Known	Y	complete	
ggPRLHR2	AY572194	Rhodopsin	B	Known	Y	complete	
ggTACR1	NP_990199.1	Rhodopsin	B	Known	Y	complete	
ggTACR2	NA	Rhodopsin	B	1	N	partial	missing parts of transmembrane region due to poor genomic sequence
ggTACR3	NA	Rhodopsin	B	1	Y	complete	
ggVT1	AAF18344.1	Rhodopsin	B	Known	Y	complete	
ggAGTR1	NP_990488.1	Rhodopsin	C	Known	Y	complete	
ggAGTR2	NA	Rhodopsin	C	2	Y	complete	
ggAGTRL1	NP_990488.1	Rhodopsin	C	Known	N	partial	missing transmembrane region 1 due to poor sequence
ggBDKRB1	*	Rhodopsin	C	3	Y	complete	
ggBDKRB2	CAA05099.1	Rhodopsin	C	Known	Y	complete	Alt. Ornithokinin
ggC3AR	NP_00102594	Rhodopsin	C	Known	Y	complete	
ggC5L2	NA	Rhodopsin	C	1	Y	complete	
ggCCBP2	NA	Rhodopsin	C	1	Y	partial	missing first Met
ggCCR11	NA	Rhodopsin	C	1	Y	complete	
ggCCR2/5n1a	CAF28777.1	Rhodopsin	C	Known	Y	complete	
ggCCR2/5n1b	NA	Rhodopsin	C	3	Y	complete	
ggCCR2/5n1c	CAF28776.1	Rhodopsin	C	Known*	N	partial	mising first part of transmembrane region
ggCCR4	NA	Rhodopsin	C	1	Y	complete	
ggCCR6	NA	Rhodopsin	C	1	Y	partial	missing first Met
ggCCR7	NA	Rhodopsin	C	1	Y	partial	missing exon 1
ggCCR8a	CAF28778.1	Rhodopsin	C	Known	Y	complete	
ggCCR8b	NP_00102616	Rhodopsin	C	Known	Y	complete	
ggCCR9	CAF28781.1	Rhodopsin	C	Known	Y	complete	
ggCCXCR1	CAF28779.1	Rhodopsin	C	Known	Y	complete	
ggCMKLR1	NA	Rhodopsin	C	1	Y	complete	

ggCMKLR2	NP_00102608	Rhodopsin	C	Known	Y	complete	
ggCXC3R1	NA	Rhodopsin	C	1	Y	complete	
ggCXCR4	NP_989948.1	Rhodopsin	C	Known	Y	complete	
ggCXCR5	AAC23950.1	Rhodopsin	C	Known	Y	complete	
ggGALR1	NA	Rhodopsin	C	1	Y	complete	
ggGALR2	NA	Rhodopsin	C	1	Y	complete	
ggGALR2n1	NA	Rhodopsin	C	1	Y	partial	missing first Met and last part of cytosolic region
ggGPR1	NA	Rhodopsin	C	1	Y	complete	
ggGPR15	NA	Rhodopsin	C	2	Y	complete	
ggCMKLR1n1	NA	Rhodopsin	C	1	Y	complete	
ggGPR7	NA	Rhodopsin	C	1	Y	complete	
ggMCHR1	NA	Rhodopsin	C	1	N	partial	missing first and last part due to poor genomic sequence
ggOPRD1	NA	Rhodopsin	C	1	N	partial	missing last exon
ggOPRK1	NA	Rhodopsin	C	1	Y	complete	
ggOPRL1	NA	Rhodopsin	C	1	Y	complete	
ggOPRM1	NA	Rhodopsin	C	1	Y	complete	
ggRDC1	NA	Rhodopsin	C	2	N	partial	Missing first part due to poor genomic sequence
ggRLN3R1	NA	Rhodopsin	C	1	Y	complete	
ggSSTR1	NA	Rhodopsin	C	1	Y	complete	
ggSSTR2	AAK33003.1	Rhodopsin	C	Known	N	partial	
ggSSTR3	NP_00101975	Rhodopsin	C	Known	Y	complete	
ggSSTR4	AAY82239.1	Rhodopsin	C	Known	Y	complete	
ggSSTR5	NP_00102000	Rhodopsin	C	Known	Y	complete	
ggUTS2R	NA	Rhodopsin	C	1	Y	complete	
ggUTIIn2	NA	Rhodopsin	C	3	N	partial	
ggUTIIn1	NA	Rhodopsin	C	3	Y	complete	
ggCYSLTR1	NA	Rhodopsin	D	1	Y	complete	
ggCYSLTR2	NA	Rhodopsin	D	2	Y	complete	
ggEBI2	NA	Rhodopsin	D	1	Y	complete	
ggEBVR	NA	Rhodopsin	D	3	N	partial	missing part of transmembrane region and cytosolic part due to poor genomic sequence
ggF2R	NA	Rhodopsin	D	1	Y	complete	Not well conserved in the start
ggF2RL1	NP_00101262	Rhodopsin	D	Known	Y	complete	Not well conserved in the start
ggF2RL3	NA	Rhodopsin	D	1	Y	complete	
ggF2RL3n1	NA	Rhodopsin	D	4	Y	complete	
ggFKSG79	NP_00100846	Rhodopsin	D	Known	Y	complete	
ggFSHR	NP_990410.1	Rhodopsin	D	Known	Y	complete	
ggG2A	NA	Rhodopsin	D	3	N	partial	missing part of transmembrane region and cytosolic part due to poor genomic sequence
ggGPR17	NA	Rhodopsin	D	1	Y	partial	missing exon 1
ggGPR18	NA	Rhodopsin	D	2	N	partial	missing part of transmembrane region and cytosolic part due to poor genomic sequence
ggGPR20	NA	Rhodopsin	D	1	Y	complete	
ggGPR34	AAP04299.1	Rhodopsin	D	Known	Y	complete	
ggGPR34n1	NA	Rhodopsin	D	1	Y	complete	
ggGPR43n1a	NA	Rhodopsin	D	1	N	partial	missing extracellular, transmembrane and cytosolic parts due to poor genomic sequence
ggGPR43n1b	NA	Rhodopsin	D	1	Y	complete	
ggGPR43n1c	NA	Rhodopsin	D	1	Y	complete	
ggGPR43n1d	NA	Rhodopsin	D	1	Y	complete	
ggGPR43n1e	NA	Rhodopsin	D	1	Y	complete	
ggGPR43n1f	NA	Rhodopsin	D	1	Y	complete	
ggGPR43n1g	NA	Rhodopsin	D	1	Y	partial	Missing C-term due to Ns
ggGPR43n1h	NA	Rhodopsin	D	1	Y	complete	
ggGPR55	NA	Rhodopsin	D	1	Y	complete	
ggGPR65	NA	Rhodopsin	D	1	Y	complete	
ggGPR82	NA	Rhodopsin	D	3	Y	complete	
ggGPR87	NA	Rhodopsin	D	1	Y	complete	
ggSUCNR1	NA	Rhodopsin	D	1	Y	complete	
ggGPR92	NA	Rhodopsin	D	3	Y	complete	

ggCYSLTRn1	NA	Rhodopsin	D	1	Y	complete	
ggMRGn3	NA	Rhodopsin	D	1	Y	complete	
ggMRGn1	NA	Rhodopsin	D	1	Y	complete	
ggMRGn2	NA	Rhodopsin	D	1	N	partial	missing part of xetracellular region and transmembrane part due to poor genomic sequence
ggMRGn4	NA	Rhodopsin	D	1	Y	complete	
ggMRGn5	NA	Rhodopsin	D	1	Y	complete	
ggH963	NA	Rhodopsin	D	3	Y	complete	
ggLGR4	NA	Rhodopsin	D	4	Y	partial	missing all exons except the last
ggLGR5	NA	Rhodopsin	D	2	Y	partial	missing parts of extracellular region
ggLGR7	NA	Rhodopsin	D	2	N	partial	missing exon 1, 2, 3 and 8
ggLGRn1	NA	Rhodopsin	D	1	Y	partial	missing parts of extracellular region
ggLHCGR	NP_990267.1	Rhodopsin	D	Known	Y	complete	
ggMAS	NA	Rhodopsin	D	1	Y	complete	
ggOGR1	NA	Rhodopsin	D	1	Y	complete	
ggP2Y1	NP_990664.1	Rhodopsin	D	Known	Y	complete	
ggP2Y10a	NA	Rhodopsin	D	1	Y	complete	
ggP2Y10b	NA	Rhodopsin	D	1	N	partial	missing part of transmembrane region
ggP2Y12	NA	Rhodopsin	D	1	Y	complete	
ggP2Y13	NA	Rhodopsin	D	1	Y	complete	
ggP2Y14	NA	Rhodopsin	D	3	Y	complete	
ggP2Y15b	NA	Rhodopsin	D	2	Y	complete	
ggP2Y15a	NA	Rhodopsin	D	4	Y	complete	
ggP2Y1n1	NA	Rhodopsin	D	1	N	partial	missing extracellular region and first part of transmembrane region due to poor genomic sequence
ggP2Y2	NA	Rhodopsin	D	4	Y	complete	
ggP2Y4	NA	Rhodopsin	D	1	Y	complete	
ggP2Y5	P32250	Rhodopsin	D	Known*	Y	complete	
ggP2Y6	NP_990526.1	Rhodopsin	D	Known	Y	complete	
ggP2Y8	NP_00100867	Rhodopsin	D	Known	Y	complete	
ggP2Y9	NA	Rhodopsin	D	1	Y	complete	
ggPTFAR	NA	Rhodopsin	D	2	Y	complete	
ggTSHR	BAE44410.1	Rhodopsin	D	Known	Y	partial	missing part of extracellular region
ggCOR7a	CAB01847.1	Rhodopsin	olfactory	Known			
ggOR1	NA	Rhodopsin	Olfactory	1			Missing TM5-stop due to Ns
ggOR2	NA	Rhodopsin	olfactory	1			Missing C-term due to Ns
ggOR3	NA	Rhodopsin	olfactory	1			Missing C-term due to Ns
ggOR4	NA	Rhodopsin	olfactory	1			Missing C-term due to Ns
ggOR5	NA	Rhodopsin	olfactory	1			Missing C-term due to Ns
ggOR6	NA	Rhodopsin	olfactory	1			Missing C-term due to Ns
ggCOR7b	CAB01848.1	Rhodopsin	olfactory	Known			Missing C-term due to Ns
ggOR8	NA	Rhodopsin	olfactory	1			Missing first half due to Ns
ggOR9	NA	Rhodopsin	olfactory	1			Missing last part due to Ns
ggOR10	NA	Rhodopsin	olfactory	1			Missing last part due to Ns
ggOR11	NA	Rhodopsin	olfactory	1			Missing Last part due to Ns
ggOR12	NA	Rhodopsin	olfactory	1			Missing Last part due to Ns
ggOR13	NA	Rhodopsin	olfactory	1			Missing Met due to Ns
ggOR14	NA	Rhodopsin	olfactory	1			Missing Met -TM3 due to N
ggOR15	NA	Rhodopsin	olfactory	1			Missing Met -TM3 due to N
ggOR16	NA	Rhodopsin	olfactory	1			Missing Met-TM3
ggOR17	NA	Rhodopsin	olfactory	1			Missing Met-TM3 due to N
ggOR18	NA	Rhodopsin	olfactory	1			Missing Met-TM3 due to Ns
ggOR19	NA	Rhodopsin	olfactory	1			Missing Met-TM3 due to Ns
ggOR20	NA	Rhodopsin	olfactory	1			Missing Met-TM3 due to Ns
ggOR21	NA	Rhodopsin	olfactory	1			Missing Met-TM5 due to Ns
ggOR22	NA	Rhodopsin	olfactory	1			Missing N och C-term due to Ns
ggOR23	NA	Rhodopsin	olfactory	1			Missing N-term due to Ns
ggOR24	NA	Rhodopsin	olfactory	1			Missing N-term due to Ns

ggOR25	NA	Rhodopsin	olfactory	1	Missing N-term-TM2 due to Ns
ggOR26	NA	Rhodopsin	olfactory	1	Missing N-term-TM3 due to Ns
ggOR27	NA	Rhodopsin	olfactory	1	Missing Start-TM1 due to Ns
ggOR28	NA	Rhodopsin	olfactory	1	Missing Start-TM2
ggOR29	NA	Rhodopsin	olfactory	1	Missing Start-TM2 due to Ns
ggOR30	NA	Rhodopsin	olfactory	1	Missing Start-TM2 due to Ns
ggOR31	NA	Rhodopsin	olfactory	1	Missing Start-TM2 due to Ns
ggOR32	NA	Rhodopsin	olfactory	1	Missing Start-TM2 due to Ns
ggOR33	NA	Rhodopsin	olfactory	1	Missing Start-TM2 due to Ns
ggOR34	NA	Rhodopsin	olfactory	1	Missing Start-TM2 due to Ns
ggOR35	NA	Rhodopsin	olfactory	1	Missing Start-TM2 due to Ns
ggOR36	NA	Rhodopsin	olfactory	1	Missing start-TM2 due to Ns
ggOR37	NA	Rhodopsin	olfactory	1	Missing Start-TM2 due to Ns
ggOR38	NA	Rhodopsin	olfactory	1	Missing Start-TM2 due to Ns
ggOR39	NA	Rhodopsin	olfactory	1	Missing start-TM3 due to Ns
ggOR40	NA	Rhodopsin	olfactory	1	Missing Start-TM3 due to Ns
ggOR41	NA	Rhodopsin	olfactory	1	Missing Start-TM3 due to Ns
ggOR42	NA	Rhodopsin	olfactory	1	Missing Start-TM3 due to Ns
ggOR43	NA	Rhodopsin	olfactory	1	Missing start-TM4 due to Ns
ggOR44	NA	Rhodopsin	olfactory	1	Missing the middle part due to N
ggOR45	NA	Rhodopsin	olfactory	1	Missing the middle part due to N
ggOR46	NA	Rhodopsin	olfactory	1	Missing TM2-start due to Ns
ggOR47	NA	Rhodopsin	olfactory	1	Missing TM2-stop due to Ns
ggOR48	NA	Rhodopsin	olfactory	1	Missing TM2-stop due to Ns
ggOR49	NA	Rhodopsin	olfactory	1	Missing TM3-Stop due to Ns
ggOR50	NA	Rhodopsin	olfactory	1	Missing TM3-stop due to Ns
ggOR51	NA	Rhodopsin	olfactory	1	Missing TM3-stop due to Ns
ggOR52	NA	Rhodopsin	olfactory	1	Missing TM3-stop due to Ns
ggOR53	NA	Rhodopsin	olfactory	1	Missing TM3-stop due to Ns
ggOR54	NA	Rhodopsin	olfactory	1	Missing TM3-stop due to Ns
ggOR55	NA	Rhodopsin	olfactory	1	Missing TM3-stop due to Ns
ggOR56	NA	Rhodopsin	olfactory	1	Missing TM3-stop due to Ns
ggOR57	NA	Rhodopsin	olfactory	1	Missing TM3-stop due to Ns
ggOR58	NA	Rhodopsin	olfactory	1	Missing TM3-stop due to Ns
ggOR59	NA	Rhodopsin	olfactory	1	Missing TM3-stop due to Ns
ggOR60	NA	Rhodopsin	olfactory	1	Missing TM3-stop due to Ns
ggOR61	NA	Rhodopsin	olfactory	1	Missing TM3-stop due to Ns
ggOR62	NA	Rhodopsin	olfactory	1	Missing TM3-TM7 due to Ns
ggOR63	NA	Rhodopsin	olfactory	1	Missing TM4-stop due to Ns
ggOR64	NA	Rhodopsin	olfactory	1	Missing TM4-stop due to Ns
ggOR65	NA	Rhodopsin	olfactory	1	Missing TM4-stop due to Ns
ggOR66	NA	Rhodopsin	olfactory	1	Missing TM4-TM7 due to Ns
ggOR67	NA	Rhodopsin	olfactory	1	Missing TM4-TM7 due to Ns
ggOR68	NA	Rhodopsin	olfactory	1	Missing TM4-TM7 due to Ns
ggOR69	NA	Rhodopsin	olfactory	1	Missing TM4-TM7 due to Ns
ggOR70	NA	Rhodopsin	olfactory	1	Missing TM4-TM7 due to Ns
ggOR71	NA	Rhodopsin	olfactory	1	Missing TM5-stop due to Ns
ggOR72	NA	Rhodopsin	olfactory	1	Missing TM5-stop due to Ns
ggOR73	NA	Rhodopsin	olfactory	1	Missing TM5-stop due to Ns
ggOR74	NA	Rhodopsin	olfactory	1	Missing TM5-stop due to Ns
ggOR75	NA	Rhodopsin	olfactory	1	Missing TM5-stop due to Ns
ggOR76	NA	Rhodopsin	olfactory	1	Missing TM5-stop due to Ns
ggOR77	NA	Rhodopsin	olfactory	1	Missing TM5-stop due to Ns
ggOR78	NA	Rhodopsin	olfactory	1	Missing TM6-stop due to Ns
ggOR79	NA	Rhodopsin	olfactory	1	Missing TM6-stop due to Ns
ggOR80	NA	Rhodopsin	olfactory	1	Missing TM6-stop due to Ns
ggOR81	NA	Rhodopsin	olfactory	1	Missing TM6-stop due to Ns

ggOR82	NA	Rhodopsin	olfactory	1	Missing TM7-C-term due to Ns
ggOR83	NA	Rhodopsin	olfactory	1	Missing TM7-c-term due to Ns
ggOR84	NA	Rhodopsin	olfactory	1	Missing TM7-stop due to Ns
ggOR85	NA	Rhodopsin	olfactory	1	Missing TM7-stop due to Ns
ggOR86	NA	Rhodopsin	olfactory	1	Missing TM7-stop due to Ns
ggOR87	NA	Rhodopsin	olfactory	1	Missing TM7-stop due to Ns
ggOR88	NA	Rhodopsin	olfactory	1	
ggOR89	NA	Rhodopsin	olfactory	1	
ggOR90	NA	Rhodopsin	olfactory	1	
ggOR91	NA	Rhodopsin	olfactory	1	
ggOR92	NA	Rhodopsin	olfactory	1	
ggOR93	NA	Rhodopsin	olfactory	1	
ggOR94	NA	Rhodopsin	olfactory	1	
ggOR95	NA	Rhodopsin	olfactory	1	
ggOR96	NA	Rhodopsin	olfactory	1	
ggOR97	NA	Rhodopsin	olfactory	1	
ggOR98	NA	Rhodopsin	olfactory	1	
ggOR99	NA	Rhodopsin	olfactory	1	
ggOR100	NA	Rhodopsin	olfactory	1	
ggOR101	NA	Rhodopsin	olfactory	1	
ggOR102	NA	Rhodopsin	olfactory	1	
ggOR103	NA	Rhodopsin	olfactory	1	
ggOR104	NA	Rhodopsin	olfactory	1	
ggOR105	NA	Rhodopsin	olfactory	1	
ggOR106	NA	Rhodopsin	olfactory	1	
ggOR107	NA	Rhodopsin	olfactory	1	
ggOR108	NA	Rhodopsin	olfactory	1	
ggOR109	NA	Rhodopsin	olfactory	1	
ggOR110	NA	Rhodopsin	olfactory	1	
ggOR111	NA	Rhodopsin	olfactory	1	
ggOR112	NA	Rhodopsin	olfactory	1	
ggOR113	NA	Rhodopsin	olfactory	1	
ggOR114	NA	Rhodopsin	olfactory	1	
ggOR115	NA	Rhodopsin	olfactory	1	
ggOR116	NA	Rhodopsin	olfactory	1	
ggOR117	NA	Rhodopsin	olfactory	1	
ggOR118	NA	Rhodopsin	olfactory	1	
ggOR119	NA	Rhodopsin	olfactory	1	
ggOR120	NA	Rhodopsin	olfactory	1	
ggOR121	NA	Rhodopsin	olfactory	1	
ggOR122	NA	Rhodopsin	olfactory	1	
ggOR123	NA	Rhodopsin	olfactory	1	
ggOR124	NA	Rhodopsin	olfactory	1	
ggOR125	NA	Rhodopsin	olfactory	1	
ggOR126	NA	Rhodopsin	olfactory	1	
ggOR127	NA	Rhodopsin	olfactory	1	
ggOR128	NA	Rhodopsin	olfactory	1	
ggOR129	NA	Rhodopsin	olfactory	1	
ggOR130	NA	Rhodopsin	olfactory	1	
ggOR131	NA	Rhodopsin	olfactory	1	
ggOR132	NA	Rhodopsin	olfactory	1	
ggOR133	NA	Rhodopsin	olfactory	1	
ggOR134	NA	Rhodopsin	olfactory	1	
ggOR135	NA	Rhodopsin	olfactory	1	
ggOR136	NA	Rhodopsin	olfactory	1	
ggOR137	NA	Rhodopsin	olfactory	1	
ggOR138	NA	Rhodopsin	olfactory	1	

ggOR139	NA	Rhodopsin	olfactory	1
ggOR140	NA	Rhodopsin	olfactory	1
ggOR141	NA	Rhodopsin	olfactory	1
ggOR142	NA	Rhodopsin	olfactory	1
ggOR143	NA	Rhodopsin	olfactory	1
ggOR144	NA	Rhodopsin	olfactory	1
ggOR145	NA	Rhodopsin	olfactory	1
ggOR146	NA	Rhodopsin	olfactory	1
ggOR147	NA	Rhodopsin	olfactory	1
ggOR148	NA	Rhodopsin	olfactory	1
ggOR149	NA	Rhodopsin	olfactory	1
ggOR150	NA	Rhodopsin	olfactory	1
ggOR151	NA	Rhodopsin	olfactory	1
ggOR152	NA	Rhodopsin	olfactory	1
ggOR153	NA	Rhodopsin	olfactory	1
ggOR154	NA	Rhodopsin	olfactory	1
ggOR155	NA	Rhodopsin	olfactory	1
ggOR156	NA	Rhodopsin	olfactory	1
ggOR157	NA	Rhodopsin	olfactory	1
ggOR158	NA	Rhodopsin	olfactory	1
ggOR159	NA	Rhodopsin	olfactory	1
ggOR160	NA	Rhodopsin	olfactory	1
ggOR161	NA	Rhodopsin	olfactory	1
ggOR162	NA	Rhodopsin	olfactory	1
ggOR163	NA	Rhodopsin	olfactory	1
ggOR164	NA	Rhodopsin	olfactory	1
ggOR165	NA	Rhodopsin	olfactory	1
ggOR166	NA	Rhodopsin	olfactory	1
ggOR167	NA	Rhodopsin	olfactory	1
ggOR168	NA	Rhodopsin	olfactory	1
ggOR169	NA	Rhodopsin	olfactory	1
ggOR170	NA	Rhodopsin	olfactory	1
ggOR171	NA	Rhodopsin	olfactory	1
ggOR172	NA	Rhodopsin	olfactory	1
ggOR173	NA	Rhodopsin	olfactory	1
ggOR174	NA	Rhodopsin	olfactory	1
ggOR175	NA	Rhodopsin	olfactory	1
ggOR176	NA	Rhodopsin	olfactory	1
ggOR177	NA	Rhodopsin	olfactory	1
ggOR178	NA	Rhodopsin	olfactory	1
ggOR179	NA	Rhodopsin	olfactory	1
ggOR180	NA	Rhodopsin	olfactory	1
ggOR181	NA	Rhodopsin	olfactory	1
ggOR182	NA	Rhodopsin	olfactory	1
ggOR183	NA	Rhodopsin	olfactory	1
ggOR184	NA	Rhodopsin	olfactory	1
ggOR185	NA	Rhodopsin	olfactory	1
ggOR186	NA	Rhodopsin	olfactory	1
ggOR187	NA	Rhodopsin	olfactory	1
ggOR188	NA	Rhodopsin	olfactory	1
ggOR189	NA	Rhodopsin	olfactory	1
ggOR190	NA	Rhodopsin	olfactory	1
ggOR191	NA	Rhodopsin	olfactory	1
ggOR192	NA	Rhodopsin	olfactory	1
ggOR193	NA	Rhodopsin	olfactory	1
ggOR194	NA	Rhodopsin	olfactory	1
ggOR195	NA	Rhodopsin	olfactory	1

ggOR196	NA	Rhodopsin	olfactory	1			
ggOR197	NA	Rhodopsin	olfactory	1			
ggOR198	NA	Rhodopsin	olfactory	1			
ggOR199	NA	Rhodopsin	olfactory	1			
ggOR200							
ggOR201	NA	Rhodopsin	olfactory	1			
ggOR202	NA	Rhodopsin	olfactory	1			
ggOR203	NA	Rhodopsin	olfactory	1			
ggOR204	NA	Rhodopsin	olfactory	1			
ggOR205	NA	Rhodopsin	olfactory	1			
ggOR206	NA	Rhodopsin	olfactory	1			
ggOR207	NA	Rhodopsin	olfactory	1			
ggOR208	NA	Rhodopsin	olfactory	1			
ggOR209	NA	Rhodopsin	olfactory	1			
ggOR210	NA	Rhodopsin	olfactory	1			
ggOR211	NA	Rhodopsin	olfactory	1			
ggOR212	NA	Rhodopsin	olfactory	1			
ggOR213	NA	Rhodopsin	olfactory	1			
ggOR214	NA	Rhodopsin	olfactory	1			
ggOR215	NA	Rhodopsin	olfactory	1			
ggOR216							
ggOR217	NA	Rhodopsin	olfactory	1			
ggOR218	NA	Rhodopsin	olfactory	1			
ggOR219	NA	Rhodopsin	olfactory	1			
ggOR220	NA	Rhodopsin	olfactory	1			Missing TM3-stop due to Ns
ggOR221	NA	Rhodopsin	olfactory	1			Missing C-tern due to Ns
ggOR223	NA	Rhodopsin	olfactory	1			
COR1	P37067	Rhodopsin	olfactory	Known			
COR2	P37068	Rhodopsin	olfactory	Known			
COR3	P37069	Rhodopsin	olfactory	Known			
COR4	P37070	Rhodopsin	olfactory	Known			
COR5	P37071	Rhodopsin	olfactory	Known			
COR6	P37072	Rhodopsin	olfactory	Known			
ggCALCRn1	NA	Secretin		1	Y	partial	missing exon 1
ggCALCRL	NA	Secretin		1	Y	partial	missing exon 1
ggCRHR1	NP_989652.1	Secretin		Known	Y	complete	
ggCRHR2	NP_989785.1	Secretin		Known	Y	partial	missing first exon and the two last exons
ggGCGR	NA	Secretin		1	N	partial	missing large part of the gene
ggGIPR	NA	Secretin		1	N	partial	only exon 7
ggGLP1R	NA	Secretin		1	Y	partial	missing exon 1
ggPACAPR	NA	Secretin		1	Y	partial	missing exon 1
ggPTHR1	NA	Secretin		1	Y	partial	missing parts of extracellular region
ggPTHR2	NA	Secretin		3	N	partial	missing exon 1 and one internal exon
ggSCTR	NA	Secretin		1	Y	partial	missing exon 1
ggVIPR1	BAA95164.1	Secretin		Known	Y	partial	missing exon 1
ggGHRHR	NA	Secretin		1	Y	partial	missing large part of extracellular region due to poor genomic sequence
ggVIPR2	NP_00101497	Secretin		Known	Y	partial	missing exon 1
ggTAS2Rn1	NA	Taste 2		1	Y	complete	
ggTAS2Rn2	NA	Taste 2		4	Y	complete	
ggTAS2Rn3	NA	Taste 2		4	Y	complete	
