# Supplementary material for: The G Protein–Coupled Receptor Subset of the Chicken Genome
Source: PLoS Comput Biol. 2006 Jun 2;2(6):e54. doi: 10.1371/journal.pcbi.0020054 (PMC1472694; doi:10.1371/journal.pcbi.0020054)
Supplement: Supplementary file 2 [file pcbi.0020054.sd003.pdf]

```
>ggFZD1
MAERRGPAGGGSGEVGGGRRAGGDRCPRRPPALPLLLLLWAAALPAGGQPAAQPAALSERGISIPDHGYCQPISIPLCTDI
AYNQTIMPNLLGHTNQEDAGLEVHQFYPLVKVQCSAELKFFLCSMYAPVCTVLEQALPPCRSLCERARQGCEALMNKFGFQ
WPDTLRCEKFPVHGAGELCVGQNASERGTPTPALRPESWTSNPHRGGGAGGSGPGEARGRFSCPRALKVPSYLNYRFLGEK
DCGAPCEPGRLYGLMYFGPEELRFSRTWIGIWSVLCCASTLFTVLTYLVDMKRFSYPERPIIFLSGCYTAVAVAYIAGFLL
EERVVCNERFAEDGSRTVAQGTKREGCTILFMMLYFFGMASSIWWVILSLTWFLAAGMKWGHEAIEANSQYFHLAAWAVPA
IKTITILALGQVDGDVLSGVCFVGINNVDALRGFVLAPLFVYLFIGTSFLLAGFVSLFRIRTIMKHDGTKTEKLEKLMVRI
GIFSVLYTVPATIVIACYFYEQAFREQWERSWVTQSCKSYAIPCPNNHSSHHPPMSPDFTVFMIKYLMTLIVGITSGFWIW
SGKTLNSWRKFYTRLTNSKQGETTV
>ggFZD2
MRAPPGALPALAVLLAGLSAAPSRGQPYGEKGISVPDHGFCQPISIPLCTDIAYNQTIMPNLLGHTNQEDAGLEVHQFYPL
VKVQCSLELKFFLCSMYAPVCTVLEQAIPPCRSICERARQGCEALMNKFGFQWPERLRCENFPRHGAEQICVGQNHSEDGG
SSAALLTSAAPPAAHGTPGAPRYATPDRPFHCPRALKVPGYLNYKFLGEKDCAAPCEPSRPDGHMFFNEDEIRFARVWILV
WSVLCCASTFFTVTTYLVDMQRFRYPERPIIFLSGCYTMVSVAYIAGFVLEERVVCNERFQEDGYRTVVQGTKKEGCTILF
MMLYFFSMASSIWWVILSLTWFLAAGMKWGHEAIEANSQYFHLAAWAVPAVKTITILAMGQIDGDLLSGVCFVGLNGIDPL
RGFVLAPLFVYLFIGTSFLLAGFVSLFRIRTITKHGGTKTEKLERLMVRIGVFSVLYTVPATIVIACYFYEQAFRQHWERS
WISQHCKSLAIPCPLHFTPRMTPDFTVYMIKYLMTLIVGITSGFWIWSGKTLHSWRKFYTRLTNSRQGETTV
>ggFZD3
MAVSWMFCCFWLLDVLVGCTRGHSLFSCEPIILRMCQDLPYNTTFMPNLLNHYDQQTAALAMEPFHPMVNLECSRDFRPFL
CALYAPVCMEYGRVTLPCRRLCQRAYSECSKLMEMFGVSWPEDMECTRFPDCDEPYPRLVDLSLGGEPTEEAPMAVQRDYG
FWCPRELKIDPDLGYSFLRVRDCSPPCPNMYFRREELSFARYFIGVISIVCLSATLFTFLTFLIDVTRFRYPERPIIFYAV
CYMMVSLIFFIGFLLEDRVACNASSPAQYKASTVTQGSHNKACTMLFMVLYFFTMAGSVWWVILTITWFLAAVPKWGSEAI
EKKALLFHASAWGIPGTLTIVLLAMNKIEGDNISGVCFVGLYDVDALRYFVLAPLCLYVVVGVSLLLAGIISLNRVRIEIP
LEKENQDKLVKFMIRIGVFSVLYLVPLLVVIGCYFYEQAYRGVWETTWIQERCREYHIPCPYQVTQMSRPDLILFLMKYLM
ALVVGIPSVFWVGSKKTCFEWASFFHGRRKKEVVNESRQVLQEPDFAQSLLRDPNTPIIRKSRGTSTQGTSTHASSTQLAM
LDDQRSKAGSVHSKVSSYHGSLHRSRDGRYTPCSYRGIEERLPHGSMSRLTDHSRHSSSHRLNEQSRHSSIRDLSSNPLTH
ITHGTSMNRVIEEDGTSA
>ggFZD4
MERRGGGGRMLALLLAGLLGGARGFGDEEERRCDAIRIAMCQNLGYNVTKMPNLVGHELQADAELQLTTFTPLIQYGCSSQ
LQFFLCSVYVPMCTEKINIPIGPCGGMCLSVKRRCEPVLKEFGFAWPDSLNCSKFPPQNDHNHMCMEGPGDEEVPLHSKTS
LQPGEECHSMGSNSDQYIWVKRNLDCVLKCGYDAGLYSRSAKEFTDIWMAVWASLCFISTAFTVLTFLIDSSRFSYPERPI
IFLSMCYNIYSIAYIVRLTVGRERISCDFEEAAEPVLIQEGLKNTGCAIIFLLMYFFGMASSIWWVILTLTWFLAAGLKWG
HEAIEMHSSYFHIAAWAIPAVKTIVILIMRLVDADELTGLCYVGNQNLDALTGFVVAPLFTYLVIGTLFIAAGLVALFKIR
SNLQKDGTKTDKLERLMVKIGVFSVLYTVPATCVIACYFYEISNWAVFRYSADDSNMAVEMLKIFMSLLVGITSGMWIWSA
KTLHTWQKCSNRLVNSGKVKREKRADGWVKPGKGNETVV
>ggFZDn1
yperpiiflsacylfvsvgyivrlvaghasvacdpehhhihyettgpalctvvflllyffgmassiwwvilsltwflaagm
kwgneaiasyaqyfhiaawXXXDTQDEAGLEVHQFWPLVEIQCSSDLRFFLCSMYTPICLEDYKKPLPPCRSVCERAKAGC
APLMRQYGFAWPDRMRCDRLPXXXTFLIDVKRFRYPERPIIYYSVCYSIVSLXXXCTILFMMLYFFGMASSIWWVILSLTW
FLAAGMKWGHEAIEANSQYFHLAAWAVPAIKTITILALGQVDGDVLSGVCFVGINNVDALRGFVLAPLFVYLFIGTSFLLA
GFVSLFRIRTIMKHDGTKTEKLEKLMVRIGIFSVLYTVPATIVIACYFYEQAFREQWEXXXPDYAVFMLKYFMCLVVGITS
GVWVWSGKT
>ggFZD6
HEVCYSIVSLMYFIGFLLGNRTACNKADDKLEIGETVVLGSQNKACTVLFMVLYFFTMAGTIWWVILTITWFLAAGRKWSC
EAIAQKAMWFHAVAWGIPGFLTIMLLAMNKVEGDNISGVCFVGLYDVDASLYFVLLPLCLCVFFGLSLLLAGIISLNHVRQ
VIQHDGRNQEKLKKFMIRIGVFSGLYLVPLVALLGCYVYELVNRKIWETTWVFDHCDQYHIPCPYQAKALARPEIFLFLMK
YLLTLIVGISPVFWVGSKKTCSEWANFFNRNRKRDPISESRRVLQESCEFFLRHNSKVKHKKKHYKSSSHRLKVISKSMGT
STGGTTNHGTSAVAITNHDYLSQETVAEIKTSPETSEKEMEADGTSAQKAEEGENSGNQMLSVSKLTVDQVEKRNKADSTC
DMSSLSESLKRIGEGRVSPRNDLIESPPLQSSSSQIADVSQSGSVSLLVYSAADGRKEMESGNSSNH
>ggFZD7
MRPAAGEAGAGLRWLGLAALLAALLGTPCAAAHQDDKAISVPDHGFCQPISIPLCTDIAYNQTILPNLLGHTNQEDAGLEV
HQFYPLVKVQCSAELKFFLCSMYAPVCTVLEQAIPPCRSLCERARQGCEALMNKFGFQWPERLRCENFPVHGAGEICVGQN
TSDAPPGPGGAGGRGATAQPTAGYLPDLLTPPQPAAGFSFSCPRQLKVPPYLGYRFLGERDCGAPCEPGRPNGLMYFKEAE
VRFARLWVGVWSVLCCASTLFTVLTYLVDMRRFSYPERPIIFLSGCYFMVAVAYAAGFLLEERVVCLERFSEDGYRTVAQG
TKKEGCTILFMILYFFGMASSIWWVILSLTWFLAAGMKWGHEAIEANSQYFHLAAWAVPAVKTITILAMGQVDGDVLSGVC
YVGIYSVDSLRGFVLAPLFVYLFIGTSFLLAGFVSLFRIRTIMKHDGTKTEKLEKLMVRIGVFSVLYTVPATIVLACYFYE
QAFRSTWEKTWLLQTCKTYAVPCPSHFAPMSPDFTVFMIKYLMTMIVGITTGFWIWSGKTLQSWRRFYHRLSTGSKGETAV
>ggFZD8
DTQDEAGLEVHQFWPLVEIQCSSDLRFFLCSMYTPICLEDYKKPLPPCRSVCERAKAGCAPLMRQYGFAWPDRMRCDRLPE
QGSPDTLCMDYNRTDLTTAAPPPAKPCEPGCQCRAPMVSVSNERHPLYNRVKTGXXXXXXXXXXXXXXXXXXXXXXXXXXX
XXXXXXXXXXXXXXXXXXXXFLLFYFFGMASSIWWVILSLTWFLAAGMKWGNEAIAGYAQYFHLAAWLLPSVKSIAVLALS
SVDGDPVAGICYVGNQSLENLRGFVLAPLLIYLAIGSMFLLAGFVSLFRIRSVIKQQGGPTKTHKLEKLMIRLGLFTVLYT
VPAASVVACLFYEQHNRPRWEATHNCPCLRDQQPDQARRPDYAVFMLKYFMCLVVGITSGVWVWSGKTLESWRALCTRCCW
ASKGAAVAGGAGAGAGGQTAIAAAGGLGAGGGGSLYSDVSTGLTWRSGTASSVSYPKQMPLSQV
>ggFZD10
MGPAAGNLVRAVLALCWLAEHCAGISSIDIERPGDGRCQPIEIPMCKDIGYNMTRMPNLMGHENQREAAIQLHEFAPLVEY
GCHGHLKFFLCSLYAPMCTEQVSTPIPACRVMCEQARLKCSPIMEQFNFKWPDSLDCSKLPNKNDPNYLCMEAPNNGSDEP
PRGSSMLPPMFRPQRPSTGHDLQQHKDSLSRTSCENPGKFHHVEKSASCAPLCTPGVDVYWSKDDKQFAVIWIAIWSILCF
```

FSSAFTVLTFLIDPQRFKYPERPIIFLSMCYCVYSVGYIIRLFSGAESIACDRDSGQLYVIQEGLESTGCTIVFLVLYYFG
MASSLWWVILTLTWFLAAGKKWGHEAIEANSSYFHLAAWAIPAVKTIMILVMRRVAGDELTGLCYVGSMDVNALTGFVLIP
LACYLIIGTSFILSGFVALFHIRRVMKTGGENTDKLEKLMVRIGVFSVLYTVPATCVIACYFYERLNMDYWKIVASQQKCK
MNNQTKNLDCMMNNSIPAVEIFMVKIFMLLVVGITSGMWIWTSKTLQSWQNVCSRRLKKRSRRKPASVITSSGIYKKPQHP
QKTHLAKYESTLQPPTCV
>ggSMO
GPCWLWALALGLALGPRRCPAAPLNASAAPPERCRRPAACERLRFGSCLGSALPYAHTSTLLAADSGSQEEAHGKLLLWSG
LRNAPRCWDVIQPLLCAVYMPKCEDGQVELPSQTLCQATRAPCTIVERERGWPDFLKCTPDRFPEGCPNEVQNIKFNSSGQ
CEAPLVRTYNPKSWYEDVEGCGIQCQNPLFTETEHREMHVYIAFSSVTISCTFFTLATFVADWRNSNRYPAVILFYVNACF
FVGSIGCVAQFMDGARDEIVCRADGTMRLGEPTSNETLSCVIIFVIVYYSLMSGVIWFVMLTYAWHTSFKALGTTYQPLLG
KTSYFHLITWSIPFVLTVAILAVAQVDGDSVSGICFVGYKNYRYRAGFVLAPIGLVLIVGGYFLIRGVMTLFSIKSNHPGL
LSEKAASKINETMLRLGIFGFLAFGFVFITFGCHFYDFFNQAEWERSFREYVLCEANVTIATQTNKPIPECEIKNRPSLLV
EKINLFAMFGTGISMSTWVWTKATLLIWKRTWCRLTGQSDDQPKRIKKSKMIAKAFSKRKELLRDPGRELSFSMHTVSHDG
PVAGLAFDINEPSADVSSAWAQHVTKMVARRGAILPQDVSVTPVATPVPPEERSNLWVVEADVSPELQKRSRKKKRRKKKK
EEVCPERRAGLSVAPLTPSSVPRLPRLPQQPCLVAIPRHRGDTFIPTVLPGLSNGAGGLWDGRRRAHVPHFITNPFCPESG
SPEDEENPGPSVGHRQHNGGRRWPPEPLPGGSGVTRTRGRRAGLAPIHSRTNLVNAELLDADLDF
>ggFZD9
ACDNPEKFQYVEKSLSCAPRCSPGVDVYWSREDKDFAFVWMAVWSTLCFVSTAFTVLTFLLDPHRFQYPERPIIFLSMCYN
VYSVAFIIRSVAGAETIACDRENGELYIIQEGLESTGCTIVFLILYYFGMASSLWWVVLTLTWFLAAGKKWGHEAIEAHSS
YFHMAAWGIPAMKTIVILTMRKVAGDELTGLCYVGSMDVSALTGFVLIPLSCYLVVGTSFILTGFVALFHIRKIMKTGGTN
TEKLEKLMVKIGVFSILYTVPATCVIVCYFYERLNVDYWNLRALERACVPLPGRRAADCSLEASVPTVAVFMLKIFMSLVV
GITSGVWVWSSKTLQTWQSLCNRKLGVRTRGKPCSGVSCGGVHCHYKAPTVMLHMTKTDPYLDNPTHV
>ggGRM7
MVDIVKALGWNYVSTLASEGNYGEKGVESFMQISREAGGLCIAQSLKIPQDRKDKTIDFDKIIKQLLETPNARAIVIFAND
EDIKQILAAAKRADQVGHFLWVGSDTWGSKVSPLLQQEDVAEGAITILPKRATIEGFDAYFTSRTLENNRRNVWFAEYWEE
NFNCKLTITGSKKEDTDRKCTGQERIGKDSHYEQEGKVQFVIDAVYAMAHALHHMSKDLCADYAGVCPEMEHAGGKKLLKY
IRSVNFNGSAGTPVMFNKNGDAPGRYDIFQYHTTNTSTPGYRLVGQWTDDLQLNIDDMQWGKGVREVPSSVCSLPCKPGER
KKMVKGMPCCWHCELCDGYQYQADEVTCLLCSYNERPNENRTGCRSIPIVKLEWHSPWAVIPVFLAMLGIIATIFVMATFI
RYNDTPIVRASGRELSYVLLTGIFLCYIITFLMIAKPNVAVCSFRRIFLGLGMCISYAALLTKTNRIYRIFEQGKKSVTAP
RLISPTSQLAITSSLISVQLLGVFIWFAVDPPNIIIDYDEQKTMNPDQARGVLKCDITDLQIICSLGYSILLMVTCTVYAI
KTRGVPENFNEAKPIGFTMYTTCIVWLAFIPIFFGTAQSAEKLYIQTTTLTISMNLSASVALGMLYMPKVYIIIFHPELNV
QKRKRSFKAVVTAATMSSRLSHKPSDRPNGEAKTELCENVDPNSDPAAKKKYVSYNNLVI
>ggGPR158
QHELGAAAGRGRAPGGRQPXYNHIKSRDPPPAPRSRSPESTILAQRLAEEVPQDVASFLYTGDSRELRRANCSGRYELASL
AGKSRFVSHPSLHGALDTLTHATNFLNMILQSNKSREQNLQEDLEWYRALIRSLLEGDPNISRAAITFSTEPFSAPQVFLQ
ASRHESQIHLQDLSSSAHRLANGSLETEWFQGLKRKWRPHQHRKVLNTGSKTLENSWKRREGFTADKNHIRWSSPYLECEN
GNYKPGWLVTLSAAFYGLQPNLIPEFRGVVKVDINLQKVDIDQCSSEGWFSGTHRCHLNNSECLPIKGLGFVLGAYKCICK
EGFYHPNIFSVNSFQRDLSEEVYTCLPCREGCSNCKDDTPCYAQEDKYLRLAIISFQTLCMLLDFISMLVVYHFRKAKSIR
ASGLVLLETILFGSLLLYFPVVILYFEPSVFRCVLLRWVRLLGFATVYGTVTLKLHRVLKVFLSRTAQRIPYMTGGRVMRM
LAVILLVVFWFLVGWTSAITQNLERNIPLIGQGQTSDHLIFNMCLMDRWDYMMAVAEFLFLLWGVYLCYAVRTVPSAFHEP
RYMAVAVHNELIISAIFHTIRQXFILASRLQSDWMLMLFFAHTHLTVTVTVGLLLIPKFSHSSNNPRDDIATEAYEDELDM
GRSGSYLNSSINSAWSEHSLDPEDIRDELKKLYAQLEIYKRKKMIANNPHLQKKRCSKKGLGRSIMRRITEIPETVTRQCS
RDEKDLMEHSAVKNTATLKKTPQDSTSSVKPKEETLKNRVFSLKKSHSTYDHVRDQTGEPNSITTESTEVTAAENSLLDSL
TGNNLTKKAEKAEAVSTESVPLVCKSLSAHNLSADKKPLHPRTSVLQKSLSVIASAKEKTLGLTGKTQSLEESAKSQKSQQ
KGKEGSKKHSAADKGEHKDSHRKNSTQSEETKKTHKSGIMKQQRVSQTPANPDTGPGKSLHKDTFDIGEVCPWEIYDQTPG
PVPSDSKIQKHVSIASSEAEKNHPSQPKGKSHHKQKTPEGYQQSNQQSPQKLEAGTREVQEQHIFENDKKQLNSKPQGSPG
LKRENVNRYAPNVCGGEREELPQKAAENVSKLVEQKKVASSEGNVLSDSRKPSGYSQQPLASRAEVCPWEFDTPDLPNAER
SVALSNTSAISANKTATPRK
>ggGPRC5A_5D
MTTQPPRGCGSIHADYFLLCDMDQAWGIVLESLAAAGVLVTVFLICSLFFLICKVQDNSKRHMISIYFFFLLGILGIFGIT
FAFIIRSNERIRPTRFFLFGVLFALCFSCLLIHASNLIRLVRGKKPFSWPVLLLLIVSFALVQVVINIEYLVTMLVKDRNT
FLNMASEEANKDFVMLLIYVLFLMALTFLVSIFTFCGPYKSWKRHGAHIFVTVLFSIAIWVVWITMLTRGNQSLGKTNWDD
PVVAIALVSNGWVFLIMYIIPEICFLTAPLKLGDYPPENDFCQPTFIKQSTGVDNHAYTQ
>ggGRM3
MFTRLQVLALALFSKGVFLSLSDHSFVRKEIKIEGDLVLGGLFPINEKGTGIEECGRINEDRGIQRLEAMLFAIDEINRDN
YLLPGIKLGVHILDTCSRDTYALEQSLEFVRASLTKVDETEYMCPDGSYAIQENLPLLIAGVIGGSYSSVSIQVANLLRLF
QIPQISYASTSAKLSDKSRYDYFARTVPPDFYQAKAMAEILRFFNWTYVSTVASEGDYGETGIEAFEQEARLRNICIATSE
KVGRSNIRKSYDGVIRELLQKPNARVVVLFMRGDDSRELIAAANRFNVSFTWIASDGWGAQESIVKGNEHIASGAITLELA
SHPVKEFDRYFQSLSPYNNRRNPWFKDFWEQKFQCNLQNRKPHKKTCDKHFTINSSNYEQESKIMFVVNAVYAMAHALHKM
QRTLCPNTTKLCDAMKHLDGKKLYKDYLLKVNFTAPFNPPNAADSTVKFDAYGDGIGRYNVFNFQYTGDRYSYVKVGHWAE
TLSLEVDSIHWSKSSVPVSQCSDPCAPNEMKNMQPGDVCCWICIPCETYEYLADEFTCADCGPGKWPTADLTGCYDLPEDY
IKWEDAWAIGPVTIACLGFICTFLVIAVFIKHNNTPLVKASGRELCYILLFGVFLSYSMTFFFIAKPSPAICTLRRLGLGS
SFAVCYSALLTKTNCIARIFDGVKNGAQRPKFISPSSQVFICLSLILVQIVMVSVWLILEAPGTRRYTLPEKRETVILKCN
VKDSSMLISLTYDVILVVLCTVYAFKTRKCPENFNEAKFIGFTMYTTCIIWLAFLPIFYVTSSDYRHLPVCTYQRSAMEGK
SWTPPPHLCDCDLQFSS
>ggGRM5
MVLLLFLAILLLKEDVCGNFGLLVSAQANERRVVAHMPGDIIIGALFSVHHQPTVDKVHERKCGEVREQYGIQRVEAMLHT
LDRINLDPTLLPNITLGCEIRDSCWHSAVALEQSIEFIRDSLISSEEEEGMVRCVDGSSSSFHSKKPIVGVIGPGSSSVAI

QVQNLLQLFNIPQIAYSATSMDLSDKTLFKYFMRVVPSDAQQARAMVDIVKRYNWTYVSAVHTEGNYGESGMEAFKDMAAK
EGICIAHSYKIYSNAGEQSFDKLLRKLRSHLPKARVVACFCEGMTVRGLLMAMRRLGLAGEFLLLGSDGWADRYDVTDGYQ
REAVGGITIKLQSPDVKWFDDYYLELRPETNHRNPWFQEFWQHRFQCRLEGFPQENPKYNKTCTSKPMTLRTQHVQDSKMG
FVINAIYSMAYGLHNMQLSLCPGYVGLCDAMKPIDGRKLLESLMKTNFTGVSGDMILFDENGDSPGRYEIMNFKKMGKDYF
DYINVGSWDNGELKMDDDEIWSEKNNIIRSVCSEPCEKGQIKVIRKGEVSCCWTCTPCKENEYVFDEYTCKACQLGSWPND
ELTGCDLIPVQYLRWGDPEPIAAVVFACLGLLATLFVTAIFIMYRDTPVVKSSSRELCYIILAGICLGYLCTFCLIAKPQQ
IYCYLQRIGIGLSPAMSYSALVTKTNRIARILAGSKKKICTKKPRFMSACAQLVIAFILICIQLGIIVALFIMEPPDIMHD
YPSIREVYLICNTTNLGVVTPLGYNGLLILSCTFYAFKTRNVPANFNEAKYIAFTMYTTCIIWLAFVPIYFGSNYKIITMC
FSVSLSATVALGCMFVPKVYIILAKPERNVRSAFTTSTVVRMHVGDGKSSSAASRSSSLVNLWKRRGSSGETLRYKGRRLA
PHKSEIECFTPKGSMGNGGRATMTSSNGKSVSWAQNEKSSRGAHLWQRLSIHINKKENPNQTAVIKPFSKSTDSSRHSSSA
TFPETSAKTLYDVSEAEEQYPAQYRPQTPSPISTVSHRTASVSRTEDDAPTFQSEPPQRSSSSQGSLMEQISSVVTRFTAN
ISELNSMMLSTATPGTMVATPLCSSYLIPREIQLPTTMTTFAEIQPLPSIEVNGASQSARKQSNGSVKEGTAETPSAKQDL
EELVALTPPSPFRDSIDSGSASPSSPVSESALCIPSSPKYDTLLIRDYTQSSSSL
>ggGRM1
MTAVLPLVLPALLLAGAALPPAAASAQRSVARMDGDVIIGALFSVHHQPPAEKVPERKCGEIREQYGIQRVEAMFHTLDKI
NADPVLLPNITLGSEIRDSCWHSSVALEQSIEFIRDSLISIRDDREGGGRCLSDSQPQPPGRLRKPIAGVIGPGSSSVAIQ
VQNLLQLFDIPQIAYSATSIDLSDKTLYKYFLRVVPSDTLQARAMLDIVKRYNWTYVSAVHTEGNYGESGMEAFKELAAQE
GLCIAHSDKIYSNAGEKSFDRLLRKLRERLPKARVVVCFCEGMTVRGILIAMRRLGVLGEFLLIGSDGWADRDEVIEGYEQ
EANGGITIKLQSPEVLSFDDYYLKLRLDTNTRNPWFPEFWQHRFQCRIPGHPLENPNFQKNCTGNESLEENYVQDSKMGFV
INAIYAMAHGLQNMHHSLCPGHVGLCDAMKPIDGSKLLEFLLKSTFIGVSGEEVWFDEKGDAPGRYDIMNLQYIEPNRYDY
VLIGTWHEGVLNIDDYRIQMNKSGMVRSVCSEPCLKGQIKVIRKGEVSCCWICTACKENEFVQDEFTCKACELGWWPNAEL
TGCEPIPVKYLQWSNIESIVAVVFSCLGILVTMFVTLIFVLYRDTPVVKSSSRELCYIILAGIFLGYVCPFTLIAKPTTIS
CYLQRLLVGLSSAMCYSALVTKTNRIARILAGSKKKICTRKPRFMSAWAQVVIASILISVQLTLVVTLIILEPPMPILSYP
SIKEVYLICNTSNLGVVAPVGYNGLLIMSCTYYAFKTRNVPANFNEAKYIAFTMYTTCIIWLAFVPIYFGSNYKIITTCFA
VSLSVTVALGCMFTPKMYIIIAKPERNVRSAFTTSDVVRMHVGDGKAPCKSNTFLNIFRRKKPGAGNPKWKTSFQGRAHWH
RLSVHVKNQETGCNQTAVIKPLTKSYHGQSKSLTFTDISSKTLYNVEEEEDREPVRLNQPSSPSMVVHRRVATTPPLQAAA
EETRLFLPEQSPKDLPLQPRSLMEQLQGVVNKLATGIPDFSAVLPAPGSGNGTRPPQQSPLLPLQAADFSGEPLSPPAEEA
ESEKLKLIQGYVYEKSTEDEEEEEPATSKLTLEDSPALTPPSPFRDSVASGSSVPSSPVSESVLCTPPDMTYASVILRDYK
QSSSTL
>ggGRM8
MVYEGKRLVSCHCFFLLTAKFCWILTLMQRTYGQEYAHSIRLDGDIILGGLFPVHAKGDRGVPCGELKKEKGIHRLEAMLY
AIDQINKDPDLLANITLGVRILDTCSRDTYALEQSLTFVQALIEKDGSDVKCANGDPPIFTKPDKISGVIGAAASSVSIMV
ANILRLFKIPQISYASTAPELSDNTRYDFFSRVVPPDSYQAQAMVDIVTALGWNYVSTLASEGNYGESGVEAFTQISREIG
GVCIAQSLKIPREPRPGEFEKIIKRLLETPNARAVIMFANEDDIRRILEAAKKANQSGHFLWIGSDSWGSKISPVQHQEEI
AEGAVTILPKRTSIDGFDRYFRSRTLANNRRNVWFAEFWEENFGCKLGSHGKRNSNIKKCTGLERIARDSAYEQEGKVQFV
IDAVYSMAYALHNMHKDLCPGYIGLCPRMSTIDGKELLTYIRAVNFNGSAGTPVIFNENGDAPGRYDIFQYQITNKSTEYK
IIGHWTNQLHLNVEDMQWANREHTHPASVCSLPCKAGERKKTVKGVPCCWHCERCEGYHYQVDEFNCELCPINKRPNANRT
DCQLIPIIKLEWHSPWAIVPVFIAILGIIATTFVIVTFVRYNDTPIVRASGRELSYVLLTGIFLCYSITFLMIATPDTVIC
SFRRIFLGLGMCFSYAALLTKTNRIHRIFEQGKKSVTAPKFISPASQLVITFSLISLQLIGVFIWFAIDPPHTIVDYGEQR
TLEPENARGVLKCDISDLSLICSLGYSILLMVTCTVYAIKTRGVPETFNEAKPIGFTMYTTCIIWLAFIPIFFGTAQSAEK
MYIQTTTLTVSMSLSASVSLGMLYMPKVYIIIFHPEQNVQKRKRSFKAVVTAATMQSKLTQKGNDRPNGEVKTELCESLET
NS
>ggGPRC5B
MKTYPAIGFFVLFVISYGSSENSSTPRGCGLDLLPQYVYLCDLDAIWGIVVEAVAGAGVLTTLLLMLILLVRLPFIKEKEK
KSPLGMHFLFLFGTLGLFGLTFAFIIQEDEMVCSTRRFLWGVIFALCFSCLLAQAWRLRRLVRHGKSPSGWHLAGVAICLM
LVQVIIATEWLILTVVRDNKMACSYEPMDFAMALIYVMFLMVFTMGFSLFTLCGKFKKWKKNGVCLIITLFFSILIWVAWM
TMYLFGNAELKRRDKWSDPTLAIALVSSGWVFVIFHAIPEVHCTILPSQQENTPNYFDTSQPRMRETAFEEDIQLPRSYME
NKAFSMDEHNAERSLFLNAALRTAGFRNGSLGSRPSAPFRSNVYQPTEMAVVLNGGT
>ggGPRC5C
MGAAVPAATCVLLALLPMGRAQNTPPSGCGQDLSSLYYNLCDLSAAWGIVLEAAASLGAVTSFVLTIVLVASLPFVQDPQK
KSLVATQAFFLLGTFGLFCLTFDFIVGPDFSTCTSRRFLFGVLFALCFSCLLAHAVALNFLARRNRGPRGWVTLAVALLLA
LVEVIINAEWLIITVARQERGPVDPCELADADFVMALIYVMVLLVAAFGTAWPALCGRYTRWRKHGAFILATSGLSMAIWV
AWTAMYLYGNQHVGKKPIWDDPTLAIALVSNAYAFILLYVIPEVTHVTRRSSEQGYEDDVYPTRGVGYETILKEQKSQSMF
VENKAFSMDEPSF
>ggGPRC6A
MALFSLALIPFVISSDAASACQNTDDFVGASSPGDIIIGGLFAVHSEMLQPEEHPIKPVIQNCAGFEIQIFLQTLAMIHAI
EMINNSTLLSGVTLGYEIYDTCAEVTKAMASALRFLSKSNTSKDIVEFKCNYSDYVPRIKAVTGASYSEVSMAVSRLLALQ
LIPQVSPASSAEILSDKIRFPSFLRTIPSDFHQTRAMAHLICESGWNWIGVIATDDDNGRFALESFGVQAMANSVCIAFKE
MLPAYLSDNTFHTKVDRAVEKIVKETRVNVIVVFMRQFHVLKLFKKAIERNVKKIWIASDNWSTAVKISTMPNIRKLGTVV
GFGFKNKDLSTFQDFLRNLHDRPTENNKFLLEYIMLLSVCAHLDNYDFQMCISSQSQYDLMQNVENKHQIWRDDFLNANIE
PGFIHSTILAVYAIAHAIKGQCKDRNCKNPSAFAPWELLEELKKVTIIDDDKEIKFDSKGDLSSGYDVLLWKEVDGRMEIT
TMAEYDPENGYFIFEDEEKKKEFLDLKKVPSTCSQHCRPGQMKKVTESPHTCCYECVYCPENHYSNQTDMDYCYRCHNKTY
WAPVNSTTCYRKTIHFLGWTDWFAIFLLLLSAFGVVLIFSISAIFTKNLSTPVVKASGGLTVCYIILLSHFFIFLSTVFFI
GEPTEFKCRTRQALFGISFALCISCILIKSLKILLAFSFDPKLQNFLKCAYKPITIVFICTGIQVIICTFWLIFRTPFVKQ
NFSIPRAIILECNEGSVVAFGIMLGYIAALAFICFICAFKGRKLPENYNEAKFITFGMLIYFIAWIVFIPVYVTTFGKYLP
AVEIIVILISNYGILCCTFFPKCYIIIYKQETNTKSAFLKMIYTYSSKSVGSIAVSQISLDSKSSSSRITESDSCNAEKSS
VNGNCHFQVSGQTPVKEKAVPKRATRTLSRKRLSSI
>ggGABBR2

CDNAKGLKAFYDAIKYGPNHLMVFGGVCATVTSIIAESLKGWNLVQLSFAATTPELADKKKYPYFFRTVPSDNAVNPAILK
LLKYYQWKRVGTLTQDVQRFSEVRNDLTGVLYGEDIEISDTESFSNDPCTSVKKLKGNDVRIILGQFDEEMAVKVFCCAYV
EEMYGSKYQWIIPGWYENLWWESWINSSHCLSKNLLAAMEGYIGVDFEPLSSKMNKTISGRTPQQYEREYNAKRGDGQSSK
FHGYAYDGIWVIAKTLQRAMKYLNATNKHQKIEDFNYTNHKLGKIFLDAMNETNFFGVTGQVVFRNGERMGTIKFTQFQER
KEVKVGEYNAVADTLEIINNSIRFQGLEPPKDKTIIQEELRKISLPLYSILSALTILGMIMASAFLFFNIKNRNQKLIKMS
SPYMNNLIILGGMLSYASIFLFGLDGSFVSEKTFETLCTVRTWILTVGYTTAFGAMFAKTWRVHAIFKNVKMKKKIIKDQK
LMVIVGGMLLIDLCILICWQVVDPLRRTVEKYNMEPDPAGRDISIRPILEHCENTHMTIWLGIVYAYKGLLMLFGCFLAWE
TRNVSIPALNDSKYIGMSVYNVGIMCIIGAAVSFLTRDQPNVQFCIVALVIIFCSTITLCLVFVPKLITLRTNPDAATQNR
RFQFTQNQKKEDSKTSTSVTSVNQASTSRLEGLQSENHRLRMKITELDKDLEEVTMQLQDTPEKTTYIKQNHYQDLNDILS
IRNFTDSKDGEKAILKNHLDQNPPAQWGTSDPSRTSKDPIEDINSPEHIQRRLSLQLPILHHAYLPSIGGVDASCASPCVS
PSTSPRHRHVPPSFRVMVSGL
>ggTAS1R1
LISYEASLEMLSTKRFYPSFLRTIPSDGQQVKAIGLLLQRFGWTWVALVGSDNTYGRDGLNALSELLAASDVCVAYRGVIP
TTKDAGSPELRKLIQTLVDSRVNVTVVFSNRRNAQPFFEAVVQENITGMVWVGSEDWSLAQTIWQVPGIQNIGSVIGISVE
QAEPTMLKRLESWENARERAVSGSAGSTGVGGGNGASSSDSVQLNCTQHCPGCHLLADTPDIYDIQASYNVYSAVYAVAHG
LHNLLGCASGVCSKGRVYPWQLLQKIKQVNFSLHKSYISFDANGNIRKGYNIIAWNWRGQSWAFDVVGAFSVNPNRLHIDQ
SKILWHTKDHQVPVSVCSWPCAAGEMRLQQNRHRCCFSCVACPAGTFLNRTALYACQACGRDEWAPVGSETCFNRTVEFLS
WADPLSWVLLIPTVLLLLLMVGLAVLFARNASTPVVRSAGGKMCFLMLGALACACSSIFFNFGEPTWLSCLVRIPLFTISF
AVFLSCVATRCFQIVCIFKLSTRWPALHEAWQRRGGPALFIAGSTVAQAVLSVAAVASGPAGPRRRYSVAAERVVLECGAG
SAPGETAAILYNLLLSLGCFALSYAGKDLPADYNEAKCLTCSLLLHLACSAAVLCTRSYFRGRSAAVTAALGALGTLAPLL
SGYFLPKGFVVLLRPHLNTAERFQQEIRSYTRRRDE
>ggTAS1R3
MIPWVLLCMSFGCAAALKPSCLSAQFRRPGDYIIGGLFPFGMDTINLTARSEPTLIVYHKSTSLLPGVELGYDIYDTCFEP
LAALQPSLLFVTQNGTTGIGIACNYTDYQPRVTAVIGPHKSDLCLLTAKLFSFFLIPQVSYGASSEKLSNKELYPSFYRTV
PSDKNLVEAVVLLLDEFGWNWIATIGSDDEYGRGAQELFLSTIGNSSICIAYEGLIPSDLTDPRAEKQLEETIQYINKTNV
NIIVLFAFRQPAQALLKQSIKMRLSKKVWIGTEAWLLSDIAASIPNIQNIGTVLGFIMKASTVPGFQKYVANLLSSVQQDE
FCQKSRGFYRHVSSDTLGTQCQQCDHISLNDISSTLSHSQIQPVYIAVYSVAYALHRALGCTHQGCPRASIRSWQLLHFMN
TVPFTVNGQSFRFDESHGTNSGYKLIFWHWENGSLTHLPVGDYQESLYLNKSLIQFHTTDQKEPTSECFRECEPGQIKQIK
GFHLCCYDCTDCPENTFCSSKDSSTCTPCLEHQWSPARSTQCYDRSERYLRWNEPLTAGLLISMSIIISLICLTAVLFVKN
LNTPLVQAAGGNLNLFALFALTLMCLSSCLFIGKPTNNLCMMQQIVCALCLNACFSTFFIKSLEIVLLTEFPRCARTALRW
VTPSRSWLLVALCLLTECLFCFCYLHLGPDYVLPDYSSLPTEVLLMCSTASWPAFALMHGYNGCLAFVCFLCTFMVQSSGK
KYNMARGITFTILIYFIIWIFFITVFATLRTVLMSVIQISTILMVSLGIVGTYYIPKCYILLLKPDLNREDYFQYSTKEEP
EGDSQ
>ggCASR
MTLYSCCLILLLFTWNTAAYGPNQRAQKKGDIILGGLFPIHFGVAAKDQDLKSRPESVECIRYNFRGFRWLQAMIFAIEEI
NNSPNLLPNMTLGYRIFDTCNTVSKALEATLSFVAQNKIDSLNLDEFCNCSEHIPSTIAVVGATGSGVSTAVANLLGLFYI
PQVSYASSSRLLSNKNQFKSFLRTIPNDEHQATAMADIIEYFRWNWVGTIAADDDYGRPGIEKFREEAEERDICIDFSELI
SQYSDEEEIQQVVEVIQNSTARVIVVFSSGPDLEPLIKEIVRRNITGKIWLASEAWASSSLIAMPEFFRVIGSTIGFALKA
GQIPGFREFLQKVHPKKSANNGFAKEFWEETFNCYLPSESKNSPASASFHKAHEEGLGAGNGTAAFRPPCTGDENITSVET
PYMDFTHLRISYNVYLAVYSIGDXFXXIYTLPPXKXLFTNESCATFRXXXXXXVLKHLRHLNFTSNMGEQVDFDEFGDLVG
NYSIINWHLSPEDGSVVFEEVGHYNVYAKKGERLFINENKILWSGFSKEVPFSNCSRDCLPGTRKGIIEGEPTCCFECVDC
PDGEYSDETDASACDKCPEDYWSNENHTSCIPKQIEFLSWTEPFGIALTLFAVLGIFLTSFVLGVFTKFRNTPIVKATNRE
LSYLLLFSLLCCFSSSLFFIGEPQNWTCRLRQPAFGISFVLCISCILVKTNRVLLVFEAKIPTSLHRKWWGLNLQFLLVFL
CTFVQIVICVIWLYTAPPSSYRNHELEDEIIFITCHEGSLMALGFLIGYTCLLAAICFFFAFKSRKLPENFNEAKFITFSM
LIFFIVWISFIPAYASTYGKFVSAVEVIAILAASFGLLACIFFNKVYIILFKPSRNTIEEVRCSTAAHAFKVAARATLRRS
NVSRKRSNSLGGSTGSTPSSSISSKSNHEDPFPLPASAERQRQQQRGCKQKVSFGSGTVTLSLSFEEPQKNAMANRNAKRR
NSLEAQNSDDSLMRHRALLALQNSESLSAEPGFQTASSPETSSQESVVGDNKEEVPNPEAEPSLPSANSRNFIGTGGSSVT
ENTVHS
>ggCOR7b
MEEKNSTQINIFLLQGFPGLMDLKVLLFIVLLLTYIVTVVENVLIISLIKANHELCKPMYFFLGQLSFIEVWYISVIVPKL
LANFIAEDRSISFAGCMTQLYFFSSFLCTECVLLSAMAYDRYVAICQPLHYLFIMTYQMCVCLIALSWFTGFTVSLIKISF
ISQLEFCGSHVINHFFCDISPVLNLACTDMSVAEMVDFVLALFILLVPLSITVVSYLLIITTILRIPTTQSKKKAFSTCAS
HLTVVVIFFSATLFMYARPKKISSYDMNKLVSAVYTIVTPMLNPFVYCLRNQGLKRAFKKALSVKITLSEVSRTIPSLQM
>ggOR1
MPNSSSISEFLLLALADTRQLQLLHFWLLLGIYLAALLGNGLISTAVACDHRLHTPMYFFLLNLALLDLGCISTTLPKAMA
NALWHTRHISYAGCAAQVFFFLFFLAAEYCILTIMSYDRYVAICMPLHYGTLLGSRACATMAAAAWGTGALYALLHTATAF
SVPLCQGNAVDQFFCEIAQILKLSCSKSDYLREVGIIYFSLCLVCGYFVFIVVSYMQIFRVVLRMPSEQGWHKAFSTCLPH
LAVVSLFVSTAMFA
>ggOR2
MPNSSSISEFLLLALADTRQLQLLHFWLLLGIYLAALLGNGLISTAVACDHRLHTPMYFFLLNLALLDLGCISTTLPKDMA
NALWHTRHISYAGCAAQVFFFVFFLSAEYSLLTIMSYDRYVAICKPLHYGTLLGSRACATMAAAAWVSGLLYSLLHTASIF
SLPLCRGNAVDQFFCEIPQNLKLSCSGSEFLRELGLLSTSAFIGIGCFVFIVVSYVQIFRAVLRMPSEQGRHKAFSTCLPH
LVVLSLFVSTAMLEST
>ggOR3
MPNSSSISEFLLLALADTRQLQLLHFWLLLGIYLAALLGNGLISTAVACDHRLHTPMYYFLLNLALLDLGCISTTLPKAMA
NALWHTRDISYAGCAEQVLLFVFFFSAEYYLLTVMSYDRYVAICMPLHYATLLDSRACATMAAAAWGTGVLNSLLHTANTF
SLPLCQGNAVDQFFCEIPQILKLSCSDAYLREAGLIVAGVIIFFGCFAFILFSYVQILRAVLRIPSEQGQHKAFSTCLPHL
AVVTLFLSTAFFAGLRPPSLSSKSLDLTMA

>ggOR4
MPNSSSISEFLLLALADTRQLQLLHFWLLLGIYLAALLGNGLISTAVACDHRLHTPMYFFLLNLALLDLGCISTTLPKAMA
NALWHTRDISYAGCAAQVFFFSFLFSAEYCILTLMSYDRYVAICKPLHYGTLLGSRACATMAAAAWGYGILNSLLHTANTF
SLPLCQGNAVDHFFCEIPPILKLSCSKSYLREFGLIMAGGLVFFGCFLFIVVSYVQIFRAVLRIPSEQGRHKAFSTCLPHL
AVVSLFVSTAMFAYLKPHSISSPSLDLGVSVLYSGGTSTSEP
>ggOR5
MPNSSSISEFLLLALADTRQLQLLHFWLLLGIYLAALLGNGLISTAVACDHRLHTPMYFFLLNLALLDLGCISTTLPKAMA
NALWHTRAISYAGCATQLFFFLFLFSAEYCILTIMSYDRYVAICKPLHYGTLLGSRACATTAAAAWGTGLLYSLLHTANTF
SLPLCQGNAVDQFFCEIPQILKLSCSDAYLREVGLIVFSALVLFGCFVFILFSYVQIFRAVMRMPSEQGRHKAFSTCLPHL
AVLSVFVSTAFLANLRPPSLSSRSLDLTIAVLYSGVPPAVNPVMHRMRNQELKAAVRKMMACTHYSREKLLHCLNKGIT
>ggOR6
MANSSSISEFLLLALADTRQLQLLHFWLLLGIYLAALLGNGLISTAVACDHRLHTPMYFFLLNLALLDLGCISTTLPKAMA
NALWHTRHISYAGCAAQVFFFVFFISAEYSLLTVMSYDRYVAICKPLHYGTLLGSRACATMAAAAWGTGLLYSLLHTANTF
SLPLCQGNAVDHFFCEIPQILKLSCSESEFLRELGLLSTSAFIGVGCFVFIVVSYMQIFRAVLRMQSEQGRHKAFSTCLPH
LAVLSLLISSGFFAYLKPYSISSPSLDLVVSFLYSVVPPAVNPLIYRRRNQELKDALWKLMTKYIWKAMNCPSLGR
>ggOR8
WILLAALLGNGLISTAVACDHRLHTPMYFFLLNLALLDLGYISTTIPQAMANALWDTRAISYAGCAAQVFFSVFFFSAEYS
LHTIMFYDRYVAICKPLHYGTLLGSRACATMAAAAWGTGLLYSLLHTANTFSLPLCQGNVLDQFLCEIPQILKLTCSDAYL
KEAGLIVISVLVILGCFVFIVVSYVQIFRAVLRMPSEQGRHKAFSTCLPHLAVLSLLISSASFAYLKPPSISTPSLNLVVS
FLYTVVPPTLNPLIYSMRNQELKDALGKLMISCILKAMYCPSLCIHDL
>ggOR9
MPNSSSISEFLLLALADTRQLQLLHFWLLLGIYLAALLGNGLISTAVACDHRLHTPMYFFLLNLALLDLGCISTTLPKAMA
NALWDTRAISYAGCAAQVFFYLFLFSAEYCLLTIMSYDRYVAICKPLHYGILLGSRACATMSAAAWCTGVLYSLLHTANTF
SLPLCHGNAVDQFFCEIPHILKLSCSDAYLREIWLLGVSASLVSGCFAFILFSYVQIFKTVLKMPSEQGRHKAFSMCLP
>ggOR10
MPNSSSISEFLLLALADTRQLQLLHFWLLLGIYLAALLGNGLISTAVACDHRLHTPMYFFLLNLALLDLGCISTTLPKAMA
NALWHTRAISYAGCATQLFFFLFLFGAEYSLLTIMAYDRYVAICKPLHYGTLLGSRTCATMAAAAWGSGVLHAVLHTASTF
SLPLCQGNAVDQFFCEIPHILKLSCSGSYLRELELVIFSILIFFGCFVF
>ggOR11
MPNSSSISEFLLLALADTRQLQLLHFWLLLGIYLAALLGNGLISTAVACDHRLHTPMYFFLLNLALLDLGCISTTLPKAMA
NALWHTRAISYAGCATQVFLFLFLFSAEYSLLTIMSYDRYVAICKPLHYGTLLGSRACATMAAAAWGTGVLNSLLHTANTF
SLPMCQGNAVDQFFCEIPQILKLSCS
>ggOR12
MPNSSSISEFLLLALADTRQLQLLHFWLLLGIYLAALLGNGLISTAVACDHRLHTPMYFFLLNLALLDLGCISTTLPKAMA
NALWHTRHISYAGCAAQVFFFLFLYSTEYYLLTLMAYDCYVAICKPLHYETLLSFRSCATMAAAVWGSGLLFSLLHTSNTF
SLPLCQGNAVDQFFCEIPHILKLSCSDAYLREVGLIVAFFHVLFGCFVFIVVSYVQIFRAVLRMPSEQGRHKA
>ggOR13
NSSSSISEFLLLALADTRQLQLLHFWLLLGIYLAALLGNGLISTAVACDHRLHTPMYFFLLNLALLDLGCISTTLPKAMAN
ALWHTRAISYAGCAAQVFFLLFLFGAEYSFLIVMSYDRYVAICKPLHYGTLLGSRACATMAAAAWGTGLLCSLLHTANTFS
LPLCQGNVVNQFFCEIPQILKLTCSKSYLSEFGLIMLSALIFFGCFAFILFSYVQIFRAVLRMPSEQGRHKAFSTCLPHLA
VLSLFLSTVMFAYLKPFSISSQSLDLTVALLYSVIPPTLNPIIYSMRNKEIKHALSKVLPYALYQI
>ggOR14
MSYDRYVAIPKPLHYGTLLGSRACATMAAAAWGTGLLCSLLHTANTFSLPLCQGNVVNQFFCEIPQILKLTCSKSYLSEFG
LIMLSALIFFGCFAFILFSYVQIFRAVLRMPSEQGRHKAFSTCLPHLAVLSLFLSTVMFAYLKPFSISSPSLDLTVALLYS
VVPPALNPIIYSMRNKEIKHALSKVLQYALFWIQ
>ggOR15
MSYDRYVAICKPLHYGTVLGSRACATMAAAAWGTGLLCALLHTANTFSLPLCKGNAVDQFFCEIPQLVKLSCSKSYLREDG
LLLFSICLSFGCFVFIVVSYVQIFRAVLRMPSEQGRHKAFSTCLPHLAVVSLLVSMGFLAYVKPHSQSSPFLDVTVALLYS
VVPPTLNPIIYSMRNQEIKHALRKLLQYVHYSSFNKVPIFLT
>ggOR16
FLSLLPRSRILSSHLMSYDRYVAICKPLHYGTLLGSRACATMAAAAWGTGLLYSLLHTANTFSLPLCQGNAVDQFFCEIPQ
ILKLSCSDAYLREVGLLLFSFCVVFGCFVFIAVSYVQIFRAVLRMPSEQGRHKAFSMCLPHLAVVSLFVSTAIFAYLKPPS
ISSPFIDLVVSFLYTVVPPALNPLIYSMRNQELKGSLRKILQHTRFHYQKDVHHALKTTENLGKA
>ggOR17
GCISTTLPKAMANALWHTRHISYAGCAAQVFFFLFFISAEYFLLTVMSYDRYVAICKPLHYGTLLGSRACATMAAAAWGSG
LLYALLHTAYTFSLPLRQGNAVDQFFCEIAQILKLSCSKSDYLSEVGIIYFSLCLICGCFAFILFSYVQIFRAVLRMPSEQ
GRHKAFSTCLPHLAVVSLFVSTAMFAYLKPPSISSPSLDLLVSFLYSVVPPAVNPLIYGLRNQELKAAVSNTYFYSIRKMH
GSLTGVPR
>ggOR18
LSPKAMANALWRTRAISYAGCAAQVFFFVFFLSAEYSLLTVMSYDRYMAICKPLHYRTLLGSRACATMAAAAWGSGLLHAV
MHTANTFSLPLCQGNVVNQFFCEIPHILKLSCSESNYLREVGRIVVSVLLELGCFVFIVVSYVQIFRAVLRMPSEQGRHKA
FSTCLPHLAVLSLLVSSAFFAYLKPSSISSPSLNLVVSFLYSVVPSAVNPIIYSMRNQELKDALWKLMTSCILKAMNFPSL
SIHDL
>ggOR19
AGCAAQVFFFLFFISAEYCILTIMSYDRYVAICKPLHYGTLLGSRACATMAAAAWGTGLLYSLLHTANTFSLPLCQGNAVD
QFFCEIPQILKLACSESEFLRELGLLSTSAFIGVGCFVFIVVSYVQIFRAVLRMPSEQGRHKAFSTCLPHLAVLSLFVSTV
VFAYLKPPAISFQSLDLVVSFLYTVGPPTVNPLIYSMRNQKLKDALWKLVTSCILKTMNCPSLSVHDL
>ggOR20

AQVFFLLFLFGAEYSLLTIMSYDRYVAICKPLHYGTLLGSRACATMAAAACGTGVLNSLLHTANTFSLPLCQGNAVDQFFC
EIPHILKLACSEYYLREVGLNVISALVLFGCFAFILFSYVQIFRAVLRMPSEQGRHKAFSTCLPHLAVLSLFLSTVVFAYL
KPFSISSSFLDVVVAFLYSVVPPTLNPIIYSMRNQDIKHALSKVLQCALFQLQ
>ggOR21
LCCTLPVHFPCLCATGNAVDQFFCEIPHILKLSCSKSFKAHLREVGFLLFSVCLAFVFFVFIVVSYVQIFRAVLRMPSEQG
RHKAFSTCLPHLAVVSLFLSTGSFAQLKPFSISSPFLDLMVSFLYSVLPSMLNPIIYSMRNKEVKHALSKVLQNVHYSSFN
KMRICLT
>ggOR22
AAPALLALAGHLKAALLGNGLISTALACDHRLHTPMYFFLLNLALLDLGCISTTLPKAMANALWHTRAISYAGCAAQVFFL
LFLLSAETFLLTIMSYDRYIAICKPLHYGTLMDSRACATMAAAAWATGVLYSLLHTANTFSIPLCQGNVVNQFFCEMAQVL
KLSCSESDYLIEAVIIDFSLSLVFGCFVFIVVSYVQIFRAVLRMPSEQGRHKAFSICLPHMSVVSLFVSTLMFAYLKPPSI
SSPSLDLVVAVLYSVVPPTVNPLIYSMRNQELRECPKKLLGNPRNIKNIKTVF
>ggOR23
CDHRLHTPMYFFLLHFALLDLGCISTTLPKAMANALWHTRAISYAGCAAQVFFFAFFISAEYSILTVMSYDRYVAICKPLH
YRTLLGSRACATMAAAAWGTGILHSLLHTASTFSLPLCQDNAVDQFFCEIPQILKLSCSDAYLREIWLLGVSASLVSVCFV
FILFSYVQIFRAVLRIPSEQGRHKAFSTCLPHLAVVTLFLSTAFFASLRPPSLSSQTLDLTMAVLYSVVPPTLNPLIYSMR
NQELKDAVRKMISWTHFSRDKFLICLYK
>ggOR24
ANALWHTRHISYAGCAAQVFLFLFFFGSEYCILTIMSYDRYVAICKPLHYGTLLGSRACATMAAAAWGTGLLYSLLHTAST
FSLPLCQGNAVDQFFCEIPQILKLSCSGSEFLRELGLLSTSAFIGVGCFVFIVVSYVQIFRAVLRIPSEQGRHKAFSTCLP
HLAVVTLFLSTAFFANLRPPSLSSQALDLTMAVLYSVVPPTLNPLIYSMRNQELKDAVRKMISWTHFSRDKLLICLYK
>ggOR25
ALLGNGFISTAVACDHRLHTPMYFFLLNLALLDLACISTTLPKAMANALWHTRHISYAGCAAQVFFFFFLFGAEYCILTIM
SYDRYVAICKPLHYGTLLGSRACATMAAAAWGAGVLNSLLHTASTFSLPLCQGNAVHQFFCEIPQILKLSCSQSYLKEISL
FVFSISLASGCFVFILFSYVQIFRAVLRMPSEQGRHKAFSTCLPHLAVVSFSLSTATFAYLKPPSLSSPSLDLVVAVLYTV
VPPVMNPVIYSMRNQEIKQALRTLFGDTLLRHH
>ggOR26
AEYSLLTIMSYDRYIAICKPLHYGTLLGSRACATMAAAAWGAGVLNSLLHTASVFSLPLCQGNAVDQFFCEIPQILKLSCS
ESYLREVELIVFSVAMEFGCFAFILFSYVQIFRAVLRMPSEQGRHKAFSTCLPHLAVLSLLVSTGFLAYLKTPSLFSPSLN
LLVSFLYSVVPPTLNPLIYSMRNEELRHAVKKTMSWTYFIRDKLFICLYK
>ggOR27
GITRQLQLLHFWLLLGPFMAALLGNGLISTFKACDHRLHTPMYFFLLNLALLDLRCISTTLPKAMANALWDTRHISYAGCS
AQVFFFFFFLSAEYSLLTIMSYDRYVAICKPLHYGTLLGSRACATMAAAAWGTGLLYSLLHTATAFSVPLCQGNLVDQFFC
EIAQILKLSCSRSDYLREVGIIYFSLCLICGCFVFIVVSYVQIFRAVLRMPSEQGRHKAFSTCLPHLAVVSLFLSTAMFAY
LKPPSMSSPSLDLLVSFLYSVVPPAVNPLIYGLRNQELKAAVSKTYIFNSHNMHTSLTGVSR
>ggOR28
KAMANALWHFRAISYAGCAAQVFFFLFLLSAEYSLLTVMSYDRYIAICKPLQYGTLLDNRACATMAAAAWGTGLLHAVLHT
ANTFSQALCRGKAVNQFFCEIPHILKLSCSESDYLRETGFIVVTVLALFGCFAFILFSYVQIFRAVLMMPSEQGRHKAFSM
CLPHLAVVSLFVSTDFLAYLKPHSLVSPSLDLVVSFLYSVVPPALNPLIYSMRNQELKDAVRKIMSWTHFSSNKLLICLYK
.LTVFLTAGLFLFFYVHSIYLFI
>ggOR29
MANALWDTTAISYAGCAAQVLLFVFFFSAEYYLLTIMSYDRYVAICKPLHYGTLLGSRACATMAAAAWGTGVLNSLLHTAN
TFSLPLCQGNAVDQFFCEIPQILKLSCSDAYLQEAGLIVAGVIIFFGCFAFILFSYVQIFRAVLRMPSEQGRHKAFSTCLP
HLAVVSLFLSTAFVANLRPPSLSSQSLDLTMAVLYSVVPPTLNPLIYSMRNQELKDAVRKMIAWTHFSRVKLLICLYK
>ggOR30
GCISTTLPKAMANALWHTRAISYAGCAAQVFFFLFFLAAEYSILTIMSYDRYVAICKPLHYGTLLGSRACATMAAAAWGTG
LPNAVLQTANTFSLPLCQGNAVDQFFCEIPHILKLSCSESYLREVGLLLFSVCLAVGCFVFIVVSYVQIFRAVLRMPSEQG
RHKAFSTCLPHLAVVSLFLSTGMVAYLKPPSISSASLDLVVSFLYSVVPPTVNPVIYSMRNQKLKDALWKVMTKYILKAMN
CPSLSMLDL
>ggOR31
TPRPSPMQPVCTGLFFLFFLSAEYSLLTIMSYDRYVAICKPLHYGTLMDSRACATMATAAWGSGLLYSLLHTASVFSVPLC
QGNAVDQFFCEIPQILRLSCSESEFLRELGLLSTSAFIGVGCFVFIVVSYVQLFRAVLRMPSEQGRHKAFSTCLPHLAVVS
LFLSTGMAAYLRPSSISSPFLDLVVSFMYSVVPPTLNPIIYSMRNQELKNSLWKLMTRCIMKTMNCSS
>ggOR32
AMANALWHTRAISNAGCAAQVFFLLFLFGAEYSLLTLMSYDRYVAICKPLHYGTLLGSRACATMAAAAWGTGLLYSLLHTA
NTFSLPLCQGNVVNQFFCEIPQILKLTCSKSYLSEFGLIMLSALIFFGCFAFILFSYVQIFRAVLRMPSEQGRHKAFSTCL
PHLAVLSLFLSTVMFTYLKPFSISSPFLDLTAALLYSVVPPTLNPIIYSMRKKEIKHALSKLLPHALFRIQ
>ggOR33
LRHRLPPPCTSSSSTSSSSTWAASPPLSPKPMANALWHTRAISYAGCAAQVFFFLFFISAEYSLLTIMSYDRYVAICKPLH
YGTLLGSRACATMAAAAWGTGLLYSLLHTANTFSLPLCQGNAVDQFFCEIPQILKLSCSDSYLREIWLLGVSASLVSGCFV
FILFSYVQIFRAVLRIPSEQGQHKAFSTCLPHLAVVFLFLSTGMVAYLKPHSETSSFLDLLVSLLYSVMPPTLNPLIYSLR
NKELMCSVRKVVS
>ggOR34
LLPGCAAQVFSISALISAEYSLLALMSYDRYIAICKPLHYGTMLGGTACATMAAAAWGTGLLFSLLHTANTFSLPLCQGNA
VDQFFCEIPHILKLSCSESDYFREAGFIMAGVIPFFGCFVFIVVSYVQIFRAVLRMPSEQGRHKAFSMCLPHLAVVSLFLS
TAIVAYLKPLSISSPPMALLFSFLYSVVPPALNPLIYGMRNQELKVALRKILQHTLFHHQKDVHHALKTTKNLGKA
>ggOR35
GCAAQFFFFSFLFQAEYSLLTVMSYDRYVAICKPLHYGTLLGSRACATMAAAAWGIGILNSLLHTANTFSLPLCQGNAVDQ

FFCEIPPILKLSCSKSYLREFGLIMAGGLVFFGCFVFIVVSYVQIFRAVLRMPFEKGRHKAFSTCIPHLAVVSLFVSTAML
AYLKPHSISSPSLDLVVSVLYSVVPPAVNPLIYSMRSQELKDALRKVRQYTLFLHQKDVQHTLRTTKI
>ggOR36
FLLNLALLDLGCISTTLPKAMANALWHTRHISYAGCAAQVFFYDFFISAEYSILTIMSYDRYVAICKPLHYGTLLGGRACA
TMAAAAWGTGLLYALLHTANTFSLPLCQGNAVEQFFCEIPQILKLSCSDSYLREVGFIVLSILAIFGCFVFIVVSYVQIFR
AVLRMPSEQGRHKAFSMCLPHLAVLTLLVSTATFANLKPPSFSSASMNLVVSFLYSVMPPAVNPLIYSMRNQELKDTLKKL
MSWTLFINNKLLISC
>ggOR37
IPLPKAMANALWHTRAISYAGCVAQLFFFLFFLSAEYSILTVMSYDRYVAICKPLHYGTLLGSRACATMAAAAWGTGLLNS
LLHTANTFSLPLCQGNAVDQFFCEIPQILKLSCSKYYLREVGLIVFNVGMVFGCFIFIVVSYVHIFRAVLPSEQGWHKAFS
TCLPHLAVLSLLVSTGMVAYLKPPSFSYPSLDLVVSFLYSVVPPALNPLIYSMRNQEIKDALWKLMTKRLLKAMNCPSLPI
>ggOR38
MYFFLLNLALLDLGCISTTLPKAMANALWHTRHISYAGCAAQVFFYDFFISAEYSILTIMSYDRYVAICKPLHYGTMLGGT
ACATMAAAAWGTGLLFLLLHTANTFSLPLCQGNAVDQFFCEIPHILKLSCSDAYLREVGLIVAFFHVLFGCFVFIVVSYVQ
IFRAVLRMPSEQGRHKAFSTCLPHLAVVSLFVSTTTFAYLKPPSISSPSLNLLVSVLYSVVPPALNPLIYSMRNQELKGGP
LEDTPTYPVSLSKRCSSCSQDYTKI
>ggOR39
LAAEFSLLTIMSYDRYVAICKPLHYGTLLGSRACATMAAAAWGTGVLNSLLHTANTFSLPLCQGNAVDQFFCEIPHILKLS
CSDAYLREIWLLGVSASLASGCFVFILFSYVQIFRAVLRMPSEQGRHKAFSMCLPHLAVLSLFLSTAFFANLRPPSLSSQS
LDLTMAVLYSVVPPTLNPLIYSMRNQELKDAVRKMISWTHFSRDKLLICLYK
>ggOR40
KPMAHPFWDTRPISYAGCAAQVFFFLFFLSAEYSILTIMSYDRYVPICKPLHYGTLLGSRACASMAAAAWGTGLLYSLLHT
ANTFSLPLCHGNVVDQFFCEIPHILKLSCSDAYLREVAFIVLGVLVFVGCFAFILFSYVHVFRAVLRMPSEQGQHKAFSMC
LPHLAVLSLLVSTATFANLKPPAFSSASMNLVVSFLYSVVPPALNPVIYSMRNQELRDTLGKLMSWTIFINNKLLISC
>ggOR41
MAAAAWGTGLLYSLLHTASTFSQALCRGKVVNQFFCEIPHILKLSCSESGSLRETGFIVLSVLAIYGCFAFILFSYVQIFR
AVLRMPSEQGRHKAFSTCLPHLAVVSLFVSTDFLAYLKPHSLVSPSLDLVVSFLYSVVTPTLNPLIYSMRNQELKDAVRKI
MSWTHFSSNKLLICLYK
>ggOR42
FSFSFRQIIPFLPMMSYDRYVAICKPLHYGTLLGSRACATMAAAACGTGLLYAVLHTANTFSLPLCQGNAVDQFFCEIPHI
LKLSCSQSYLKEIGLIALSVLVFFGSFVFIVVSYVQIFRAVLRMPSEQGRHKAFSMCLPHLVVLSLFVSTAMFAYLKPHSI
SSPPVDLVVAVLYAVVPPVMNPLIYSMRNKELKVAMSELMNKCVSKAMNCSYFSVCHL
>ggOR43
AWGTGLLYSLLHTANTFSLPLCQGNAVDHFFCEIPQILKLSCSESEFLRELGLLSTSAFIGVGCFVFIVVSYMQIFRAVLR
MQSEQGRHKAFSTCLPHLAVLSLLISSGFFAYLKPYSISSPSLDLVVSFLYSVVPPAVNPLIYSMRNQELKDALWKLMTKY
IWKAMNCPSLSLHVL
>ggOR44
MPNSSSISEFLLLALADTRQLQLLHFWLLLGIYLAALLGNGLISTAVACDHRLHTPMYFFLLNLALLDLGCISTTLPKAMA
NALWDNRAISYAGCAAQVFFFLFFISAEYSLLTIMSFDRYVAICKPLHYGTLLGSRACATMAAAAWGTGLLYSLLHTANTF
SLPLCQGNAVDQFFCEIPQILKLSCSKSYLKEIWLPGVXXXXXXXXXXXXXXXXXXXXXXXXXXXXXXXXXXXXXXXXXXX
XXXXXXXXXXXXXXXXXXXXXXXXXXXXXXXXXXXXXXXXXXFSGSKWEISIFPKLH
>ggOR45
MSYDRYVAICKPLHYGTLLGSRACATMAAAAWGTGVLNSLLHTAKTFSLPLCQGNAVDQFFCEIPHILKLSCSXXXXXXXX
XXXXXXXXXXXXXXXXXXXDSISTAVACDHRLHTPMYFFLLNLALLDLGCISTTLPKDMANALWHTRHISYAGCAAQVFFF
VFFLSAEYSLLTIMSYDRYVAICKPLHYGTLLGSRACATMAAAAWVSGLLYSLLHTASIFSLPLCRGNAVDQFFCEIPQNL
KLSCSGSEFLRELGLLSTSAFIGIGCFVFIVVSYVQIFRAVLRMPSEQGRHKAFSTCLPHLVVLSLFVSTAMFAYLKPHSI
SSPPVDLVVAVLYAVVPPVMNPLIYSMRNKELKVALSELLNKCVSKAMNCSYFSVRHL
>ggOR46
YLAALLGNGLISTAVACDHRLHTPMYFFLLNLALLDLGCISTTLPKAMANALWHTRHISYAGCAAQVFFFLFFISAEYCIL
TIMSYDRYVAICKPLHYGTLLGSRACATMAAAAWGSGLLYSLLHTANTFSLPLCQGNAVDQFFCEIPQILKLSCSDSNLRE
IWLLGVSASLVSGCFAFILFSYVQILRAVLRMSSEHGRHKAFSTCLPHLAVVFLFLSTGMVAYLKPPSETSSFLDLLVSLL
YSVMPPTLNPLIYSLRNKELMCSVRKVIS
>ggOR47
MPNSSSISKFLLLALADTRQLQLLHFWLLLGIYLAALLGNGLISTAVACDHRLHTPMYFFLLNLALLDLGCISTTLPKAMA
NALWHTRHISYAGCATQFFFFLFLFGAEYSILTIMSYDRYVAICK
>ggOR48
MPNSSSISEFLLLALADTRQLQLLHFWLLLGIYLAALLGNGLISTAVACDHRLHTPMYFFLLNLALLDLGCISTTLPKAMA
NALWHTRHISYTGCAAQLFFYLFLFSAEYCILTIMSYDRYVAICTLRHYGTLLGSRACAT
>ggOR49
MPNSSSISEFLLLALADTRQLQLLHFWLLLGIYLAALLGNGLISTAVACDHRLHTPMYFFLLNLALLDLGCISTTLPKAMA
NALWHTRDISYAGCAAQVFFFIFFISAEYSLLTIMSYDRYVAICKTVHYGTLLGSRASSTMQQLPGALSSQ
>ggOR50
MPNRSSISEFLLLALADTRQLQLLHFWLLLGIYLAALLGNGLISTAVACDHRLHTPMYFFLLNLALLDLGCISTTLPKAMA
NALWHTRAISYAGCAAQVFFFSFLFSAEYCILTVMSYDRYVAICKPLHYGTLLGSRACATMAAAAWGTGFFNSLLHTASTF
SLPLCQGNAVDQFFCEIPHILKLSCSKSFKSYLREVGFLLFSV
>ggOR51
MPNISSISEFLFLALADTRQLQLLHFWLLLGIYLAALLGNGLISTAVACDHRLHTPMYFFLLNLALLDLGCISTTLPKAMA
NALWHTRAISYAGCAAQTFLFVFLISAEYSLLTMMSYDRYIAICKPLHYGTLLGSRACATMAAAKGGTGVLNALLHTANRF

SLPLCQGNA
>ggOR52
MSNSSSISEFLLLALADTRQLQLLHFWLLLGIYLAALLGNGLISTAVACDHRLHTPMYFFLLNLALLDLGCISTTLPKAMA
NALWHTRHISYAGCAAQIFFLLFFFGAEYSILTMMSYDRYVAICKPLHYGTLMDSRACSTMAAAAWGAGLLYSLLHTATTF
SMCLCHGNAVDQFFCEIAQILKLSCSKS
>ggOR53
MPNSSSISEFLLLALADTRQLQLLHFWLLLGIYLAALLGNGLISTAVACDHRLHTPMYFFLLNLALLDLGCISTTLPKAMA
NIVFDTQAIFYAGCVAKVFLFVFFISAEYSLLTIMSYDRYVAICMPLHYRTLLGSRACATMTAAAWGAGLLYSLKHTANTF
SMPLCQG
>ggOR54
MPNSSSISEFLLLALADTRQLQLLHFWLLLGIYLAALLSNGLISTAVACDHRLHTPMDFFLLNLALLDLGCISTTLPKAMA
NALWHTRAISYAGCAAQVFFLLFLFSAETFLLTIMSYDRYVAICKPLHYGTLMNSRTCATMAAAAWGTGV
>ggOR55
MPNSSSISEFLLLALADTRQLQLLHFWLLLGIYLAALLGNGLISTALACDHRLHTPMYFFLLNLALLDLGCISTTLPKAMA
NALWHTRAISYAGCAAQVFFFAFFISAEYSILTVMSYDRYVAICKPLHYRTLLGSRACATMAAAAWGTGILHSLLHTASTF
SL
>ggOR56
MPNSSSISEFLLLALADTRQLQLLHFWLLLGIYLAALLGNGLISTAVACDHRLHTPMYFFLLNLALLDLACISTTLPKDMA
NALWHTRHISYSGCAAQVFFFLFFISAEYFLLTVMSYDCYIAICKPLHYRTLLGSRACATMAAAAWGTGVLNSQLHTANTF
SLPLCQGNAVDQFFCEIPHILKL
>ggOR57
MPNSSSISEFLLLALADTRQLQLLHFWLLLGIYLAALLGNGLISTAVACDHRLHTPMYFFLLNLALLDLGCISTTLPKAMA
NALWH
>ggOR58
MPNSSSISEFLLLALADTRQLQLLHFWLLLGIYLAALLGNGLISTAVACDHRLHTPMYFFLLNLALLDLGCISTTLPKAMA
NALWHTRHISYAGCAAQVFLFAFLQSAEYSLLTIMSYDRYVAICKPLHYGTLLGNRACATMAAGAWGTGVLASVLHTANES
TT
>ggOR59
MPNSSSISEFLLLALADTRQLQLLHFWLLLGIYLAALLGNGLISTAVACDHRLHTPMYFFLLNLALLDLGCISTTLPKAMA
NALWHTRAISYAGCATQLFFFLFLFGAEYSLLTIMSYDRYVAICMPLHYGTLLGSRACATMAAAAWGTGLLYSLLHTASRI
PPQGENQ
>ggOR60
MPNSSSISEFLLLALADTRQLQLLHFWLLLGIYLAALLGNGLISTAVACDHRLHTPMYFFLLNLALLDLGCISTTLPKAMA
NALWGTRAISYAGCAAQVFFFVFFLSAEYSLLTMMSYDRYVAICKPLHYGTLMDSRACATMAAAAWGAGLINSLLHTANTF
SL
>ggOR61
MPNSSSISEFLLLALADTRQLQLLHFWLLLGIYLAALLGNGLISTAVACDHRLHTPMYFFLLNLALLDLACISTTLPKAMA
NALWHTRHISYAGCAAQVFFFFFLFGAEYCILTIMSYDRYVAICKPLHYGTLLGSRACATMAAAAWGAGVLNSLLHTASTF
SLPLCQGNAVHQF
>ggOR62
MGQGNETVVPEFILEGFSGLNQRLQLFLSLVFLLIYLTTVMGNATIIFLVCVDNRLQIPMYFFIGNLAFLEICFTSCTSIK
LLVILSSGRRTISVSACFVQSYFYFALGCTELILLVVMSFDRYLAICQPLRYAAIMTQQLCILLLVAAWVAGFTFMSYHLV
LLSKLTFCGSNKIQHFFCDNSPLFQLSCSDT
>ggOR63
MPNSSSISEFLLLALADTRQLQLLHFWLLLGIYLAALLGNGLISTAVACDHRLHTPMYFFLLNLALLDLGCISTTLPKAMA
NALWHTRDISYTGCAAQVFFFAFFIDAEYCILTIMSYDRYVAICKPLHYGTLLGSRACATMAAAACGTGVLYSVLHTANTF
SLPLCQGNAVNQFFCEIP
>ggOR64
MSNSSSISEFLLLALADTRQLQLLHFWLLLGIYLAALLGNGLISTAVACDHRLHTPMYFFLLNLALLDLGCISTTLPKAMA
NALWGTRAISYAGCAAQVFLFVFLLSAEYSLLTIMSYDRYVAICKPLHYGTLLGSRACATMAAAAWGTGVLYSMLHTANTF
SLPLCQGNAVDQFFCEIPQILKLFCSESYL
>ggOR65
MPNSSSISEFLLLALADTRQLQLLHFWLLLGIYLAALLGNGLISTAVACDHRLHTPMYFFLLNLALLDLGCISTTLPKAMA
NALWHTRAISYAGCAAQVFFFVFFLSAEYSLLTVMSYDRYVAICKPLHYGTLLGSRACATMAAAAWGTGLLNSLLHTANTF
SLPLCQGNAVDQFFCEIPQILKLSCSEAYLREIWLLGGLLGEYLRVCL
>ggOR66
MPNSSSISEFLLLALADTRQLQLLHFWLLLGIYLAALLGNGLISTAVACDHRLHTPMYFFLLNLALLDLGCISTTLPKAMA
NALWHTRHISYAGCAAQVFFFLFFISAEYCILTIMSYDRYVAICKPLHYGTLLGSRACATMAAAAWGTGLLYSLLHTANTF
SLPLCQGNAVDQFFCEIPQILKLACS
>ggOR67
MPNSSSISEFLLLALADTRQLQLLHFWLLLGIYLAALLGNGLISTAVACDHRLHTPMYFFLLNLALLDLGCISTTLPKAMA
NALWHTRHISYAGCAAQVFFYDFFISAEYSILTIMSYDRYVAICKPLHYGTM
>ggOR68
MPNSSSISEFLLLALADTRQLQLLRFWLLLGIYLAALLGNGLISKAVACDHRLHTPMYFFLLNLALLDLGCISITLPKAMA
NALWHTRAISYAGCAAQVFFFVFFLSAEYSILTVMSYDHYVAICKPLHYGTLLGSRACATMAAAAWGTGVLTSVLHTANTF
SLPLCQGNAVDQFFCEIPQILNLSCSKSFKSYLREVGLLLFIVCLSFGCFVFIVVSYVQIFRAVLRMPSDQGWHKAFSTCL
PHLAVVSLFLNTGMVAYLKPSSISSP
>ggOR69

MPNSSSISEFLLLALADTRQLQLLHFWLLLGIYLAALLGNGLISTAVACDHRLHTPMYFFLLNLALLDLGCISTTLPKAMA
NALWHTRHISYAGCATQVFFLVFFISAEYSLLTVMAYDRYVAICKPLHYRTLLGSRACATMAAAAWGTGVLNSLLHTANTF
SLPLCQGNAVDHFFCEIPQILKLS
>ggOR70
MPNSSSISEFLLLALADTRQLQLLHFWLLLGIYLAALLGNGLISTAVACDHRLHTPMYFFLLNLALLDLGCISTTLPKAMA
NALWGTRDISYAGCAAQVFFLVFFISAEYSLLTVMSYDRYVAICKPLHYGALLGSRACATRAERAWGSGLLHSLLHT
>ggOR71
MPNSSSISEFLLLALADTRQLQLLHFWLLLGIYLAALLGNGLISTAVACDHRLHTPMYFFLLNLALLDLGCISTTLPKAMA
NALWHTRAISYAGCAAQIVFFVFFLSAEYCILTIMSYDRYVAICKPLHYGTLLGSRACATMAAAAWGTGLLNAVLQTANTF
SLPLCQGNAVDQFFCEIPQIIKLTCS
>ggOR72
MPNSSSISEFLLLALADTRPLQLLHFWLLLGIYLAALLGNGLISTAVACDHRLHTPMYFFLLNLALLDLGCISTTLPKAMA
NALWHTRAISYAGCAAQVFFFFFFLSAEYSLLTIMSYDRYIAICKPLHYGTLLGSRACATMAAAAWVTGVLNSLLHTANTF
SLLFCQGNAVHQFFCEIPHILKLSCSDAYLREIWLLGVSATLSSGCFVFILF
>ggOR73
MPNSSSISEFLLLALADTRQLQLLHFWLLLGIYLAALLGNGLISTAVACDHRLHTPMYFFLLNLALLDLGCISTTLPKAMA
NALWHTRHISYAGCATQVFFFLFLLSAEHSLLTVMSYDRYIAICKPLHYGTLLDNRACATMAAAAWGTGLLHAVLHTANTF
SQALCRGKAVNQFFCEIPHILKLSCSESDYLRETGFIVVTVLALFGCFVFILFSYVQIFRARA
>ggOR74
MPNSSSISEFLLLALADTRQLQLLHFWLLLGIYLAALLGNGLISTAVACDHRLHTPMYFFLLNLALLDLGCISTTLPKAMA
NALWHTRHISYAGCVAQVFFFLFVFSAEYSILTIMSYDRYVAICKPLHYGTLMDSRACATMAAAAWGTGLLYSLLHTASTF
SQALCR
>ggOR75
MPNSSSISEFLLLALADTRQLQLLHFWLLLGIYLAALLGNGLISTAVACDHRLHTPMYFFLLNLALLDLGCISTTLPKAMA
NALWHTRHISYAGCAAQVFFFVFFISAEYCILTIMSYDRYVAICKPLHYGTLLGSRACATMAAAAWGTGLLYSLLHTANTF
SLPLCQGNAVDQFFCEIPQILKLSCS
>ggOR76
MANSSSISEFLLLALADTRQLQLLHFWLLLGIYLAALLGNGLISTAVACDHRLHTPMYFFLLNLALLDLGCISTTLPKAMA
NALWHTRDISYAGCAAQVFFFLFFISAEYSILTIMSYDRYVAICKPLHYGTLLGSRACATMAAAAWGIGLLCSLLHTASTF
SLPFCQGNAVDQFFCEIPQILRLSCSESDNRRETGFIALSVLAVFGCFVFILFSYVQIFRAVLRMPSEQGRHKPF
>ggOR77
MPNSSSISEFLLLALADTRQLQLLHFWLLLGIYLAALLGNGLISTAVACDHRLHTPMYFFLLNLALLDLGCISTTLPKAMA
NALWDNRAISYAGCAAQVFFFLFFISAEYSLLTIMSFDRYVAICKPLHYGTLLGSRACATMAAAAWGTGLLYSLLHTANTF
SLPLCQGNAVDQFFCEIPQILKLSCSDAYLREIWLLGVSATLSSGCFVFVLFSYV
>ggOR78
MPNSSSISEFLLLALADTRQLQLLHFWLLLGIYLAALLGNGLISTAVACDHRLHTPMYFFLLNLALLDLGCISTTLPKAMA
NALWHTRHISYAGCAAQVFFFLFLFSAEYYLLTVMAYDRYVAICKPLHYETLISYRTCATMATAAWGTGLLYSLLHTASVF
SLPLCQGNAVDQFFCEIPHILKLSCSQSYLREAG
>ggOR79
MPNSSSISEFLLLALADTRQLQLLHFWLLLGIYLAALLGNGLISTAVACDHRLHTPMYFFLLNLALLDLGCISTTLPKDMA
NALWHTRHISYAGCAAQVFFFVFFLSAEYSLLTIMSYDRYVAICKPLHYGTLLGSRACATMAAAAWVSGLLYSLLHTASIF
SLPLCRGNAVDQFFCEIPQNLKLSCSGSEFLRELGLLSTSAFIGIGCFVFIVVSYVQIFRAVLRMPSEQGRHKAFSTCLPH
LVVLSLFVSTAMLEST
>ggOR80
MPNSSSISEFLLLALADTRQLQLLHFWLLLGIYLAALLGNGLISTAVACDHHLHTPMYFFLLNLALLDLGCISTTLPKAMA
NALWHTRHISYAGCAAQVVFFLFFISAEYSILTIMSYDRYVAICKPLHYGTLLGSRACATMAAAAWGTGLLYSLLHTANTF
SLPLCQGNAVDQFFCEIPHILKLSCSESYLREVGLLLFSVCLACGCFVFIVVSYVQIF
>ggOR81
MPNSSSISEFLLLALADTRQLQLLHFWLLLGIYLAALLGNGLISTAVACDHRLHTPMYFFLLNLALLDLGCISTTLPKAMA
NALWHTRAISYAGCAAQVFFLLFLFGAEYSFLIVMSYDRYVAICKPLHYGTLLGSRACATMAAAAWGTGLLCSLLHTANTF
SLPLCQGNVVNQFFCEIPQILKLTCSKSYLSEFGLIMLSALIFFGCFAFILFSYVQIFRAVLRMPSEQGRHKAFSTCLPHL
AVLSLFLSTVMFAYLKPFSISSQSLD
>ggOR82
MPNSSSISEFLLLALADTRQLQLLHFWLLLGIYLAALLGNGLISTAVACDHRLHTPMYFFLLNLALLDLGCISTTLPKAMA
NARWHTRDISYAGCAAQVFFFVFFLSAEYSLFTIYDRYVAICKPLHYGTLLGSRACASMAAAVWGTGLLYSLLHTANTFSL
PLCQGNAVDQFFCEIPHILKLSCSQSYLREAGLLMFSALFFFASFVFIVVSYVQIFRAVLRMPSEQ
>ggOR83
MLNSSSISEFLLLALADTRQLQLLHFWLLLGIYLAALLGNGLISTAVACDHRLHTHMYFFLLNLALLDLGCISTTLPKAMA
NALWHTRDISYAGCAAQVFFFVFFISAEYFLLTVMSYDRYVAICKPLHYGTLLGSRACATMAAAAWGTGLLYSLLHTATAF
SVPLCQGNLVDQFFCEIAQILKLSCSRSDYLREVGIIYFILCLICGCFVFIVVS
>ggOR84
MPNSSSISEFLLLALADTRQLQLLHFWLLLGIYLAALLGNGLISTAVACDHRLHTPMYFFLLNLALLDLGCISTTLPKAMA
NALWHTRHISYAGCAAQVFFFLFLYSTEYYLLTLMAYDCYVAICKPLHYETLLSFRSCATMAAAVWGSGLLFSLLHTSNTF
SLPLCQGNAVDQFFCEIPHILKLSCSDAYLREVGLIVAFFHVLFGCFVFIVVSYVQIFRAVLRMPSEQGRHKA
>ggOR85
MPNSSSISEFLLLALADTRQLQLLHFWLLLGIYLAALLGNGLISTAVACDHHLHTPMYFFLLNLALLDLGCISTTLPKAMA
NAFWDTRDISYTGCAAQVFFFLFFLAAEYSILTIMSYDRYVAICKPLHYGTLLGSRACATMAAAAWGTGVIYSLLHTANTF
SLPLCQGNAVDQFFCEIPHILKLSCSDAYLREVGLIVLSVPMEFGCFVFIGVSYVPIFRAV

>ggOR86
MPNSSSISEFLLLALADTRQLQLLHFWLLLGIYLAALLGNGLISTAVACDHRLHTPMYFFLLNLALLDLGCISTTLPKAMA
NALWHTRHISYAGCAAQVFFLLFLFGAEYSLLIVMSYDRYVAICKPLHYGTLLGSRACATKAAAAWGTGLLCSLLHTANTF
SLPLCQGNVVNQFFCEIPQILKLACSKSYLSEFGLIMLSALIFFGCFAFILFSYVQIFRAVLRIPSEQGRHKAFSTCLPQR
VVLSLFLSTVMLTDLKPFSISSPSLDES
>ggOR87
MPNSSSISEFLLLALADTRQLQLLHFWLLLGIYLAALLGNGLISTAVACDHRLHTPMYFFLLNLALLDLGCISTTLPKAMA
NALWHTRHISYAGCATQVFFLVFFISAEYSLLTVMAYDRYVAICKPLHYRTLLGSRACATMAAAAWGTGVLNSLLHTANTF
SLPLCQGNAVDQFFCEIPQILKLSCSHSYLKDIWLLIVSASLASGCFVFIVVSYVQIFRAVLRMPSEQGRHKALSGCLPHL
AVLSLFLRAGIFAYQKPPPSPPH
>ggOR88
MPNSSSISEFLLLALADTRQLQLLHFWLLLGIYLAALLGNGLISTAVACDHRLHTPMYFFLLNLALLDLGCISTTLPKAMA
NALWHTRAISYAGCAAQVFLFVFLMSTEYSVLTVMSYDRYVAICKPLHYGTLLGSRACATMAVAAWGTGVLYSLLHTANTF
SLPLCQGNAVDQFFCEIPQILKLSCSDAYLREAGLIAFSVAMEFGCFIFIVVSYVQIFRAVLRMPSEQGRHKAFSTCLPHL
AVLSLF
>ggOR89
MPNSSSISEFLLLALADTRQLQLLHFWLLLGIYLAALLGNGLISTAVACDHRLHTPMYFFLLNLALLDLGCISTTLPKAMA
NALWHTRHISYAGCATQFFFFLFLFGAEYSILTIMSYDRYVAICKPLHYGTLLGSRACATMAAAAWGTGLLYSLLHTASTF
SQALCRGKVVNQFFCEIPHILKLSCSESGSLRETGFIVLSVLAIYGCFAFILFSYVQIFRAVLRMPSEQGRHKAFSTCLPH
LAVVSLFVSTDGLAYLKPHSLVSQWLDLVVSFLYSVVTNS
>ggOR90
MPNSSSISEFLLLALADTRQLQLLHFWLLLGIYLAALLSNGLISTAVACDHRLHTPMYFFLLNLALLDLGCISTTLPKAMA
NALWDTRAISYAGCAAQVFFSLLFISSEYSLLTIMSYDRYVAICKPLHYGTMLGGTACATMAAAAWGTGLLFSLLHTANTF
SLPLCQGNAVDQFFCEIPQILKLSCSESDYFREAGLIMADLQGRAEDAF
>ggOR91
MPNSSSISEFLLLALADTRQLQLLHFWLLLGIYLAALLGNGLISTAVACDHRLHTPMYFFLLNLALLDLGCISTTLPKAMA
NALWHTRAISYAGCAAQVFFFLFFLSAEYSILTMMSYDRYVAICKPLHYGTLLGSRACATMAAAACGTGLLYAVLHTANTF
SLPLCQGNAVDQFFCEIPHILKLSCSQSYLKEIGLIALSVLVFFGSFVFIVVSYVQIFRAVLRMPSEQGRHKAFSMVDGLN
GL
>ggOR92
MANSSSISEFLLLALADTRQLQLLHFWLLLGIYLAALLGNGLISTAVACDHRLHTPILHTPMYFFLLNLYLLDLGCISTTL
PKAMANALWHTRHISYAGCAAQVFLFAFLISAEYSLLTIMSYDRYVAICKPLHYGTLLGSRACATMAAAAWGTGVLTSVLH
TANTFSLPLCQGNAVDQFFCEIPQILKLSCSQSYLREVGLIFFSVLVLFGCFVFIFFSYVQIFRALLRMPSEQGRHKAFST
CLPHLAVLSLFLSTVVFAHLKPFSISSPSLDLVVSFLYTVVPPTLNPIIYSMRNKDIRHALSEMFQYTLFQHQ
>ggOR93
GTLLGSRACATMAAAAWGSGLLNALLHTANTFSLPLCQGNAVDQFFCEIPHILKLSCSDAYLREVGLIVFSALALLGCFVF
ILFSYVQIFRAVLRMPSVQGRHKAFSTCLPHLAVLSLLVCSAFFAYLKPPSISSPFLDQLVSFLYSVVPPALNPLIYSIRN
QELKDALWKLMTKRLLKAMNCPSLSI
>ggOR94
MPNSSSISEFLLLALADTRQLQLLHFWLLLGIYLAALLGNGLISTAVACDHRLHTPMYFFLLNLALLDLGCISTTLPKAMA
NALWHTRDISYAGCAAQVFFLLFFFGAEYSLLTIMSYDCYVAICKPLHYGTLLGSRACATMAAAAWRSGLLYSLLHTANTF
SLPLCQGNAVDQFFCEIPQILKLSCSESYLREVGLLLFSVCLSFGCFVLIVVSYVQIFRAVLRMPSEQGRHKAFSTCLPHL
AVVSLFVSTVVFAHLKPFSISLPSLDLKALLINPASLLPRIFVAILVSQFLSAISIPLFSRRCPKP
>ggOR95
MANSSSISEFLLLALADTRQLQLLHFWLLLGIYLAALLGNGLISTAVACDHRLHTPMYFFLLNLALLDLGCISTTLPKAMA
NALWGTRAISYAGCAAQVFFFLFLFGAEYCILTIMSYDRYAAIYKPLHYGTLLDSRACATMAAAAWGTGVLNSLLHTASTF
SLPLCQGNAVDQFFCEIPHILKLSCSESYLREVGLLLFSVCLASVFFVFLLCPMC
>ggOR96
MPNSSSISEFLLLALADTRQLQLLHFWLLLGIYLAALLGNGLISTAVACDHRLHTPMYFFLLNLALLDLGCISTTLPKAMA
NALWHTRHISYAGCAAQVFFLLFFFGAEYSLLTIMSYDRYVAICKPLHYGTLLGSRACATMAAAAWGTGLLYSLLHTANTF
SIAFCLSNAVDQFFCEMAQILKLSCSESDYLRATRIIYFSLCLIFGCFVFIVLSYVQIFRAVLRMPSEQGQHKTFSTCLPH
LAVVSLFVSTVMFSYLKPPSISSRSLDLLVSSVYSLLPPTLNPLIYSMRNQELKDTLRKLRIISGCIR
>ggOR97
MPNSSSISEFLLLALADTRQLQLLHFWLLLGIYLAALLGNGLISTAVACNHRLHTPMYFFLLNLALLDLGCISTTLPKAMA
NALWHTRNISYAGCAAQVFLFVFFLSAECCILTIMSYDRYVAICKPLHYGTLLGSRACATMAAAAWGIGFFNSLLHTANTF
SLPLCQGNAVDQFFCEIPQVLKLSCLNSYLREVGLLLFSACVVFGCFVFIVVSYVQIFRAVLRMPSEQGRHKAFSTCLPHL
AVLSLFLSSAFFAYLKLFSIFSPSLDLTVALLYSVVPPALNPIIYSMRNKEIKHALSKVLQYALFQHQESAHLPHMCPRTV
>ggOR98
MPYSSSISEFLLLALADTWQLQLLHFWLLLGIYLAALLGNGLISTAVACKHRLHTPMYFFLLNLSLLDLGCISTNLPKAMA
NALWGIRAISYAGCAAQLFFFVFFISAEYSLLTIMSYDRYIAICKPLHYGTLLGSRACATMAAAAWGTGVLYSLLHTANTF
SLPLCQGNAVDQFFCEIPHILKISCSESDYLRETGIIAFSFSFILGCFAFILFSYVQIFRAVLRMLSEQGRHKAFSTCLPH
LAVLSLFLSTVMFAYMKPPSISSRSLDLVVSFLYSVVPPTANPCIYSMRNQELKDAVKTLRRIPWCIRKF
>ggOR99
MPNSSSISEFLLLALADTRQLQLLHFWLLLGIYLAALLGNGLISTAVACDHRLHSPMYFFLLNLALLDLGCISTTLPKAMA
NALWHTRAISYAGCAAQVFFFVFFISAEYCILTIMSYDRYVAICKPLHYGTLLGSRACATMAAAAWGTVLLYSLLHTANTF
SLPLCQGNAVDQFFCEIPQILKLSCSESYLREVGLLLFSVCACLGCFVFIVVSYVQIFRAVLRMPSEQGQHKTFSTCLPHL
AVVSLFVSTGFFAYVKPPSISSPFIDLTVALLYSVVPPTLNPIIYSMRNKEIKHALRKLLQYVHYSTFNKVPIFLT
>ggOR100

MSNSSSNSEFLLLAFGERWELRLLHFWLFLGIYLAALLGNGLIIIGIACDHRLHTPMYFFLLNLALLDLGGISTTVPKSMA
NSLLDSRSISYSGCVAQVFFFVFLAGAEFSLLMVMSYDRYIAICKPLHYGTLLSSRTCATMAAAAWGCGFLSAILHTANTF
SLPLCQSNAVNQFFCEIPQILKLSHSHSFLREIWATVAVVSLASLFFVFILFSYVWIFRAVLRMPCEQGRHKAFSTCLPHL
AVVSLFLSTAFFAYLKPPSISSPISDLVVAVLYSVLPPTLNPLIYSMRNMELKVAVRKVISQMFLNICKFTLSCSYL
>ggOR101
MYFFLLTLAFFDLGCFSTTLPKAMANALWHTRDISYAGCAAQVFFLLFFFSAETFLLTIMSYDRYVAICKPLHYGTLMDSR
ACATMAAAAWATGVLYSLMHTVNTFSIPLCQGNVVNQFFCEMAQVLKLSCSESDYLIEAGIIDFSLSLIFGCFVFIVVSYV
QIFRAVLRMPSEQGRHKAFSICLPHMSVVSLFVSTLMFAYLKPPSISSPSLDLVVAVLYSVVPPTVNPLIYSMRNQELRDA
LRKLWEIPSNIRKY
>ggOR102
MANSSSISEFLLLALADTRQLQLLHFWLLLGIYLAALLGNGLISTAVACDHRLHTPMYFFLLNLALLDLGCISTTLPKAMA
NALWHTRHISYAGCAAQVFFFLFFISAEYSLLTIMSYDRYVAICKPLHYGTLLGSRACATMAAAAWGTGLLYSLLHTANTF
SLPLCQGNAVNQFFCEIPQILKLSCSKSYLREFGLIVTSALVFFGCFAFILFSYVQIFRAVLRMPSEQALDNLYELRHRTV
GITEKILLGYQCHLSKTFNLQAEHGNFKMT
>ggOR103
MSYDRYVAICKPLHYGTLLGSRACATMAAAAWGTGVLNSLLHTANTFSLPLCQGNAVDQFFCEIPHILKLSCSDAYLREIW
LLGVSASLASGCFVFILFSYVQIFRAVLRMPSEQGRHKAFSMCLPHLAVLSLFLSTAFFANLRPPSLSSQSLDLTMAVLYS
VVPPTLNPLIYSMRNQELKDAIKAYIHIIQAAN
>ggOR104
MPNSSSISEFLLLALADTRQLQLLHFWLLLGIYLAALLGNGLISTAVACDHRLHTPMYFFLLNLALLDLGCISTTLPKAMA
NALWHTRHISYTGCAAQVFFFVFFLSAEYCLLTMMSYDRYVAICKPLHYGTLLGSRACATMAAAAWGNGILNSLLHTANTF
SLPLCQGNAVDQFFCEIPQILKLSCSESYLSEFGLIMLSALIFFGCFAFILFSYVQILRAVLRMPSEQGRHKAFSTCLPHL
AVVSLFLSTVVFAYLKPFSISSPFLDLTVALLYSVIPPTLNPIIYSMRNKEIKHALSKVLQCARFQLQ
>ggOR105
MPNSSSISEFLLLALADTRQLQLLHFWLLLGIYLAALLGNGLISTAVACDHRLHTPMYFFLLNLALLDLGCISTTLPKAMA
NALKHTRHISYAGCAAQVFLFVFLLSAEYSILTIMSYDRYVAICKPLHYGTLLGSRACATMAAAAWSTGVLNALLHNANTF
SLPLCQGNAVDHFFCEIP
>ggOR106
MPNSSSISEFLLLALADTRQLQLLHFWLLLGIYLAALLGNGLISTAVACKYRLHTPMYFFLLNLSLLDLACISTTLPKAMA
NALWHTRDIYAGCAAQHFFFLFLFGAEYSLLTIMSYDRYIAICKPLHYGTLLGSRACATMAAAAWGTGVPYSLMHTASTFS
QTQCPFKGVNQFFCEIPHILKLSCSESDFLRETGFIVVTVLAIFGCFAVILFPYVQIFRAVLRMPSEQGRHKAFSTCLPHL
VVVSLFVCTDFLAYLKPHSLVSPYLNLVVSFLYSVVPPTLNPLIYIMRNQELKDA
>ggOR107
MPNSSSISEFLLLALADTRQLQLLHFWLLLGIYLAALLGNGLISTAVACDHRLHTPMYFFLLNLALLDLGCISTTLPKAMA
NALWHTRAISYAGCAAQVFFLLFLFGAEYSFLIVMSYDRYVAICKPLHYGTLLGSRACATMAAAAWGTGALYALLHTATAF
SVPLCQGNAVDQFFCEIAQILKLSCSKSDYLREIVDIIVGKDEKGRKIPEYLIHFNGWNRSWDRWAAEDHVLRDTDENRRL
QRKLARKAVARMLNKLSSLNIYSQERCSSPLIILMTHSNSSASFLYWGPRPGCSTPDGAFQGQSIVGKSP
>ggOR108
MPNSSSISEFLLLALADTRQLQLLHFWLLLGIYLAALLGNGLISTAVACDHRLHTPMYFFLLNLALLDLGCISTTLPKAMA
NALWHTRHISYAGCAAQVFFFLFLYSTEYYLLTLMAYDCYVAICKPLHYETLRCCSTAYVAFKYQISTLHGDGKQLLPDSS
ASSRLGPL
>ggOR109
MANSSSISEFLLLALADTRQLQLLHFWLLLGIYLAALLGNGLISTAVACDHRLHTPMYFFLLNLALLDLGCISTTLPKAMA
NALWHTRHISYAGCAAQVFFLLFFFGAEYSLLTIMSYDRYVAICKPLHYGTLLSSRACATMAAAAWGTGLLYSLLHTANTF
SIAFCLSNAVDQFFCEMAQILKLSCSESDYLRATRIIYFSLCLIFGCFVFIVLSYVQIFRAVLRMPSEQGQHKTFSTCLPH
LAVVSLFVSTVMFSYLKPPSISSRSLDLLVSSVYSLLPPTLNPLIYSMRNQELKDTLRKLRIISGCIR
>ggOR110
MPNSSSISEFLLLALADTRQLQLLHFWLLLGIYLAALLGNGLISTAVACDHRLHTPMYFFLLNLALLDLACISTTLPKAMA
NALWGTRAITYAGCAAQVFFFVFFLSAEYSLLTMMSYDRYVAICKPLHYGTLLGSRACATMAAAAWGIGFFNSLLHTASTF
SLPLCQGNAVDQFFCEIPHILKLSCSKSFKSYLRNPPQG
>ggOR111
MPNSSSISEFLLLALADTRQLQLLHFWLLLGIYLAALLGNGLISTAVACDHRLHTPMYFFLLNLALLDLGCISTTLPKAMA
NALWHNRAISYAGCATQVFFFVFFISSDYSLLTMMSYDCYVAICKPLHYGTLLGSRACAIMAAAAWGTGLFHALLHTANTF
SLPLCQGNAVDQFFCEIPQILKLSCSDAYLREAGLIVVSVLLELGCFVFIVVSYVQIFRAVLRIPSEQGRHKAFSTCLPHL
AVLSLLVSSAFFAYLKPSSISSPSLDLVVSFLYSVVPPAVNPLIYSMRNQELKDALWKLMTKYIWKAMNCPSLSLHVL
>ggOR112
MSYDRYVAICKPLHYGTLLGSRACATMAAAAWGTGVLNSLLHTASTFSLPLCKGNAVDQFFCEIPQILKLSCSESYLREVG
LLLFSVCLASVFFVFIVVSYVQIFRAVLRMPSKQGRHKAFSTCLPHLAVVSLFLSTVMFTYLKPFSFSSPFLDVAAALLYS
VIPPTLNPIIYSMRNKEITHALSKVLQYALFRIQ
>ggOR113
MPNISSISEFLFLALADTRQLQLLHFWLLLGIYLAALLGNGLISTAVACDHRLHTPMYFFLLNLALLDLGCISTTLPKAMA
NALWHTRAISYAGCAAQTFLFVFLISAEYFLLTMMSYDRYIAICKPLHYGTLLGSRACATMAAAAWGTGVLNALLHTASTF
SLPLCQGNAVDQFFCEIPHILKLSCSQSYLREVGLIVFSVLVLFGCFAFILFSYVQIFRAVLRMPSEQGRHKAFSTCLPHL
AVVSLFLSTGFFANLRPPSLSSPSLDLMMAFLYSVVPPTLNPLIYSMRNQELRHAVRKMVPWSHFSRDKLLVCLYK
>ggOR114
MSYDRYMPICKPLHYATLLDSRACATMAAAAWGTGVLNSLLHTANTYSLPLCQGNAVDQFFCEIPHILKLSCSDAYLREAG
LIVAGVIIFFGCFAFILFSYVQIFRAVLRMPSEQGRHKAFSTCLPHLAVVSLFVSTVVFAHLKPFSISLPSLDLVLALLYS
VVPPTLNPIIYSMRNQDIKHAISKVLQYALFQVQ

>ggOR115
MPNSSSISEFLLLALADTRQLQLLHFWLLLGIYLAALLGNGLISTAVACDHRLHTPMYFFLLNLALLDLGCISTTLPKAMA
NALWGTRAISYAGCAAQVFFFLFLFSTEYYLLTIMSYDRYVAICKPLHYRTLLGSRACATMAAAAWGSGILYSLMHTVSTF
SIPLCQGNVINQFFCEIPQILKLSCSESDYLSEAGIIDFSLSLIFGCFVFIVVSYVQIFRAVLRMPSEQGRHKAFSTCLPH
IFVVSLFVSTLLFACLKPHSLSSTSLDLVVAVLYSVVPPIVNPLIYSMRNQELRDALRKLWKIPSYIRKY
>ggOR116
MPNSSSISEFLLLALADTRQLQLLHFWLLLGIYLAALLGNGLISTAVACDHRLHTPMYFFLLNLALLDLGCISTTLPKAMA
NALWGTRAISYAGCAAQVFFFLFLFSTEYYLLTIMSYDRYVAICKPLHYRTLLGSRACATMAAAAWGSGILYSLMHTVSTF
SIPLCQGNVINQFFCEIPQILKLSCSESDYLSEAGIIDFSLSLIFGCFVFIVVSYVQIFRAVLRMPSEQGRHKAFSTCLPH
IFVVSLFVSTLLFACLKPHSLSSTSLDLVVAVLYSVVPPIVNPLIYSMRNQELRDALRKLWKIPSYIRKY
>ggOR117
MAAAAWGSGLLHSLLHTANTFSLPLCQGNAVDQFFCEIPQTLKLSCSKSYLREVGLTVINALLFFGCFAFILFSYVQIFRA
LLRIPSEQGRHKAFSTCLPHLAVVSLFVSTATFAYLKPPSLSSPSLDLVVAVLYTVVPLVMNPIIYSMRNQEIKQALTTLF
ADTLLRHH
>ggOR118
MPNSSSISEFLLLALADTRQLQLLHFWLFLGIYLAALLGNGLIIIGIACDHRLHTPMYFFLLNLALLDLGCISTTLPKAMA
NALWDTRDISYAGSEYFLLTVMSYDRYVAICKPLHYETLLGSRACASMAAAAWGCGVLNAVLQTANTFSLPFCRGNAVNHF
FCEILKLSCSNFFLRKSGFLLVSAMLTFGCFIFIVFSYVQIFRAVLRMPLKQGRHKAFSTCLPHLAVVSLFLTSDFLAYLK
PPSISSPPLDLAVSFLYSVLEAITPFITTMPSGRPAVAQQCTLRACHQPQDDAVETAELSPRHTRPQGRHKFKGTTALGTR
LQPRLHSLQPTWDTELSALCSVLQQEQVLLQNKRMHSASSLIR
>ggOR119
MPNSSSISEFLLLALADTRQLQLLHFWLLLGIYLAALLGNGLISTAVACDHRLHTPMYFFLLNLALLDLGCISTTLPKAMA
NALCGTRAISYTGCAAQVFFLLFLFGAEYSILTIMSYDRYVAICMPLHYGTLLGSRACATMAAAAWGTGVLYSLMHTANTF
SLPLCQGNAVDQFFCEIPQILKLSCSEYYLREVGLLLFSVCLAFGCFVFIVVSYMQIFRAVLSIPSEQGRHKAFSTCLPHL
AVVCLFLSTGFFAYLKPPSISSPSLDLVVALLYSMVPPTLNPIIYSMRNKEIKHALSKLLQYAVFLLQ
>ggOR120
MPNSSSISEFLLLALADTRQLQLLHFWLLLGIYLAALLGNGLISTAVACDHRLHTPMYFFLLNLALLDLGCISTTLPKAMA
NALWHTRHISYAGCAAQVFFLLFLFGAEYSLLTIMSYDRYVAICKPLHYGTLLGSRACATMAAAACGTGVLNSLLHTANTF
SLPLCQGNAVDQFFCEIPHILKLACSEYYLREVGLNVISALVLFGCFAFILFSYVQIFRAVLRMPSEQGRHKAFSTCLPHL
AVLSLFLSTVVFAYLKPFSISSSFLDVVVAFLYSVVPPTLNPIIYSMRNQDIKHALSKVLQCALFQLQ
>ggOR121
MPNSSSISEFLLLALADTRQLQLLHFWLLLGIYLAALLGNGLISTAVACDHRLHTPMYFFLLNLALLDLGCISTTLPKAMA
NALWHTRAISYAGCAAQVFFFLFLLSAEYSLLTIMSYDRYIAICKPLHYGTLLGSRACATMAAAAWGTGLLHAVLHTANTF
SQALCRGKVVNQFFCEIPHILKLSCSESDYLRETGFIVLSVLAIFGCFVFILFSYVQIFRAVLRMPSEQGRHKAFSMCLPH
LAVVSLFVSTDFLAYLKPHSLVSPYLNLVVSFLYSVVPPTLNPLIYSMRNQEFKDAVRKIMSWTHFSTDKLLICLYK
>ggOR122
MPNSSSISEFLLLALADTRQLQLLHFWLLLGIYLAALLGNGLISTAVACDHRLHTPMYFFLLNLALLDLGCISTTLPKAMA
NALWRTRHISYTGCAAQVFFYLFLFSAEYCILTIMSYDRYVAICKPLHYGTLLGSRACATMAAAAWGSGLLYSLLHTANTF
SLPLCQGNAVNQFFCEIPQILKLSCSKSYLREVGLSVISALVLLGCFAFILFSYVQIFRAVLRMPSEQGRHKAFSTCLPHL
AVVSLFLSTVVFAHLKPFSISSPSLDLVVALLYSVVPPTLNPIIYSMRNPDIKLALRKVLQYALLQLQ
>ggOR123
MSYDRYVAICKPLHYGTLLGSRACATMAAAAWGTGLFHALLHSANTFSLPLCQSNAVDQFFCEIPHILKLSCSESYLREVG
LIVVSVLLELGCFVFIVVSYVQIFRAVLRIPSEQGRHKAFSMCLPHLAVLSLLVSSAFFAYLKPSSISSPSLDMVVSFLYS
VVPPTVNPLIYSMRNQELKDAVWKLMTSFILKTMNCPTLSIHDL
>ggOR124
MAAAVWGTGLLNAVLQTAYTFSLPLCQGNAVDQFFCEIPQILKLSCSKSYLSEVGLLLFILCLSFGCFVFIVVSYVQIFRA
VLGMPSEQRRHKAFSTCLPHLAVLSLSVSTGFFAYVKPHSQSSPFLDLAVALLYSVVPPTLNPIIYSMRNKDIKHALCKLL
QYVRYSSFNKVPIFLT
>ggOR125
VPPPGVGRRAAAAGLALLALAGHLPGCPPGQRLHQHSRSLRPPLAHPHVFFLLNLALLDLACISTTLPKAMANALWHTRHI
SYTGCAAQNFFFLFFLAAEYCILTIMSYDRYVAICKPLHYGTLLGSRACATMAAAAWGTGVLNSLLHTANTFSLPLCQGNA
VDQFFCEIPHILKLSCSKSYLREVGLTVVSALVLFGCSAFILFSYVQIFRAVLRIPCEQGRRKAFSTCLPHLAVLSLFVST
ATFAYLKSPSLSSQSLDLVVTVLYTVVPPVMNPIIYSIKNQEIKQAIRTVFKGKLLRHQ
>ggOR126
MPNSSSISEFLLLALADTRQLQLLHFWLLLGVYMAALLGNGLISTAVACDHRLHTPMYFFLLNLALLDLGCISTTLPKAMA
NALWHTRDISYAGCAAQLFFFLFFFSAEYYLITVMAYDRYVAICKPLHYRTLLSFRACATMAAAAWGTGLLYSLLHTANTF
SLPLCQGNAVDQFFCEIPQILKLSCSDAYLREAGLIMACILVLFGCFVFTVVSYVQIFRAVLKMPSEQGRHKAFSMCLPHL
AVVSLFASTAMFAYLKPPSVSFPSLDLVVSVLYSVVPPTLNPLIYGMRNQELK
>ggOR127
MSYDRYIAICKPLHYGTLLGSRACATMAAAAWGTGLLNSLLHTANTFSLPLCQGNAVDQFFCEIPQILKLSCLNSYLREVG
LLLYSVCLSFGCFVFIVVSYVQIFTAVLRMPSEQGRHKAFSTCLPHLAVLSLFLSTDFFAYLKPFSISSPSLDLTVALLYS
VVPPALNPLIYSMRNKEIKHALSKVLQYALFQHQ
>ggOR128
MSYDRYVAICKPLHYRTLLGSRACATMAAVVWGTGLLYSLLHTANTFSLPLCQGNAVDQFFCEIPQILKLSFSDGYLREAG
LIIAFVLAVFGCFAFILFSYVQIFRAVLRMLFEQGRHKAFSTCLPHLAVLSLLVSTAMFAYLKPPSISSPSLDLVVSFLYS
VMPPALNPLIYGMRNQELKGALRKILQHTLFRHQKYVHHDLKTTKNLEKA
>ggOR129
MPNSSSISEFLLLALADTRQLQLLHFWLLLGIYLAALLGNGLISTAVACDHRLHTPTYFFLLNLALLDLGCISTTLPKAMA

NALWHTRHISYAGCAAQVFFFFFFFSAEYYLLTVMAYDRYVAICKPLHYGTLLGSRACATMAAAAWGTGVLNSLLHTACVF
SLPLCQGNAVDQFFCEIPHILKLSCSDAYLREIWLLGVSASLSSGCFVFILFSYVQIFRAVLRMPSEQGRHKAFSTCLPHL
AVVTLFLSTAFFANLRPPSLSSQALDLTMAVLYSVVPPTVNPLIYSMRNQELKDAVRKMVSWTHFSRDKLLICLYK
>ggOR130
MPNSSSISEFLLLALADTRQLQLLHFWLLLGIYLVALLGNGLISTAVACDHRLHTPMYFFLLNLALLDLGCISTTLPKAMA
NALWGTRAISYAGCAAQVFFFFFFFSAEYSLLTIMSYDRYIAICKPLHYGTLLGSRACATMAAAAWGAGVLNSLLHTASVF
SLPLCQGNAVDQFFCEIPQILKLSCSESYLREVELIVFSVAMEFGCFAFILFSYVQIFRAVLRMPSEQGRHKAFSTGQPWD
QSSSAFISGMDDEIESTLSNSADVTKLRGACGTTE
>ggOR131
MPNSSSISEFLLLALADTRQLQLLHFWLLLGIYLAALLGNGLISTAVACDHCLHTPMYFFLLNLALLDLGCISTTLPKAMA
NALWHTRHISYAGCAAQVFFFSFLFSAEYSLLTVMSYDRYIAICKPLHYGTLLGSRACATMAAAAWGYGILNSLLHTANTF
SLPLCQGNAVDQFFCEIPHILKLSC
>ggOR132
MPNSSSISEFLLLALADTRQLQLLHFWLLLGIYLAALLGNGLISTAVACDHRLHTPMYFFLLNLALLDLGCISTTLPKAMA
NALWHTRAISYAGCAAQVFFFLFFLSAEYSILTMMSYDRYVAICKPLHYGTLLGSRACATMAAAACGTGLLYAVLHTANTF
SLPLCQGNAVDQFFCEIPHILKLSCSQSYLKEIGLIALSVLVFFGSFVFIVVSYVQIFRAVLRMPSEQGRHKAFSMCLPHL
VVLSLFVSTAMFAYLKPHSISSPPVDLVVAVLYAVVPPVMNPLIYSMRNKELKVAMSELMNKCVSKAMNCSYFSVCHL
>ggOR133
MYFFLLNLALLDLGCISTTLPKAMANALWHTRHISYAGCATQVFFFLFLLSAEYSLLTVMSYDRYIAICKPLHYGTLLDNR
ACATMAAAAWGTGLLHAVLHTANTFSQALCRGKAVNQFFCEIPHILKLSCSESDYLRETGFIVVTVLALFGCFVFILFSYV
QIFRAVLMMPSEQGRHKAFSMCLPHLAVVSLFVSTDFLAYLKPHSLVSPSLDLVVSFLYSVVPPTLNPLIYSMRNQELKDA
DKVTHLVDEGKVVSVVYLDFSKASDAVRHSILVEKKMAAHSLDGRTLR
>ggOR134
MPNSSSISEFLLLALADMWQLQLLHFWLLLGIYLAALLGNGLISTAVACDHRLHTPMYFFLLNLALLDLGCISTTLPKAMA
NALWHTRHISYAGCAAQVFFIFFLFSAEYSILTIMAYDRYVAICKPLHYGTLLGSRACATMAAAAWGTGLLHAVLHTANTF
SQALCRGKAVNQFFCEIPHILKLSCSESDYLRETAFIVVTVLAIYGCFAFILFSYVQIFRAVLRMPSEQGRHKAFSMCLPH
LAVVSLFVSTDFLAYLKPHSLVSPSLDLVVSFLYSVVPPTLNPLIYSMRNQELKDAGKETPVGTVEAVGMQQELNGRLEED
SSGIPVHTQCL
>ggOR135
MPNSSSISEFLLLALADTRQLQLLHFWLLLGVYMAALLGNGLISTAVACDHRLHTPMYFFLLNLALLDLGCISTTLPKAMA
NALWHTRDISYAGCAAQLFFFLFFFSAEYYLITVMAYDRYVAICKPLHYRTLLSFRACATMAAAAWGTGLLYSLLHTANTF
SLPLCQGNAVDQFFCEIPQILKLSCSDAYLREAGLIMACILVLFGCFVFTVVSYVQIFRAVLKMPSEQGRHKAFSMCLPHL
AVVSLFASTAMFAYLKPPSVSFPSLDLVVSVLYSVVPPTLNPLIYGMRNQELKGALRKILQHALFRHQKDVHHALKTTKHL
GKA
>ggOR136
MPNSSSISEFLLLALADTRQLQLLLFWLLLGIYLAALLGNGLISTAVACDHRLHTPMYFFLLNLSLLDLACISTTLPKAMA
NALWDTRHISYAGCAAQVFFFLFFIFAEYSLLTVMSYDRYVAICKPLHYGTLLGSRACATMAAAAWGTGLLYALLHTATAF
SIPLCQGNVVDQFFCEIAQILKLSCSKSDYIREVGIIYFSLCLICGCFVFIVLSYVQIFRAVLRMPSEQGRHKAFSMCLPH
LAVVSLFVSTAMFAYLKSSSMSSPSLDLLVSFLYSVVPPAVNPLIYGLRNQELKAAIFRAVLRMPSEQGRHKAFSTCLPHL
AVVSLFVTTCMFAYLKPPSISS
>ggOR137
MPNSSSISEFLLLAFADTRQLQLLHFWLFLGIYLAALQGNGLIIIGIACDHRLHTPMYFFLLNLALLDLGCISTTLPKAMA
NALWDTRDISYAGCAAQCFLFVFLMSAEYSLLTVMSYDRYVAICKPLHYETLLGSRACASMAAAAWGSGVLNAVLQTANRF
SLPVCRGNAVNQFFCEIPHLLKLSCSISHLREIWATVVGVSLAFGCFVFILFSYVQIFRAVLRMPSEQGQHKAFSTCLPHL
AVVSLFVSAGFFSYLKPSSISFPSLDLVVAVLYSLVPPTLNPIIYSVRNKDIKCAVRKVTSHVSELSSLYSHFLPSLVSFA
VIVL
>ggOR138
MPNSSSISEFLLLALADTRQLQLLHFWLLLGIYLAALLGNGLISTAVACDHRLHTPMYFFLLNLALLDLGYISTTIPQAMA
NALWDTRAISYAGCAAQVFFSVFFFSAEYSLHTIMFYDRYVAICKPLHYGTLLGSRACATMAAAAWGTGLLYSLLHTANTF
SLPLCQGNVLDQFLCEIPQILKLTCSDAYLKEAGLIVISVLVILGCFVFIVVSYVQIFRAVLRMPSEQGRHKAFSTCLPHL
AVLSLLISSGSF
>ggOR139
MPNSSSIIEFLLLALADTRQLQLLHFWLLLGIYLAALLGNGLISTAVACDHRLPTPMYFFLLNLALLDLGCISTTLPKAMA
NALWHTRHISYAGCAAQVFFSLLFISAEYSLLTIMSYDRYVAICKPLHYGTLLGSRACATMAAAAWGTGVLYSLLHTANTF
SLPLCQGNAVDQFFCEIPQILKLSCSDSYLREVGLLLFSVCACLGCFVFIVVSYVQIFRAVLRMPSEQGRNKAFSTCLPHL
AVVSLFVSTGFFAYVKPPSISSPFIDLTVALLYSVVPPTLNPIIYSMRNKEIKHALRKLLQYVHYSSFNKVPIFLT
>ggOR140
MPNSSSISEFLLLAWADTRQLQLLHFWLLLGIYLAALXGNGLISTAVACDHRLHTPMYFFLLNLALLDLGCISTTLPKAMA
NALWHTRDISYAGCAAQVFFFLFFISAEYSLLTIMSYDRYVAICKPLHYGTLLGSRACASMAAAVWGTGLLYSLLHTANTF
SLPLCQGNAVDQFFCEIPHILKLSCSDAYLREVGLIMAGVIIFFACFVFIVVSYVQIFRAVLRMPSEQGRHKAFSMCLPHL
AVVSLFVSTGMFAYLKPPSISSPSLDLVVSFPYTVVPPAVNPLIYSLRNQELKGALRKIFQHALFHHQKDVHHVLRTTENL
GKA
>ggOR141
MPNSSSISEFLLLALADTRQLQLLHFWLLLGIYLAALLGNGLISTAVACDHRLHTPMYFFLLNLALLDLGCISTTLPKAMA
NALWHTRHISYAGCAAQVFFLLFFFGAEYSLLTIMSYDRYVAICKPLHYGTLLGSRACATMAAAAWGTGLLYSLLHTANTF
SIAFCLRTPSTRLGYPGPHPTWT
>ggOR142
MYFFLLNLALLDLGCISTTLPKAMANALWHTRAISYAGCAAQVFFFLFFLSAEYSILTMMSYDRYVAICKPLHYGTLLGSR

ACATMAAAACGTGLLYAVLHTANTFSLPLCQGNAVDQFFCEIPHILKLSCSQSYLKEIGLIALSVLVFFGSFVFIVVSYVQ
IFRAVLRMPSEQGRHKAFSMCLPHLVVLSLFVSTAMFAYLKPHSISSPPVDLVVAVLYAVVPPVMNPLIYSMRNKELKVAM
SELMNKCVSKAMNCSYFSVCHL
>ggOR143
MGAAEYSFLTVMSYDRYVAICKLLHYETLLGSRACASMAAAAWGCGVLNAVFQTANTFSLPFCRGNAVNQFFCEIPHLLKL
SCSNFFLRKSGFLLVSAMLTFGCFIFIVFSYVQIFRAVLRMPLKQGRHKAFSTCLPHLAVVSLFLTSDFLAYLKPPSISSP
PLDLAVSFLYSVVPPAVNPLIYSMRNQELKGALRKMMTLTHFSCGSQRKDVAAPIPIPLDLLHTWTSTASTNSPIYTPTDP
KRDVERKELIWD
>ggOR144
MMSYDRYVATFKPLHYRTLLGSRPCATMAAAAWGAGVLFSLLHTASTFSLPLCQGNAVDQFFCEIPHILKLSCSKSFKSYL
REVGFLLFSVCLALVVFVFIVVSYVQIFRAVLRIPSEQGRHKAFSTCLPHLAVVSLFLSTGSFAQMKPFSNSSPSLDLMVS
FLYSVFPPTLNPIIYSMRNKEIKHALSKVLQNVHYSSFNKMRICLT
>ggOR145
MSYDRYVAICKPLHYLTLLGSRACATMAASAWGTGLLYSLLHTASTFSLPLCQGNAVDQFFCEIPQILKLSCSKSYFREVG
LIVFSVLVLFACFAFILFSYVQMFRAVMRMPSEQGRHKAFSTCLPHLAVVTLFLSTAFFANLRPPSLSSQALDLTMAVLYS
VVPPTLNPLIYSMRNQELKDAVRKMIAWTHFSRDKLLISV
>ggOR146
MPNSSSISEFLLLALADTRQLQLLHFWLLLGIYLAALLGNGLISTAVACDHRLHTPMYFFLLNLALLDLGCISTTLPKAMA
NALWHTRHISYAGCAAQVFFLLFLFSTETFLLTIMSYDRYVAICKPLHYGTLMDIRACATMAAAAWGTGVLHSLLHTANTF
SIPLCQGNAVDQFFCEMAQVLKLSCSESDYLSEAGIIDFSLSLIFGCFVFIVVSYVQIFRAVLRMPSEQGRHKAFSTCLPH
IFVVSLFVSTLMFAYLKPSSLSSPSLDLVVAVLYSVVPPTLNPLIYSMRNQELRDALRKLWEIPSNIRKY
>ggOR147
MANARWHTRDISYAGCAAQVFFFVFFLSAEYSLFTIYDRYVAICKPLHYGTLLGSRACASMAAAVWGTGLLYSLLHTANTF
SLPLCQGNAVDQFFCEIPHILKLSCSQSYLREAGLLMFSALFFFASFVFIVVSYVQIFRAVLRMPSEQGRHKAFSTCLPHL
AVLSLFVITGFFAYLKPHSISSPSLDLMVSFLYSVVPPTLNPIIYSMRNREHKDALWKLMTKCIWKAMNCPSFSI
>ggOR148
MPNSSSISEFLLLALADTRQLQLLHFWLLLGIYLAALMGNGLISTAIACDHHLHTPMYFFLLNLALLDLGCISTTLPKAMA
NAFWGTRDISYAGCAAQVFFFLFFLAAEYSILTIMSYDRYVAICKPLHYGTLLGSRACATMAAAAWGTGVIYSLLHTANTF
SLPLCQGNAVDQFFCEIPHILKLSCSDAYLREIHICQRLWQASSSTWFAHWLSDPLFLNLCETLNRKGSRIWILISEV
>ggOR149
MYFFLLNLALLDLGCISTTLPKAMANALWGIRAISYTGCAAQVFFFLFFISAECCLLTIMSYDRYVAICKPLHYGTLLGSR
ACATMAAVAWGTGLLYALLHTANTFSLPLCQGNAVDQFFCEIPQILKLSFSDGYLREAGLIIAFVLAVFGCFAFILFSYVQ
IFRAVLRMLFEQGRHKAFSTCLPHLAMLSLLVSTAMFAYLKPPSISSPSLDLVVSFLYSVMPPALNPLIYGMRNQELKGAL
RKILQHTLFRHQKDVHHDLKTTKNLEKA
>ggOR150
MMSYDRYVAICKPLHYGTLQGSRACATMAAAAWGTGLLYSLLHTANTFSLPLCQGNVLDQFLCEIPQILKLTCSDAYLKEA
GLIVISVLVIFGCFVFIVVSYVQIFRAVLRMPSEQGRHKAFSMCLPHLAVLSLLISSASFAYLKPPSISTPFLNLVVSFLY
SVVPPTLNPLIYSMRNQELKDALGKLMTGCILKAMYCPSLSIHDL
>ggOR151
MMSYDCYVAICKPLHYGTLLGSRACAIMAAAAWGTGLFHALLHTANTFSLPLCQGNAVDQFFCEIPQILKLSCSDAYLREA
GLIVVSVLLELGCFVFIVVSYVQIFRAVLRIPSEQGRHKAFSTCLPHLAVLSLLVSSAFFAYLKPSSISSPSLDLVVSFLY
SVVPPAVNPLIYSMRNQELKDALWKLMTKYIWKAMNCPSLSLHVL
>ggOR152
MPNSSSISEFLLLALADTRQLQLLHFWLLLGIYLAALLGNGLISTAVACDHRLHTPMYFFLLNLALLDLGCISTTLPKAMT
NALWHTRDISYAGCAAQVFFLLFLFGAEYSLLIVMSYDRYVAICKPLHYGTVLGSRACATMAAATWGTGLLCSLLHTAN
>ggOR153
MPNSSSISEFLLLALADTRQLQLLHFWLLLGIYLAALLGNGLISTAVACDHRLHTPMYFFLLNLALLDLGCISTTLPKAMA
NALWHTRHISYAGCVAQVFFFLFVFSAEYSILTIMSYDRYVAICKPLHYG
>ggOR154
MPNSSSISEFLLLALADTRQLQLLHFWLLLGIYLAALLGNGLISTAVACDHRLHTPMYFFLLNLALLDLGCISTTLPKAMA
NALWHTRAISYAGCATQLFFFLFLFGAEYSLLTIMSYDRYVAICMPLHYGTLLGSRACATMAAAAWGTGLLYSLLHTASRI
PPQGENQ
>ggOR155
MPNSSSISEFLLLALADTRQLQLLHFWLLLGIYLAALLGNGLISTAVACDHRLHTPMYFFLLNLALLDLGCISTTLPKAMA
NALWHTRDISYAGCAAQVFFFSFLFSAEYCILTLMSYDRYVAICKPLHYGTLLGSRACATMAAAAWGYGILNSLLHTANTF
SLPLCQGNAVDHFFCEIPPILKLSCSKSYLREFGLIMAGGLVFFGCFLFIVVSYVQIFRAVLRIPSEQGRHKAFSTCLPHL
AVVSLFVSTAMFAYLKPHSISSPSLDLVVSVLYSVVPPAVNPLIYSMRSQELKDALGKVRQYTLFLHQKDVQHTLRTTKI
>ggOR156
MPNSSSISEFLLLALADTRQLQLLHFWLLLGIYLAALLGNGLISTAVACDHRLHTPMYFFLLNLALLDLGCISTTLPKAMA
NALWDTRAISYAGCAAQVVFFLFFLSAEYSLLTIMSYDRYVAICKPLHYGTLMDSRACATMAAAAWGTGLLYSLLHTANTF
SLPLCQGNAVEQFFCEIPQILKLSCSDSYLREVGFIVLSILAIFGCFVFIVVSYVQIFRAVLRMPSEQGRHKAFSTCLPHL
AVLSLLVSTATFANLKPPSFSSASMNLVVSFLYSVMPPAMNPLIYSMRNQELKDTLKKLMSWTLFINNKLLISC
>ggOR157
MPNTCSISEFLLLALADTRQLQLLHFWLLLGIYLAALLGNGLISTAVACDHRLHTPMYFFLLNLALLDLAYISTTIPQAMA
NALWDTRAISYAGCAAQVFFSVFFFSAEYCILTMMSYDRYVAICKPLHYGTLLGSRACATMAAAAWGTGLLYSLLHTANTF
SLPLCQGNVLDQFLCEIPQILKLTCSDAYLKEAGLIVISVLVIFGCFVFIVVSYVQIFRAVLRMPSEQGRHKAFSMCLPHL
AVLSLLISSASFAYLKPPSISTPFLNLVVSFLYSVVPPTLNPLIYSMRNQELKDALGKLMTGCI
>ggOR158

MGQEKTGKSRPVTEYYLLTVMSYDRYVAICMPLHYATLLDSRACGTMAAAAWGTGVLNSLLHTANTFSLPLCQGNAVDQFF
CEIPQILKLSCSDAYHREAGLIVAGVIIFFGCFAFILFSYVQIFRAVLRMPSEQGRHKAFSTCLPHLAVVSLFLITALVAN
LRPPSVSSQSRDLTLAVLYSVVPPTMNPLIYSMRNQELKDAVKKMIAWTHFSRGKLLICLYK
>ggOR159
MPNSSSISEFLLLALADTRPLQLLHFWLLLGIYLAALLGNGLISTAVACDHRLHTPMYFFLLNLALLDLGSISTTVPKAMA
NALWDTRAISYTACLSVLILMSAEFYLLTLMSYDRYVAICKPLHYGTLMGSTACASLAAAAWGTGLLHALLHTANTFSLPL
CRGNTVDQFFCEIPHIQKLSCSYPYLRNLSLMTFSIFLFSGCFVSIVLSYVQIFRAVLRMPSVQGRHKAFSTCLPHLAVVS
LSISTGLFTYLKPHSISSPAMDVSVAILYSVVPPAVNPLIYSVRNQALKDAI
>ggOR160
MPNSSSISEFLLLALADTRQLQLLHFWLLLGIYLAALLGNGLISTAVACDHRLHTPMYFFLLNLALLDLGCISTTLPKAMA
NALWHTRHISYAGCAAQVFFFLFFISAEYCILTIMSYDRYVAICKPLHYGTLLGSRACATMAAAAWGSGLLYSLLHTANTF
SLPLCQGNAVDQFFCEIPQILKLSCSDSNLREIWLLGGPSRDSEDLTYAEMPAAIPCSQPPTSPTAPQSPVIYTTVSTDLL
C
>ggOR161
MPNSSSISEFLLLALADTRQLQLLHFWLLLGIYLAALLGNGLISTAVACDHRLHTPMYFFLLNLALLDLGCISTTLPKAMA
NALWHTRAISYAGCAAQVFFFLFFISAEYCILTIMSYDRYVAICKPLHYGTLLGSRACATMAAAAWGTGLLYSLLHTANTF
SLPLCQGNAVDQFFCEIPQILKLACSES
>ggOR162
MPNSSSISEFLLLALADTRQLQLLHFWLLLGIYLAALLGNGLISTAVACDHRLHTPMYFFLLNLALLDLGCISTTLPKAMA
NALWHTRHISYTGCAAQVFFFLFFLTAEYSLLTIMSYDRYVAICKPLHYGTLLGSRACATMAAAAWGTGLLYSLLHTANTF
SLPLCHGNAVDQFFCEIPQILKLSCSDAYLREIWLLGVSASLVSGCFAFILFSYVQIFRAVLRMPSEQGRHKAFSMCLPHL
AVVSLFLSTAFVANLKPPSLSSQSLDLTMAVLYSVVPPTLNPLIYSMRNQELKDSMRKRIAWTHFSRDKLLICLYK
>ggOR163
MANSSSISEFLLLALADTRQLQLLHFWLLLGIYLAALLGNGLISTAVACDHRLHTPMYFFLLNLALLDLGCISTTLPKAMA
NALWHTRHISYAGCAAQVFFLLFFFGAEYSLLTIMSYDRYVAICKPLHYGTLLNSKACATMAATAWGTGLLYSLLHTAHTF
SITFCLKNAV
>ggOR164
MAAAAWGTGVLASVLHTANTFSLPLCQGNAVDQFFCEIPRILKLSCSQSYLREVGLIVFSVLVLFGCFVFILFSYVQIFRA
VLRMPSEQGRHKAFSTCLPHLAVLSLFLSTVVFADLKPFSISSPSLNLLVSFLYSVVPPTLNPIIYSMRNKEIRHALSEVF
QYTLFQRQ
>ggOR165
MPNSSSISEFLLLALADTRQLQLLHFWLLLGIYLAALLGNGLISTAVACDHRLHTPMYFFLLNLALLDLGCISTTLPKAMA
NALWHTRHISYAGCAAQVFFFLFFISAEYCILTIMSYDRYVAICKPLHYGTLLGSRACATMAAAAWGTGLLYSLLHTANTF
SLPLCQGNAVDQFFCEIPQILKLACSESEFLKELGLLSTSAFIGVGCFVFIVGA
>ggOR166
MGAMWHIALGLALSCGNAVDQFFCEIPQILKLSCSGAYLKEFGLIIFSIFIFFGCFAFIVVSYVQIFRTVLRMLSEQGRHK
AFSTCLPHLAVVSLFISTCMFAYLKPPSISSPSVDLVVAVLYTVVPPVMNPIIYSMRNKEIKQTLRKLFGQILLQHQ
>ggOR167
MGSWICCKMRMVMLPKLMDILQSVFAAREDKLILLVPAEYSLLTILLYDRYVAICKPLHYWTLLGSRACATMAAAAWGTGL
LYSMLHTANTFSLPLCQGNAVDQFFCEIPHMLKLSCSKSYLKEFGLIVISALVLFGCFAFILFSYVQIFRAVLRMPSEQGR
HKAFSTCLPHLAVVSLFLSTVVFAHLKPFSISSPPLDLVVSFLYSVVPPTLNPIIYSMRNQDIKHALSKVLQYALFQLQ
>ggOR168
MPNSSSISEFFLLALADTRQLQLLHFWLLLGIYLAALLGNGLISTAVACDHRLHTPMYFFLLNLALLDLGCISTTLPKAMA
NALWHTRAISYAGCAAQVFFFSFLFSAEYCILTIMSYDRYVAICKPLHYGTLLGSRACATMAAAAWGTGVVNALLHTANTF
SLPLCQGNAVDQFFCEIPQILKLSCSKSCLREVALIVISVLLVLGCFVFIVVSYVQIFRAVLMMPSEQGRHKAFSTCLPHL
AVVSLFLITDFLAYLKPPSLFSPSPNLMVSFLYSVVPPTLNPLIYSMRNQELRDAVRKMMPWSHFSKDELLICLCK
>ggOR169
MPNSSSISEFLLLALADTRQLQLLHFWLLLGIYLAALLGNGLISTAVACDHRLHTPMYFFLLNLALLDLGCISTTLPKAMA
NALWHTRHISYAGCAAQVFFFLFFLSAEYFLLTVMSYDRYVAICKPLHYGTLLGSRACATMAAAVWGGGV
>ggOR170
MPNSSSISEFLLLALADTRQLQLLHFWLLLGIYLAALLGNGLISTAVACDHRLHTPMYFFLLNLALLDLGCISTTLPKAMA
NALWHTRHISYAGCAAQVFFFFFLYSAEYSLLTIMSYDRYVAICKPLHYGTLLGSRACATMAAAAWGTGLLYSLLHTANTF
SLPLCQGNAVDQFFCEIPQLVKLSCSKSYLREDGLLLFSICLSFGCFVFIVVSYVQIFRAVLRMPSEQGRHKAFSTCLPHL
AVVSLLVSTGFFAYVKPHSQSSPFLDVTVALLYSVVPPTLNPIIYSMRNKEIKHALRKLLQYVHYSSFNNVPIFLT
>ggOR171
MSNSNSISEFLLLAFTDTRQLQLLHFWLFLGIYMAALLGNGLIIIGIACDHRLHTPMDFFLLNLALLDLGCISTTLPKAMA
NALWDTRDISYAGCAAQVFFFLFLFSAEYSLLTVMSYDRYVAICKPLHYETLLGSRACASMAAAAWGCGVLNAVLQTANTF
SLPFC
>ggOR172
MPNSSSISEFLLLALADTRQLQLLHFWLLLGIYLAALLGNGLISTAVACDHRLHTPMYFFLLNLALLDLGCISTTLPKAMA
NALWHTRAISYAGCAAQVFFFVFFLSTEYSLLTIMSYDRYVAICKPLHYRTLLGSRACATMAAAAWGSGLLYSLLHTATAF
SVPLCQGNAVDQFFCEIPQILKLSCSKSDYFREIGIIYFSLCLSFGCFVFIVVSYVQIFRAVLRMPSEQERHKAFSTAAAL
VGLQKKGAVSGDPWALETTPCP
>ggOR173
MSNSSSISEFLLLALADTRQLQLLHFWLLLGIYLAALLGNGLISTAVACKHRLHTPMYFFLLNLALLDLGCISTTLPKAMA
NALWDTRTISYAGCIAQTFLVFPLTIMSYDRNVAICKPLHYRTLLGSRACATMAAAAWGAGLPYSLLHTANTFSQPLCQGN
TVDQFFCEIPQILKISCSESDYLRETGFFAFSFSLVLGCSVFILFSYVQIFRAVLRMPFEQGRHKAFSTCLPHLAVVSLFL
STTMFAFLKLLSISSPSLDLVVAVLYSVVPPAVNPLIYSMRNQELKGALRKILQRTPFFHQKDVQHTLQTIKT

>ggOR174
MPNSSSISEFLLLALADTRQLQLLHFWLLLGIYLAALLGNGLISTAVACDHRLHTPMYFFLLNLALLDLGCISTTLPKAMA
NALWHTRHISYTGCAAQVFFFDFFISSEYSLLTIMSYDRYVAICKPLHYGTLLGSRACATMAAAAWATGVLNSLLHTANTF
SIPLCQGNAVNQFFCEIPHILKLSCSDAYLREVGLIMAGVLVFFGCFAFILFFYVQIFRAVLRMPSEQGRHKAFSTCLPHL
AVLSLLVSTAIFAYLKPPSISSPSLDLVVSFLYSVMPPALNPLIYGMRNQELKGALRKILQHTLFHYQKDVHHALKTTKHL
GKA
>ggOR175
MNLIKTFPYYKQWQEKLSVVEDSGAQWTTVSYGELLIRTLDKVSALLPSDQVYRSHLSVENIFHMLFLIRKPGMDTANAVD
HFFCEIPHILKLSCSQSYLKEIGLIALSVLVFFGSFVFIVVSYVQIFRAVLRMPSEQGRHKAFSTCLPHLAVLSLFLSTGM
FAYLKPPSISSTSLDLVVAVLYSVVPPALNPIIYSMRNQLLKDALWELMAKCLLKAMNCPSLYM
>ggOR176
MPNSSSISEFLLLAWADTRQLQLLHFWLLLGIYLAALLGNGLISTAVACDHRLHTPMYFFLLNLALLDLGCISTTLPKAMA
NALWHTRHISYAGCAAQVFFFLFFISAEYCILTIMSYDRYVAICKPLHYGTLLGSRACATMAAAAWGTGLLCSLLHTANTF
SLPLCQDNAVDQFFCEILHILKLSCSESDYFRETGFIVGCAEDAL
>ggOR177
MYFFFLNLALLDSGCISTTLPKAMTNAFWHTRAISYAGCTAQVFFFFFLFGAEYCILTIMSYDRYVAICKPLHYGTLLGSR
ACATMAAAAWGTGVLNSLLHTANTFSLPLCQGNAVHQFFCEIPQILKLSCSQSYLKEISLFVFSISLASGCFVFILFSYVQ
IFRAVLRMPSEQGRHKAFSTCLPHLAVLSFSLSTATFAYLKPPSLSSPSLDLVVAVLYTVVPPVMNPIIYSMRNQEIKQAL
RTLFGDTLLRHH
>ggOR178
TGCAAQVFFFFFLYSAEYSLLTIMSYDHYVAICKPLHYGTLLGSRACATMAAAAWGTGVLNSLLHTANTFSLPLCQGNAVD
QFFCEIPLIFKLSCSKSYLREVGLIVFSVLVLFGCFAFILFSYVQIFRAVLRMPSEHGRHKAFSTCLPHLAVVSLFLSTGF
FACLRPPSHSSPSLDLMMAFLYSVVPPTLNPLIYSMRNQELRDA
>ggOR179
MPNSSSISEFLLLALADTRQLQLLHFWLLLGIYLAALLGNGLISTAVACDHRLHTPMYFFLLNLALLDLGCISTTLPKAMA
NALWHTRHISYAGCATQFFFFLFLFGAEYCILTIMSYDRYVAICKPLHYGTLMDSRACATTAAAAWGSGLLYSLLHTASTF
SQALCRGKAVNQFFCEIPQILKLSCSESDYLRETGFIVLRVLAVYGCFAFILFSYVQIFRVVLRMPSEQGRHKAFSMCLPH
LAVVSLFVSTGFLAYLKPHSLVSPSLNLVVSFLYSVVPPTLNPLIYSMRNQELKDALIDLESECADVRQCPHQHPPSVKEH
LLGKYDAAQIFIPAHRHLALE
>ggOR180
MSYDRYVAICKPLHYGTLLGSRACATLAAAAWGTGLLYAVLHTANTFSLPLCQGNAVDQFFCEIPHILKLSCSQSYLKEIG
LIALSVLVFFGSFVFIVVSYVQIFRAVLRMPSEQGRHKAFSTCLPHLAVVFLFLSTGMFAYLKPPSISSTSLDLVVAVLYS
VVPPAVNPIIYSMRNQLLKDALWELMAKCLLKAMNCPCLYM
>ggOR181
MYFFLLNLALLDLGCISTTLPKAMANALWHTRDISYTGCAAQVFFLLFFFDAEYCILTIMSYDRYVAICKPLHYGMLFGSR
ACATMAAAAWGTGLLYSLLHTANTFSIAFCLSDVDQYFCEIAQILKLSCSESDYLRATGIIYFSLSLIFGCFVFILFSYVQ
IFRAVLRMPSKQGRHKAFLMCLPHLAVVSLFVSTVLFAYLKPPSISSPSLDLMAAVLYSVVPPAVNPLIYSMRNQEIKSAL
RKILQYTLVLHQKDVHHSLRTTKT
>ggOR182
MASYTRQLQLLHFWLLLGIYLAALLGNGLISTAVACDHRLHTPMYFFLLNLALLDLGCISTTLPKAMANALWHTRHISYAG
CAAQVFFFLFFLSAEYSLLTIMSYDRYVAICKPLHYGTLLGSRACATMAAAAWGNGILNSLLHTASTFSLPLCQGNTVDQF
FCEIPQILKLTCSKSFKAHLREVGLLLFSVCLAFVFFVFIVVSYVQIFRAVLRIPSEQGRHKAFSTCLPHLAVVSLFLSTG
SFAQLKPFSISSPFLDLMVSFLYSVLPSMLNPIIYSMRNKEIKHALSKVLQNVHYSSFNKVRICLT
>ggOR183
SLGPPPCTPPMYFFFLNLPLLDLGCIPTTFPKARANALWHTRHISYAGCAAQVFFYDFFISAEYSILTIMSYDRYVAICKP
LHYGTMLGGTACATMAAAAWGTGLLFLLLHTANTFSLPLCQGNAVDQFFCEIPHILKLSCSDAYLREVGLIVAFFHVLFGC
FVFIVVSYVQIFRAVLRMPSEQGRHKAFSTCLPHLAVVSLFVSTTTFAYLKPPSISSPSLNLLVSVLYSVVPPALNPLIYS
MRNQELKGALWKILQHTLFHYQKDVHHALKTTENLGKA
>ggOR184
MPNSSSISEFLLLALADTRQLQILHFWLLLGIYLAALLGNGLISTAVACDHRLHTPMYFFLLNLALLDLGCISTTLPKAMA
NALWHTRAISYAGCAAQLFFFVFFISAEYSLLTIMS
>ggOR185
MPNSSSISEFLLLALADTRQLQLLHFWLLLGIYLAALLGNGLISTAVACDHRLHTPMYFFLLNLALLDLGCISTTLPKAMA
NALWHTRHISYAGCATQVFFFLFLFSAEYSLLTIMSYDRYVAICKPLHYGTLLGSRACATMAAAAWGAGVLNSVLHTANTF
SLPLCQGNAVDQFFCEIPQILKLSCSQFYLKEISLLVFRLC
>ggOR186
MPNSSSISEFLLLALADTRQLQLLHFWLLLGIYLAALLGNGLISTAVACDHRLHTPMYFFLLNLALLDLACISTTLPKAMA
NALWGTRAISYAGCAAQVFFFVFFLSAEYSLLTIMSYDRYVAICKPLHYGTLLGSRACALSRGWKDPNSFSQHALSMYYRD
LISLYSM
>ggOR187
MAVAXWGTGVLYSLLHTANTFSLPLCQGNAVDQFFCEIPQILKLSCSDAYLREAGLIAFSVAMEFGCFIFIVVSYVQIFRA
VLRMPSEQGRHKAFSTCLPHLAVLSLFLSTGMIAYLKPHSVSSPSLDIVVSFLYSVVPPALNPLIYSMRNQELKDALWKLM
TKRLLKAMNCPCLSI
>ggOR188
MPNSSSVSEFLLLALADTRQLQLLHFWLLLGIYLAALLGNGLISTAVACDHRLHTPMYFFLLNLALLDLGCISTTLPKAMA
NALWHTRAISYAGCAAQVFFFLFFLAAEYSILTIMSYDRYVAICKPLHYGTLLGSRACATMAAAAWGTGLPNAVLQTANTF
SLPLCQGNAVDQFFCEIPHILKLSCSESYLREVGLLLFSVCLTVGWFVFIVVS
>ggOR189

MYFFLLNLALLDLGCISTTLPKAMANAFWHTRAISYAGCAAQVFFLLFFFSAEYCLLTMMSYDRYVAICKPLHYGTLLGNR
ACATMAAAAWGTGVLYSLLHTVNTFSLPLCQGNAVDQFFCEIPHILKLSCSESGSLRETGFIVLSVLAVYGCFAFILFSYV
QIFRAVLRMPSEQGRHKAFSTCLPHLAVVSLFLITDLFAYLKPPSLFSPSLNLMVSFLYSVVPPTLNPLIYSMRNQELRDA
ANEMPAGNTEII
>ggOR190
MAAAAWGSGLLCSLLHTASTFSLPFCQGNAVDQFFCEIPQILRLSCSESDNRRETGFIALSVLAVFGCFVFILFSYVQIFR
AVLRMPSEQGRHKAFSTCLPHLVVLSLFLSTVFFAYLKPFSISSPSLDLTVALLYSVVPPALNPLIYSMRNQEIKHALSIV
LQYALFQIQ
>ggOR191
MSYDRYVAICKPLHYGTLLGSRACATMAAAAWGTGLLYAVLHTANTFSLPLCQGNAVDQFFCEIPHILKLSCSQSYLKEIG
LIALSVLVFFGCFVFIVVSYVQIFRAVLRMPSEQGRHKAFSTCLPHLAVVSLFVSTAIFAYLKPPSISSTSLDLVVAVLYS
VVPAALNPIIYSMRNQLLKDALWELMAKCLLKAMNCPSLYM
>ggOR192
VDSPRLACISTTLPKAMATVNWHTRDISYAGCPSKVFFFVFFISAEYSLLTIMSYDRYVAICKPLHYGTLLGSRACATMAA
AAWGTGVLNSLLHTASTFSLPLCQGNAVDQFFCEIPHILKLSCSDAYLREIGLLVVTASLVSGCFVFIVLSYVQIFRAVLR
MPSEQGRHKAFSTCFPHLVVVFLFVNTASVAYLKPPSVSSPSLDLVVSFLYSLVPPTLNPLIYSLRNKELMCSVRKVISRM
FLYCDKSALCFQNY
>ggOR193
MNDVRLPPPLRSHPPTWHPTKPKQHTEYSLLTVMSYDRYVAICKPLHYGTLLGSRACATMAAAAWGTGLLNSLLHTANTFS
LPLCQGNAVDQFFCEIPQILKLSCSDAYLREIWLLGVSASLVSACFVFIVVSYVQIFRAVLRTPSEQGRHKAFSTCLPHLA
VVSLFVSTATFAYLKPPSLSSPSLDLVVAVLYTVVPPVMNPIIYSMRNQEIKQALRTLFGDALLRHH
>ggOR194
MPNSSSISEFLLLALADTRQLQLLHFWLLLGIYLAALLGNGLISTALACDHRLHTPMYFFLLNLALLDLGCISTTLPKAMA
NALWGTRAISYAGCAAQVFFFVFFLSAEYSLFTMMSYDRYVAICKPLHYGTLLGSRACATMAAAAWGTGALYSLLHTANTF
SLPLCQGNLVDQLS
>ggOR195
MANALWHTRAISYAGCAAQVFFFFFFLSAEYSLLTIMSYDRYIAICKPLHYGTLLGSRACATMAAAAWVTGVLNSLLHTAN
TFSLLFCQGNAVHQFFCEIPHILKLSCSDAYLREIWLLGVSATLSSGCFVFILFSYVQIFRAVLRMPSEQGRHKAFSTCLP
HLAVVFLFLTTAFFANLRPPSVSSQSLDLTMAVLYSVVPPTLNPLIYSMRNQELKDAANEMAAGTVKLI
>ggOR196
MPNSSSISEFLLLALADTRQLQLLHFWLLLGIYLAALLGNGLISTAVACDHRLHTPMYFFLLNLALLDLGCISTTLPKAMA
NALWHTRHISYAGCAAQVFLFAFLQSAE
>ggOR197
MPNSSSISEFLLLALADTRQLQLLHFWLLLGIYLAALLGNGLISTAVACDHRLHTPMDFFLLNLALLDLGCISTTLPKAMA
NALWHTRAISYAGCAAQVFFFVFFLSAEYCLLTMMSYDRYVAICKPLHYGTLLGSRACATMAAAAWGIGFFNSLLHTASTF
SLPLCQGNAVDQFFCEIPQILKLSCSKSFKSYLREVGFLLLRVCLALVFFVFIVLSSVQIFRAVLRMPSEQGRHKAFSTCL
PHLAVVSLFLSTGSFAQMKPFSNSSPFLDLMVSFLYSVLPPTLNPIIYSMRNKEIKHALSKVLQNVHYSSFNKVRICLT
>ggOR198
MMSYDRYVPIGRPLHYGTLLGSRACATMAAAAWGNGILNSLLHTANTFSLPLCQGNAVDQFFCEIPQILKLSCSKSFKAHL
REVGFLLFSVCLALVFFVLIVVSYVQIFRAVLRMPSEQGRHKAFSTCLPHLAVVSLFLSTGSFAQMKPFSISSPFLDLMVS
FLYSVLPSMLNPIIYSMRNKEVKHALSKVLQNVHYFSFNKVRICLT
>ggOR199
MPNSSSISEFLLLALADTRQLQLLHFWLLLGIYLAALLGNGLISTAVACDHRLHTPMYFFLLNLALLDLGCISTTLPKAMA
NALWDTRHISYAGCAAQVFFFLFFLSAEYSILTIMSYDRYVAICKPLHYGTLLGSRACASMAAAAWGTGLLYSLLHTANTF
SLPLCHGNVVDQFFCEIPHILKLSCSDAYLREVAFIVLGVLVFVGCFAFILFSYVHVFRAVLRMPSEQGQHKAFSMCLPHL
AVLSLLVSTATFANLKPPAFSSASMNLVVSFLYSVVPPALNPVIYSMRNQELRDTLGKLMSWTIFINNKLLISC
>ggOR200
MPNSSSISEFLLLALADTRQLQLLHFWLLLGIYLAALLGNGLISTAVACDHRLHTPMYFFLLNLALLDLGCISTTLPKAMA
NALWHTRHISYAGCAAQVFFFLFLLSAEYYLLTVMAYDRYVAICKPLHYGTLLGSRACATMAAAARGTGLSVISKLEGTLN
PAVYVIDKNIEEHQFQDGPLGGTTCVQPPPG
>ggOR201
MANALWHTRAISYAGCAAQVFFFLFFLSAEYFLLTVMSYDRYVAICKPLHYGTLLGSRACATMAAAAWGTGLLYALLHTAD
TFSLPLCQGNAVDQLFCEIAQILKLSCSKSDYLSEVGIIYFSLCLIFGCFVFILFSYVQIFRAVLRMPSEQGRHKAFSMCL
PHLAVVSLFVSTAMFAYLKAPSLFSPSLNLLVSFLYSVVPPAVNPLIYGLRNQELKAAVSNTYFYSIRKMHGSLTGVPR
>ggOR202
MYFFLLILALLDLGCISTTLPKAMANALWDTRAISYAGCAAQVFFFVFFISAEYCLLTIMSYDRYVAICKPLHYRSLLGSR
ACATMAAAAWGTGLPNAVLQTANTFSLPLCQGNAVDQFFCEIPHILKLSCSKFYFREVGLLLFIVCLSFGCFVFIVVSYVQ
IFRAVLRMPSEQGQHKAFSTCLPHLAVVSLFLSTVVFAHLKPFSISSPSLDLLVALLYSVVPPTLNPII
>ggOR203
MPNSSSISEFLLLALADTRQLQLLHFWLLLGIYLAALLGNGLISTAVACDHRLHTPMYFFLLNLALLDLGCISTTLPKAMA
NALWHTRAISYAGCAAQVFFFLFFISSEYSILTIMSYDRYVAICKPLHYGTLLGSRACATMAAAAWGTGVLYSLMHTASVF
SLPLCQGNAVDQFFCEIPQILRLSCSESEFLRELGLLSTSAFIGVGCFVFIVVSYVQIFRAVLRMPSEQGRHKAFSTCLPH
LAVVSLFLSTGMAAYLRPSSISSPFLDLVVSFMYSVVPPTLNPIIYSMRNQELKNSLWKLMTRCIMKTMNCSS
>ggOR204
MSYDRYVAICKPLHYRTLLGSRACATMAAAAWGTGLLNALLHTANTFSLPLCHGNVVDQFFCEIPHILKLSCSDAYLREVG
LIVFSALALLGCFVFILFSYVQIFRAVLRMPSVQGRHKAFSTCLPHLAVLSLLVCSAFFAYLKPPSISSPFLDQLVSFLYS
VVPPALNPLIYSIRNQELKDALWKLMTKRLLKAMNCPSLSI
>ggOR205

MSNSSSISEFLLLAFTDTRQLRLLHFWLFLGIYLAALLGNGLISTAVACDHRLHTPMDFFLLNLALLDLGCTSTTLPKAMA
NALWDTRDISYAGYAARCFLFVFLMSAEYSFLTVMSYDRYVAICKPLHYGTLLGSRACASMAAAAWGSGVLHALLHTANTF
ALPLCQGKAVDLFFCEIPHLLKLSCSDTYVRETGLLLTFCGFVFSGCITFLVLSYVQIFRAVLRMPSDQGLFHVPPSPGCG
LPVCQCWFLCLPESPSSISYPSLDLVVAVHHLVEFPGVNPLIYITRNQDLKGALRVPSLPPSLLPFPLSFLFSPS
>ggOR206
MSYDPFIAICKPLHYGTLLGSKACATMAAAAWGTGLLHAVLHTANTFSQALCRGKAVNQFFCEIPHILKLSCSESDYLRET
GFIVVTVLALFGCFVFILFSYVQIFRAVLRMPSEQGRHKAFSMCLPHLAVVSLFVSTDFLAYLKPHSLVSPSLDLVVSFLY
SVVPPTLNPLIYSMRNQELKDAEGDGTAYCGCHLQAIGREEGYEEESAWVHQE
>ggOR207
MANALWHTRHISYAGCAAQVFFFFFLYSAEYSLLTIMSYDRYVAICKPLHYGTLLGSRACATMAAAAWGTGVLNSLLHTAN
TFSLPVCQGNAVDQFFCEIPQLVKLSCSKSYLREDGLLLFSICLSFGCFVFIVVSYVQIFRAVLRMPSEQGRHKAFSTCLP
HLAVVSLLVSTGSLAYVKPHSQSSPFLDVTVALLYSVVPPTLNPIIYSMRNKEIKHAIRKVLQYVHYSSFNKVPIFLT
>ggOR208
MPNSSSISEFLLLALADTRQLQLLHFWLLLGIYLAALLGNGLISTAVACD
>ggOR209
MPNSSSVSEFLLLALADTRQLQLLHFWLLLGIYLAALLGNGLISTAVACDHRLHTPMYFFLLNLALLDLGCISTTLPKAMA
NALWHTRAISYAGCAAQVFFFFFLFSAEYSLLTIMSYDRYVAICKPLHYRTLLGSRACATMAAAAWATGLLYSLLHTAYTF
SLPLCQGNAVDQFFCEIPQILKLSCSESYCREIWLLGVNASLVSGCFAFILFSYVQIFRAVLRMPSEQGRHKAFSTC
>ggOR210
MPNSSSISEFLLLALADTRQLQLLHFWLLLGIYLAALLGNGLISTAVACDHRLHTPMYFFLLNLALLDLACISTTLPKAMA
NALWHTRDISYAGCAAQVFFFVFFISAEYSLLTIMSYDRYVAICKPLHYGTLLGSRACATMAAAAWGTGVLNSLLHTASTF
SLPLCQGNAVDQFFCEIPHILKLSCSDAYL
>ggOR211
MSYDRYVAICKPLHYGTLLGSRACATMAAAAWGTGVIYSLLHTANTFSLPLCQGNAVDQFFCEIPHILKLSCSDAYLREVG
LIVLSVPMEFGCFVFIVVSYVQIFRAVLRMPSEQGRHKAFSTCLPHLAVVSLFLSTGMVAYLKPHSVSSPSLDIVVSFLYS
VVPPALNPLIYSMRNQELKDALWKLMTNRLLKAMNCPSLSI
>ggOR212
MPNSSSISEFLLLALADTRQLQLLHFWLLLGIYLAALLGNGLISTAVACDHHLHTPMYFFLLNLSLLDLGCISTTLPKAMA
NALWGTRAISYTGCAAQVFFFLFFISAECCLLTIMSYDRYVAICKPLHYRTLLGSRACATMAAVVWGTGLLYSLLHTANTF
SLPLCQGNAVDQFFCEIPQILKLSFSDGYLREAGLIIAFVLAVFGCFAFILFSYVQIFRAVLRMLFEQGRHKAFSTCLPHL
AMLSLLVSTAMFAYLKPPSISSPSLDLVVSFLSQ
>ggOR213
MANSSSISEFLLLALADTRQLQLLHFWLLLGIYLAALLGNGLISTAVACDHRLHTPMYFFLLNLALLDLGCISTTLPKAMA
NALWHTRHISYAGCAAQVFFFFFLFSAEYSLLTVMSYDRYVAICKPLHYGTLLGSRACATMAAAAWGTGLLYSLMHTASIF
SLPLCRGNAVDQFFCEIPHILKLSCSGSEFLRELGLLSTSAFIGIGCFVFIVVSYVQIFRAVLRMPSEQGRHKGFSTCLPH
LVVLSLFVSTAMFAYLKPHSISSPPVDLVVAVLYAVVPPVMNPLIYSMRNKELKVALSELMNKCVSKAMNCSYFSVCHL
>ggOR214
MPNSSSISEFLLLALADTRQLQLLHFWLLLGIYLAALLGNGLISTAVACDHRLHTPMYFFLLNLALLDLGCISTTLPKAMA
NALWHTRHISYAGCAAQVFFLLFLFSTETFLLTIMSYDRYVAICKPLHYGTLMDIRACATMAAAAWGKRVLHSLLHTA
>ggOR215
MSYDRYVAICKPLHYGTLLGSRACATMAAAAWGTGLLYSLLHTANTFSLPLCQGNAVDQFFCEIPQILKLACSESEFLREL
GLLSTSAFIGVGCFVFIVVSYVQIFRAVLRMPSEQGRHKAFSTCLPHLAVLSLLVSTVVFAYLKPPAISFQSLDLVVSFLY
TVGPPTVNPLIYSMRNQKLKDALWKLVTSCILKTMNCPSLSVHDL
>ggOR216
MSNSSSISEFLLLALADTRQLQLLHFWLLLGIYLAALLGNGLISTAVACDHRLHTPMYFFLLNLALLDLGCISTTLPKAMA
NALWHTRHISYAGCAAQVFFFLFFLSAEYSLLTIMSYDRYVAICKPLHYGTLLSSRACATMAAAAWGTGAL
>ggOR217
MPNSSSISEFLLLALADTRQLQLLHFWLLLGIYLAALLGNGLISTAVACDHRLHTPMYFFLLNLALLDLGCISTTLPKAMA
NALWHTRHISYAGCAAQVFFYDFFISAEYSILTIMSYDRYVAICKPLHYGTLLGGRACATMAAAAWGTGLLYALLHTANTF
SLPLCQGNAVEQFFCEIPQILKLSC
>ggOR218
MSYDRYVAICKPLHYRTLLGSRACATMAAAAWGTGLLYSLLHTASTFSQALCRGKVVNQFFCEIPHILKLSCSESGSLRET
GFIVLSVLAIYGCFAFILFSYVQIFRAVLRMPSEQGRHKAFSTCLPHLAVVSLFVSTDFLAYLKPHSLVSPSLDLVVSFLY
SVVTPTLNPLIYSMRNQELKDAVRKIMSWTHFSSNKLLICLYK
>ggOR219
MPNSSCISEFLLLALADTRQLQLLHFWLLLGIYLAALLGNGLISTAVACDHRLHNPMYFFLLNLALLDLGCISTTLPKAMA
NALWHTRAISYAGCAAQLFFFLFFLSAEYCILTIMSYDRYVAICKPLHYGTLLGSRACATMAAAAWGTGALYALLQTATAF
SLPLCQSNAVDQFFCEVAQILKLSCSKSDYVREVGIIYFSLCLICGCFVFIVVSYVQIFRAVLRMPSEQGRHKAFSMCLPQ
GAAVSLFVSTAMFDCLKPPSISSPSLDL
>ggOR220
MPSDNQTYVTGFILVGFPGKPLIQLLLFLIFFLAYVLTVTENVIIVVLVWTNIQLHKPMYIFLGNLSFLEIWYISVTVPKV
LVSLVIKSQGISFTGCMAQLFFFLALVCTECTLLAVIAYDRYVAVCKPLHYPIIMNCTLCAHLSIGSWVSGFLISTGKVYF
ISHQTYCGPNIINHFFCDVSPLLKLACTNASLAELMDFLLALLILLVPLTVIVASYVCIIPVVLGIPTAQGRHKAFSTCAS
HLLVVTVFYTASLFIYARPQPIDSFSSNKLVSVIYTVFTPLINPIIYCLRNQEFKIALRKTTCWRDI
>ggOR221
MDENYTVVREFVLLGLSQTHKVQSVLFFFFLLFYMIILPGNILTILTIQGDSQLGSPMYFFLANLAFLDICYCSVTPPKML
ADFFSNPATISYNACMIQLLFLHSLGAAEAFLLLAMAYDRYVAICKPLHYTVLVNRTVCCVLVGASWGSGFIHGIILFALT
IKLPFCGPNILDNFFCDVRQLAKLACANTYTVELLMFLNNGVVIVLCFALLLISYTFLLLKLWTQSTKAKNRIISSCVSHI

IVVFVMCGPAIYIYVLPFQTVPMEKVVALFHTVIFPLTNSMIYTLCNKEIKSSMWKLVSKHILWCRL
>ggOR223
GCIFTTFPKAMANALWATRAISYAGCAAQVFFFVFFISAEYCILTIMSYDRYVAICKPLHYGTLLGSRSCATMAAAAWGTG
LLYALLHTANTFSLPLCQGNAVDQFFCEIPQILKLSCSESYLREVGLLLFSVCACLGCFVFIVVSYVQIFRAVLRMPSEQG
RHKAFSMCLPHLAVVSLFVGTGFFAYVKPPSISSPFIDLTVALLYSVVPPTLNPIIYSMRNKEIKHALRKLLQYVHYSSFN
KVPIFLT
>ggCOR3
MASGNCTTPTTFILSGLTDNPGLQMPLFMVFLAIYTITLLTNLGLIRLISVDLHLQTPMYIFLQNLSFTDAAYSTVITPKM
LATFLEERKTISYVGCILQYFSFVLLTTSECLLLAVMAYDRYVAICKPLLYPAIMTKAVCWRLVESLYFLAFLNSLVHTCG
LLKLSFCYSNVVNHFFCDISPLFQISSSSIAISELLVIISGSLFVMSSIIIILISYVFIILTVVMIRSKDGKYKAFSTCTS
HLMAVSLFHGTVIFMYLRPVKLFSLDTDKIASLFYTVVIPMLNPLIYSWRNKEVKDALRRLTATTFGFIDSKAVQ
>ggCOR6
MASGNCTTPTTFILSGLTDNPGLQMPLFMVFLAIYTITLLTNLGLIALISIDLQLQTPMYIFLQNLSFTDAVYSTVITPKM
LATFLEETKTISYVGCILQYFSFVLLTVRECLLLAVMAYDRYAAICKPLLYPAIMTKAVCWRLVKGLYSLAFLNFLVHTSG
LLKLSFCSSNVVNHFFCDNSPLFQISSSSTALNELLVFIFGSLFVMSSIITILISYVFIILTVVRIRSKERKYKAFSTCTS
HLMAVSLFHGTIVFMYFQPANNFSLDKDKIMSLFYTVVIPMLNPLIYSWRNKEVKDALHRAIATAVLFH
>ggCOR5
MALGNCTTPTTFILSGLTDNPRLQMPLFMVFLAIYTITLLANLGLIALISVDFHLQTPMYIFLQNLSFTDAAYSTVITPKM
LATFLEERRTISYVGCILQYFSFVLLTSSECLLLAVMAYDRYVAICKPLLYPAIMTKAVCWRLVEGLYSLAFLNSLVHTSG
LLKLSFCSSNVVNHFFCDNSPLFQISSSSTTLNELLVFIFGSWFAMSSIITTPISYVFIILTVVRIRSKDGKYKAFSTCTS
HLMAVSLFHGTVIFMYLRPVKLFSLDTDKIASLFYTVVIPMLNPLIYSWRNKEVKDALRRVIATNVWIH
>ggCOR4
MASGNCTTPTTFILSGLTDNPGLQMPLFMVFLAIYTITLLTNLGLIALISVDLHLQTPMYIFLQNLSFTDAAYSTVITPKM
LATFLEERKTISYIGCILQYFSFVLLTVTESLLLAVMAYDRYVAICKPLLYPSIMTKAVCWRLVKGLYSLAFLNSLVHTSG
LLKLSFCSSNVVNHFFCDNSPLFQISSSSTTLNELLVFIFGSLFAMSSIITILISYVFIILTVVRIRSKDGKYKAFSTCTS
HLMAVSLFHGTVIFMYLRPVKLFSLDTDKIASLFYTVVIPMLNPLIYSWRNKEVKDALRRVIATNVWIH
>ggCOR1
MASGNCTTPTTFILSGLTDNPGLQMPLFMVFLAIYTITLLTNLGLIALISVDLHLQTPMYIFLQNLSFTDAAYSTVITPKM
LATFLEERKTISYVGCILQYFSFVLLTVTESLLLAVMAYDRYVAICKPLLYPSIMTKAVCWRLVESLYFLAFLNSLVHTSG
LLKLSFCYSNVVNHFFCDISPLFQISSSSIAISELLVIISGSLFVMSSIIIILISYVFIILTVVMIRSKDGKYKAFSTCTS
HLMAVSLFHGTVIFMYLRPVKLFSLDTDKIASLFYTVVIPMLNPLIYSWRNKEVKDALRRLTATTFGFIDSKAVQ
>ggCOR2
MASGNCTTPTTFILSGLTDNPRLQMPLFMVFLVIYTTTLLTNLGLIALIGMDLHLQTPMYIFLQNLSFTDAAYSTVITPKM
LATFLEERRTISYVGCILQYFSFVLLTTSEWLLLAVMAYDRYVAICKPLLYPSIMTKAVCWRLVKGLYSLAFLNSLVHTSG
LLKLSFCSSNVVNHFFCDNRPLFQISSSSTTLNELLVIISGSLFVMSSIITILISYVFIILTVVMIRSKDGKYKAFSTCTS
HLMAVSLFHGTVIFMYLRSVKLFSLDTDKIASLFYTVVIPMLNPLIYSWRNKEVKDALRRLTATSVWLH
>ggCOR7a
MEEKNSTQINIFLLLGFPGLMDLKVLLFIVLLLTYIVTVVENVLIISLIKANHELCKPMYFFLGQLSFIEVWYISVTVPKL
LANFIAEDRSISFAGCMTQLYFFISFMCTECVLLSAMAYDRYVAICQPLHYLFIMTYQMCVCLIALSWFTGFTVSLIKISF
ISQLEFCGSHVINHFFCDISPVLNLACTDMSVAEMVDFVLALFILLVPLSITVVSYLLIITTILHIPTTQSKKKAFSTCAS
HLTVVVIFFSATLFMYARPKKISSYDMNKLVSAVYTIVTPMLNPFIYCLRNQELKRAFKKALSVKITVSEVSRTIPSIQM
>ggGPR148
MRIFCASVIRMNTSLLRQSNTSSDLEVSSYYLLEEWSLNTPDTNVKMFLIPPVVCLVAGVLIIPSILFVIFSRLSIRQETR
YMLLGNALLCDLLYLLLYTLSAALSAAHVSLPKEACILQLFSLAVAYCGGLFTAAAMVLDTYIAILFPLRYIAILPSSRTR
KVIVLLWVCSGALPGVFFLVLSSTHSFVPCVLEMCAIPVIILLTLSGTDAVKLSFWLSAVVIFLFLSLIFCCYGVLYFKTR
RSGIWESICSRASVTFLMHNTVFFFYFSPLLVLFVESFLYVNIVIGLQTSIWISLTICNVLMILPKVLFPFLYGLRYREIS
ASLRSLVRIQRLHLVSPAPLPS
>ggGPR136n1
HPVVDYGAGVFLLIIAILTILGNSAVLATAVKRSSLLKSPELLTVNLAVADIGMAISMYPLAIASAWNHAWLGGDASCIYY
ALMGFLFGVCSMMTLCAMAVIRFLVTNSSKSNSNKISKNTVHILITFIWLYSLLWAILPLVGWGYYGPEPFGISCTIAWSK
FHSSSNGFSFILSMFLLCTVLPALTIVACYLGIAWKVHKAYQEIQNINRIPHAAKLEKKLTLMAVLISVGFLSAWTPYAAA
SFWSIFNSSDSLQPIVTLLPCLFAKSSTAYNPFIY
>ggMECn1
MNGYSNCSANHTEIWSRNLVLALGIPQLIINIASVIFNCTAIITRKATKDLHKPISILFCNLAFSDLFTSFSGFWISMLFI
TKPDDTIFGSKDMLAPYALYTTSILSTIYNLVSIGIERYLAVAESIRTRFRVARSHSIAVVLINWVLAFFLGCLPLMGWNC
LHTGKNLSVLYSPFCVDYLIFITTPNVVAAFIVPLFTYLRIIMILRKRKLRMKACGQAIGTYKSAEIQVARTSIFIWLLAL
VSYAPLFAGVIFDAINHQCPLDLYPSVYIFRNCTAMLITMNCLGNPIIYTLKAKTLGAKLKSLKCLSGKRLQACTIANI
>ggRGR
MVTSHPLPEGFTEIEVFAIGTALLVEALLGFCLNGLTIISFRKIKELRTPSNLLVLSIALADCGICINAFIAAFSSFLRYW
PYGSEGCQIHGFQGFLTALASISSSAAVAWDRYHHYCTRSKLQWSTAISMMVFAWLFAAFWATMPLLGWGEYDYEPLRTCC
TLDYSKGDRNYITFLFALSIFNFMIPGFIMMTAYQSIHQKFKKSGHYKFNTGLPLKTLVICWGPYCLLSFYAAIENVMFIS
PKYRMIPAIIAKTVPTVDSFVYALGNENYRGGIWQFLTGQKIEKAEVDNKTK
>ggRE2
MSSNSSLSNGNGLKNLTTQEDGAVRVTEAIAIIVIAVFICVGNLIIVVTLYRKSYLLTLSNKFVFSLTLSNFLLSVLVLPF
VVTSSIRREWIFGVVWCNFSALLYMLISSASMLTLGLIAIDRYYAVLYPMVYPMKITGNRAVVALVYVWLHSLIGCLPPLF
GWSSLEFDQFKWMCVAAWHKEAGYTAFWQVWCALLPFVVMMICYGFIFRVARIKARKIHCGSVVIVEEDSQRNGRKNSSTS
TSSSGSRRNAFQGVVYSANQCKAFITILVVIGAFVITWGPYMVVITSEALWGKNSISPALETLATWLSFSSAICHPLIYGL
WNKTVRKELLGMCFGDRYYRESFVQRHRTSRLFSISNRITDLGLSPHLTALMAGGRPLGNSSSTGDTGFSCSQDSGK

```
>ggBlueSensitiveOpsin
MHPPRPTTDLPEDFYIPMALDAPNITALSPFLVPQTHLGSPGLFRAMAAFMFLLIALGVPINTLTIFCTARFRKLRSHLNY
ILVNLALANLLVILVGSTTACYSFSQMYFALGPTACKIEGFAATLGGMVSLWSLAVVAFERFLVICKPLGNFTFRGSHAVL
GCVATWVLGFVASAPPLFGWSRYIPEGLQCSCGPDWYTTDNKWHNESYVLFLFTFCFGVPLAIIVFSYGRLLITLRAVARQ
QEQSATTQKADREVTKMVVVMVLGFLVCWAPYTAFALWVVTHRGRSFEVGLASIPSVFSKSSTVYNPVIYVLMNKQFRSCM
LKLLFCGRSPFGDDEDVSGSSQATQVSSVSSSHVAPA
>ggVioletSensitiveConeOpsin
MSSDDDFYLFTNGSVPGPWDGPQYHIAPPWAFYLQTAFMGIVFAVGTPLNAVVLWVTVRYKRLRQPLNYILVNISASGFVS
CVLSVFVVFVASARGYFVFGKRVCELEAFVGTHGGLVTGWSLAFLAFERYIVICKPFGNFRFSSRHALLVVVATWLIGVGV
GLPPFFGWSRYMPEGLQCSCGPDWYTVGTKYRSEYYTWFLFIFCFIVPLSLIIFSYSQLLSALRAVAAQQQESATTQKAER
EVSRMVVVMVGSFCLCYVPYAALAMYMVNNRDHGLDLRLVTIPAFFSKSACVYNPIIYCFMNKQFRACIMETVCGKPLTDD
SDASTSAQRTEVSSVSSSQVGPT
>ggIodopsin
MAAWEAAFAARRRHEEEDTTRDSVFTYTNSNNTRGPFEGPNYHIAPRWVYNLTSLWMIFVVAASVFTNGLVLVATWKFKKL
RHPLNWILVNLAVADLGETVIASTISVINQISGYFILGHPMCVVEGYTVSACGITALWSLAIISWERWFVVCKPFGNIKFD
GKLAVAGILFSWLWSCAWTAPPIFGWSRYWPHGLKTSCGPDVFSGSSDPGVQSYMVVLMVTCCFFPLAIIILCYLQVSLAI
RAVAAQQKESESTQKAEKEVSRMVVVMIVAYCFCWGPYTFFACFAAANPGYAFHPLAAALPAYFAKSATIYNPIIYVFMNR
QFRNCILQLFGKKVDDGSEVSTSRTEVSSVSNSSVSPA
>ggGPR52
MNRSRWIEWRTLNMSGSVVNLSEHLSCPLGFGHYNAVDICILETVVIVLLTFLIIAGNLTVIFVFHCAPLLHHYTTSYFIQ
TMAYADLFVGVSCLVPTLSLLHYSTGVHESLTCQVFGYIISVLKSVSMACLACISVDRYLAITKPLSYNQLVTPCRLRICI
ILIWIYSCLIFLPSFFGWGKPGYHGDIFEWCATSWLTNAYFTGFIVCLLYAPAAFVICFTYFHIFKICRQHTKEINDRRAR
FPSHEVDAAGETGHSPDRRYAMVLFRITSVFYMLWLPYIIYFLLESSRVLENPALSFLTTWLAISNSFCNCVIYSLSNSVF
RLGLRRLSETMCSSCTCLKDRGVQDPKPRKRANSCSI
>ggOPSINn1
MDVFRALGNESLLSNSSGPARWDPFHHPLDSIQPWHFRLVAAVMFVVTSLSLAENLAVILVTFKFKQLRQPVNYVIVNLSV
ADFLVSLTGGTISFLANLKGYFYMGHWACVLEGFAVTFFGIVALWSLALLAFERYIVICRPVGNMRLRGKHAAQGIAFVWT
FSFIWTIPPTMGWSSYTTXVSNTQGRLRTARKPERQVTRMVVVMIIAFLICWMPYAVFSILATAYPSIELDPHLAAIPAFF
SKTATVYNPIIYVFMNKQ
>ggADORA1
MAQSVTAFQAAYISIEVLIALVSVPGNILVIWAVKMNQALRDATFCFIVSLAVADVAVGALVIPLAIIINIGPQTEFYSCL
MMACPVLILTESSILALLAIAVDRYLRVKIPLRYKSVVTPRRAAVAIACCWIVSFLVGLTPMFGWNNLNKVLGTRDLNVSH
SEFVIKCQFETVISMEYMVYFNFFVWVLPPLLLMLLIYLEVFNLIRTQLNKKVSSSSNDPQKYYGKELKIAKSLALVLFLF
ALSWLPLHILNCITLFCPSCKTPHILTYIAIFLTHGNSAMNPIVYAFRIKKFRTAFLQIWNQYFCCKTNKSSSSSTAETVN
>ggADORA2Bb
mlvhgkedflsdiayiileliiavlailgnvlvcwavylnsnlqnvtnyfvvslaaadiavgvlaipfaitistgfcaffy
gclfiacfvlvltqssifsllaiaidriiairiplrynglvtgsrakgiiaicwvlsfiigltpmlgwhkrsqieevgank
sspincsnsmvaclfeavvtmeymvyynffacvllplllmfgiylkifmaarrqlkqmenkmvhgersrstlqkevhaaks
laiivglfavcwlplhiincftlfcpncahaplwlmylaiilshansvvnpliyayrirefrytfrkiisqhilgrkeqfk
agtasirtsthggdeenasiriseyalevytngeihrdpekqdlnkckavlewhqngnaldmetnghlprsckngilsdac
vnrelhheelidaqvsfsdlettalatadvs
>ggADORA2Ba
MNTMKTTYIVLELIIAVLSIAGNVLVCWAVAINSTLKNATNYFLVSLAVADIAVGLLAIPFAITISIGFQVDFHSCLFFAC
FVLVLTQSSIFSLLAVAIDRYLAIKIPLRSSFSWRSSKYLTDNPQSFGWNQKTYFEVDENDLGLQPEDTCHFHNKSADFQW
LFDKRRLVMNSVPQGSVLRPVLFNTFINDIDDGIECILSKFADNTKLSDVVDTVKGRDAIQRDLNRLKGQQNVVEYWWEGS
ISTARPPTSTFDVMGQHNKTGGITFGAALIFHTELKGCAASKNFTVKIITVYWITDFGHKIFHIHTPEDIVLIEFVNQFVY
I
>ggADORA3
MPASSSPINVTFNTSQVMGSIDVIYISAECLVALLAALGNIPVVWAVKLNAAFHNTTMYFIASLALADIAVGVFVVPLAVL
VSLQVSIPFHFCLFLCCLMVVFTQASILSLLAIAIDRYLRVKLPIRYKIISTERRIWGALGLCWSLSLLVGLTPMFGWNKQ
RSAPYHTCGFTSVIRMDYMVYFSFFAWTLIPLSIMCALYVAVFYIIRTKLSQGATGARGAGTFYGKEFRKAKSLALVLFLF
AVSWLPLCIMNCVLYFHPEYKIPKPWIFLGILLSHANSPMNPLVYPCKIKKFKTTYLLILRTYILCRKKPQAMPSSYRLNT
PAVVAQNER
>ggADRA1A
MVFLPGNSSNCSNCTYSAEPVNISKAILLGVILGGLIIFGVLGNILVILSVACHRHLQSVTHYYIINLAVADLLLTSTVLP
FSATMEILGYWAFGRIFCNIWAAVDVLCCTASIMSLCIISIDRYIGVSYPLRYPSIVTEKRGLLALLCVWALSLVISIGPL
FGWKEPAPEDETICQITEEPGYVLFSALGSFYLPLTIILVMYCRVYVVAKRENKGLRSGLKMEKSRSEAVTLRIHRKNVPE
NSGATASSKSKHHFSVRLLKFSREKKAAKTLGIVVGCFVLCWLPFFVVMPLVSFFPTIKPPDTLFKITFWLGYLNSCINPI
IYPCSSQEFKKAFQNVLRAQCLPRKHAAKKQSPSFNLNHPSNQSMGGGKGVVRIPVGSGETFYKISKSDGVCEWKIFSAVQ
SVPTKSAIPKDKSSCTAAKVKSKGFLQECCCAGTSGNTVHENCKVPTIKIHTMSLSESGEDV
>ggADRA1D
MTFPELSNGSLSENRTGQGSQSGANRSWGLQGEESPEGNGTTLPFALDVQAVGVGVFLAVFILSAIVGNILVILSVACNRH
LQTVTNYFIVNLAIADLLLSTTVLPFSATLDALGFWVFGRIFCNIWAAVDVLCCSASIMSLCIISVDRYIGVQYSLKYPTI
MTERKAGVILVVVWLSSMVISIGPLLGWKEPPPPDESICSITEEPGYALFSSLFSFYLPLMVILVMYFRIYVVARRTTRSL
EAGVKKERNKSMEVVLRIHCRSVLEEPLSSTQSKGHNFRSSLSVRLLKFSREKKAAKTLAIVVGVFILCWFPFFFVLPGSF
FPSLKPSEMVFKVIFWLGYFNSCVNPIIYPCSSKEFKRAFIRLLKCQCHRRRRPIWRVYDHHWRGASVNSSVQDSETDLRP
RISTLNSSFIFNSSLQERAGKRRTLSFKGWKMLTPFQKPASAQLKEKMNSLSNKIRGGSSKGGVTATYKTEVESVSIGIPH
DFMETIDYQTYDLTDCCGLRETDI
```

>ggADRA2A
GLLMLFTVFGNVLVIIAVFTSRALKAPQNLFLVSLASADILVATLVIPFSLANEVMGYWYFGKVWCEIYLALDVLFCTSSI
VHLCAISLDRYWSITQAIEYNLKRTPRRIKCIIFIVWVISAVISFPPLISIEKKSGQQVDQWAAGCKINDEKWYIISSSIG
SFFTPCLIMILVYVRIYQIAKRRTRVPLNKRPERPEKKQNGLADKEDLPASAQLNGEKAAGAGDGQEGEVNGIDMEETSSS
EHQENNQPKKSERPLRGKTKTKLSQIKPGDTLPRKTEEERNTKGSRWRGRQNREKRFTFVLAVVIGVFVICWFPFFFTYTL
TAVCKSCSVPPTLFKFFFWFGYCNSSLNPVIYTIFNHDFRRAFKRILCLIERKRIV
>ggADRA2C
DSVLAALLQPPSPYSPAAVASLAALVGFIIVFTIVGNVLVVIAVLTSRALRAPQNLFLVSLASADILVATLVMPFSLANEL
MNYWYFGKVWCNIYLALDVLFCTSSIVHLCAISLDRYWSVTQAVEYNLKRTPRRIKAIIVTVWLISAVISFPPLISMYRDP
EGDVFPQCKLNDETWYILSSCIVSFFAPCLIMVLVYIRIYRVAKLRTRTLSEKRTMPEGSSQTENGLSRAAGGCTSLRMQL
GENGHYSVHHWRKASELEDIELEESSTSESRRRRSREEHPRKSSKSQSFSYSYSSKHSSSRLSRSSNRSMEFFSYRRRRKR
SSICRKKVTQAREKRFTFVLAVVMGVFVVCWFPFFFSYSLYGICREACEVPETLFKFFFWIGYCNSSLNPVIYTIFNQDFR
RSFKHILFKKKKKNFRH
>ggADRB1
MGDGWLPPDCGPHNRSGGGGATAAPTGNRQVSAELLSQQWEAGMSLLMALVVLLIVAGNVLVIAAIGRTQRLQTLTNLFIT
SLACADLVMGLLVVPFGATLVVRGTWLWGSFLCECWTSVDVLCVTASIETLCVIAIDRYLAITSPFRYQSLMTRARAKGII
CTVWAISALVSFLPIMMHWWRDEDPQALKCYQDPGCCDFVTNRAYAIASSIISFYIPLLIMIFVYLRVYREAKEQIRKIDR
CEGRFYGSQEQPQPPPLPHQQPILGNGRASKRKTSRVMAMREHKALKTLGIIMGVFTLCWLPFFLVNVVNVFNRDLVPDWL
FVFFNWLGYANSAFNPIIYCRSPDFRKAFKRLLCFPRKADRRLHAGGQPAPLPGGFISTLGSPEHSPGGMWSDCNGGMQGG
SESSLEERHSKTSRSESKLAARSFMLLAACFALAPSRALPRQEPCPVLLGTMLNAVSASCKHQSNGKYPDLEASNLKGKAE
VHRGFCRCCR
>ggADRB3
MTPLPTGNGTAWGAAAPGTVPNCSWAAILSRQWAVGAALSITILVIVAGNLLVIVAIAKTPRLQTMTNVFVTSLACADLIM
GLLVVPPGATILLSGHWPYGTVVCELWTSLDVLCVTASIETLCAIAVDRYLAITAPLQYEALVTKGRAWAVVCMVWAISAF
ISFLPIMNHWWRDGADEQAVRCYDDPRCCDFVTNMTYAIVSSTISFYVPLLVMIFVYVRVFAVATRHVQLIGKDKVRFLQE
TPSLSSRAGRRRRPSRLLAIKEHKALKTLGIIMGTFTLCWLP
>ggCHRM2
MNNSTYINSSSENVIALESPYKTIEVVFIVLVAGSLSFVTIIGNILVMVSIKVNRHLQTVNNYFLFSLACADLIIGIFSMN
LYTLYTVIGYWPLGPVVCDLWLALDYVVSNASVMNLLIISFDRYFCVTKPLTYPVKRTTKMAGMMIAAAWVLSFILWAPAI
LFWQFIVGGRTVPDKDCYIQFFSNPAVTFGTAIAAFYLPVIIMTVLYWQISRASKSRIKKGKKEAAQNQDPVSPSLVQGKI
VKPNNNNIPTSSDGLEHNKVQNGKTTGESVMENCVQGEEKDSSNDSTSVSVVPSNTKEDEAAKDASQISASQDHLKVENSK
LTCIRIVTKSQKGDCCAPTNTTVEIVGTNGDEKQNSVARKIVKMTKQPAKKKPPPSREKKVTRTILAILLAFIITWTPYNV
MVLINSFCASCIPGTVWTIGYWLCYINSTINPACYALCNATFKKTFKHLLMCHYKNIGATR
>ggCHRM3
MLTHYQLCFQKRSSQNYTVPDPTSCFDVPPWTILCQRATMIMHNNSSALPLFSNVSSFWKRDSHGPGLLDEGASLIGSYDS
PETTESFPFSTVETTNSSLNATIKDPLGGHAVWQVVLIAFLTGIIALVTIIGNILVIVSFKVNKQLKTVNNYFLLSLACAD
LIIGVISMNLFTTYIIMGHWALGNLACDLWLSIDYVASNASVMNLLVISFDRYFSITRPLTYRAKRTTKRAGVMIGLAWII
SFVLWAPAILFWQYFVGKRTVPLDECFIQFLSEPIITFGTAIAAFYLPVTIMSILYWRIYKETEKRTKELAGLQASGSEAE
TARFVHQTGSSRSLSSYELQRQSTKRSSRRKYRRCHFWLTMKSWEPNTDQGDQEHSSSDSWNNNDAAASLENSASSDEEDI
TAETRAIYSIVLKLPGHSAILNSTKLPSSEDLNESADELQKSDTDSQEKKPKKLQPPKSIQDGGSFQKSFSKLPIQPGSAE
TATASDGISSVTKTSAALPLSFKEATLAKKFALKTRSQITKRKRMSLIKEKKAAQTLSAILFAFIITWTPYNIMVLVNTFC
DCVPKTVWNLGYWLCYINSTVNPVCYALCNKMFRNTFKMLLLCQCDKRKRRKQQYQQRQSVIFHKRIPREAS
>ggCHRM4
MHNLSAQPWQAKMANLTYDNVTLSNRSEVAIQPPTNYKTVELVFIATVTGSLSLVTVVGNILVMLSIKVNRQLQTVNNYFL
FSLACADLIIGVFSMNLYTVYIIKGYWPLGAVVCDLWLALDYVVSNASVMNLLIISFDRYFCVTKPLTYPARRTTKMAGLM
IAAAWILSFILWAPAILFWQFIVGKRTVHERECYIQFLSNPAVTFGTAIAAFYLPVVIMTVLYIHISLASRSRVRRHKPES
RKERKGKSLSFFKAPPVKQNNNNSPKRAVEVKEEVRNGKVDDQPSAQTEATGQQEEKETSNESSTVSMTQTTKDKPTTEIL
PAGQGQSPAHPRVNPTSKWSKIKIVTKQTGTESVTAIEIVPAKAGASDHNSLSNSRPANVARKFASIARSQVRKKRQMAAR
EKKVTRTIFAILLAFILTWTPYNVMVLINTFCETCVPETVWSIGYWLCYVNSTINPACYALCNATFKKTFKHLLMCQYRNI
GTAR
>ggCHRM5
MEVNLFSNSTVTNSSSINHKQLEGHSLWEVITIAAVTAVVSLITIVGNVLVMISFKVNSQLKTVNNYYLLSLACADLIIGI
FSMNLYTSYILIGHWSLGSLACDLWLALDYVASNASVMNLLVISFDRYFSITRPLTYRAKRTPKRAGIMIGLAWLISFILW
APVILCWQYFVGERTVPPEECQIQFLYEPTITFGTAIAAFYIPVSVMTILYCRIYKETEKRTKDLAELQGSESVAEFETIK
PRKAFLKSCFSCKQQNLVKRERCQASWSSSSRSTSATVKASQAASTCTEWAKADQLTTCSSYASSEEEDKLATDPVFQVAY
RSPSKGKEEEFNERESQDIVVKEQPEENDFETQQYFLSPAHRPKSKKCVAYKFRLVVKADGTQEANNGCRKVKITPCSAAL
SKDPSIKSMDPNINNQTTKRKRMVLIKERKAAQTLSAILLAFIITWTPYNIMVLVSTFCSDCIPLTWWHLGYWLCYVNSTV
NPMCYALCNKTFRKTFKMLLFCQWKKKKVEEKLYWQGNTRMP
>ggCNR1
MKSILDGLADTTFRTITTDLLYVGSNDIQYEDMKGDMASKLGYYPQKFPLSSFRGDPFQEKMTAGDDPLLSIIPSDQINIT
EFYNKSLSTFKENEENIQCGENFMDMECFMILNPSQQLAIAVLSLTLGTFTVLENLLVLCVILHSRSLRCRPSYHFIGSLA
VADLLGSVIFVYSFVDFHVFHRKDSPNVFLFKLGGVTASFTASVGSLFLTAIDRYISIHRPLAYKRIVTRPKAVVAFCVMW
TIAIVIAVLPLLGWNCKKLNSVCSDIFPLIDETYLMFWIGVTSVLLLFIVYAYMYILWKAHSHAVRMLQRGTQKSIIIQST
EDGKVQITRPDQTRMDIRLAKTLVLILVVLIICWGPLLAIMVYDVFGKMNKLIKTVFAFCSMLCLLNSTVNPIIYALRSKD
LRHAFRSMFPTCEGTAQPLDNSMESDCQHKHANNAGNVHRAAESCIKSTVKIAKVTMSVSTDTTAEAL
>ggCNR2
MINCKMYENASKCNVSSKSMECFMVLSTQAQKISTATLCGLFGTLGIFENCLVLYLIFSSPGTRRKPSYIFISSLALADLL
ASIIFVRSFVNFHVFNETDSSKELFLLKLGGVNTSFMASLSSLLLMALDRYISISYPSKYKLLVTRKRAWTALGVLWVTCV

ATAFLPLMGWNCCTLNSACSELFPFVDNNYLSTWICLVMVLLGCIVCAYARMLWRAHQHVAYMGKHQVHVGKQNTRMRMDV
MLAKTLVMVLTVLVLCWLPALILMIYSIFVNLNNHLRKVFAFCSTLCLLNSMVNPIIYALRSKELYSSLRMRLSQCRRQLR
TSNDSPEAESVHRSSMIETVCEDTHVL
>ggDRD1D
MASMAGGPRALRALTGCLLGALVLGTLAGNALVCLAVLRFRHLRTKVTNWFVLSLAISDLCVAILVMPWKAVTEVAGGFWL
FGSRFCDTWVAFDIMCSTASILHLCIISLDRYWAIASPFRYERRMTQRLACAMIAMAWALSILISFVPVQLHWHKAKDRRH
PSSSCDVSLNRTYAITSSLISFYIPVAIMIVTYTRIYRIAQAQIRRISTLERAGGQWPAHSKEPASTLRSSLRKETKVLQT
LSIIMGVFVCCWLPFFLLNCLLPFCQPESDSNGQSPCVGQTTFNVFVWFGWANSSVNPVIYAFNADFRRAFSNLLGCQSLC
CSTPRAAVERVNLSNELVSYHQDTTCQKDGATAAIPPTTVTAWVQPEPHVVSPQQEDREELPTEERPVAAPTQAMPGSSPK
CCHVTATSQLDCGADLSLETINAFTGFSRHEGHHPIPEG
>ggDRD1A
MTWNDTTMDGEGLLVERDSSFRILTGCFLSLLILSTLLGNTLVCAAVIRFRHLRSKVTNFFVISLAVSDLLVAVLVMPWKA
VSEIAGFWPFGSFCNIWVAFDIMCSTASILNLCVISVDRYWAISSPFRYERKMTPKAAFIMISVAWTLSVLISFIPVQLNW
HKATTTSFLDLNASLQGISMDNCDSSLNRMYAISSSLISFYIPVAIMIVTYTRIYRIAQKQIRRISALERAAVHAKNCQNT
SGNRSSMDCQQPESNFKMSFKRETKVLKTLSVIMGVFVCCWLPFFVLNCMIPFCEPTQPSKGAEAFCINSTTFDVFIWFGW
ANSSLNPIIYAFNADFRKAFSTLLGCYRLCPMSGNAIETVSINNNGAVFSSQHEPKGSSPKESNLVYLIPHAIICPEEEPL
KKEEEGELSKTLEKMSPALSGMLDYEADVSLEKITPITQNGQHKT
>ggDRD4
XLSVCTERALKTTTNYFIVSLAVADLLLALLVLPLYVYSEFQGGVWSLSTVLCDALMTMDVMLCTASIFNLCAISVDRFIA
VSVPLNYNRRQIDLRQLILISTTWIFAFAVASPVIFGLNNVPNRDPSLCQLEDDNYIVYSSICSFFIPCPVMLVLYCAMFQ
GLKRWEEARKAKLRGGIYGGNRMLYHPSPFIERERVGMEPEEYHPYAHPEHPLSGDYVMSNGLQTVSYPHLKYPHPAHGQK
RAKINGRERKAMRVLPVVVGAFLFCWTPFFVVHITRALCKSCTIPTQVTSIVTWLGYVNSAVNPIIYTVFNAEFRNFFRKV
LHLFC
>ggDRD5
XGGSSLLPPPAGPPGGARGQAGAPGAAQVAAGSLLALLILWTLFGNVLVCAAIVRYRHLRSKVTNIFIVSLAVSDLLVAVL
VMPWKAVAEVAGYWPFGAFCNVWVAFDIMCSTASILNLCVISVDRYWAISSPFRYERKMTQRLALVMIGVAWALSVLISFI
PVQLNWHRGGDAATAAAAGDIEDGFGTGWEAAGAFTTWAEDMSTTWVALAAMTPSEGTSGSNNTVPGPSESCDSSLNRTYA
ISSSLISFYIPVAIMIVTYTRIYRIAQVQIRRISSLERAAEHAQSCRSNHVDCHHHTSLKSSIRKETKVLKTLSIIMGVFV
CCWLPFFILNCMVPFCESPPSDPRAGLPCVSETTFNIFVWFGWANSSLNPIIYAFNADFRKVFSNLLGCGQFCSSTPVETV
NISNELISYNQDTLFHKEIVTAYVNMIPNVVDCEENREDPFDRMSQISPDPEVATDSVCELDCEGEISLGKITPFTPNGLH
>ggEDG1
MSSGTTAPVRVVSSLTNTDVNYVIKEHYNYTGKLNENADSGIKVTSVVFIIICCFIILENIFVLLTIWKTKKFHRPMYYFI
GNLALSDLLAGVAYTANLLLSGHKTYSLTPSQWFVREGSMFVALSASVFSLLAIAIERYITMLKMKLHNGSNSFRSFLLIS
ACWVISVILGGLPIMGWNCISLLSNCSTVLPLYHKHYILFCTTVFTGLLLSIVVLYCRIYSMVRTRSRRLTFRKNITKATR
SSEKSLALLKTVIIVLSAFIACWAPLFILLLLDVGCRVKTCPILYKAEYFLVLAVLNSATNPIIYTLTNKEMRRAFIKILC
CCKCPPTDSGTKFKRPIIGGMEFSRSKSDNSSHPQKEEGDRPETIMSSGNVTSSS
>ggEDG2
MSEPQCYYNETIAFFYNRSGKYLATEWNTVSKLVMGLGITVCIFIMLANLLVMVAIYVNRRFHFPIYYLMANLAAADFFAG
LAYFYLMFNTGPNTRRLTVSTWLLRQGLIDTSLTASVANLLAIAIERHITVFRMQLHTRMSNRRVVVVIVVIWTMAIVMGA
IPSVGWNCICDITHCSNMAPLYSDSYLVFWAIFNLVTFVVMVVLYAHIFGYVRQRTMRMSRHSSGPRRNRDTMMSLLKTVV
IVLGAFIICWTPGLVLLLLDVCCPQCNVLAYEKFFLLLAEFNSAMNPIIYSYRDKEMSATFKQILCCQRSESTNGPTEGSD
RSASSLNHTILAGVHSNDHSVV
>ggEDG3
MSTVTVIPAAVVGTEGNEEMDSLIVLHYNYTGKLILREKATDNIDLTTVAFLILCSFIVLENLMVLIAIWKNNKFHNRMYF
FIGNLALCDLLAGIVYKVNILMSGKKTLSLSSTIWFIREGSMFVALGASTFSLLAIAIERHLTMIKMRPYDANKKYRVFLL
IGTCWLISISLGALPILGWNCINNLPDCSTILPLYSKKYVVFCISIFIAILVAIVILYARIYILVKSSSRNVTNHNNSERS
MALLRTVVIVVSVFIACWSPLFILFLIDVACQVRECSVLFKANWFIALAVINSAMNPIIYTLASKEMRRAFFRLVCGCLVR
SRAARSMPIQPTPDHSRSKSSSSNTQKPKDDFPQMSIPSCVIEKREPSFCNGSFCK
>ggEDG4
MKHCFYNESVGFFYNNSRKELTTYWRTKDILVVALGLTVSLIVLLTNLLVIAAIIINRRFHYPIYYLLGNLAAADLFAGIA
YMFLMFHTGPRTAELSLKTWFIRQSLLDTSLTASVVNLLAIAVERHQTVLTMQLHSKMSNQRVMILIFCIWATALLLGLIP
SYGWHCLCAX
>ggEDG6
MDLFNTQGSCLQLAAMQNVNIILQHYNFSGKLTNRHSRDEGMGLTRTIFVIISCAIILENLLVLLAVLRCLRARRWVYSCI
ASITVSDLLAGIAYLSNLCLSGKKTFQLSPQLWFLREGILFVALAASTFSLLVTAIERYSAMVRPIAENEASKTLRLRGLI
VFCWLLAFVIGLLPLLGWNCLCDFSACSVLLPLYSKNYILFSVVMFSIILLGIIGLYISIFLLVQASSRQTTTRHSRKRSL
RLLKTVLMILGAFIICWSPLFALMLFDVFCETHTCRLLHSLDWTLALAMINSGINPIIYSLRSVEVRHAVCSLLCCCCIRI
GLCKPGNCLVITDINSGSSTESSLRYRESFRSPIAPIPRPRAPLSSNSSMMSNLPSL
>ggEDG7
MNECYYDKHMDFFYNKTNTHTADEWTGPPLIGVLCFGTFFCLFIFISNSLVIAAVVKNKRFHFPFYYLLANLAAADFFAGI
AYVFLMFHTGPVSKTLTVNRWFLRQGLLDTSLTASLVNLLVIAVERHMSIMRMKIHSNLTKKRVTFLIISIWAIAIFMGAV
PTLGWNCLCDISACSSLAPIYSRSYLVFWSVLNLVVFFIMVVVYIRIYMYVQRKTNVLSSHTSGSISRRRTPVKLMKTVMT
LLGAFVVCWTPGLVVLLLDGLNCTNCGIQNVKRWFLLLALLNSVMNPVIYSYKDDEMWGTMKRMLCCSSDDRNQERRSSRI
PSTVLGRSTDTTGQYIEDSIIQGTICGKGDLGEKGNS
>ggGPR12
MNEEPTVNASWLPQDRVEASSTENASGSSLVPVVEPEPELWVNPWDIVLCTSGTLISCENAVVVLIIFHNPSLRAPMFLLI
GSLALADLLAGVGLIVNFVFAYLLRSEATKLVTVGLIVASFSASVGSLLAITVDRYLSLYYALTYNSERTVTFTYVMLILL
WGAAICIGLLPVMGWNCLRDESTCSVIRPLTKNNAAVLSVSFLLMFALMLQLYIQICKIVMRHAHQIALQHHFLATSHYVT

TRKGVSTLAIILGTFAACWMPFTLYSLIADYTYPSIYTYATLLPATYNSIINPVIYAFRNQEIQKALWLVCCGCVPSNLSQ
RARSPSDV
>ggGPR162
MGDSESESTLHNNSLWWLACGMLALLANSWIILSITAKQQKHKPLELLLCFLAGTHILMAAVPLTTYAVVQLRRESSNYDW
NESICKVFVSTYYTLALATCFTVASLSYHRMWMVRWPVNYRLSNAKKQALHAVMGIWMVSFILSTLPSIGWHNNGERYYAR
GCQFIVSKIGLGFGVCFSLLLLGGIVMGLVCVGITFYQTLWAHRRRQCCHQRPEEASSCSSAHNTFNVPAIVVEDVRGKR
RSSLDGSESAKTSLQMTNLISAIVFLYDTLTGVPILVVSFFSLRYDTAPTWMVLAVLWCSMVQTLLLPSFIWSCERYRADL
RTIWEQCVAIMSEEDGDDDGVCDDYGDGRICKVRFDANGAAAVKRDSRDIKLLPMHHMLLPQDKVHYLQVPISRRMSHDET
NIFSAHRSAPSFLHKWSSSDDIRISTLRKPGGPGFLPPQLHDYQHRQQPPEDELTSLRQFLEGGLVPRGSSSSACFFRDEI
TTFIDETPQPTPVCSPRHSRLPIAACRDRRLSLGSREEESTNRPRCCSLAGSEAWHLQDEEQAPRERTPEACEAQPLQDPK
L
>ggGPR26n1
MDLAGVLLALLLVLVLVVSLLSNLLVLLCFVYSTEIRKQVAGVFLVNLSFCNLLLTILNMPFTLLGILRNQQPLGGCICKA
VGFLETFLTSNTMLSMAALSIDKWIAVVFPLSYTSKMRYKDAVILMGYSWLHSLTFPLVSLFYSWVDYNSVYASCTLHLKE
ETERRRFTVFTIVFHSTSFMLSLVILCFTYLKVLKVARFHCKRIDIITMQTLVLLVDIHPSVKQRCLNEQKRRRQRATKKI
SIFIGSFVICFGPYIITRRLIELLPFVTINYYWGIISKCLTYSKAASDPFVYSLLRQQYKKVLINIVNRILKRDLYPSSGY
NSSLDTENDYCLHRTN
>ggGPR26
MSIWEVILAFVVVVLMLVALLANVLVLMCFLYSADIRKQVPGLFILNLTFCNLLMTVSSMPLTLAGIIYKRQPGGDQICHV
VGFLETFLTTNSMLSMAALSIDRWIAVVFPLSYHSKMSFRDAALILSYTWLHSVSFPIVAASLSWVGFHHLYASCTLYNKR
PEDRTQFVIFTGVFHTLSFLLSLIVLCFTYLKVLKVARFHCKRIDVITMQTLVLLVDIHQSVRERCLEEQKRRRQRATKKI
STFIGTFILCFAPYVITRLVELSSTIPINSHWGIISKCLAYSKVVSDPFVYSLLRNQYKKTWKDIINKILKRSSINSSALT
SESHNRNILQLNE
>ggGPR6
MEPTAVLNESSAAVPWLPARAGNASLELASRPPAPTAINPWDVMLCVSGTAIACENAIVVAIICYSPTLRTPMFVLIGSLA
TADLLAGLGLILNFVFQYVIRSETVSLLTVGFLVASFAASVSSLLAITVDRYLSLYNALTYYSERTVLCIHTMLVCTWGVS
LCLGLLPVLGWNCLHDRAACSVVRPLTKSNVTLLSASFFLIFIIMLHLYIEICKIVCRHAHQIALQQHFLTASHYVATKKG
VSTLAIILGTFGASWLPFAIYCVVGDPDYPSVYTYATLLPATYNSMINPIIYAYRNQEIQRSMWVLFCGCFQSKVSFRSRS
PSDV
>ggGPR61
MLSGSAVFDSPVLGEAMCRVYLFLSVCFISMCILSISTINVERYYYVVHPMRYEVRMTVGLVVCVLVGVWLKAVATSLVPV
LGWLAPDRPPPAGRGCSLQWSRGPYCKFFVVFFAAFYFLLPLLIIVVVYCSMFKVARVAAMHHGPPPTWMETPRRRSESLS
SRSTMVTTSGAPRTTPQRTFGGGKAAAILLAVGGQFLFCWLPYFSFHLYTALSAQPLAGPAADTVVTWLGYFCFTSNPFFY
RCLNRHIRTELGRLLTCLFTPPPEEDLR
>ggGPR63
MVFSAMLTPTNSGTSNATFILYENAYMNFTTAQVLLRSGTTQPLKYSSGAVLTTERSTFLVNTTAVLPSQEAFRSLSLPLQ
IILSAAMIFIMLVSFLGNFVVCLMVYQKAAMRSAINILLASLAFADMLLAVLNMPFALITIITTHWIFGDIFCRVSAMFFW
LFVIEGVAILLIISIDRFLIIVQRQDKLTPYRAKILIVVSWAASFVVAFPLSVGNPNLQIPSRAPQCVFGYSTSPGYQVYV
IIILLISFFIPFLVMLYSFMGILNTVRHNAVRIHSHPDSICLSQASKLGLMSLQRPFQMNIDMSFKTRAFTTILILFLVFI
VCWAPFTTYSLIATFNSHFYYKHNFFEISTWLLWLCYLKSALNPLIYYWRIKKFHDACLDLMPKYFKFLPQLPGHTRRRIR
PSAIYVCGEHRSVV
>ggHRH2
MDPCSNLTDPSKTMDFPLQLLVGSCLALLITFTLCGNIIVCLAVTLDRRLRSLTNCIIVSLAITDLLLALLVLPFSAYYEL
TSEWPFSSTLCNIYLSLDVMLCTASILNLFIISLDRYFAVTTPLRYQQLVTRSRVAVGLVVIWTVSLMVSFLPIHLGWNTN
GTMVQNTTPNCTRECKLEVNSVYGLVDSLLTFYIPLVIMCITYYRIFKIAREQAKRINHTWCCSSNGPMPPMVKEHKATVT
LAVVMGAFIICWFPYFTVFTYRGVWGDSSVKGIPMSIVLWLGYANSALNPILYGTFNRDFRVAYQHLLQCWRTGDPKSSLQ
KGQPRGRSCGESLDGQEGKCLKLEMRNGKGTLLADGALQR
>ggHRH3
MLGAAAAGREGLHPGGAGGGAGPGRGGSPAAAERRWAMESGGELNGSAAASGRFATAGTAALGALMALLIAVTVAGNALVM
LAFVADSSLRTQNNFFLLNLAISDFLVGAFCIPLYVPYVLTGRWIFGRSLCKLWLVVDYLLCTSSVFNIVLISYDRFLSVT
RAVSYRAQQGNTRRAVLKMVLVWVLAFLLYGPAIISWEYISGESIIPTGECYAEFFYNWYFLMTASTLEFFTPFISVMFFN
LSIYLNIQKRTKLRLDVFHEVHNRSFAEEMEMSPEAKLSSKCYKWEQKEPAETLGLSKSKAQAAASSAAEDLLTATAGGSQ
CCNKKGCKNSASALSLEKRVKVVSQGVTQRFRLSRDKKVAKSLAIIVGIFGICWAPYTLLMIIRAGCHGHCISDYWYETSF
WLLWINSAVNPVLYPLCHYSFRRAFIKLLCPKKLKIQPHDPLQNCWK
>ggHRH3n1b
MHNSTAETPCMMGPCNGTVSPQPLTPQFPLGVLIFLALLMVLLALVTILGNVLVILAFILDKNLRHRNNYFFLNLAISDFA
VGVFCMPLYIPYALTGTWHLGKGLCKLWLLLDYLMCSASVFNIVLISYDRFLSVTKAVSYRTQQGMMSNPVVKMVAIWVFA
FLLYSPAVLLWEYMAGYSMVEEGQCYAEFFNNWYFLLCASTLEFFVPLISVTYFNLSIFYNIQRRQRRGSIQYCESPGSRS
MTWRFCFLLRRGSSLSETEDSASSVMRSLRPSLASNSTSPTSPVADKRDFSASFRANSQSRLQRDKKVAKSLAIIVCVFVI
CWAPYTLLMIIRGACQGKCVHPILYEITFWLLWINSSLNPFLYPLCHVRFQMAFMKILCPQKLTMLRSNDTPSV
>ggHRH3n1a
MLLCNGTVPPQPSTPQFSLGVLVLLALLMVLLALVTILGNVLVILAFILDKNLRHRNNYFFLNLAISDFAVGAFCIPLYIP
YALTGIWHFGRGMCKLWLLIDYLMCTASVFNIVIISYDRFLSVTKAVSYRTQQGIMSNPVVKMVAIWVFAFLLYPPAVLLW
EYMAGCTVVREGQCYAEFFHNWYFLLCASTLEFFVPLISVTYFNLSIFYNIQRRQRRGSTQYCEPPGSSSVTWRFCFLLRR
GPSLSETEDSASSSMRIKKESLVTESSSPSRANSVTPENDFSVSARTRSKLYHDKKIAKSLAIIVCVFAICWAPYTLVMII
RGACQSECVHSVLYQITFWLLWINSSLNPFLYPLCHDKFRMAFMKILCPKKFALLRSASC
>ggHTR1A
MDVANNTTSPERSPEGEGGPGLAEVTLGYQLLTSLLLGTLILCAVSGNACVIAAIALERSLQTVANYLIGSLAVTDLMVSV

LVLPMAALYQVLNKWTLGQVICDIFISLDVLCCTSSILHLCAIALDRYWAITDPIDYVNKRTPRRAAVLISLTWLIGFLIS
IPPMLGWRTPEDRSNPDACTISKDHGYTIYSTFGAFYIPLLLMLVLYGRIFKAARFRIRKTVKKAEKKKIADTCLTLSPAA
LQKKSNGEPGKGWRRTVEPRSGACVNGAVRQGEDGAALEVIEVQRCSSSSKTHLPLPSEACGSPPPPSFERRNEKNTEAKR
RMALSREKKTVKTLGIIMGTFILCWLPFFIVALVLPFCDSKCYMPEWLGAVINWLGYSNSLLNPIIYAYFNKDFQSAFKKI
IKCKFCRQ
>ggHTR1B
MEPASPCPAPLLPANDSYHGRNCSAEEGIYQDATPLSGKIVLAVVLALVTLATVLSNAFVIATVYQTRKLHTPANYLIASL
AFTDLLVSILVMPISTLYTVTGKWTLGQVVCDIWLSSDITCCTASILHLCVIALDRYWAITDAVEYSTKRTPKRAAGMIAL
VWVFSICISMPPLFWRQAKAEEASNCAVNTDHVLYTVYSTVGAFYFPTLLLIALYGRIYVEARSRILKQTPKKAGKRLTRA
QLITDSPGSSSSVTSINSKAPEGPSETGSPVYMNQVKVKVSDALLEKKKLTAARERKATKTLGIILGAFIVCWLPFFIISL
VLPICKDACWFHMAIFDFFTWLGYLNSLINPIIYTMSNEDFKQAFHKLIRFRCTG
>ggHTR2B
NSWATTIIKITIVWLISIGIAIPVPIRGIEDGNGNSTNITCLLTPDRFHDFILYGSVAAFFIPLAIMIVTYFLTIQVLRKK
AYLINKPPQRFTWSTVSTVFQRDATPACSPEKVAMLNGSRKDKTPTNDMPICRTSTIGRKSMQTITNEQRASKVLGIVFFL
FLLMWCPFFITNISSVLCNSCNKEVFQKLMEIFVWIGYVSSGVNPLVYTLFNKTFREAFSRYITCNYRTTKTSKVLRKCSS
RISFRNSVTENSKLFVMHGMRNGINPIM
>ggHTR2C
MSTLSSTGILLSLTTVSVTLDFSLHGGLMAWSLSTNLTLNQSLPTSDPLNASEKGEVSRMSVREKNWPALLILVVILLTIG
GNILVIMAVSLEKKLQNATNFFLMSLAVADMLVGILVMPVSLIAVLYDYAWPLPKQLCPIWISLDVLFSTASIMHLCAISL
DRYVAIRNPIEHSRFNSRTKAIMKIAAVWTISIGISMPIPVIGLQDDSRVFINGTCVLNDENFVLIGSFMAFFIPLIIMVI
TYCLTVQVLQRQATVFMCGEVPKQRRSSVNCLKKENNTEHISMLHNHEGASHLNSPVSKEAVLFRKGTMQSINNERRASKV
LGIVFFLFLIMWCPFFITNVMSVLCKEACDKDLLSELLDVFVWVGYVCSGVNPLVYTLFNKTYRRAFSSYIRCQYKTSKKS
ALRQNQCLNVASTALYGKDLTLTSYRNGNELNSMELDEAEEGLEMQPGTSELSINSCNVVSERVSCV
>ggHTR4
MPAYVQCHAQGEGTSFVCSHSVRSSEGFGVAEKIVLLTFISAVILMAILGNLLVMVAVCRDRQLRKIKTNYFIVSLAFADL
LVSVLVMPFGAIELVQDNWIYGEMFCLVRTSLDVLLTTASILHLCCISLDRYYAICCQPLVYRNKMTPLRIAVMLGGCWVI
PTFISFLPIMQGWNSIGIIDLIQQRQFNKASNSTYCIFMVNKPYAITCSVVAFYFPFLLMVLAYYRIYVTAREHARQIRVL
QRAGTPADSRHQPDQHSTHRMKTETKAAKTLCIIMGCFCLCWAPFFVTNIVDPFINYTVPGKLWTAFLWLGYINSGLNPFL
YAFLNKSFRRAFLIILCCGDERYRRPSILGQTVPCSTTTINGSTHVLSSVCPNCSSFAYNQLTGASPCANSTLVLIKGIKA
VTETVEFPRQSKAVGVGQPCAAAALPCSPSSCTQGPGIWTACRPQPGSTAPPRCSLISSPALLAEGYLLMSERCIELQLVN
VCLHYHLSPWNSGGRWRKE
>ggHTR5
MDRQLNISCLGGATPDASNRSGSSRGLEGGRAQLSVFSVLVLTLLAMLVVATFLWNGLVLATIFRVRTFHRVPHNLVASMA
ISDVMVAALVMPLSLVHELSGRRWWLGRSLCQVWISFDVLCCTASIWNVTAIALDRYWSITRHLEYTLRTRRRISNIMIAL
TWVLSAFISLAPLLFGWGETYSEDSEECQVSQEPSYTIFSTFGAFYLPLCVVLFVYWKIYKAAKFRIGSRKSNSITPVSPE
APEIKEAAQQPQMVFTVRHATVTFQTDGDTWREQKERRAALMVGILIGVFVLCWIPFFITELISPLCSCDIPPIWKSIFLW
LGYSNSFFNPLIYTAFNKNYNNAFRNLFFRQH
>ggHTR6
MEGNLGSPNASVLGERSLLVGSSWVAAFLCFIILLTTAGNFLLILLIVTQRSLRNTSNYFLVSLFMSDLMVGLVVMPPAML
NQLYGHWVLRGDFCTLWYAFDVMCCSASILNLCVISLDRYLLIISPLKYKLRMTSCRALWLILATWTLAALASFLPIKLGW
HKLEFEVWSPNVTSRGDEEQCRLVVSLPYALVASCLTFFLPSAAISFTYCRILLAARKQAVQVASLASNVATATTDESMEQ
VPHAPSQPAAGSESRRFANKHSKKALKASLTLGILLGMFFVAWLPFFVTNVTQAVCDCVPAGFFDVLTWLGYCNSTMNPII
YPLFMRDFKRAMGKYLPCCRRTGEPRPSAISLSMKNSNSGPRAATSLKNVLTLQAETDSVVSEVVANEHRLLPAAEGALPA
PSAHLVGLFEMETSELELQRRQLGTPVA
>ggHTR7
XXPNPFMTEEPSVPAEPDLLTSNLTNATDCGDVILLYGDTEKIVIGAVLSIIILMTIAGNGLVIISVCIVKKLRQPSNYLV
VSLAAADLSVAFAVMPFVTITDLVGGEWLFGKVFCNVFIAMDVMCCTASIMTLCIISVDRYLGITRPLTYPVRQNGKLMAK
MVFIVWLLSASITLPPLFGWAKNVTVERVCLISQDFGYTVYSTGVAFYIPMAVMLVMYSRIYKAAKVSAEKHRFMNFSKHY
EEEGVYCLEASSRGHPSSKRTKAVEECATLSKLLRQDRKNISIFKREQKAARTLGIIVGAFTFCWFPFFLMSTARPFICGI
HCSCLPLRLERTLLWLGYTNSLINPLIYAFFNRDLRTTFWNLLRCRYRNINRRLSAASMHEALKATERHECIL.
>ggHTR7n1
XKKLRQPSNYLIVSLALADLSVALAVMPFVSVTDLIGGEWIFGRLFCNVFIAMDVMCCTASIMTLCVISIDRYLGITRPLT
YPVRQNGKCMAKMILCVWLLSASITIPPLFGWAQNVNDEKVCLISQDFGYTIYSTAVAFYIPMSVMLFMYYQIYKAAKRSA
AKHKFAGFPRPEEMEGISMNGVIKLHKESEECTNFSRLLKNDKKNISIFKREQKAATTLGIIVGAFTVCWLPFFLLSTARP
FICGTACSCIPLWVERTFLWLGYANSLINPFIYAFFNRDLRTTYRNLLQCRYRNINRKLSAAGMHEALKLAEKPEFVL
>ggMC1R
MSMLAPLRLLREPWNASEGNQSNATAGAGGAWCQGLDIPNELFLTLGLVSLVENLLVVAAILKNRNLHSPMYYFICCLAVS
DMLVSVSNLAKTLFMLLMEHGVLVIRASIVRHMDNVIDMLICSSIVSSLSFLGVIAVDRYITIFYALRYHSIMTLQRAVVT
MASVWLASTVSSTVLITYYRNNAILLCLIGFFLFMLVLMLVLYIHMFALARHHVRSISSQQKQPTIYRTSSLKGAVTLTIL
LGVFFICWGPFFFHLILIVTCPTNPFCTCFFSYFNLFLILIICNSVVDPLIYAFRSQELRRTLREVVLCSW
>ggMC2R
MSTEKPFNLILSAHAGQTSIPSLENITDFSLNITDCNQVVVPEEVFFTVAAAGILENLLVLVAVIRNKNLHLPMYFFICSL
AISDMLGSLYKTLENIFIILCKMGYLTRRGDFEKKLDDAMDSMFILSLLGSIFSLLAIAADRYITIFYALRYHNIMTLQRA
LVILAIIWTFCAGSSIAIALFSHEVATVIPFTILFPLMMIFILCLYIHMFLLARSHAKKIASLPTSAVHQRTNMKGAITLT
IFLGVFLCCWAPFVLHILLARFCPHNPYCACYMSIFHVNGTLIMCNAIIDPMIFAFRSPELRSTFKKMFCCARYNWNWWKL
NEGEYYRSTPMQHHFAELKILTQNDTTLAGNCR
>ggMC3R
MNSTHFTFSFQPVLLNVTEDISDSILNNRSSDGFCEQVFIKAEVFLTLGIISLMENILVILAVLKNGNLHSPMYFFLCSLA

VADMLVSTSNALETIMIAILSSGYLIIDDHFIQHMDNVFDSMICISLVASICNLLVIAIDRYITIFYALLYHSIMTVKKAL
TLIVLIWISCIICGIIFIAYSESKTVIVCLITMFFTMLFLMASLYVHMFLFARLHVKRIAALPVDGVPSQRTCMKGAITIT
ILLGVFIVCWAPFFLHLILIISCPMNPYCVCYTSHFNTYLVLIMCNSVIDPLIYAFRSLEMRKTFKEIVCCCYGVSVGQCM
L
>ggMC4R
MNFTQHRGTLQPLHFWNQSNGLHRGASEPSAKGHSSGGCYEQLFVSPEVFVTLGIISLLENVLVIVAIAKNKNLHSPMYFF
ICSLAVADMLVSVSNGSETIVITLLNNTDTDAQSFTINIDNVIDSVICSSLLASICSLLSIAVDRYFTIFYALQYHNIMTV
KRVGVIITCIWAACTVSGILFIIYSDSSVVIICLISMFFTMLILMASLYVHMFMMARMHIKKIAVLPGTGPIRQGANMKGA
ITLTILIGVFVVCWAPFFLHLIFYISCPYNPYCVCFMSHFNFYLILIMCNSIIDPLIYAFRSQELRKTFKEIICCCNLRGL
CDLPGKY
>ggMC5R
MNTSSQLYVSELNLSAFGSNFTVPTVKSKSSPCEQVVIAAEVFLILGIVSLLENILVICAIVKNKNLHSPMYFFVCSLAVA
DMLVSVSNAWETITIYLINNRHIIMEDAFVRHIDNVFDSLICISVVASMCSLLAIAVDRYITIFYALRYHNIMTVKRSGLI
IACIWTFCTGCGIIFILYYESTYVIICLITMFFTMLFLMVSLYIHMFLLARTHVKKIAALPGYNSVHQRTSMKGAITLTML
LGIFIVCWAPFFLHLILMISCPQNLYCVCFMSHFNMYLILIMCNSVIDPLIYAFRSQEMRKTFKEIICCYSVRMVCGLSNK
Y
>ggMTN1C
MERPGSNGSCSGCRLEGGPAARAASGLAAVLIVTIVVDVLGNALVILSVLRNKKLRNAGNIFVVSLSVADLVVAVYPYPLI
LSAIFHNGWTMGNIHCQISGFLMGLSVIGSIFNITAIAINRYCYICHSLRYDKLFNLKNTCCYICLTWTLTVVAIVPNFFV
GSLQYDPRIYSCTFAQTVSTSYTITVVVVHFIVPLSIVTFCYLRIWILVIQVKHRVRQDCKQKIRAADIRNFLTMFVVFVL
FAVCWGPLNFIGLAVSINPSKVQPHIPEWLFVLSYFMAYFNSCLNAVIYGLLNQNFRKEYKRILLMLRTPRLLFIDVSKGG
TEGLKSKPSPAVTNNNQAEIHL
>ggMTN1A
MRANGSELNGTVLPRDPPAEGSPRRPPWVTSTLATILIFTIVVDLLGNLLVILSVYRNKKLRNAGNIFVVSLAIADLVVAI
YPYPLVLTSVFHNGWNLGYLHCQISGFLMGLSVIGSIFNITGIAINRYCYICHSLKYDKLYSDKNSLCYVGLIWVLTVVAI
VPNLFVGSLQYDPRIYSCTFAQSVSSAYTIAVVFFHFILPIAIVTYCYLRIWILVIQVRRRVKPDNNPRLKPHDFRNFVTM
FVVFVLFAVCWAPLNFIGLAVAVDPETIIPRIPEWLFVSSYYMAYFNSCLNAIIYGLLNQNFRREYKKIVVSFCTAKAFFQ
DSSNDAADRIRSKPSPLITNNNQVKVDSV
>ggMTN1B
GNAFVVSLALADLVVALYPYPLVLLAIFHNGWTLGEMHCKVSGFVMGLSVIGSIFNITAIAINRYCYICHSFAYDKVYSCW
NTMLYVSLIWVLTVIATVPNFFVGSLKYDPRIYSCTFVQTASSYYTIAVVVIHFIVPITVVSFCYLRIWVLVLQVRRRVKS
ETKPRLKPSDFRNFLTMFVVFVIFAFCWAPLNFIGLAVAINPSEMAPKVPEWLFIISYFMAYFNSCLNAIIYGLLNQNFRN
EYKRILMSLWMPRLFFQDTSKGGTDGQKSKPSPALNNNDQMKTDTL
>ggOPN3
MHSGNGTGATSRPQLAAAGHEVPGERPLFSAGTYELLALLIATIGTLGVCNNLLVLVLYYKFKRLRTPTNLFLVNISLSDL
LVSVCGVSLTFMSCLRSRWVWDAAGCVWDGFSNSLFGIVSIMTLTVLAYERYIRVVHAKVIDFSWSWRAITYIWLYSLAWT
GAPLLGWNRYTLEIHGLGCSMDWKSKDPNDTSFVLLFFLGCLVAPVVIMAYCYGHILYAVRMLRCVEDFQTSQVIKLLKYE
KKVAKMCFLMISTFLICWMPYAVVSLLVTYGYSNLVTPTVAIIPSFFAKSSTAYNPVIYIFMSRKFRQCLLQLLCFRLMRF
QRIMKEPSGAGNVKPIRPIVMSQKVGDRPKKKVTFSSSSIIFIIASDDTQQIDDNSKHNGTKVNVIQVKPL
>ggOPN4
MDLNMGTQPHSVTKSEIPDHVLYTVGTCVLVIGSIGIIGNLLVLYAFYSNKKLRTPQNFFIMNLAVSDFLMSASQAPICFV
NSLHREWILGDIGCDLYAFCGALFGITSMMTLLAISVDRYLVITKPLRSIQWTSKKRTIQIIAAVWLYSLGWSVAPLLGWS
SYVPEGLMISCTWDYVTYSPANRSYTMILCCCVFFIPLIIILHCYLFMFLAIRSTGRDVQKLGSCSRKSFLSQSMKNEWKL
AKIAFVVIIVYVLSWSPYACVTLIAWAGRGNTLTPYSKSVPAVIAKASAIYNPIIYAIIHPRYRKTIHNAVPCLRFLIRIS
KNDLLRGSINESSFRTSLSSHQSLAGRTKNTCVSSVSTGEANWSDVELDTVEPAHEKLQPRRSHSFSSSLRQKRDLLPDSY
SCSEETEEKVSLSSSYLEKVLGRSAFPSSPVALVTSSLRAASLPVGLNSSSASRGAGSDISQMKTEESHNNGGLDSIVSNT
VPQIIIIPTSETNLFQEEPEEEETELFHFHDKKNNLLDLEGLSSSTEFLEAVEKFLS
>ggOPNn1
MTVKDVRGAFPTVDVPDHAHYTIGTVILIVGITGTLGNFLVIYAFCRSRTLQKPANIFIINLAVSDFLMSITQSPVFFTNS
LHKRWIFGEKGCELYAFCGALFGITSMITLMVIALDRYFVITKPLASVRVMSKKKALIILVGVWLYSLAWSLPPFFGWSAY
VPEGLLTSCSWDYMTFTPSVRAYTMLLFCFVFFIPLIAIIYSYVFIFEAIKKANKSVQTFGCKHGNRELQKQYHRMKNEWK
LAKIALIVILLYVISWSPYSVVALVAFAGYSHVLTPFMNSVPAVIAKASAIHNPIIYAITHPKYRTAIATYVPCLGFLLRV
SPKESRSFSSYPSSRRTTITSQSSETSGLQKGKRRLSSISDSESVRKTLPIHIFNCRVIDFHRESSKVIIALDEMHYDVYD
KNCEKGR
>ggPNR
MGSAQSRSVEKGMLIASCYEVNGSCHRTLHPFGVHLAIYLACALGTLITVLGNLLVVIVVSHFQALHTPTNFLLLSLALAD
MLLGLTVLPFSTIRSVESCWYFGDDFCKLHTFLDTLFCLTSIFHLCFISIDRHCAICDPLLYPTKFTLRVACMCIGVGWAV
PMVYTSVFLYTKEIAEGLGHFLQDVPCVGSCQLLFNKLWGWLNFPVFFFPCLITIALYVKIFTVANKQARLINNMNKSFGA
HLHRGASRSERRAAKTLGVAVGVYLLCWLPFTIDTMIDSLLNFITPPVLFDTLIWFAYFNSACNPLIYVFSYHWFRKAVKL
VLTQGIFCSRTSRVDLYQ
>ggPTGER2
LTALGRGGHICLYFGFAMSFFGLATMLVLFAMALERCLALGRPYFYERFLSPRAGLVALPAIYTFSAAFCSLPLLGFGRYV
QYCPGTWCFIQMRVDDGRHKAAGLHVTFSLLYATLLLFLILSVLLCNLSVIANLARMRRRGQRTRRLATLEQPRGVGGSGR
RIFSMAEEIDHLLLLAIMTITFVICSLPFTEVYWPPKRVWADVSMGCDCSSPADGFLAVLQPGDGCGAGLCQCLTTLLEKK
FWPSPNTTGGHRSHPIAVTCEQRPAASKHKGTYTGRDGTSCVPGQHPGDTPWWESSGCPKSMKQLEGVGDS
>ggPTGER3
MKETRGYGGDAPFCTRLNHSYTGMWAPERSAEARGNLTRPPGSGEDCGSVSVAFPITMLLTGFVGNALAMLLVSRSYRRRE
SKRKKSFLLCIGWLALTDLVGQLLTTPVVIVVYLSKQRWEHIDPSGRLCTFFGLTMTVFGLSSLFIASAMAVERALAIRAP

HWYASHMKTRATRAVLLGVWLAVLAFALLPVLGVGQYTVQWPGTWCFISTGRGGNGTSSSHNWGNLFFASAFAFLGLLALT
VTFSCNLATIKALVSRCRAKATASQSSAQWGRITTETAIQLMGIMCVLSVCWSPLLVGSSRVGGSQDACASKREPKTLLSG
STRRGVFATAER
>ggPTGER4
MSFNSTTILQPANGSANGTASAAEGGKPATIPTVMFIFGVVGNLIAIVVLCKSRKEQKETTFYTLVCGLAVTDLLGTCLVS
PVTIATYLQNRWPGGPALCEYSSFILLFFGLSGLSIICAMSIERYLAINHAYFYNHYVDKKLAGLTLFAIYASNVLFCALP
SMGLSKSTLQYPDTWCFIDWRAEKATHAAYSYMYAGFSSFLIMVTVICNILVCVALIRMHRQFMRRTSLGTDTTSSRLSDF
RRRRSFRRMAGAEIQMVILLIATSLVVLICSIPLVVRVFVNQLYRPPFVKDLRQNPDLQAIRIASVNPILDPWVYILLRKT
VLRKAIEKIKCLFCQIGGARRQHSGSNFNCVDGRRTSSAMSSQSPSFISRELREISSTSQTLLYPPELSESSVGGGVLLPG
PSASLAQSDTTSVRTLRSSETSDSSQGQDSESVLLVNDIRSGGGATSMSKGSPLQVTFPTETLNLSEKCI
>ggPTGFR
MNSSKSPGLAGFGILKNTTCHTEKKISVFFSIIFMTVGILSNSLAIAILMKAYQRFRQKSKASFLLLASGLVITDLFGHLI
NGAIAVFVYASDKDWIRFNQSNILCSVFGICMVFFGLCPLFLGSVMAVERCIGVTKPIFHSTKMTSKHVKMMLTMVCLFAV
LIALLPILRFRAYQIQASRTWCFYKTEHVEDWEDRFYLLLFSSLGLLALAISFLCNAVTGITLLRVKFKSQQRQGRSHHFE
MIIQLLAIMCVSCICWSPFLVSMARIGINASHSKENCETILFALRMATWNQILDPWVYILLRKAVLKNLYKITRGCCGMHI
INLHMWELSSIKNSLKVAAISESPVSSKQINLQPLSSTGQ
>ggRHO
MNGTEGQDFYVPMSNKTGVVRSPFEYPQYYLAEPWKFSALAAYMFMLILLGFPVNFLTLYVTIQHKKLRTPLNYILLNLVV
ADLFMVFGGFTTTMYTSMNGYFVFGVTGCYIEGFFATLGGEIALWSLVVLAVERYVVVCKPMSNFRFGENHAIMGVAFSWI
MAMACAAPPLFGWSRYIPEGMQCSCGIDYYTLKPEINNESFVIYMFVVHFMIPLAVIFFCYGNLVCTVKEAAAQQQESATT
QKAEKEVTRMVIIMVIAFLICWVPYASVAFYIFTNQGSDFGPIFMTIPAFFAKSSAIYNPVIYIVMNKQFRNCMITTLCCG
KNPLGDEDTSAGKTETSSVSTSQVSPA
>ggGREENOPSIN
MNGTEGINFYVPMSNKTGVVRSPFEYPQYYLAEPWKYRLVCCYIFFLISTGLPINLLTLLVTFKHKKLRQPLNYILVNLAV
ADLFMACFGFTVTFYTAWNGYFVFGPVGCAVEGFFATLGGQVALWSLVVLAIERYIVVCKPMGNFRFSATHAMMGIAFTWV
MAFSCAAPPLFGWSRYMPEGMQCSCGPDYYTHNPDYHNESYVLYMFVIHFIIPVVVIFFSYGRLICKVREAAAQQQESATT
QKAEKEVTRMVILMVLGFMLAWTPYAVVAFWIFTNKGADFTATLMAVPAFFSKSSSLYNPIIYVLMNKQFRNCMITTICCG
KNPFGDEDVSSTVSQSKTEVSSVSSSQVSPA
>ggRRH
MHWNDSANSSESDAEAHSVFTQTEHNIVAAYLITAGVISIFSNIVVLGIFVKYKELRTATNAIIINLAFTDIGVSGIGYPM
SAASDLHGSWKFGYTGCQIYAALNIFFGMASIGLLTVVAVDRYLTICRPDIGRRMTTRNYAALILAAWINAVFWASMPTVG
WAGYAPDPTGATCTVNWRKNDVSFVSYTMSVIAVNFVVPLTVMFYCYYNVSRTMKQYTSSNCLESINMDWSDQVDVTKMSV
VMIVMFLVAWSPYSIVCLWSSFGDPKKISPAMAIIAPLFAKSSTFYNPCIYVIANKKFRRAILAMVRCQTRQEITISNALP
MTVSLSALTS
>ggSREB2
MANYSHAADNILQNLSPLTAFLKLTSLGFIIGVSVVGNLLISILLVKDKTLHRAPYYFLLDLCCSDILRSAICFPFVFTSV
KNGSTWTYGTLTCKVIAFLGVLSCFHTAFMLFCISVTRYLAIAHHRFYTKRLTFWTCLAVICMVWTLSVAMAFPPVLDVGT
YSFIREEDQCTFQHRSFRANDSLGFMLLLALILLATQLVYLKLIFFVHDRRKMKPVQFVAAVSQNWTFHGPGASGQAAANW
LAGFGRGPTPPTLLGIRQNANTTGRRRLLVLDEFKMEKRISRMFYIMTFLFLTLWGPYLVACYWRVFARGPVVPGGFLTAA
VWMSFAQAGINPFVCIFSNRELRRCFSTTLLYCRKSRLPREPYCVI
>ggSREB3
CTVPHTTCCRQVLGRWAPLAGLPSKVLLSVRSGAAWPHGLLSCKVLAFLAVLFCFHAAFLLFCVGVTRYMAIAHHRFYAKR
MTGWTCLAVVCMVWTLSMAMAFPPVFDVGTYKFIREEEQCIF
>ggHTR1E
MNFTNCTTEASAAAKPKTVTEKMLVTLTLATITTLTMLLNSAVIAAISTTKKLHQPANYLICSLAVTDLLVAVLVMPLSIT
YIMIDKWTLGYFICEIWLSVDMTCCTCSILHLCVIALDRYWAITDAIEYARKRTAKRAGLMIVTVWTISIFISMPPLFWRN
HHSVSIPSECRIQHDHVIYTIYSTFGAFYIPLTLILILYYRIYHAAKSLYQKRGSSRHLSNRSTDSQNSFASCKLTQTFCV
SDFSTSDPTTEFDKINASVRIPPFENDLDLAGDRQQISTTRERKAARILGLILGAFILSWLPFFIKELLVGLHLCIVSPEV
ADFLTWLGYVNSLINPLLYTSFNEDFKLAFRKLIRCREHT
>ggGPR119
MASLAVGIILAVLAPLIITANVLVAIALLRLIWKTGYKGHYFVLNLAAADALVGLSVAGLIRDELFERSHPPHMFCVLRMA
FVTTSSAASILSLTLVACYRHLAIRKPFHYHQLVTGMRVGLHLVGLWLLAGIVGFLPLLIPGFQKIPLEEKCSFFGVFHLR
YMLIVSCMGFFPALFLFIYLYCDMLKIASVHVQQIREVERAGLARGTTSDMKAMRTVAMLIGCFVLSWLPFFIAGIVQTVC
TECFPYRVLESYLWLLGLCNSLLNPLLYSYWQKDVRRQLSQLAAGVKRKALRVGKGCCFPSRGTKSIPTVSCLQLQD
>ggGPR119n1
MVSVLCAVLHALLSCLIPPANLLVIVAVCQLLRKHPGSGYIYVLNLATADLLVGVMCITEALDDVLDGDFDRSKSFCLLRI
SLSMTPCIGSILTLLLISLDRYLAVRLPLSYRTLLKKAPVALSLLFLWMLAFLLGHLPLIFPSLQQSNYTGYCGLLSVARS
EYLYTLCFGIFAPSLLVLLCLHVSVCSIAYAQHTRLPHSCTQPGPLRARLRHFKALRTVLFVLIGFGLSWGPYLVGGTVQA
ACRSCDLSGALKDALFILGETNSLLNPLIYALHSRDIRSHLAKLLGCRQRGRVQPVASSIRTVGGGSWPRTSGL
>ggTAR1
MQLCCESVNGSCVKSNWSNSIRISMYIFMVCVILTTVIGNLMVIISISHFKQLHTPTNFLIISMATADFLLGFLIMPCSMV
RSVEHCWYFGDFFCKIHTSTDIMLSTTSIFHLSFISIDRYYAVCDPLRYKSKINAFVILVMILVSWTVPAAFAFGMIFLEL
NLLGAKEIYNHTHCAGGCLVFFSETSGVVTSVVSFYIPGLVMLFIYRKIYSIAKRQARSIDAVSKKKKRYETKHISFCRER
KAAKTLCIVMGVFLICWSPFFFFTATDPFMNYAAPPVLTDTLVWFGYLNSAFNPIVYAFFYMWFRRALKTILFGNIFQQDS
SRTQLFLE
>ggGPR153
MHDEQRLPGNAVAWLACAGISLLANAWGILSISAKQKKWKPLEFLLCSLAGTHILNTAIPITTYAVVQLRRWHSGYEWNEG
LCKVFVSTFYTLTLVTCFSVTSLSYHRMWMVRWPVNYRLSNTRKQAVHTVMGIWMVSFILSTLPAVGWHDTTERFYARDCR

```
FVVTEIGLGFGVCFLLLIGGSMAVGAVCIGIALFHTVLGRRGPDASNSRFHVPTIVVEDAQGKRRSSIDGSESVKTSLQIT
YLITVIVLVYDILMGFPVLVVSAASLRSDTSSEWMVLCLLWCSLAQSLLLPLFLWACDRYRANPRAVCEKCRTVLANDDE
>ggHRH1
MSKNTTENSNYTHSALLGFLLASISLITVVMNILVLCAVKTEKKLQTVGNLYIVSLSIADLIVGAAVMPLNIVYLLNRVWT
LGLTACLFWLSMDYVASTASIFSLFILCLDRYRSVQQPLKYLQYRTKMRASLVILGAWLLSSLWVIPILWWHDSANDEKRK
GKANCDTQFSHVTWFKVLTAIVNFYLPSIIMLWLYCKIFRAVRKHCQQREVVTGSYCSFSENKNVIHGKTKDKQNICLQKQ
ILHENTSHKDKQMSSEHKSTAAKYHFSNSDKSSKAFVSSSNSKAFQCSCLPLTTAQSEPDLEKAGKKCVCVKKDNKNEEDP
HSQDSDLSATSDSDTFTENVPSGEQSNPNPDRACSPQENKENRDFRGLSYLRKTWQTLHTHSKSRLRRQHVSREWKAAKQL
GVIMAAFMLCWIPYFVLYMVTVFQHREQFYTLHMFTIWLGYVNSTLNPFLYPLCNENFKKTFKKILHIQ
>ggHTR2A
MDTLCDEESSVNPTANPLIQINHERRLYRNVYGPGEINVSHFCNLTADPENLTNLSCESPPCYSSLFQLSEKNWPALLTVI
VIVLTIAGNILVIMAVSLEKKLQNATNYFLMSLAIADMLLGFLVMPVSMLTILYGYKWPLPRKFCAIWIYLDVLFSTASIM
HLCAISLDRYIAIRNPIYHSRFNSRTKAFAKIIAVWTISVGISMPIPVFGLQNDCKVFKKGHCLLADENFVLIGSFAAFFI
PLTIMVVTYFLTIRSLQKEAMLCVNDIGPKTKFASFSFLPQSSLSSEKLFQRSLNRDMGTSGRRTMQSISNEQKASKVLGI
VFFLFVVMWCPFFITNVMAVICKESCNEEVIGGLLNIFVWIGYLSSAVNPLVYTLFNKTYRSAFSRYIQCRYKEEKKPFQL
ILVNTIPALAYNSSQLHLAQMKSLKKEAKLMSKDYSMVTIGIRHMDGTSKGSISPGNEKVSGV
>ggDRD2
MDPLNLSWYNSGDRNWSKPLNESSADQKPQYNYYAVLLTLLIFVIVFGNVLVCMAVSREKALQTTTNYLIVSLAVADLLVA
TLVMPWVVYLEVVGEWRFSRIHCDIFVTLDVMMCTASILNLCAISIDRYTAVAMPMLYNTRYSSKRRVTVMIAVVWVLSFA
ISSPILFGLNKADEKECIIANPAFVVYSSVVSFYVPFIVTLLVYVQIYIVLRKRRKRVTTKRSSHVLDSDTQAPLKEAESH
IEMEMEMVSSTSPPERTLAKPAAPSNHQLVVPIASRRCTLDSPGKVEKNGHAKENLHTAKVFEIQSMPNGKTRSTLLKAMN
RRKLSQQKEKKATQMLAIVLGVFIICWLPFFITHILNMHCDCNIPPAMYSAFTWLGYVNSAVNPIIYTTFNIEFRKAFMKI
LHC
>ggADRA1B
MSTYLAEKSNSSILGYLKVSALNSGNLSANSSNETTSNLDSPTLDISRAITVGLILGAFILFAIIGNILVILSVACNRHLR
IPTNYFIINLAIADLLLSFTVLPFSATLEILGYWVLGRIFCDIWAAVDVLCCTASILSLCAISIDRYIGVRYSLQYPTLVT
RRRAILALLGVWVLSMVISIGPLLGWKEPAPKDDKECRITEEPFYALFSSLGSFYIPLIVILVMYCRVYIVAKRTTKNLEA
GVMKEMSNSKELTLRIHYRNIHEDTLNSNKSKGHNSRNSLALKLLKFSREKKAAKTLGIVVGMFILCWLPFFIVLPLGSLF
SALKPPEIVFKVIFWLGYFNSCLNPIIYPCSSKEFKRAFIQILKCQCHRRKQSGWWAYSYRNWNRCSFEHPRKDSLEDSGS
FLSGSQRTLSSASPSPGYLSKITHPHMEMCTFQEWKNPSSFLSPLQEGSRQKDPCQFFTFNLLTERNGHIAASSQASSNGD
HEDIFDTVNGKTDCRTNIMLE
>ggADRB2
MGVAGTELPGGNVSANQSAADPTASRDVWVVGMGILMSLIVLVTVFGNVLVITAIARFQRLQTVTNYFITSLACADLVMGL
GVVPFGACHIIMEMWKFGNFWCEFWTSLDVLCVTASIETLCVIAVDRYFAITSPFKYQSLLTKSKARVVILVVWAISALTS
FLPIQMHWYRADRDEAILCYEKDTCCDFFTNQAYAIASSIISFYLPLVVMVFVYARVFQVAKKQLQKIDQSEGRFHIQNKE
QDQNGKAGHRRSSKFFLKEHKALKTLGIIMGTFTLCWLPFFIVNIVHVIQDDIIPKYVYILLNWLGYVNSAFNPLIYCRSP
DFRYAFQELLCLRRSALKMYANGYSNSNGRSDYNEEDNGYPLAPDKACELLCEEGSFPHPEDFLHCKGTVPSG
>ggHTR1F
MDLINSTEQNSTSEELFKWVTSKILISITLSVLALMTTAINSLVMTAIIVTRKLHHPANYLICSLAVTDFLVAVLVMPFSI
VYIVKETWIMGQVVCDIWLSVDITCCTCSILHLSAIALDRYRAITDAVEYARKRTPKHAGIMIAVVWIISIFISMPPLFWR
HQTTSRDDECIIKHDHIVSTIYSTFGAFYIPLALILILYYKIYKAAKTFHRRSVSRIVREEVNGQVLLDAGERTTKLTSMP
STIEKTSDPLVDCDKINISIRSPRSESKHEKSWKKQRISSTRERKAATTLGLILGAFVICWLPFFVKEVVVNTCERCHISE
DMSNFLAWLGYINSLINPLIYTIFNEDFKKAFQKLVRCRQYL
>ggTAR2
MFSMNISRDFTDCSEFGNRSCSDSSRSPGVRGVLYFSIAAAFLVTVSGNMAIIFSILYFKQLHSPTNFLILSMAVTDFLLG
FAIMPYSMVRSVENCWYFGMMFCKIHYSFDLMLCLVSIFHLCSIAVDRFYAICHPLHYASTMTMAAIKQITAVCWSVPAVF
AFGVVFSEAHASGIEGYESLVKCSSLCPIMFNKAWGIVLFTIGLFAPACIMLGIYIKIFVVSQKHIRVLSQTHKHRKSDMK
NELSKSKDKKAAKTLSVVMLAFLVCWFPCFFAILLDPFLNFSIPSPLFDALNWFGYLNSTCNPLIYGFFYPWFRKAFKYIV
TGKIFNPQLRTTKLSSED
>ggADRA2B
MEAAVTLPNASNNGSQAGSPPHSPAATGLILLAVLAVLLATLVGNALVVAAVSTSRALRAPQNLFLVSLASADILVAVLIL
PFSLAKELMGYWYFGSLWCSLYLALDVLLCTASIGHLCAISLDRYWAVTRAARLNLRRSPGRVKGMIGAVWAAAALVALPP
LFKARPRARQCELIDETWYVLASCVASFFAPCLVMVTVYCRIYRLTTRRSAAVLAARRGSAPCPGDANRRASWAAAPSRRH
RSQHQSVSLCRRRLLRVRERRFTIVLAVVMGAFVLCWFPFFFTYSLEAVCGEGCRVSKPLFSFFFWIGYCNSSLNPIIYTV
FNRDFRAAFRRLLAALVRHGS
>ggHTR1D
MTQYNLSAELSFQSSANKSLNFTEPPLAVDERTLLGLKISLSVLLSVITLATILANVFVVITIFLTRKLHTPANYLIGSLA
VTDLLVSVLVMPISIAYTVTHTWAFGQVLCDIWLSSDITCCTASILHLCVIALDRYWAITDALEYAKRRTAGRAMLMIAVV
WMISISISVPPFFWRQVKAHEEIAKCNVNTDQISYTIYSTCGAFYIPTVLLLILYGRIYVAARSRILKPPSLYGKRFTTAH
LITGSAGSSLCSINASLHEGHSHSGGSPIFINHVQIKLADSILERKRISAARERKATKTLGIILGAFIFCWLPFFVMSLVL
PICQDACWFHPILLDFFTWLGYLNSLINPVIYTAFNEEFKQAFQKLIHFKKCSS
>ggGPR136
GNGYVIFMSSKRKKKLRPAEIMTVNLAVCDLGISKPFSIISFFSHRWIFGWMGCRWYGWAGFFFGCGSLITMTAVSLDRXX
XXXXXXXXXWLKRHHAFICLALIWAYATFWATVPFAGVGSYAPEPFGTSCTLDWWLAQASVAGQAFVLSILFFCLLFPTAV
IVFSYVKIILKVTKEVAHYDTRIQNSHILEMKLTKVAMLICAGFLIAWIPYAVVSVWSAFGQPDSVPIQFSVVPTLLAKSA
AMYNPIIY
>ggGNRHRII
MARLGGGTGQDAAAAGGGWLDPGPTVGNVSTEPSSSTSHPKRGCAWSPRLESAEEPLLLPTFSPAAQARVAATFVLFVLSA
```

GCNVAVLRAAGGRRGGGRSHIRVLLRHLAAADLLVTVVVMPLDAIWNITLQWRAGDLACRLLMYLRLLAMYASAFVTVVIS
LDRQAAILRPLAIARARCRNRAMLRAAWMLSAALAVPQLFLFHTVTLHAPHNFTQCTTHGSFPQPWHETLYNMLSFSCLFL
LPLLIMVCCYTRILLEISRRMGSSLFSSRDVPLRCSGSNIPRARLRTLKMSLVIVSSFILCWTPYYLLGLWYWFCPRAMQQ
KVPPSLSHILFIFGLFNACLDPITYGLFTIPFRRRCGCPCGHSSEPEPPSPATGSFHCSASSLRGRQGMGGTEGPHPPIEL
GLPTGAGSCQSSAL
>ggGPR103
MRSLNITPEQFAQLLRDNNVTREQFIALYGLQPLVYVPELPGRTKVAFVLICVLIFALTLFGNCLVLYVVTRSRAMRTVTN
IFICSLALSDLLIAFFCVPFTMLQNISSEWLGGAFACKMVPFVQSTAIVTEILTMTCIAVERHQGIVHPLKMKWQYTNKRA
FTMLGIVWLLAIIVGSPMWHVQRLEVKYDFLYEKVHICCLEEWASPTYQKIYTTFKKRAIVMMVTVVFLFAVCWAPFHIIH
MMMEYSNFEKQYDDVTIKMIFAIVQIIGFFNSICNPIVYAFMNENFKKNFLSAICFCVVKENASPTRQLGNSGITMRRQKA
GDSQRAPTDSDEARREAFSDGNIEVKFCDQPSSKRNLKRHLTLFSSELPAHSASAQ
>ggAVPR1B
MEPGWSWNSTHSSQTTHSTELQSSQPPAGTPNRTLLQGRDEQLARAEVGVLAAILLVATTGNLAVLLAVCRRGRKLSRMHL
FVLHLALSDLGVALFQVLPQMLWEVTYRFAGPDLLCRAVKYLQVLSMFASTYMLMAMTLDRYVAVCHPLRTLRRPGRQPCA
MVGAAWLLSCLLSTPQIFIFSLREVQPGSGVLDCWADFGYPWGARAYITWTTLCIFLLPVGILTACYSLICREICKNLKGK
TQSCTPVAGGLLAASPSAPCCSQKGSQCPSGQPSRVSSVRTISRAKIRTVKMTFVIVVAYVACWAPFFSMQMWSVWDEDAP
DDESADAAFTITMLLASLSSCCNPCIYMFFSGRPLQDTARCLALWGCPPSAPRRQGSAGSLCSRRTTILSHGLSAGIPLHR
GSGTDLSPPEEEAVTESGTL
>ggVT1
MKNFSFPMQDSTHQTESPPHRLLSLTNKSDPVGRPERDEQLAQVEIAVLGVIFLTASVGNFILILVLWRRRKKLSRMYVFM
LHLSIADLVVAFFQVLPQLIWDITDVFIGPDFLCRIIKYLQLLGMFASTYMIVVMTVDRYQAVCYPMVTFQKKRALWNIPI
CTSWSISLILSLPQVFIFSKIEISPGIFECWAEFIQPWGPRAYVTWILVVIFFIPSTILITCQVKICKIIKRNIYVKKQNE
YQVTNQKQVLPSRASSVNCISKAMIKTVKMTIVTVVAYVLCWSPFFIAQLWSVWFPSGITEGSAFTIIMLLGNLNSCTNPW
IYMYFCGHIPYCTNKQLENTSAQEDSVVTGSIHLVDRDPEENSTCA
>ggBRS3
MGKEMSQVYLHPSNQTLCAATNGTELKSILDNETTNEKWTEDSFPGLEILCTIYVTYAVIISVGLLGNAILIKVFFKIKSM
QTVPNIFITSLAFGDLLLLLTCVPVDATRYIVDTWIFGRIGCKLLSFIQLTSVGVSVFTLTVLSADRYRAIVKPLELQTSD
ALLKTCCKAGCVWIVSMVFAIPEAVFSDLYSFSNPEKNVTFEACAPYPVSEKILQEVHSLVCFLVFYIVPLAVISVYYFLI
ARTLYKSTFNMPAEEHGHARKQIESRKRVAKTVLVLVALFAFCWLPNHILYLYRSFTYHTSVDASTFHLIVTIFSRALAFS
NSCVNPFALYWLSKSFRQHFKKQVSCCKAKLCTKPPSAPHSNSPPRALSVTGSTHGSEISVTLLTDYSITKEEESV
>ggEDNRA
MEALYLRVSSLLLLLGFVLCDSSDKYATNWSDVGIYLPTSSGIESSLLPTTRRPPVSNQTVKCSQQTKIAETFKYINTVVS
CAIFIVGMVGNATLLRIIYQNKCMRNGPNALIASLALGDLIYIVIDIPIIVYKLLAQKWPFGDSEFGQFLCKFLPFIQKAS
VGITVLNLCALSVDRYRAVASWSRVQGIGIPMITAIEIFSIWLLSFILAIPEAIGFAVVPFRYKDESYVTCMLNPTNKFML
FYKDAKDWWLFGFYFCMPLACTAIFYTLMTCEMLNRRNSNLRIALSEHLKQRREVAKTVFCLVVIFALCWFPLHLSRILKK
MVYNERDPGRCELLSFLLPLDYISINLATMNSCINPIALYFVSKKFKNCFQSCLCCCCSQSKSLATSVPMNGTSIQWKNQE
LNNHNTDRSSHKDSIN
>ggEDNRB
MARIPAPTTLAILLTCLFSGACSQTPEAFQASSIPFEALSQEQAYSLVQPSLFQDAKAPNYSESLPKSSGSEPPLLPVCVK
PADIRHIFKYINTIVSCTIFILgiiGNsTLLRIIYkNKCMRNGPNiLIASLALGDLlhIVIDIPInVYKlLAKDWPFGVQV
CKLVPFIQKASVGITVLSLCALSIDRYRAVASWSRIQGIGIPMWKAVEVTVIWAVAIVLAVPEAIAFDMVEINYWDQDLWV
CMLASEQKSSFMMFYRDVKDWWLFGFYFCLPLVCTGIFYTLMSCEMLSKRNGMRIALNDHMKRRREVAKTVFCLVVIFALC
WLPLHLSRILKKTIYDQTDPNRCELLSFLLVMDYFGINMASLNSCINPVALYFVSRKFKNCFQVGMVLPLP
>ggEDNRBn1
MPAARLWLLPALLLLLLGRRPAAAEGDGALRPPAPGRLSPAADDRTPPTAAPPAPRGAEPNASWAAALPQGGGEAGRGRPR
SGSLPMCTGQTEIKETFKYINTGVSCLVFVLGIIGNSTLLRIIYKNKCMRNGPNILIASLALGDLLHIIIDIPISVYKLLA
EDWPFGVEMCKLVPFIQKASVGITVLSLCALSIDRYRAVASWSRIKGIGVPRWTAVEIVLIWVISVVLAVPEAIAFDMITM
EYRGKDLRICLLHPTQKTSFMMFYKKAKDWWLFSFYFCLPLAITALFYTLMTCEMLRKKSGMQIALNDHLKQRREVAKTVF
CLVLVFALCWLPLHLSRILKLTIYDQKDPNRCELLSFFLVMDYIGINMASLNSCINPIALYLVSKRFQNCFK
>ggETBRLP1
LIPAAGSALPAPPQRSPLAPTPLAPPDRRAAAAAPGRGAEAEHGRRRARSSESGAGAEEGSGPRWLPLNGSARGESAAGGR
GNGTGRRARLRNPFYPLTEESYGAYAVMCLSVVIFGIGIMGNMAVMCIVCHNYYMRSISNSLLANLAFWDFLIVFFCLPLV
IFQELTKKWLLEDFSCKIVPYIEVASLGVTTFTLCALCIDRFRAATNVQMYYEMIENCTSTTAKLAVIWVGALLLALPEVV
LRQLTKEDPEYSGSPPGERCVVKISTTLPDTIYVLALTYDGARLWWYFGCYFCLPTLFTITCSLVTARKIRRAEKACTRGN
KRQIQLESQMNCTVVALTILYGFCIIPENICNIVTAYMATGVSRQTMDLLHLISQFLLFFKSCVTPVLLFCLCKPFSRAFM
ECCCCCCDECIQKSSTVTSDDNDNEYTTELELSPFSTIRREMSTFASVGTHC
>ggETBRLP2
MPGAAVQHAEGGVHPNRGAGGGGMLPVAAQDNVELKARPRRGTVEDSKSVQQYVPGVRVEFPRPINAASLHPTRAMVPSST
DPSEQQRGVPTDGGAEPATNLTTVSDKRLQIQNPLYPVTENSYSAYAVMFLSLIVFVVGIIGNLSVMCIVWHNYYMKSAWN
SILASLAFWDFLILFFCLPVVIFNEITKKRLLGDISCRIVPFMEVSSLGVTTFSLCALGIDRFHAATSPQASARPIEQCQS
IIAKLAVIWVGSMTLSVPEVLLWQLAQDTSPVSGVVTEYCTMKPSSNLPESVYSLVLTYQNARMWWYFGCYFCLPILFTVS
CQLVTRRIRGTEKKAECRGTKHSQCESHLNCTIIGLTIIYGLCTTPENVCNIVVAYMSPDMSKQTLDLLNLINQFFLFFKC
SVTPVLLLCLCRPLGQAFMDCCCCCCEGCGPDAASSEGSADSKLKTEMSSSIFFDKPRESPPPLLALGTPC
>ggGNRHR
MCVPAALIEAEPPHHPTTEGDTNTSATHCLEHWVEPRFTKAAKVRVAITAVFFLLAACSNTAVLGSLLRKRRKCHVRPLIL
SLALADLLVTVAVMPLDAAWNVTVQWYGGDLSCKLLNFLKLFAMYAAALVLVVISLDRHAAVLQPFARARRRNGLLLRAAW
LGSVLLASPQLFLFHVHTVPGGNFTQCVTHGSFRAHWEETVYNMFTFTTLYITPLSIMIVCYVRIIWEISKQLKINKSLVR
SQNDHISKARMKTLKMTIVIVASFIICWTPYYLLGLWYWFHPAMIQRMPEYINHSFFLFGLLHTCTDPIIYGLYTPSFRED

VQLCLRGIEAAISQHVRHKPISVSEKTTKDGDVNGQVTSGGSNGTTVNTVC
>ggGPR72
MSRRMWFPLQYISKPFWRAENHTSRSFLSTRYSFPNQSFFHSDLDLEDLGDFDSGTKYEGESQSRTVQALLIIAYSVIICI
SLFGNTLVCHVVIKNKRMHSATSLFIVNLAVADVMITVLNTPFTLVRFVSSTWVFGKLMCHISRFVQYCSVHVSVLTLAAI
ALDRHQVIMHPLKPRMSMVKGGICIIIIWVMASCFSLPHAIYQTLTRFYIGNRTIRTVCLPSFPPPADLFWKYLDLTTFVL
LYVLPLLVISITYTMVAKKLWLRNAIGDITMEQYYAHQRKKKMTLKMLMVVVIVFAVCWFPLNCYVVLISCRAIHSSNALY
FAFHWFAMSSTCYNPFIYCWLNENFRSELKSLLCVCRRRSAAQSHALQSISPPFRPAWLESCRHKRGSTCQKTAPSQRNST
KTDISSVQPIVAEH
>ggGPR72n1
MFSCFVWLSLPCFVNTFPTSGKLPLNRSLEEVLEIPTISGFFPWDNDTLADWQSFVGRSRHGADSQHVAAKALLIAAYSII
IVFSLFGNVLVCHVIIKSKRLRSATSLFIVNLAVADIMITLLNTPFTLVARFVNSTWIFGKGMCHVSRFVQYCSLHVSALT
LTAIAVDRHQVIMHPLKPRLPMAKGVIYISVIWVMAACFSLPHAIYQKLFTFEYXXEEVTRCLCIPDFPEPADLFWKYLDL
TTFVLLYVLPLLIISAAYMTVAKKLWLRNVIGDVTTEQYFALRKKNKKTIKMLMLVVILFAICWFPLNCYVVLLSSQTIRS
NNALYFAFHWLAMSSTCYNPFIYCWLNDSFRAELKALLNICRKPPGPVEQRLPSTVPSYRLAWPENSHFKRLQVSHVPVAA
SNSHLGKTDISAVEPIVAVS
>ggGRPR
MASGECLLLDLETDNFILYNISANQSANLSVLSDEWFYPAFLYAIPTIYGIIILIGLIGNITLIKIFCTVKSMRNVPNLFI
SSLALGDLLLLVTCVPVDASRYLADEWLFGRIGCKLIPFIQLTSVGVSVFTLTALSADRYKAIVRPMEIQASHALMKICVR
AAIIWIASMLLAIPEAVFSDLHPFHDKGTNKTFISCAPYPHSDGLHPKIHSMASFLIFYIIPLSVISVYYYFIAKNLIRSA
YNIPVEGNVHVRKQIESRKRLARTVLVFVCLFAFCWLPTHIIYLYRSYHYSEVDTSVLHFIASICARILAFTNSCVNPFAL
YLLSKSFRKQFNNQLFCCRARLLIRSQSMARSTTRMTSLKSTNHSLATFSLINGNHVCHEGYV
>ggHCRTR2
MSGTQPEDVSPPCRNWTSSPELNETREPFLNPSADYDDEEFLRYLWKEYLHPKEYEWALIAGYIVVFIVALVGNVLVCIAV
WKNHHMRTVTNYFIVNLSLADILVTITCLPATLVVDITETWFFGHHLCKAIPYLQTVSVSVSVLTLSCIALDRWYAICHPL
MFKSTAKRARNSIIIIWIVSCIIMIPQAIVMECSSVFPGLANKTTLFTVCDEHWGGECVYPKMYHTCFFLVTYMAPLCLMV
LAYLQIFRKLWCRQIPGTSSVVQKKWKSLQSSAQQRGLGQSTKSKISAVAAEIKQIRARRKTARMLMVVLLVFALCYLPIS
ILNILKRVFGMFNHADDRETVYAWFTFSHWLVYANSAANPIIYNFLSGKFREEFKAAFSCCIFGIHSHHDERLTRGRASTE
SRKSLTTQISNFDNVSKQSEHVVLTNINTLTANGNALITH
>ggNMBR
MEAELLSELTALSFGGNGSVQLVPETTLLPPAWGMAMFTVRCVIPSLYLLIITVGLLGNVTLMKIFISNSAMRSVPNIFIS
SLAAGDLLLLVTCVPVDASRYFSEEWLFGEVGCKLIPAIQLTSVGVSVFTLTALSADRYKAIVKPMDIQTSSAVLRTCAKA
IAIWIISILLSVPEAVFSELAHINDTDNATFTACIPYPMTDDMHPKIHSVLIFLVYFLIPLAIISIYYYHIAKSLIRSAHN
IPGENSEHSKRQMETRKRLAKIVLVFVGFFAICWFPNHVLYMYRSFNYNEIDSSIGHMVVTLVARVLSFCNSCVNPFALYL
LSESFRRHFNNQLRCRKKPQQERSASYLRNSSAIQTTSLKSNTRNTVTSVTQLNGHNLKQEMSS
>ggNMU1R
MEPPSPDLCNRTHPDALFDPKDANLTEEQLRHKYLGPRRSSFFIPVCVIYLLIFAVGAVGNTLTCIVILRHRFMRTPTNYY
LFSLAVSDLLVLLLGMPLELYDMWSNYPFLLGAGGCYFKTLLFEAVCFASILNVTALSVERYIAVVHPLKAKYVVTRNHAK
RVIITIWVVSVICSIPNTSIHGLQALYVPGRGRVPDSEICTLVKPRLTYNLIIQITTIVFFFLPMGTISILYLLIGLQLKK
EKMLEALGAKSSGDSDYHRVRGQKKVKRRQVTKMLFVLVVVFGICWAPFHTDRLVWSFISTWTGPMLHMFQYVHIISGVFF
YLSSAANPILYNLMSTRFREMFKEVMCHPGQRPPGSCKHSPSVTHATTRSTECEPIPSANGLPLSDAEEYEMEEMEEEMEM
EGGRAVTHATSLC
>ggNMU2R
MAWIGNSSWFNHLALQEHFRRYLNSTEDYLAFLCGPKRSHLFLPMALVYSVIFVVGVIGNFLVCLVILKHQNMKTPTNYYL
FSLAVSDLLVLLFGMPLEVYEMWSNYPFLFGPIGCYFKTALFETVCFASILSVTTVSIERYFAILHPFRAKLESTRKRALR
TIVVLWVLSVLFALPNTGTHGIMLQYFPNGTLVPGSATCTVVAPMWIYNWIVQITSFLFYVLPMGVISVLYYLMGLRLKGD
RSLEAEEMSVNVQRPSRKSVTKMLFVLVMVFAVCWAPFHIDRLFFSFVVEWTEPLANIFNLIHVVSGVFFYLSSAVNPIIY
NLLSQRFRMAFLSVISPGCKHWAPKHTTSKTPPQQSIFMVEDHNLVDSAEDTSVPGTNRTSVCSSQLSTGL
>ggNPFF1
MQALQHPEPSGDPSNGSWANVSESHLLKENYTFLAYYQHSSPVAVMFILAYTFIFLMCMIGNILVCFIVVKNRQMRTVTNM
FILNLAISDLLVGIFCMPTTLVDNLITGWPFDNAMCKMSGLVQGMSVSASVFTLVAIAVERFRCIVHPFRQKLTLRKALVT
IAIIWVLALLIMCPSAITLTVTREEHHFMVDTYNNSYPLYSCWEAWPETEMRRIYTTVLFSHIYVAPLALIVVMYARIAFK
LFKSAAPARGTRPEEPEGRKVSRRKAKVINMLIIMALFFTLSWLPLWTLMLLTDYRHLSEHQLHLVTGYVFPFAHWLAFFN
SSANPIIYGYFNENFRRGFQEAFRAPFCLPPRRQHRGPYGARNRVFTQPQASDSPPHSESRPLASRRAGVPAWNG
>ggNPFF2
AHHNMIKETYSNSSFNWPHLLSYNGTYKYLYLEGNVSYVDFYHHQPSVAAVFIASYLLIFLLCMVGNGVVCYTVLRSKHMR
TVTNLFILNLAVSDLLVGIFCMPTTLLDNIIAGWPFGSMVCKMSGMVQGISVSASIFTLVAIAVDRFRCIVYPFKQKLTTS
TAVIIITVIWILAIAIMCPSAVMLQVQEEKHIRVILGYGNETRPVYWCRENWPNPGMRKIYTTVLFANIYLAPLSLIVIMY
ARIGIALFNTAMPTVGKHRQEQRHTVSKKKQKVIKMLIIVALLFTLSWLPLWTLMMLSDYANLSDIQLQIINIYIYPFAHW
LAFFNSSINPIIYGFFNENFRRGFKAAFKLQLCSKNIIHREVYSHRGQSNAILPAANYQTPQDQACQNVKAERKPIRKGNW
MNNQQDLMMEDLEEPYNNRMK
>ggNPY1R
MNASVLDPLGNNSSHLNFSEKNSQILQFEDEDCHVPLAMVFTLALAYGTVIILGVSGNLALIVIILKQKEMRNVTNILIVN
LSFSDLLVTIMCLPFTFVYTLMDHWIFGEAMCKLNPFVQCASITVSVFSLVLIAIERHQLIINPRGWRPNNRHAYMGIAAI
WVLATASSLPFLIYHVLTDEPFRNITFDEYKDKYVCLDLFPLDTARLSYTTTLLVIQYFGPLCFIFICYLKIYFRLKKRSN
MMDKMRDSKYRSSETKRINIMLISIVVAFAVCWLPLTIFNIVFDWNHEILPVATCSHNLLFLICHLTAMISTCVNPIFYGF
LNKNFQRDLQFLFHFCHFRSREEDYETIAMSTMHTDVSKTSLKQASPVAFKKINSDDDDKI
>ggNPY2R
MGPLEAIGEENQTDEMKMELFTKLYLPRYTTPVSELALDPKPELKDSTTLVEVQIILIFAYCSIILLGVIGNSLVIHVIIK

FKSMRTVTNFFIANLAVADLLVNTLCLPFTLVYTLLGEWKLGPVLCHLVPYAQALAVHVSTVTLTVIALDRHRCIVYHLES
KISKRISFLIIGVAWAVSALLASPLAIFREYSLIEIIPDFKIVVCSEKWPGEGQLNYGTIYSVSMLLIQYVLPLAIISYAY
TRIWTKLKNHVSPGAGNDHYHHRRQKTTKMLVCVVVVFAVSWLPFHAFQLVSDIDSQVLDLKEYKLIYTVFHVIAMCSTFA
NPLLYGWMNNNYRTAFLTAFQCEQRLDSIHPEVSAAFKARKKLEAKKSQFPGDSFTQPTNV
>ggNPY5R
MDLGFKDYTNRTPTKNTSATTKNFSAWEDYKSSVDDIQYFLIGLYTLISLAGFVGNLLVLTALTKRKQKTIINILIGNLAF
SDILVVLFCSPFTLTSVLLDRWMFGTVMCHIMPFLQCTSVLVSTLMLISIAAVRYRMVKYPLSSNLTAKHGYFLIVIIWAV
GCAICSPLPVFHKIVDLHKTLNLEALENRLLCIESWPSDSYRIAFTISLLLMQYILPLVCLTASHTSVCRSVGSRLSSKEG
KFQENEMINLTLHPSKSAGTEAQPSSHTSWSCALVRKHHRRYSKKTSTVMPAILRQQQDADFRDLPETSGTEKSQLSSSSK
FIPGVPICFEMKPEENTEIQDMITVSQSIIRIKTRSRRVFCRLTVLILVFGFSWMPLHLFHIVTDFNATLISNRHFKLVYC
ICHLLGMMSCCLNPILYGFLNNSIKADLMSLIPCCQIL
>ggNTSR1
VNTFISFVFPMVVISVLNTIIANQLMIMFKQAAQENQVCTIGGQQTMLSMSMEPGRVQALKHGVRVLRAVVIAFVVCWLPY
HIRRLMFCYVPSNHWTDFLYNFYHYFYMLTNVLFYVSSAINPILYNLVSANFRQIFLSTLTLVCLPWRKKKKRLAFTRKSN
SISSNHTFSSQVTRETTY
>ggPPY1R
MNKTRAVHDAFPFLNNKNLSSNRSFPSHLSNQCRNVTDLTVFLATSYSLETVLGIVGNICLIAVIARQKEKTNVTNILISN
LIISDLFMCLVCLPFTVVYTMMDYWIFGEVMCKMTSFTQCTSVTVSILSLVLIALERHQLIINPTGWRPSISQAYLGIGVI
WTLACLMSLPFLTTSILSNDLYEQLSHIMNFSYDKAICIDSWPSEQHRLIYTTTLLLLQYCIPLFFIILCYLRIYLRLQKR
KDMFEKSEYSNRAVQLRRINILLASMVAAFAVCWLPLHVFNTIVDWNYKIISPCHHNLIFSLCHLVAMASTCVNPVIYGFL
NSNFKKEVKSLILSCQHNSVTASIEDYDHLPLSTMQTEVSKGSLSLNCRHNSI
>ggPRLHR2
MAQLPNDSWQNNSAPLFTGLDLLLELKPLFIPLYATLVAVACTGNLFLILLIALTKKLHCTTNFLIGNLAAADFVMCLACV
PLTVSYAFEVRGWLFGMFMCYFVTLMQATTVFVSVLSLTAIAIDRYIVVAYPIHRRISRKSCVCVVACIWLLSILASVPTS
LHTQYLDLNAIGHDMIICEEFWKHKERERLLYSCLMLLLSYMLPLLAVSVSFCAISYHLQRRNIPGAAYPGRDKWSKTKQK
TFRVLTVSVVCFAVCWLPLQVVNFIRDIDEEFTILDKRYVNVIQVSCHLIAMSSACYNPFIYASLHDKFRFHLSSYFYRKK
KSSKHRVLQSFSVNTCSTLADAPAGALG
>ggPRLHR
MADDKRREMMNSDNLTSQSFLSAIHSNASNLFSGLQFVQSFKPLIIPCYSLVVFVGVIGNYLLIYVICKTKKMHNVTNFLV
GNLAFSDMLMCATCVPLTLAYAFEPRGWVYGRFMCYFVFLMQPVTVFVSVFTLTVIAVDRYYATVYPFRRRLTIPVCAYIL
AAIWLLSCTLAAPALVHTYHAEFPELDFSICEEFWFHMRRDRLAYAYSTLIITYVLPLAVISLSYLRISVKLKNRVVPGNV
TQGQAEWDRARRRKTFRLLVLVVAAFGVCWLPLHIFNMMKDIDISLIDKQYFNFIQLLCHWFAMMSACTNAFLYAWLHDSF
RGELKKMFAWRKKKIGPATNCIMASVVL
>ggPRLHR1B
MEPSGDGANASTYGLVLQSRPNGSTQFTGVQLIQSFKPLIIPCYALVVLVGIFGNYLLLYVICKSKKMHNVTNFFIGNLAF
SDMLMCATCVPFTLAYAFNPQGWIFGKFLCYFVFLMQPMTVYVSVFTLTAIAVDRYYATVHPLKKRLSLTSCIYVVGAIWL
LSCALVAPAVAHTYHVEFQQQGFAICEEFWMEQEEQRLAYAYSTLIVTYILPLSAVSLSYLCISVKLRNRVVPGHPTQSQA
ESDRLRKRKIFRLIVLVVAAFGVCWLPIHVFNIIRDIDIDLIDKHYFLLIQLLCHWFAMSSACCNPFLYAWLHDRFRGELR
KMFSCEPRILPASHCAAAVL
>ggTACR1
MDDPPPLEAELEHRWLLNASLNESSANQFVQPPWQVALWAVAYTLIVVVSVVGNVVVMWIILAHKRMRTVTNYFLVNLAFA
EASMSAFNTVVNFTYAIHNEWYYGLLYCKFHNFFPIAAVFASIYSMTAIALDRYMAIIHPLQPRLSATATKVVICVIWLLA
FLLAFPQGYYSVTEELPGRLVCLVAWPEHSTDVYGKTYHFCMTVLIYFLPLLVIGCAYTVVSITLWASEIPGDSSDRYHEQ
VSAKRKVVKMMIIVVCTFALCWLPYHIYFTLQYFNPEWYLQKFIQQVYLAVMWLAMSSTMYNPIIYCCLNDRFRVGFKHAF
RWCPFVSAAEYEGLEMKSARYLQTQSSMYKVSRIETTVSLAVGAAEEELEESKKGKRLSVDMTSNGSSRSDSKTVSESFSF
YSNTLT
>ggTACR2
MSTFPISNTSNISQVDYLEDDDNWTAVTQFTQPGWQIALWAITYSFIVIASIVGNVTVIWIILAHRRMRTATNYFIVNLAL
SDLLMSAFNTIFNFIYASHNVWYFGEEFCRFQNWFPITAMFVSIYSMTAVAAERYVAIIHPFKPRLPAXXXXXXXXXXXXX
XXXXXXXXXXXXXXXXXXXXXXXSSGKREEKTHHFYRKYHIAVIVLIYLLPLMVMFVTYSIIGITLWSSAVPGNHVNRVRY
KHQVNAKKKFVKTMVVVVIIFAVCWLPYHIYFILGSFKEDIYQQKYIQQVYLTIFLLAMSSTMYNPIIYCCLNQRFRSGFK
LAFRWCPCINATEKDKLKLTAPSLCQSTRRKARRSISNTETQFNEKEKTSLTLAQQTL.
>ggTACR3
MNSVSAANWNVSMSSRWNMSLAAAGDPWGQALGVPAGNASTPVAANFSNQFVQPSWRIALWSLAYGGVVAVAIFGNLIVIW
IILAHKRMRTVTNYFLVNLAFSDASMAAFNTLINFIYALHSEWYFGEAYCRFHNFFPITAVFASIYSMTAIAVDRYMAIID
PLKPRLSATATKVVIGSIWILAFLLAFPQCLYSITKVMPGRTLCYVAWPGGPKQHFTYHVIVIVLVYCFPLLVMGITYSVV
GITLWGGEIPGDTSDKYHEQLKAKRKVVKMMIVVVMTFAICWLPYHTYFIVTGIYQQLNRWKYIQQIYLASFWLAMSSTMY
NPIIYCCLNRRFRAGFKRAFRWCPFIELSSHDELELKAARFHPTRQSSLYAVSRMESSTTVVLDTNDGDNSHPSRKKKAAP
RNASFNGCSQRNSKAVSTASSFVSSLNTAGDDYS
>ggCCKBR
MEIVDASFLENSTNITALLCDILLENETFYCVDDPPYSSKDLHQIIRILLYCLIFLLSVLGNVLVITVLIRNKQMRTVTNT
FLLSLAVSDLMLCLFCMPFTLIPNLLKDFIFGSTVCKTATYFMGISVSVSTFNLVAISLERYSAICKPLQSRVWQTKSHAL
KVIAATWCVSFTIMSPYPIYSKLVPFTKYNNSTANMCRLLWPSDVIQQSWYTFLLLILFLIPGIIMMVAYGLISLELYRGI
KFDTSQRRSSRERKGSTSIAKYEDGDGCYLHKAKRKRKVPLQQLSTMSNSKIDRVRSSSSSANLMAKKLVIRMLMVIVVLF
FICWTPIFSVNAWRAFDTASADLHLSGAPISFIHLLSYTSACVNPIIYCFMNKRFRMGFLATFTCCAKQKPPVIRGEVGDE
EEGKTTRASLSKCSYTHMNMSAPP
>ggCCKAR
MDPRRLNESLQELLCRPGNGTGSANGTGTGPTWNGSACDLLRRGLRGPQAPKELDLTVRVLLYALIFVLSVCGNALVVAVL

LLNRRLRTVTNSFLLSLALSDLMLALCCMPFTLIPNLMGTFVFGEAVCKLMAYLMGVSVSVSTFSLVAIAIERYSAICNPL
QSRVWQTRSHACRVIASTWLFSALLMLPYAVYSTTRLLPVPGARPPLSQCTHAWPSEHVRQAWYVLLLLVLFFIPGVVMIV
AYGLISRELYRGIRFELDIKGEAAAQRSTEPLPTCDEGDGCYLQLSRPGGALELRALGAAGAQQERARINSSEAKLVAKRR
VIRMLVVIVAMFFLCWLPIFAANTWRAFAPRAAQRALSGTPISFIHLLSYTSACANPLIYCFMNRRFRSAFLGTCAGCRRA
CPRRAPDEDVANANASLSKFSYTTVSSLGPP
>ggGPR
MGHGASRAPLNGSEPAPFRAAALGARNGTTAQMAWGGGNGSAAGPWGGAERGGEPGYRHFATAAQLVIFVGSLLATSVLKS
VTNRFIKNLACSGICASLVCVPFDIVLSASPHCCWWIYTMLFCKIAKFLHKVFCSVTILSFPAIALDRYYSVLYPLERKIT
DAKSRDLVIYIWAHAIVASIPVFAVSNVSDIYAMSTCSESWSYSLGHLIYVVVYNITTVIVPVAVVFLFMILIRRALSASQ
KKKVIIAALRTPQNTISIPYASQREAELHAMLLSMVVIFIFCSIPYVTLVIYRTILNVSDISVFLLLTAIWLPKVSLLANP
LLFLTVNKSVRKCLVGMIVQLHRRYSRRNIVSSGGVGDANLEPGARSGSQLLEMFHIGQQQIFKPTEDEENETKSVGSSDF
QQKEIPVISSEVGETSVHKFIPQTIADSAAQVAPAVPAEADVENDKYSMQFGFGPFELPPQWLSESRNSKKRLLPPLGNTP
EELIQTKQPKCKAERKMSRNNKVSIFPKVDS
>ggGPR154
MLTDWLNPSYYPLTCLHDFFVFQMQTEQLVTLWILFIVTIAGNAIVLFSTWRRKRKSRMTFFVTQLAITDSFTGLINIMTD
IIWRYTGDFMAPDIVCRVVRYLQVVLLYASTYVLVSLSIDRYHAIVYPMKFMQGERQAKVLIGVAWSLSFLFSIPTLIIFG
KRQLSNGEVQCWALWPDDSYWIPYMTVVAFLVYFIPLIIISVIYSIVIRTIWMKSKAQAVIVSSCTDGKTSAGYTSRGFIS
RAKVKAIKYSIVIVLAFALCWSPYFLFDILDNFNILPETKERFYASVIIQNLPALNSAINPLIYCLFSNHLCRERRTRRLE
GTFRDRSDGGQEMQVLSKPEYI
>ggGPR19
MTVMLDHSTDNNASFVLPTLLLLLQNKSYPKTSLPAADHEMTESPQEPRPSRNRTVLQYELKPGEIVSASMVFGVLWLISI
FGNSLVCLVIHRSRRTQSTTNYFVVSMACADLLISVGSAPFVLLQLTSGRWTLGSVMCKLVRYIQYFTPGVQIYVLLSICM
DRFYTIVYPLSFKVSREKAKKMIVAAWIFDLAFMSPVFFFYGSSEEDHCNFFLPNSWEGAIYSIIHLLVVFFTPSILIILF
YQKVVKYIWRIGTDGRTVRRTMNIVPRTKVKTIKMFLMLNLVFLLSWLPFYVVQLWHPQETDYRESSLVFMAITWISFSSS
ASKPTLYSVYNANFRRGMKETFCMSTMKCYRSNAYTITTSSRIAKKNYVGISEIPAPAKTVTKDSIYYSFNREAKEKKLAW
PIHSNPPNTFV
>ggGPR39
MAGQTSSSDCSHLIDHSHISEFEVSPWIKITLALLDICIFVAGILGNSITIKATRILQKKGYLQKEVTDHMVSLACSDLLV
ILLGMPVEFFSAIWKPFATPNGNVACKLYYFLFEACSYATVLHVATLSFERYVAICHPFKFKAVSGPRKVKILIAFVWGTS
VIVALPLLFAMGTEYPLEIIENYQGVTACAKSTARHHLPELKQNMTICTSLSSKWPVFQASIFSAFAVYIIVLGSVAFMCR
SMMKTLMIHKKGTVAVKGEPGQQEQYLRKSESSEGKSSRRQIILFLLIVATLAICWMPNQIRRIMAAAKPKQDWTVPYFRA
YIILLPIADIFFYLSSVVNPLLYNISSQQFRSVFLQVLRCHLTIEHANKEKFLRANLSSRARSSRSLRPLLFISSRRSSST
RSNSKGLSTFQNEANPNCSLQKPGLELQEPSLEEVPLEMSSKPRPDAQNGLCEREV
>ggMLNR
MWGNGSRAEPWAAPPCDEWLCSALPLRALVPVTAVCLGLFAVGVVGNVLTVLVTCGHRDGRSTTDLYLGSMALSDLLILLG
LPLDLYRLWRSRPWIFGQLLCRLSHYLSEGCTYCTILHITALTVERYLAVCFPLRAKVLVTRRRVRAVIGALWAFALLSAT
PFFFLVGVEQPDNRTDFSRECKPTPRALESGLLGTMFWVTTSYFVLPVVCLSVLYGFIGRELWRSRGRLRGPGSALRERGH
RQTVRILGESPSPAAAPPRRLSLPGAVGSVPVRAPVCPRTAGGKAPFHRLAPLVAVVILAFVICWLPFHIGRIIFISTRDT
RTMLFSQYFNIFALQLFYLSASINPILYNLISQRYRAAVCRLLLPCRRMRRALVGTKVSRTYTETSSGIRHG
>ggGPR73_73L1
MVLEQDEHNPNATINLNFAAIYNLHDGDLTSMRNFSFPFNFSYSDYDLPLDSEDDMTKTRTFFAAKIVIGVALVGIMLVCG
IGNFIFIAALARYKKLRNLTNLLIANLAISDFIVAIVCCPFEMDYYVVRQLSWEHGHVLCASVNYLRTVSLYVSTNALLAI
AVDRYLAIVHPLKPRMNYQTATILIALVWIVSILVAIPSAYFATETVLFIVKNQEKIFCGQIWPVDQQMYYKSYFLFIFGI
EFVAPVITMTLCYARISRELWFKTVPGFQTEQIRKRLRCRRKTVMVLMCILTAYVLCWAPFYGFTIVRDFFPTIFVKEKHY
LTAFYIVECIAMSNSMINTMCFVTVKNNTMKYFKKIMLLRWRSTYHGSKSSTDLDLRTSAMPVTEEVDCIKLK
>ggGPR75
VVDSFCLGSYGNLIVFLSFLDPALRKFRTNFDFMILNLSFCDLFICGVAAPMFAFVLFFDSARGVPGAFCFTFHLTSSGFI
IMSLKTVAVIALHRLRMVLGKQPHRAAPFPCTLLLTLLLWATSFTLATMATLRTHGSRLCLPMSSFARGDGKVILYLYVTD
FICCVAVVSVSYVMIAQALRRNAQVRKCPPAVAVDASRPQPFVGPTAASAGQSALPALYRNQGSGKLQHSQAHGYTTHLGQ
PPAASRLQLVSAVNLSTTKDSRAVVTCVIIVLSVLVCCLPLGISLVQDMLSGSGGFVLYQFELCGFTLIFFKSGLNPFIYS
RNSAGLRRRALWCLQYLSLVFFCCKQKTRLQAMGKGSLEVNRNKSSHHETNSAYMLSPKPQKKFVDQACGPSHSKESVLSP
KASYGHQHYGQSTSTPMNTRIEPYYSIYNSSPSQEASTPNSLQPVNSTFGFTKSYIAMHYHTTTDLVRDYDSASAKPIPVP
SV
>ggAVPR1A
HGPDGLCRVVKHPLVFGMFASAYMLVAMTADRYIAVCHPLKTLQQPTKRSYAMIAAAWALSLLLSTPQYFIFSLSEVERGS
RVYDCWAHFIMPWGPRAYITWITGGIFVAPVLILATCYGFICFRIWRSARGRARPGEAAGGGPRRGLLLAPCVSGVKTISR
AKIRTVKMTFVIVSAYVVCWAPFFTIQMWSVWDQHFPWPLDSENTATTVTALLASLNSCCNPWIYMFFSGHLLQDCVQSFP
CCQKIKQTLSKEDSNSNSRRQTSFTNNRSPTHSLNTWRESPHSKSTSFIPVPT
>ggNPY7R
MLCCVPCSHFCCVCVPDLLLPSEGARPGMGLLEGANSTLTMEKLAVDEKLPQGWHTEDSVTPSLDLSGSHSVMMDSTKIPG
VQVVLIAAYSLIILLGFIGNSLVIYIIVKYKTMRTVTNLFIANLALADLMVDTLCLPFTLVYTLLDEWKFGAVLCHLVPYA
QALSVHVSTLTLTVIALDRYRCIVFHLDSRISRRLSFTIIAIMWLAAAILAGPLAIFREFRYEEIPSINLKMAVCSEKWPS
GNNRDATIYSLSMLLLQYVFPLAVICYAYTRIWFKLKNHVSPTSRNENQCRRRKTTKMLVMVVVFAVCWLPFHIFQLAID
LDLVLIFHEYKLLYTVFHVGAMCSTFVNPLLYGWMNKNYRNGFLMFFRCQNKPESIHAEGSVRGRSYIFRASTLNGSIKHA
AGNGPTEV
>ggOXTR
VEVTVLCLILFLALSGNLCVLLAIHTTRQKHSRMYFFMKHLSIADLVVAVFQVLPQLIWDITFRFYGPDFLCRLIKYLQVV
GMFASTYMLLLMSLDRCLAICQPLRSLHRRADRVSVLLTWLLCLLVSIPQIHIFSLRDVGNGVYDCWADFIQPWGPKAYVT

WITLMVYIIPVLMLSVCYGLISFKIWQNVKLKTAHGPPGGQSSTARGGAAFARVSSTRLISKAKIRTVKMTFIIVLAFIVC
WTPFFFVQMWSVWDTNAPQEGTSSPRGCVSAGTAALRAGGCGRQQCLGASGGWKGARRGSERGTAPGEAAAAAPELPSLQP
RVARAESFVFLRYRSVPADFSASNSSALPVPPLRSHPPERCAAAVWPWLEHRSSKALWFKNEVLPP
>ggGHSR_Ghrelin
ARGGTIGEGSSENRTGGESPLRLFPAPVLTGITVACLLLLVVGVLGNLMTMLVVSRFRDMRTTTNFYLSSMAFSDLLIFLC
MPLDLFRLWQYRPWNFGDLLCKLFQFISESCTYSTILNITALSVERYVAICFPLRAKVIITKRKVKLVILILWAVSFISAG
PIFVLVGVEHENGTNPLSTNECRATEYAIRSGLLTIMVWISSIFFFLPVFCLTVLYSLIGRKLWRRKRKNIGPSTIIRDKN
NKQTVKMLVVVVFAFILCWLPFHVGRYLFSKSFEAGSLEIAVISQYCNLVSFVLFYLSAAINPILYNIMSKKYRVAACRLF
GLKVLPKKRLSSTKQDSSRVWTEPTVAT
>ggNPY6R
MDKAIQHPTEILSNQTIPNSSYSQFLNFDTCQPSFPAVFLLITAYILVTTVGLFGNLCLIVIIKRQKEAQNVTNILIANLS
LSDVLICIMCIPVTAAYTLMDYWIFGEAMCKISSFIQSMSVTVSVFSLVLIAIERHQLIVNPRGWKPNTSHAYWGIVLIWV
FSLMISIPFLIFHQLTDEPFKHLSFHSDFYKNKAACIEAWPSVTERLIFTTSLLLFQYCFPLGFILICYLKIFLCLRRRHS
KIDRMRENESRLSENKRINLMLISIVVTFAACWLPLNIFNVVFDWNHEALMSCNHNLAFTLCHLVAMISTCINPIFYGFLN
KNFQKDLIGLVLHCRCSASQEEYENIALSNLQTDVSKGSLKLNNHPPVNV
>ggUTIIn1
MEPNGTAAAGDNGTAGGGGPPEGGPLLFPSDFSTVLAGMYMAGDWYFGDLGCRILLSLDLLTMHASIFTLTLMCTERYLAV
TRPLDTLKRSRGYRKVTAGAVWSVSLLLTLPMMLMVTLTEGSKAEGKVKRMCAPTWSMDAYRTYLTVLFSTSIMAPGIIIG
FLYTRLARTYLESQRNPPHKEKSKRSPRQKVLIMIFSIVLVFWACFLPFWIWQLVRLYSSPLQLTTQTQKCINYLVTCLTY
SNSCINPFLYTLLTKNYREYLRNRHRNFYRFTSSFRKRGSNLQCSWGRSMSSSNQYDYSSEALGMATLKDK
>ggCXCR5
MIRECAAPQWGGFGGDWVPLFRSCPPVTRLSVPQSLVELSGYYEAENTTPSLEGYFCFNPSSLWLANQRDPFRKVFIPLAY
LLMFVLGTVQNALVLVILERFKRSRTTTENFLFHLTLANLALLLTFPFSVVESLAGWVFGTFLCKILSAVHKINFYLHEHA
AGLHRVDRYLAIVYAIHTYRKARARSIHLTCTAIWLSSLLLTLPDLIFMEVWTDESNRSICYFPEAGIHGNNVWLATRFLY
HSVGFFMPLLVMCYCYMAIVRTLCQSQRLQRQKAVRVAILVTGVFLLCWSPYHIVIFLNTLTKLEAFAKDCLLEDHLDTAI
MVTEAIGFTHCCLNPIIYAFIGVKFRNDFFRILHELGCISQETLQEILEVTRKGCGIESDNTTSISTF
>ggC3AR
MYCLLICSLFYNYRMSPLLANNSSHVQDAVYYAPESISSLVIFILVFIIGIPGNGLVIWVAGLKMKRSVNIIWFLNLAVAD
FMCCLSLPFEIVHLFLNAHWPYGWFLCKVIPSVIIFTMFASVFLLTVISIDRCLLVMKPVWCQNHRTVKFISLVCSGIWIL
AFIICCPVFHYRTTITDGGKTECGYDFGDYEIPEYMDDSDPASGLLEDYSPLVPTVTYVNTIFAHQPTDTYPDFQANSIST
NKGIITAAHLSHATVDVNSLLNSSAYPDIRLLESQSDLPHTSLPVPSNNDFNLKLLDPPLDLADFDSVFGDNDYAVSLPLI
VITITRAVFGFILPFSIVAACYAFIAFRMRASNYRKPQNKMLKTIILVVVAFFICWAPYHTVGILSLVATPGTELKESLIL
WDHLSVALAYANSCINPLLYVFVGRDFRAKARQSLQGVLERVFTEEPTCSTPYSLDKSKTSKDKDISTTV
>ggRDC1
GEXTGKITHLIFSINLYGSIFFLACMSVDRYLSVAYFTNSSNRKKKIVRRCICILVWLLAFSASLPDTYFLKTVSTSNETY
CRPAYPEESFKEWLIGMELISVVLGFLIPFPVIALFYFLLAKTISASSDQERKSSGKIIFSYVVVFLVCWLPYHTTVLLDI
FYSLHFIPFSCQMENFLYATLHITQCFSLVHCCVNPILYSFINRNYRYELMKAFIFKYSAKTGLTKLIDASRVSEAEYSAL
EQNTK
>ggRLN3R1
MDELCESGSCSAATGMKEGADQEPWDTPLGLLEDSQMGNKSNVSFLQLFKNLNLERADGMQGDSSDVLRIVISLVYSVVCA
LGLVGNLLVLYLMKSKQGWRKSSINLFVTSLAVTDFQFVLTLPFWAVENALDFNWLFGKAMCKIVSYVTAMNMYASVFFLT
AMSVARYRSVASALKNQGRGDPLGGCCSTKWLCALIWLLAIVASLPQAIFSTTATVFDDVLCLVKFPEGQGSNAQFWLGLY
QTQKVLLGFVLPLAIISLCYLLLVRFISEKHVGSTCSGPSTKRRSKVTKSVSIVVLSFFLCWLPNQALTTWGILIKLNVVH
FSAEYFLSQVYLFPISVCLAHSNSCLNPILYCLMRREFRKALKSLLWRITSPSLTTMRPFTDTTKPEQEEQALHALVPVHP
VAPPAPAAAAVQPELAYYPPGVVVYSSRCDMLPTASGEQHY
>ggUTIIn2
MSLTDELESHFSVTPSMVTDTNESSLFRIRPNASANATWDGAPAAGSMEDMIAICTIGTILSLMCVIGVTGNVYTLLVMCH
YLRSSASMYIYIINLALADLLYLLTIPFIVGTYFIQKWYFGDVGCRILFSLDFLTMHASIFTLTVMSTERYFAVLKPLDTV
KRSKSYRKAIAVVIWLVSLLLTLPMLIMIQLVQRDNKSICLPTWSKLSYKVYLTILFGTSIVGPGVIIGYLYIRLAKIYWV
SQTASFKQTKRLPNQKVLYLIFTIVLVFWACFLPFWIWQLLFQYYESFTLSPKVMKNINYLTTCLTYSNSCINPFLYTLLT
KNYREYLKNRQRTLSSSSGYFQRRNRFQRISGRSMSTSSQHCTETYVLAHAPLGNSSA
>ggSSTR1
MLPDGTCTRLPGGAGSHSGGSHSGASGALEEAAARGMDSGGRNSSGAPNSTLSESQGSAILISFIYSVVCLVGLCGNSMVI
YVILRYAKMKTATNIYILNLAIADELLMLSVPFLVTSTLLHHWPFGSLLCRLVLSVDAINMFTSIYCLTVLSVDRYIAVVH
PIKAARYRRPTVAKMVNLGVWVLSILIILPIIIFSNTAANSDGTVACNMLMPEPTQRWLVVFVVYTFLMGFLLPVVAICLC
YILIIAKMRMVALKAGWQQRKRSERKITLMVMMVVMVFVICWMPFYIVQLVNVFVEQDDATISQLSVILGYANSCANPILY
GFLSDNFKRSFQRLLCLSWMDNAPEEPIDYYATALKSRAYSVEDFPPDNLESGSMYRNGTCTSRITTL
>ggSSTR2
AFILGFLVPLTIICLCYLFIIIKVKSSGIRVGSSKRKKSEKKVTRMVSIVVAVFIFCWLPFYIFNVSSVSVLIVPTPVLKG
MFDFVVVLSYANSCANPILYAFLSD
>ggSSTR3
MDTSAFSLPTPTVSEEGNASGSWAGFTTPNSSTTTTSPGVVVSGVLIPMVYLIVCVVGLIGNSLVIYVVLRHSVSESVTNV
YILNLALADELFMLGLPFLAAQNALSYWPFGSFMCRLVMAVDAINQFTSIFCLTVMSVDRYLAVVHPGKSSKWRTARVAKA
VSATVWMLSSIVVLPVVVFSDVPLGMNTCHIQWPEPASVWRAGFIIYTATLGFFGPLLVICLCYLLIVVKVRSSGRRVRAL
SSKHKLSERRVTRMVVTVVAVFVLCWLPFYVLNIINVVCPLPEEPSLFGVYFLVVVLPYANSCANPIIYGFLSYRFKQGFR
RAIFRPSRRVQSQEVPECPPEKSDDGGEEKEISKITQNGNDREERPLSSRAGERNGQKPLPEEPVGCEKSSKLHVSYL
>ggSSTR4
MSTNAEHLPPWAASAVPPNASTAWMEGPSARGGRVGELAGMVVIQCIYALVCLLGLLGNSLVIFVILRYTKMKTATNIYLL

NLAIADELFMLSIPFVATSAALHHWPFGRALCRTVLGVDGLNMFTSVFCLTVLSLDRYIAVVHPLRAATYRRPRVAKMVNG
AVWLLSLLVASPIPIFAGTAVTRDGQAVACNLLWPSPAWSAAFVVYTTLLGFLLPVLAMGLCYLLIVGKMRAVAQRVGWQQ
RRRSEGKLTRLVLMVVAMFVVCWMPFYVVQLVNLLLPGRLDATVNNACLILSYSNSCANPILYGFLSENFRHSFHGVLRRC
LDASLCCCPPEARAVGDEEEEEEEEPLDYRAVPRSDPKGKGCMCPPLPCQQEPARPEPFCKPGTLLAKTTTF
>ggSSTR5
MDPLYFSNAFNMDSSSSDLNSSLLTNTTENGTLSEQLPFKYIHKVLIPIFYILVCAVGLSGNTLVIYVVLRYAKMKTVTNI
YILNLAVADVLFMLGLPFLATQNAISYWPFGSFLCRLVMTVDGINQFTSIFCLTVMSMDRYLAVVHPIKSTKWRRPRVAKL
ISMTVWTFSFLVVLPVFIFSDVQEDFHTCNMNWPEPVNIWSAAFIIYTSVLGFFGPLLVICLCYLLIVIKVKSSGIRVGST
RRRRSERKVTRMVVIIVVVFVFCWLPFYMMNIVNLIFILPEDPVLVGVYFFVVVLSYANSCANPILYGFLSDNFKQSFQKV
LCLRKGNGVEDGDPIEHRQENSSRLQESMLTQRNIEFNGHMQTSKV
>ggOPRL1
MDPLFPAHILEDPDLRKLLNDSSMLNLSFLPSNWFNNGTGDSFLPLSIKITIVVVYSIVCIVGLVGNCSVMYVIVRFTKMK
TATNIYIFNLALADTLCLMTLPFQGTDTFLGFWPFGNVLCKIAISIDYYNMFTSTFTLTMMSVDRYIAICHPIKALDIRTP
HKAKVVNVCIWALASVFGIPAMVMGSAENENNEIDCLIKLPSPVDYWDPVFGICVFLFSFMIPVLIITICYSLMIRRLKNV
RVLSGSKEKDRNLRRITRMVLVVVAVFIICWTPIQIFVLVQCLGAKAESELELAISCFCTALGYANSSLNPVLYAFLDENF
KACFKKFCFPTAFRTELQMSNRMCSIAKDVAYACKNSEGTNNPA
>ggOPRM1
MAVAYLLGNGSAPLLAGALEVPFGANASAAACRPAVPPCAAAPPGAWGNSTAGWNRSEPCGGANGSAGGGPCAPAGGGGPS
VVTAIAIMALYSVVCVVGLFGNFLVMYVIIRYTKMKTATNIYIFNLALADALATSTLPFQSVNYLMGTWPFGTILCKIVIS
IDYYNMFTSIFTLCTMSVDRYVAVCHPVKALDFRTPRNAKIVNVCNWILSSAIGLPVMFMATTKYRQGSIDCTLTFSHPAW
YWENLLKICVFIFAFIMPVLIITVCYGLMILRLKSVRMLSGSKEKDRNLRRITRMVLVVVAVFIICWTPIHIYVIIKALVN
IPETTFQTVSWHFCIALGYINSCLNPVLYAFLDENFKRCFREFCIPTSSTIEQQNSTRVRQNTRDHASTANTVDRTNHQV
>ggOPRD1
MELNTELPTEPPAAWDNRTAWDALPGAGGNETGPPRAKNATSILIAIVITALYSVVCVVGLLGNVLVMYGIVRYTKMKTAT
NIYIFNLALADALATSTLPFQSAKYLMETWPFGELLCKLVLSIDYYNMFTSIFTLTMMSVDRYVAVCHPVKALDFRTPAKA
KVINVCIWVLSSVIGVPIMVMA
>ggOPRK1
MDAPVQIFREELDSTCMPGPCRPSSTSSSWPLGWADYDSNATGTFRDTQHNSTSISPSIPIIITAVYSVVFVVGLVGNSLV
MFVIIRYTKMKTATNIYIFNLAMADALVTTTMPFQSTEYLMNSWPFGDVLCKIVISIDYYNMFTSIFTLTMMSVDRYIAVC
HPVKALDFRTPLKAKIINICIWLLSSSVGISAIVLGGTKVREDTGSTECSLQFPDDRDYVWWDIFMKICVFVFAFVIPVLII
IVCYTLMILRLKSVRLLSGSREKDRNLRRITRLVLVVVAVFIICWTPIHIFVLVEALGDVSHSTAAVSSYYFCIALGYTNS
SLNPILYAFLDENFKRCFKDFCFPFKMRMDRQSTSRVRNTVQDPAYRREADGTNKPV
>ggGALR1
MEPGEPLNRSQEGAEAPRGEFNASGLPEEEGKPLFGIGIENFITLIVFGLIFALGVLGNSLVITVLARSKPGKRRSTTNIF
ILNLSIADLAYLLFCIPFQSTVYVLPTWVLGAFICKFIHYFFTVSMLVSIFTLSAMSVDRYVAIVHSRRSSTLRIPRNALL
GVGLIWALSFAMASPVAHHQRLFHRNGSDQTFCWEHWPNPRHKKVYVVCTFVFGYLLPLLLISFCYAKMLVLNHLHKKLRN
MSKKSEASKKKTAQTVLVVVVVFGISWLPHHVIHLWAEFGVFPLTQASFLFRIAAHCLAYSNSSVNPIIYAFLSENFRKAY
KQVFKCQIGNESPLNDAKENKSRIDTPPSTNCTHV
>ggGALR2
MNGSAAGSDEGWHPESVLIPLAYLLIFLVGTVGNCLVLAVLLRNGQVKNTTNLFILNLGVADLCFILFCVPFQATIYTLEG
WVFGSFLCKAVHFFIYLTMYASSFTLATVSLDRYLAIRYPLHSRELRTPRNALVAICFIWALSIIFSSPYLSYYQQLQLAN
LTVCHPVWATSQRKVMDLCTFIFSYIIPVLILSLTYTRTIRYLWRSVDPLQEMSESKKAKRKVTRMIVIVAVLFCLCWLPH
HLVILCFWFGYFPLNHTTYVLRIVSHLISYANSCVNPIVYALVSKHFRKGFKKIFICLLHKKAANKVHVAQGTNTVSMLEA
ELSEVTRLSDAGPGCSSVRCKAQPWGEAALGGCQQRADGSFITFDVT
>ggGALR2n1
PASVIVPVVFSLIFLLGTVGNSLVLAVLLRNRQMGHNTTNLFILNLSLADFFFIVFCVPFQATIYSLEGWVFGSFICKAVH
FFIYLTMYASSFTLAAVSVDRYLAIRYPLRSRELRTPYNAVAAMAVIWGLSVVFAGPYLSYYDLIEWESSYICMPGWEEWR
RKVMDTSTFAFGYVIPVLVISLSYTRTIKYLWTAVEPLEDMSESKKAKRKVTKMIIIVTILFCLCWLPYHVVILRYLYGDF
PFNQATYAFRLLSHCMAYANSCLNPIVYALVSKHFRKGFKRVFSCLLRKKHRNKVHV
>ggMCHR1
YFRVIMPSLFSIICFLGVVGNLIVISTIVKKKKLKRNQTVPDIFIFSLSIVDLLFLLGMPFLIHQLLGNGSWYFGATLCTI
ITALDNNSQITSTNILTVMTLDRYLATVYPLGSTYVRTPCVAALVICLVWLLSFLTVIPVWMYAGLMPLADGTVRCALLLP
NPETDIYWFTLYQFMLPFAMPLIVICVGHLQILQHITTTVVPLPQRSLQVPT
>ggCCR2_5n1a
MENYTDLGDMPTTTFDYGDTAPCMGTEEKHFAANFLPPLYSLVVIFGFIGNILVVLILVKYKKLKSMTDIYLLNLAISDLL
FVFSLPFWAYYAAHDWIFGDALCRILSGVYLLGFYSGIFFIILLTVDRYLAIVHAVFALKARTVTYGILTSIVTWAVALFA
SVPGIVFHKTQQENTQCTCSFHYPSDALINWQHSYILKMNILGLIIPMIIMIFCYSQILRTLFGCRNEKKQKAVRLIFVIM
IFYFIFWTPFHVASFVHTFQTSFFSPDCDSQSRLEKTIQVTETISMVHCCINPVIYAFVGEKFRKYLHMFFRKHVATHLCK
KCPSLYREKLERVSSTFTASTAEHDISTGL
>ggCCR4
MSSSSTESLEADTTTFYDFIDNYNDAPQPCSKENFKRFAASFFPVLYTLVFLIGLIGNTLVIVVLFKYKRLKSMTDVYLLN
LAISDLLFVLSLPFWSYFMIDQWVFGTPWCKIISWIYLVGFYSGIFFIMLMSIDRYLAIVRAVFSMKARTAFHGLIASLTV
WLVALLASVPELVFRESFVEQNYTTCKLRYPSNYLTWKLFYTLEINILGLLLPLIVMAFCYSMIIKTLLHCRNEKKNKAVR
MIFAVMIVFFFFWTPYNIVILLQLLEATGVIRNCQASRNLDYASQITESLGLFHCCLNPVIYFFMGEKFKKYLKMLFKNWQ
LPGDICKWCGLHITYHTESTGSFHTQSTGDQEAL
>ggCCR6
TEFFDTDYASLISTVCSKSEVRSFTKVFLPVAYSLICIVGLVGNIFVVMTFALYERTKSMTDVYLFNMAIADILFVLTLPL
WAVNYAADKWIFGNFICKMAKGIYAINFSCGMLLLAFISVDRYIAIVQATKSFKLRARTLAYSKLICLAVWASAILISSSS

FLYSESYGFATNETQICDHRFDKTSDSIVLKSLLLCLQVGFGFFIPFVFMIFCYAFIVKSLQQAQNSKRNKAINVIVLIVV
VFLVCQVPYNTVLLMAVANMGKTEKSCDSDNIMAYAKYTTETIAFLHCCLNPVLYAFIGVKFRSYFVKIMKDLWCRRYKKY
NKRSSRINSDIYLSRQTSEILTDNASSFTI
>ggCCR7
SLLFCAGNNVTDDYDANTTIDYNMFEMMCEKKEVRDFRAAFLPAMYSLICFTGLLGNGLVMLTYIYFKRLKTMTDIYLLNL
ALADILFLLTLPFWATSAATFWCFGEFACKAVYCICKMSFFSGMLLLLSISIDRYFAIVQAASAHRFRPRMIFISKVTCIL
IWLLAFVLSIPELVHSGVNNYDSHPRCSIIASDLQTFSTGIKVSQMVFGFLVPLVVMSVCYLIIIKTLLQARNFEKNKAIK
VIIAVVIVFVVFQLPYNGVMLAKTISVFNNTSSCDESKKLDMADDVTYTLACFRCCLNPFLYAFIGVKFRNDLFKLLKELG
CLSQQRLWQLSSCRESKRFSFAMETETTTTFSP
>ggCCR8a
MEQRKKPTGRHTRALYLWFFPSQESKMNPTDLFLSTTEYDYGYDENTAPCNEGNSFPRFKSLFLPILYCLVFVFCLLGNSL
VLWILLTRKRLMTMTDICLLNLAASDLLFIVPLPFQAYYASDQWVFGNALCKIMAGIYYTGFYSSIFFITLMSIDRYIAIV
HAVYAMKIRTASCGTMISLVLWLVAGLASVPNIVFNQQLEIEQSVQCVPVYPPGNNIWKVTTQFAANILGLLIPFSILIHC
YAQILRNLRKCKNQNKIKAIKMIFIIVIVFFLFWTPFNVVLFLDSLQSLLIIDNCQASSQITLALQLTETISFIHCCLNPV
IYAFAGVTFKAHLKRLLQPCARILWSPTRGSGVTQSSLVLSQISGCSDSAGVL
>ggCCR8b
MYYPELATDCEFESIPAFASSFFPVLYSILFVIGLMGNALVVWVLTAFKKIRAMTDVYLLNLAISDLVFVFSLPFLAQYSL
VSQWTFGNAMCKIVSSAYFIGFYSSAFFITIMSIDRYLAIVHSVYALKVRTTKHGIIASLALWAVAILASVPGLVFFREVD
EDNRTQCIPHYPGSGNSWKVFSNSEVNILGWLFPVSILIFCYHNILRNLQRCHTQNKYKAMKLVFIVVIVFFLFWTPINIM
LLLDSLRSLHIIDDCQNSQRLDLALELAETLSLVHCCLNPIIYAFVGEKFKKYLCEAFGKYAHFLLICKGHSAFNRRNTDR
RTSMYTASSQSSFVGSVL
>ggCCR9
MTSLDYYRNDSAGLSVIGNPINDTELMCDRRQVWQFARAFLPVFFWLIFFVGTVGNALVVLIYCKYRFRRSMMDRYLLHLA
VADLLLLFTLPFWATAASSGWIFRNFMCKVVNSMYKINFYGCILFLTCISFDRYLTIVQATKAKSSKQRRILRSKVVCFAV
WLASVSLCLPEIMYSQSKQIGAVTVCKMTYPPNIGMAFRVAVLVLKVTIGFFLPLLVMVICYTLIIHTLLQAKRCQKHKSL
KIITMIITAFLLSQFPYNIVLLIKTINMYTGAVYSCQTINGLDIGLQVTQSIAFLHSCLNPFLYVFAGERFRMALARMVQS
TGRYWLGGQDQCSSLGDSQEHSSNWSFAMLGRRRVRNSLTLSTNLASSVVPASCQVFV
>ggCCR2_5n1b
MTDIYLLNLAISDLLFIFSLPFWAYYAAHDWIFGDALCRILSGVYLLGFYSGIFFIILLTVDRYLAIVHAVFALKARTVTY
GILTSIVTWAVALFASVPGIVFHKTQQEHTRYTCSAHYPQEQRDEWKQFLALKMNILGLVIPMIIMICSYTQIIKTLLQCR
NEKKNKAVRLIFIIMIVYFFFWAPYNICILLRDFQDSFSITSCEISGQLQKATQVTETISMIHCCINPVIYAFAGEKFRKY
LRSFFRKQIASHFSKYCPVFYADTVERASSTYTQSTGEQEVSAAL
>ggCCR11
MGWVVNNSTDYWIEDDEHYLNSDVDYNTYEFLCEKEDVRKFMKVFLPVFYALTFTVGVAGNSLVVAIYAYCKKPKTKTDVY
IMHLAIADLLLLFTLPFWAANAVQGWELGNSMCKLTSSLYTMNFSSSMLFLACISLDRYRATSESQRHRRAGKHCSVTCIC
VWLSAVLLSIPELIFNQVITHNNRNECLPIFPTNMETLLKATIQILEVILEFLLPFLVMVTCYSVTARAIFRSANAKKSRP
FMVLLAVVAAFIITQLPYNIIKFWRAIDSIYLLITDCEASKTIDVALQVTKSIALFHTCLNPILYAFLGASFKMRVMKIAK
NYGYWRRQQQHEVPEEISMNSEARTEETISFTI
>ggCXC3R1
MTEAPPEVTTEYVFYESALACDESDIQAFGKIFLPLFYIAVFALGLAGNVMVVLAIVKEGSKKSITDIYLMNLAVSDLLFV
ISLPFWASNTVRGWTLGTIPCKVVSSLYYIGFFGGMFFITVISIDRYLAIVRATYSMRSRTIKHSLLITCGVWATAVLVSV
PHFVFSQMFENDCIPVLPQELMNIWPVFCNVELNTAGFFIPVCIICYCYCGIIKTLLYCKNQKKARAIKLTLAVVIVFFLF
WTPYNVLIFLETLRHYELFISCNQIKSLDYAMHLTETIAFSHCCLNPLIYAFAGEKFRKYLHRVCFKYCPCLCFCGPCNHY
DVRPSVSYAESMVNSNITLNTSDQDGTVFL
>ggCXCR4
MDGLDLSSGILIEFADNGSEEIGSADYGDYGEPCFQHENADFNRIFLPTIYSIIFLTGIIGNGLVIIVMGYQKKQRSMTDK
YRLHLSVADLLFVITLPFWSVDAAISWYFGNVLCKAVHVIYTVNLYSSVLILAFISLDRYLAIVHATNSQRPRKLLAEKIV
YVGVWLPAVLLTVPDIIFASTSEVEGRYLCDRMYPHDNWLISFRFQHILVGLVLPGLIILTCYCIIISKLSHSKGHQKRKA
LKTTVILILTFFACWLPYYIGISIDTFILLGVIRHRCSLDTIVHKWISITEALAFFHCCLNPILYAFLGAKFKTSAQNALT
SVSRGSSLKILSKSKRGGHSSVSTESESSSFHSS
>ggCCXCR1
MDEEQYPSGWDDNYSFEYVLNESNVCEMGNYFIFYTHFTTVLYTLAFLLSLLGNTLVLWILFKYENLTSLTNIFIMNLCIS
DLVFSCMLPFWAVDQTFGWIFGEFLCKAVNAIFSIGYYSGVFFLTLMTILRYLSVVSPLSTLRSQTQYCGSLVCLLVWTCS
ILIVVPEMIHTTVVETLEEVSTCDYDDWKWKKVDIYQRNILFLISFGIIIFCYINILIILLRTRSRRKHRTVKLILVIVVA
FFLSWAPYNILSFLITFPPPTCQYEKDTLLAFHISRKIAFSHCCLNPVLYVFAGVKFKSHLFRLCGQYLPCCDEVSRIGSQ
AKFHYEDASIY
>ggCMKLR1
MALSNLSEYSDDSDTYDYLDYTYEEPGSVWTGPSYDPKDVARILSVVIYSVSCVLGILGNGLVIAIITLKMKKSVNAIWFL
NLAVADFLFNIFLPINIAYTAMRYNWIFGTVMCKLNSFLLILNMYTSVLLLTTISFDRYVSVVFPVWSQNHRSTNLACMVC
LIIWMVGIIMSCPSLVFRDTAQARNSIICFSNFSLSRNRSYQALALVRHRTVNITRLFAGFLLPITIITFCYVSIALNLRR
NRLAKSKKPFKIIVTIIVTFFLCWSPYHLLNILETEPDLTPRSVFEIGVPITTALAASNSCMNPVLYVFMGQDFKKFKVTI
LSRLVNALSEETGHSSIVHRSFSKMSSMTEKETTVL
>ggC5L2
MSSPVPWHTPTPFPDYSDLNNVTFNDIGGHHRIPWTHRVALALYAAIFLLGIPGNAAVVWLSSAAQRHAVPAVWFLHLAVA
DLLCCAALPFLAIPVAGDHRWPLGDFGCKLFPALAVLNMYASVLLLATISADRCLMVTRPAWCHNHRTPRAAHRVCLIVWS
AAAVLTVPTFLFRATSQDPFADAEMCVVDYGAARQHRRFAEAFTAALRFAAGFLLPFSAITACHAFLLARLHARHFTRTRR
AAGTVLAVIVTFFVCWLPYHVVGLILAATAPGAALYQGAMAADPVLTGLAYANSCANPLIYAAAGRRGGWGGGGRGTGAW
CAALRRVLSEEPNSSANTGSRSAAHVATIDTEV

>ggCCBP2
XNSSDYPYEYLNEEDYILYGVCTKEEVVSFSKAFLPAFYTVVFLIGFTGNVLLFTVLMYISKKKKMAEVYLLNLVVSDFLL
LLTLPFWALFISQWVTWDLLCPVLNAMYIMNFYSGIFFVSCMSLDTYLQIVHACSPHSSVTRKKSFLLLLMLWVLSILLSI
PDALFSSTKEMHNKTIVCTHDYGQKHLFWKVVFQVTQNILGFLLPFFFMVFCYSRSMCVLTTSRVPGSRTALRFVFILVAV
FFVLWFPYNVVLILHSLQFVGLIQSCERSRQLDYAIQITESLSFVHCCLNPVLYAFVKKRFRLYLQKIPQAFCRKSTFDIQ
LSETSCSCSRYIAQIEMLSITNT
>ggGPR1
MTFEDFENYSYFYDLPEEEESPQSTLSIAHMISLSFYSVAFLLGVPGNAIVIWFMGFKWDKSVSTLWFLNLAIADFIFVLF
LPLYITYVAMGFHWPFGKWLCKMNSFIALLNMFASVFFLTFISLDRYIRLVHPVFSYKYRTIKNTLILSVIIWMSAAVIGG
PALYFRDTATALNNVTICYNNFHVHDRELILLRHHILIWVRLAFGYLFPLLTMVICYSLLIVKVKRRTVLTSSRLFWTIVA
VVVAFFLCWTPYHIFSIVELSAHHDENLHDLLQDGIPLSTGLAFINSCLNPILYVLISKKFQAQVKTTVSEVLKLALWEVS
RSGTVSEQLWSSENTHAPVHHCETAQ
>ggGPR15
MRTAGPEMDLIKLSPMTTVTFNYDDYYEDSCQYEQLQHMTAFLPVLYAAVFLVGILGNSILIIALVFKRRVQRLIDIFIIN
LAASDFIFLITLPFWVDKEASDGIWRVGSFLCKASSYIISVNMYCSILLLTCMSADRYLAIMYPSIARKVRTRSYSSGLCF
CVWLLSCCLGMPTLLSRELTERYGKMYCTDKAMTESKQITSLMILILAFFFPLLSILTFYCSITRRLCVHYQRSGKHDKKL
RKSIKIVFIVVAAFVISWVPFNLFKLMAILLGLQKLPDCFLGMVAQVGMTVSSPFAFANSCANPFIYYCFDNYIRRAMLRC
LCPWVKASSGSTISDTMDTRLSHSLSNFITGDYTARKRKRSVSL
>ggCCR2_5n1c
MENYTDLGDMDVTTTFDYGDTAPCMGTEEKHFAANFLPPLYSLVVIFGFIGNILVVLILVKYKKLKSMTDIYLLNLAISDL
LFIFSLPFWAYYAAHDWIFGDALCRILSGVYLLGFYSGIFFIILLTVDRYLAIVHAVFALKARTVTYGILTSIVTWAVALF
ASVPGIVFHKTQQEHTRYTCSAHYPQEQRDEWKQFLALKMNILGLVIPMIIMICSYTQIIKTLLQCRNEKKNKAVRLIFII
MIVYFFFWAPYNICILLRDFQDSFSITSCEISGQLQKATQVTETISMIHCCINPVIYAFAGEKFRKYLRSFFRKQIASHFS
KYCPVFYADTVERASSTYTQSTGEQEVSAAL
>ggAGTR1
MVPNYSTEETVKRIHVDCPVSGRHSYIYIMVPTVYSIIFIIGIFGNSLVVIVIYCYMKLKTVASIFLLNLALADLCFLITL
PLWAAYTAMEYQWPFGNCLCKLASAGISFNLYASVFLLTCLSIDRYLAIVHPVKSRIRRTMFVARVTCIVIWLLAGVASLP
VIIHRNIFFAENLNMTVCGFRYDNNNTTLRVGLGLSKNLLGFLIPFLIILTSYTLIWKTLKKAYQIQRNKTRNDDIFKMIV
AIVFFFFFSWIPHQVFTFLDVLIQLHVITDCKITDIVDTAMPFTICIAYFNNCLNPFFYVFFGKNFKKYFLQLIKYIPPNV
STHPSLTTKMSSLSYRPPENIRLPTKKTAGSFDAE
>ggAGTR2
MQSNYSMGITTRETLQILYTAQANSSAVLRSPPPCPLTSSSYQFSLIPAFFSVVFALGLVGNSVVVVVLCHRSSPRTVANI
YIFNLAMADLLCLATLPFWATYYAQGYNWIFGSLMCKISSSVLCLNMFASIFFITCMSVDRYHAIVHPIRSQRRTPHQAYF
IALVVWGLACLSSLPTFYFRDTHYIKSMEVKACIMAFPHESYAEWSVGTAFLKNALGFFIPLAVITTCYVWIRRHLIQAQE
FGKNKQKRDRVLKLVAAVVVAFLICWLPFHILTFLDALAHMNIIDNCDVTGVIDTALPFGICMAFANSCVNPLVYCFIGNQ
FQEKLHRLFKRRVYQFNSQRESSSLRKGSCFRDTEIAVGREREPESPL
>ggAGTRL1
MGRRGGELLNFPRGLPPXXXXXXXXXXXXXXXXXXXXXXXXXXXXXXXXXXXXXXXXXXXXXXXXXRERRRSADTFIAHLAAADL
TFVVTLPLWAAYAWLGYHWPFGTATCKVSSYLVFVNMYASVFCLTGLSFDRYLAIVRPLATAKLRSRVSGLLATVALWVLA
ALLALPALVLRQAATLGGDTKVTCYMDYGGLAALGTEAAWEVGLGLSSTLLGFVAPFAVMLTCYFFIARTVATHFRRGAGR
GAPQAQAAADHHRRLVAAFGGCWLPFHLVKTLYILMELEALPWSCSLYNFLNNFHPYCTGIAYINSCLNPFLYAFFDPHFR
RACAALLCCHPPGPGAERSGSFSSGHSHPPGGKGALAMGGKLDPATQETLFRTL
>ggBDKRB2
MITITTENVTQLYNVTATQEFTISPAEFHNNSSVHQMDEYKCINPDTWKWLQTFQPGFLWIIFILGTIENAFVLIVLYFHK
SRCSVAEIYLANMAFADLMLVCSLPFWAINISNGFQWPFGLFLCKAVNTINYMNSYSSIYFLTLVSIDRYLALVKTMSLGR
MRRTACAKWNSLIIWMCALLICSPTMVFRNLQYFKEYNITACSLDYPTPYWHPANNCLLNAVGFMIPLCIITYCTTQIIKA
LQSNKSQKLKLIQTERKATLLVLAVLLLFVICWLPFQISTLIDTVCYLAGNLKCLEDINDIVTQMAIYCAFSNSCLNPILY
VIVGKHFQKKAVELFKDLIPQRCRKSESVKIENSQDTLRTSISSEHLRKKSVLPLSQ
>ggCMKLR1n1
MESVSSSPSPFPDSSPSVPSGNATAHDHYSGLQKSLHVLSMVVYSIACVLGVTGNGLVIWIAGFKMKKTVNSVWFLNLAIA
DFIFTFFLPLSIAYTALDFHWPFGKLLCKLNSTIAFLNMFASVFLLMVISVDRCVSVAFPVWSHNRRSPELAARVALGTWG
MAVLLSSPYLIFRDTAVSSRNITSCYNNFALSDDYTAEETRRLWRMRHKAMIITRFLCGFLIPFTVILICYSIVAVKLKRR
QLASSAKPYRIIIAVTVSFFLCYFPYHVFSLLEISQNSSSHEMKMALYIGIPLVSSLAFFNSCINPILYVFVGPDFKEKFC
QSILSTFEGAFTEESMLGSLSSRRKSRSASEAEVPRL
>ggUTS2R
MEPGACTPELCYPKNGSTQLNATSDTASDLLATSFLGCILGTMCVVGAAGNIYTLVVTTVSMRRSGSMYVYIINLALADLL
YLSTIPFVVCTYFVRDWYFGDLGCRILFSLDLLTMHASIFILTIMSTERYLAVARPLATLRGSRDRRRALTFLAWLVAFLL
ALPSMILIDLRTSHRHGVTKRICHPTWRMGPYKVYLTVLFNTCILAPGVTICCVYTKLARTYWKSQRALVAHAKHASHCPK
QKVLYMIFSIVLAYWACFVPFWLWQLFSLYWHERGERGSSTVVPINFLVTCLAYSNSCVNPFLYTLLSKNYSEYLRRHHRA
AQRGSSAQPPAPVPPLGGSGEDDCGL
>ggBDKR1
MTESPLLSIPFSNQTNTTFCPDLDNWWEIVYYLVPKCIDAICVIGMLGNLFVLFVYSLYKGPLKIAEIYLVNLAVADLIFL
TCLPFWAENIRNKYNWPFGNFLCRSASTSIHLNMYTSIYLLVAVSLDRYLTFVHTLTHRRIRSSTTAKGICLLIWIFGILL
SIPTFTFRTVKHFPELNVSACALDFPTPLWGMANRLVFNIVGFALPSTAIVFLNFCSIRSLQEHTREQRALRTKSFKDLRD
TKATRLIFLVVLMFLLCWTPYHLVIFLDILFRMEVIQGCFWEYFLNFSEQFCSTLALANSCINPIVYVFAGKYFRQKALKV
FSQFFCCGYFLSQVSFQEKSSYFNLFPVKNSSTS
>ggGPR7
MTSLVGHTWEQKGRHQWLNTLEEASLRITRFLVFLSFFQALLATSRSGPSAVTMENTSLPEPNSSCVAGHTDCTRRGDVEL

SIPTPPPSPSSYITMPVIYSIICAVGLTGNTAVIYVILKAPKMKTVTNIFILNLAIADELFTLVLPINIADYLLLQWPFGE
FMCKLIISIDQYNIFSSIYFLTVMSVDRYLVVAATTKSRKMSYRTYRAAKIVSLCIWSFVTVIILPFTVFAKVHKEQGRSQ
CVFVFPHPESVWWKGSRIYTLILGFAIPVSTICILYTIMLCRLRRVHLHSNAKALDKAKKKVTLMVVIILAVCLLCWTPYH
LSTVVALTTDIPQTPQIVGISYFITSLSYANSCFNPFLYAFLDDSFRRSFRKLMDCRTTS
>ggCMKLR2
METYSASVPPVLCNSTTFNLSGSHLCNESLSSRLADKSEHQQYVIGLFLSCLYTIFLFPIGFVGNILILVVNISFREKMTI
PDLYFINLAVADLILVADSLIEVFNLDEKYYDITIICTFMSLFLQINMYSSIFFLTWMSFDRYIALAKVMRSNLFRTMQHA
RLSCGLIWMASISAALVPFTAVHLQHTGEVYFCFADVREIQWLEITLGFIIPFVIIGLCYSLIVRVLIKAHKHRSLRLRRQ
KALRMIFVVVLVFFICWLPENVFISVQLLQKKSEPTSSSSPSFRHDYPLTGHIVNLAAFSNSCLNPLIYSFLGETFRDKLR
LYIEQKTKMSTLHRFCQAALTSVIPDSNEQSEV
>ggEBVR
SAKVTFSLFYTVLLVFGACGNILALCITFQRRKKKLNSTDLYLVNLALSDALFTLALPGRIAYYVLEFNWPFGDWFCRVTA
FVFYMNTYVSIYFMTCVSVDRYIAVVRIRHPGRTRRVSCARAVCALVWFLVFLQTAPLLLRPMTRRMGDKLTCMEYFNFEE
IPKLPYLLLVACVLGFFLPVGIILVCYVQINLKLCQTAKENPLTVRNGHHHRAFTVILVVLLALLLCFSPYHINIVQFMVR
KILYQPSCRSSRRI
>ggPTFAR
MSGEGKGDAAGSVCPIDSEFRYSLFTVFYSIIFILGFVANCYVLWIFSRIYPTKKLNEIKIFMLNLTVADLLFLVTMPLWI
VVYHNHGNWIMPKFLCSVAGSLFFVNTYASVAFLMVITYNRYQAVTNPIRAAQFTTQRRGIFLSAAIWIITVGSALYYIFD
DNTNVEKIDSINYTRCFERYDSTGNVTPVLAIHIIICTLFYVIFLFILTWNIIIIRTLFSKSGQQRKSAHVKQRALWMVCT
VLVVFIISFVPHHIVDLPWTLTVLEQWKKENCHLRQQLNDAHQVTLCLLSMNCVLDPVIYCFLTKKFQKHVSQNLKSMKGS
RKCSRQTTDTVIEGTIHQEDAIGF
>ggMRGn1
MEPNHATASPAMPTADGENMCAMDVFDTAVDGATLLISLCGLVGNGAVLWLLGCRIRRNPITVYYVLNLAVADFTFLLFMLT
YTLLNLLKELSCFTLPISVKHLVALLLISLFAHNMGMYLLAAISIERCASAFCSGRSHCHRPQHLSAVVCALLWVLSIAVI
TMVTFLCLSHQQEQCRLALITMYILNFLIFAPSMVISNVILLVKVLCRSRQRQHRRLYIVIFLTVLFFLLFGVPLSVWNFM
QHFSSDPGYHQVVFLLACINSSINPFIYVLVGSCRRHCSLASVQVAFQRVFEELGVDGVGSDSSMMHTLSSAP
>ggMRGn4
MMAYGTAWHHGLLRQAGSYKNDYDWTHCEANALSEAPITVLICLCGLVGNAAALWLLRPHIRRNFITIYIFNLAVADFIFL
FSVVIALVIFYGPQSFCHSLGSQDMMTVLSVVITFAFIAGVYLMAALGARTCLPAIPRPLCPCQNSWCLPALLCALLWALA
LLLTLTLYFSPPMLVAFVLSYLLSVLILILSGLALFAKLLCCSWQYLPRKFYIAVLLAIISFPFFTADFAYWLLLRLFDFS
VLAFDTSLLFACVNSSIKPVLYFLAGCCMKKFTFSIRVACHRAFEGVMEPDIVGETLQESTVETPQESTVNV
>ggP2Y9
MGNHSNNHTCLTDDSFKYNLYGAVYSVVFILGLITNCASLFVFCCRMKMRSETAIFMTNLAVSDLLFVFTLPFKIFYNFNR
HWPFGDSLCKISGTAFLTNIYGSMLFLTCISVDRFLAIVYPFRSRTIRTRRNSAIVCAGVWILVLSGGISASLFSTTNVSN
TSTTCFEGFSKRIWKTYLSKITIFIEVVGFIIPLLLNLTCSSLVLRTLRKPATLSQIGTNKEKVLKMIIVHVAIFVVCFVP
YNSILFLYALVRSQAIANCSLERFARTMYPITLCIATMNCCFDPFIYYFTSESFQKSFNIKTQIKMDSLFKTETPLTKTAL
PAQQDEISDQAITNGGDPTSESHF
>ggGPR55
MIFFNFSIPGDMNDTCTNSSINPRVELFQLIMYTPTFILGLLFNVMALSFLFFPAKKLSESTVYLIALIFLDTLLLFTLPF
KIISYHLQNKWKLGSVFCSILESLYFINMYGSILISLCICVDRYIAIQHPFMAPTLRSTKKAALVCVVICLGISAGSVSTS
QFHEESYNISSCFHNFSKSTWENTGLIIGLEMIFFGSMVAMAFCTTQIIRCLRNHRKSDNPQTHMTRAEKVVVTNFVTFLV
CFTPYHVTFFLYFLVRNSIMHIPCHTVLRDILQVTLCWANLNCCLDAVCYYFVLKESAEGSLQNKARNHTEESFDPHTER
>ggCYSLTRn1
MANVSWTEGVSNGTAVRLEADFQYSLFTIIYSIVFVLGLTENVSALYLLSNKARHTSPSYVYMINLALADTLFVCGLPFKI
HYHLNRNNWIFGDVACRITGTLYYINIYLSIVFFTCICVDRYIAVLHPFTYIQIRVTHYVVVATILWVVALSTTVALVLGG
PLHNKGLRNTTMCFENFARSSWISRMALYNILALVFGFVIPFSIILICYPLIARRISRIKHSIRKRKALTTIYIILLICTF
CFLPYHLTHFLHFLMRSQVIQNKSFTNLIYKMRRVTLALVSFNCCLNPFLYYFTSSNRTWHFNFRFRFRSKMVYTICDQKL
GEYSYIYKLHQSCENKKQRDRIN
>ggP2Y6
MSMANFTGGRNSCTFHEEFKQVLLPLVYSVVFLLGLPLNAVVIGQIWLARKALTRTTIYMLNLAMADLLYVCSLPLLIYNY
TQKDYWPFGDFTCKFVRFQFYTNLHGSILFLTCISVQRYMGICHPLASWHKKKGKKLTWLVCAAVWFIVIAQCLPTFVFAS
TGTQRNRTVCYDLSPPDRSTSYFPYGITLTITGFLLPFAAILACYCSMARILCQKDELIGLAVHKKKDKAVRMIIIVVIVF
SISFFPFHLTKTIYLIVRSSASLPCPTLQAFAIAYKCTRPFASMNSVLDPILFYFTQRKFRESTRYLLDKMSSKWRQDHCI
SYGS
>ggP2Y13
MGDFANESTSSNSSGAPTSAPCQRDTAVTHVLFPVLYTLVFLLGLTLNSLAIWAFFHIPSTSTFVVYLKNTLVSDFIMTLM
LPLKILTDSGLGPWQLKAFVCRFSAVIFYDTMYISIVLLGLIAFDRFLKIMRPFGKFWVQQLSSAKILVGLVWLFFLALSV
PNMILSNKKATPQSVKKCALLKSYLGLKWHEAVNYICQFVFWTVLILMLLFYVIIAKKVYESYLKTQKKESKKKPRVKGKV
FIIFTVFFFCFAPFHFSRVPYTLSQTTTRMDCRLQNQLFVAKESTLWLAATNTCMDPLIYIFLCKPFLEKILCGKVSTLQR
TAHVNPRTELDTGTLAIES
>ggFKSG79
MENSSSCSTSTDLKPYYAITYAFILIPGLIGNVLALWVFYGYMKETKRAVIFMINLAIADLSQVLSLPLRIFYYLTGTWEF
GGGLCMLCFYLKYVNMYASIFFLVCISVRRFLFLMYPFRFSDCKRVCDVYISIVGWVVVCVGCLPFPLLRLTQNATNACFA
DLPVQEVDLPTSVIMMTIGELVGFVTPLLIILYCSWKTVLSLKERNSASPDLGEKKKALKMILTCALVFLICFAPYHISFP
LDFFVKTKKIKNCCIQEVISVFHAVALCLASLNSCVDPILYYFTTDEFRRRLSRQELQDSAPLHSHGQQHMQDTLQDNVPL
SAPGQQHTQGTLQDKPTDC
>ggOGR1
MLNFTENATEKCIIDHNIHQTLSPVVYIVVFIIGFPANCLSLYYGYLQIKAKNELGIYLCNLTIADLFYIFSLPFWIQYVL

QHDNWTYDELLCKICGILLYENIYISVGFLCCISIDRYLAVVHPFRFHQFRTMKAAVIVSAIIWTKEIVTCSFVFVHGEIS
MDAESHVVCFEHYPIKEWEHSINYYRFSAGFLFPFFLLAFSYCGILRVVHRSHGTQKKKKLQIKRLVSSTVFIFLVCFGPY
HILLVVRSVFESNCSFAGKIFNIYHTSLLLTTFNCVADPVLYCFSSESTYLNFVKMRDSCLRHLGCLRTETKESYQLNAPE
TPSRAQHEHQPRLLQESQGETGAKDSSSTRADHLQHPAKGALV
>ggGPR87
MNTLTLLSPHRGTPERSCQRRPNRSVGPPGNTTQDEFTTIVLPVLYLVIFLASILLNGLAVWIFFHIRNKTSFIFYLKNIV
VADLLMTLTFPFKIIQDSQLGPWHFNSFLCRYTTVLFYANMYTTIVFLGLISIDRYLKVVKPFGDSRMYSITFTKVLSACV
WVVMAFLALPNLILTNGYPTRRNIDDCIKLKSPLGVKWHTAVIYINTCTFMVVLVVLIGCYIAISRYIYKSSKQFISSSSR
KRKHNQSIRVVVAVFFTCFLPYHLCRIPFTFSHLDKLLDDSAHKILYYCKEMTLFLSACNVCLDPIIYFFMCRSFSRRLFR
KSNMRTRSESIRSLQSVRRSEVRIYHEYTDV
>ggSUCNR1
MMKRADFCFFILPLQAPNGTDGCQEVGAALEKAYLSTMYSLEFALGAIGNSVVVLGYIFCLKEWKSGNIYLFNLSLSDLLF
LCTLPVLVSSYSRGQWIGSSILCHSNRFLLHVNLYTSILFLTVISIDRYMLMRYPFRDHALQKRKTAFIVCIAVWVLVILE
LLPLTFFLETVSPANNFTCLDYASSGDPEKSLIYSLLLTLFGFLIPLAVMCFFYVKMLLFLKTWKDQSSSVLALERPLTLV
VLAVTTFSLLFTPYHVMRNVRLASRIPALNMSVCTQSAINTAYIVTRPIAFLNSAINPVFYFLMGDHFREMLMAKVVQVLG
RLKSTGK
>ggGPR65
MNNTAKCHDDHALDKYLFPFVYSTVMVISIPINCISLYVSCIQVRKKNELAVYLFSLSMADLLYSVILPLWIDYAWNGDNW
TLSARLCQFSAFLMYMNFYTSTALLACISLDRYLALVHPLKFQHLRTRRISLIVSIIVWLLEGTVNSVILMNKDVFHDPCN
STDHTLCYDKYPLELWQARLNLFRICSGYLLPLIIILFCYHKIYQAVRCNQATVDEEKKKIRKLILNITITFIVCFTPYHV
VLLIRSIKEPQTSDLQLLQSMYKIYRITQAFTSLNCIADPILYCFVSESAQTDILNLLRCCLCLQKPEGNQPEEYALCNSA
TKSNALVTYRSSCETQTQ
>ggGPR34
MAATSADLLTTLPYEKAFQGNQNNLALNASETQRNENCFLEDNALSFALISFYSVIFVIGLVGNIIALFAFLCIHQKRNSI
QVYLLNVAIADLLLIFCLPFRILYHISNNTWMFGWILCKIVGTLFYMNMYISIILLGLISLDRYVKINKSVKRPKMLTTTR
SVHICCIVWAVALTGFSLVVVPSLFRSDISNSTLCFHYRNKKNAMTEAILNYIIVIMFWTVFLLLILSYVKIAKNLLKISR
KRANFPNAVKYTQTARNSFIVLIIFTVCFVPYHMFRFVYITSQLQNPSCYWKGIIHTCNEVMLIFSSFNSCLDPVMYFLMS
SSVRKTVLQLICRRIHGDSSVTLESTSEIKLGQYMQERLSTTTPHSSSVKEKSDLIK
>ggGPR34n1
MQLFLHISFQVRTLTMDPLSTPPPSIEVSEQFLGNSCCQIHDGFLSVTLPVMYSLIFAVGLLSNALALWVFSRSVQRRTSI
TVYMRNLALSDLLLALCLPFRIAFQDRSEPRVFCSIVGAFFYLNMYVSITFLSLISLDRYLKIIRPLQKYRIHTVSCSTLA
SGMVWAVNFAFMLPFFFERRGKGPCDHKCFHFRSKSTTAAAFNMAAVAAFFIVLLLFLYFYSKIFAKLHQVSSVKAQQLNK
RTSTRAITKTFVVLVIFIVCFTPYHAVRIPYILAQVGAISSLPWKQGLHLANELVLCISALNSCLDPIIFFFLSSSFRRAV
LCTIQGRLKGALLSNQGGLTPSRSVTEL
>ggGPR43n1h
MLVFAVHALTFTVGFPANVFTFLTLLIKIRCHRPHPHLTAADLLLLNLITADLLVLLFLPFKMAEEAALMVWPFPTALCPI
ANFCFFSSIYLSTLFMAALSVERYFGVVFPHRYNQHQRLWRTMATSAVLSVMALSHCSIIFVAEYHREFSVNGSEADGEGG
YGVNGTKTRESGMENPYDVGLTGLKMGSSHSFEANVINLGKFELIQPDCVDTNVPNTSTANITISSVSKNYSADICRISSP
NANLSKSSFKSSACTTPNTPHIPTTTPWTSQNASETQRHRGYHCYDDFSQTQLQFVLLLRLELFLILFLIPFAITMFCYIG
LIRALLTRPHIPLQKKYRTVGLAVVTMVNFGVCFGPFNLSHVVGFVQQCNPEWRPYALLLTTLSAALDPFIFYFSSSAVPE
GGSVGMGGPISGHNGWVFGVGV
>ggGPR43n1b
MLALTIYALTFAVGLPANVFTFLTLLIKIRCHRPHPHLTAADLLLLNLTTADLLLLLFLPFKMAEAAAKMLWPFPTALCPI
ANFCFYSSIYLSTLFMAALSVERYFGVVFPHRYNRRRRLWQTMAANAVLSVMALSHCSIVFVAEYHREFGVNGSEADGKGG
LGVNGIKTRESGMENPGHVDPTGLKMGISHCFGVTGFDRGGFGLTKSDGFHTNVPNTSTADITIPSGSKIPGADIYRISSP
NAFLNISSLKSSACTTPNTPHIPTTTPWTFQNASETQRHRGYRCYDDFSQAQLRFVLPLRLELFLVLFLIPFAITMFCYIR
LIRALLTRPHIPLQKKYRTVGLAVVTMVNFGVCFGPYNISHVVGFVQQRSPEWRPYALLLTTLSAALDPFIFYFSSSAVRR
AVS
>ggGPR43n1f
MLVFAVHALTFALGFPANVFTFLTLLIKIRCHRPHPHLTAADLLLLNLITADLLVLLFLPFKMAEEAAMMVWPFPTALCPI
ANFCFYSSIYLSTLFMAALSVERYFGVVFPHRYNRRRRLWRTMAASAVLSVMALSHCSIIFVAEYHRDFGVNGSEADGEGG
YGVNGTKTRESGMENPYDVGLTGLKMGSSHSFEANVINHGKFELIQPDCVDTNVPNTSTANITISSVSKNSSDDICRISSP
NANLNNSSFKSSACTTPNTPHIPTTTLWTSQNASETQPSLGYHCYDDFSQAQLRFVLPLRLELFLVLFLIPFAITMFCYIR
LIRALLTRPHIPLQKKYRTVGLAVVTMVNFGVCFGPFNLSHVVGFVQQRSPEWRPYALLLTTLSTALDPFIFYFSSSAVRR
AVSGVVGAIQGTMCGFWGWCWHRE
>ggGPR43n1e
MLVFAVHALTFALGFPANIFTFLTLLIKIRCHRPHPHLTAADLLLLNLITADLLVLLFLPFKMAKEAAMMVWPFPTVLCPI
ANFCFFSSIYLSTLFMAALSVERYFGVVFPHRYNRRRRLWRTMAASAILWVMALSHCSIIFVAEYHREFGINGSEADGEGG
YGVNGTKTRESGMENPYDVGLTGLKMGSSHSFEANVINLGKFELIQPDCVDTNVPNTSTANITIPCVSKNSSADICRISSP
NANLNKSSFKSSACTTPNTPHIPTTTLWKSQNASETQPSLGYHCYDDFSQTQLQFVLLLRLELFLILFLIPFAITMFCYIR
LIRAFLTRPHIPLQKKYRTVGLAVVTMVNFGVCFGPYNISHVVGFVQQRSPEWRPYALLLTTLSAALDPFIFYFSSSAVRR
AVSGMVGAIRGTMCGFWGWCGQRQ
>ggGPR43n1g
MLVFAVHALTFTVGFPAKVFTFLTLLIKIRCHRPHPHLTAADLLLLNLITADLLVLLFLPFKMAEEAALMVWPFPTALCPI
ANFCFFSSIYLSTLFMAALSVERYFGVGVPHRYNRRRRLLRTMAASAILWVMALSHCSIIFVAEYHRDFGVNGSEADGEGG
YGVNGTKTRESGMENPYDVGLTGLKMSSSHSFEANVINLGKFELIQPDCVDTNVPNTSTANITIPCVSKNSSADICRISSP
NANLNKSSFKPSACTTPNTPHIPTTTPWTSQNASETQRHRGYHCYDDFSQTQLQFVLLLRLELFLILFLIPFAITMFCYIR
LIRAFLTRPHIPLQKKYRTVGLAVVTMVNFGVCFGPYNLSHVVGFVQQRSPEWRPYALLLTTLSAALDPFIFYFSSSAVRR

AGSGMVGAIRGTMCGFWG
>ggGPR43n1d
MLVFAVHPLTFAVGFPANVFTFLTLLIKIWCHRPHPHLTAADLLVLLFLPFKMAEEAAMMVWPFPTALCPIANFCFFSSIY
LSTLFMAALSVERYFGVVFPHRYNRRRRLLRTMAASAILWVMALSHCSIIFVAEYHREFGINGSVADGEGGLGVNVTKTRE
SAIRRISSPSTNCNVSSLKSSACTTPNTPHIPTTTPWTSQNAPEIQPGLGYHCYDDFSQAQLHFVLPLRLSLFLILFLIPS
AITMFCYIGLIRALLTRPQIPLQKKYRTVGLAVVTMVNFGVCFGPYNISHVVGFVQQRSPEWRPYALLLTTLSAALDPFIF
YFSSSAVRRAVSGMVGAIRGTMCGFWGWCWQRQ
>ggGPR43n1c
MLIFAVHALTFAVGFPANVFTFLTLLIKIWCHRPHPHLTAADLLLLNLITADLLVLLFLPFKMAEEAAMMVWPFPTALCPI
ANFCFFSSTLFMAALSVERYFGVVFPHRYNRRRRLWRTMAASAVLSVMALSHCSIVFVAEYHREFGVNGSEADGEGGYGVN
LTKTIESGIRRISSPNTNCNISSHKSSACTTPNTPHIPTTTLWTSQNAPETQRHWGYHCYDDFSQTQLHFVLPLLLELFLI
LFLIPFAITMFCYIGLIRALLTRPHIPLQKKYRTVGLAVVTMVNSGVCFGPYNISHVVGFVQQRSPEWSPLLLTTLSAALD
PFIFYFSSSAVRRAVSGVAGAIQGTICRFWGWCWHRE
>ggGPR43n1a
SSSAPQRWQTSSSSTSTTADILFLLFLPFKMAEAAAKMLWPFPTALCPIANFCFYSSIYLSTLFMAALSVERYFGVVFPHR
YNRRRRLWQTMAANAVLSVMALSHCSIVFVAEYHREFGVNGSEADGKGGLGVNGIKTRESGMENPGHVDPTGLKMGISHCF
GVTGFDHGGFGLTKPDGADTNVPNTSTADITIPSGSKIPGADNYRISSPNAFLNISSRKSSACTTPNTPHIPTTTPWTFQN
ASETQRHRGYRCYDDFSQAQLRFVLPLRLELFLVLFLIPFAITMFCYIRLIRALLTRPHIPPQKKYRTVGLAVVTMVNFGI
CFGPYNISHVVGFVQQRSPEWRPYALLLTTLSAALDPFIFYFSSSTVRRAVSGMVGAIRDTVCGFWG
>ggGPR17
MKGPADPSSLLFNCSNQSNFPFETSEQCGKETHLENVLFATFYFLDFILAFIGNSLALWLFVRDQKSGTPANIFLMHLAVA
DLSFVLVLPTRLVYHFSGNHWPFGEIPCRLTGFLFYLNMYASIYFLMCISVDRFLAIVHPVKSIKLRRPLYAHLACAFLWL
IVGVAMAPLLLSVQTVEVNNTTICLQLYREKASRHALVSLAVAFTFPFVTTVTCYLLIIRSLKSGNRVEKHLKEKAIKMII
MVLMIFLVCFVPYHVNRYIYILHYNGTKASCETQRLLALNNRITSCLTSLNGAFDPVMYFFVAEKFREALCNLFCGRRPVM
TLQTCEGKTNESSLSAKSEL
>ggGPR18
MMPENHYPEEYRISSLVFYSFVFIVGLVVNATALWVFSCSTKKRTTITIYMMNVALLDITFILSLPFRIIYHGKEMWPFGD
IFCRIIGAFTVFYPAIALWLLAFISVDRFMAIVQPKHVKELKNTKKALLACTGIWIMTLATTSPLLFLRSDPDKAFNFTTC
MKMLDIIHLKEVNMLNFSRLIFFFLIPLLIMMGCYLVIIYNFIHGRTSQLKPKAKERSIRIIVTLIAQVLVCFVPFHICXX
XXXXXXXXXXXXXXXXXXXXXXXXXXXQVITLPDSRILATLKKHSMPRSNRCLLHCNYERRTMIMAPLHVCSTVFVNLK
>ggGPR20
MPTSSTQVPALGHINSTQQPNSSIYTFSKFIHPDKELYKQFYSLWIALIVVNAIIFVVGVVLNSLALYVFCFRTKTKTTSV
IYTINLIVTDLLVGFSLPVRIIMFYTAEGCVHCSFVHIFGYFVNMYCSILFLTCICVDRYLAIVQVQASRKWRNPTCAKGI
CVFIWIFATVVTFSILTMAIRFAQCCLSEILVLMVCEYFVPLIIIIFFTTRIMCALSKPSLMHQSRERRMKAVQLLITVLI
IFMICFTPFHVRHLAISIKPDMPHNVSLLVYHVTVTLSSLNSCMDPIVYCFVTNNFQSTMKNIFRKTEPEQTTVDILGVNK
NTKGSNAIITFSNTIGSPVSLPSPSSVQI
>ggEBI2
MDTFTTSQTNSSTCDLYEHKDTARIILSVFYSLILILGVLGNTIALTVIFKNRKKINSTTLYSTNLVFSDLLFCIALPTRI
AYYALGFHWPFGEALCRITALLFYINTYAGVNFMTCLSIDRFFAVVHPFRYKIRRIKYAKGICVFIWFLVFSQTFPLLIQP
MTHEENERTTCMEYPNFEKIAHLPFILLAACLVGYLIPLGIILFCYSQISCKLFQTAKENPLTEKSGINKKAINTILFVII
VFIICFTPYHVAIIQHMIKKLQYQPLCNENQIFQKSLHYTVFLMNFNCCLDPFIYFFACKGYKRTVLKILRRQVSVSISSA
ARSHHEESSRDVGETQMMVLAKSANGKLPEKSSL
>ggCYSLT1
MTTFLNNSLCQHAIDDFRNRVYSTLYSMISIMGFAGNGVVLFVLIRTFRQRTAFQVYMFNLAVSDFLCVCTLPLRVVYYVH
QGHWFFSDILCRISSYALYVNLYCSIFFMTAMSFFRCIAIVFPVQNINLVTERKAKFVCIGIWIFVILTSTPFLMNGTYQH
GNKTKCFEPPEDIQKTNIIVNLDFIALFLGFIIPFIIITICYTMIIRTLLKNSLKKNQANRKKAIWMIVIVTATFLVSFTP
YHILRTVHLHVLRLRHASCEDTVYLQKSVVVTLPLAAANCCFDPLLYFFSGGNFRKRLTTFRKASSSSITQAFRKKFSLKE
RDDEPFGESHKENGNAPVVAL
>ggCYSLT2
MNSSEMMLNDSSTNSSFNCSTDAFKQVIYPIVYLLIFFLGSVGNSLSIYVFLQPSQRKTSVNTYMQNLAVSDLMFVSTLPF
RATYYLLGSQWVFGDILCRIMTYTMYVNMYCSIYFLTVLSVVRFTAIVYPFKSWKVSNMKYAKIMCAVIWVFVLAASSPLL
SKAIAGYENPAKCLDLHPSSTHRLLMMNSFVLVVGFILPFCTIIVCYIFAIRALLKSKTPQRKKAVCHKKALSTIIITLIL
FLLCFLPYHILRTVYLVFGGCSPDNLFVHKAQVVSLCLAATNSCLDPVLYYFAAENFKARIRSLYRRTGMLCHRGGQLIVP
IIPLCPEHSCRGRRLQNLLSGPWPKQPMELHNLSSPSPSLSSSVLPPSFSPSPSSAPSAFTTVGGSSGGPCHPTSSSLVSA
FLAPILALEFVLGLVGNSLALFIFCIHTRPWTSNTVFLVSLVAADFLLISNLPLRVDYYLLHETWRFGAAACKVNLFMLST
NRTASVVFLTAIALNRYLKVVQPHHVLSRASVGAAARVAGGLWVGILLLNGHLLLSTFSGPSCLSYRVGTKPSASLRWHQA
LYLLEFFLPLALILFAIVSIGLTIRNRGLGGQAGPQRAMRVLAMVVAVYTICFLPSIIFGMASMVAFWLSACRSLDLCTQL
FHGSLAFTYLNSVLDPVLYCFSSPNFLHQSRALLGLTRGRQGPVSDESSYQPSRQWRYREASRKAEAIGKLKVQGEVSLEK
EGSSQG
>ggMAS
MDESNITFHPSEGTENISMHRNISTQERVWEILTPLWVIMIISFLGFCENGIVLWCLCFQIKRNPFTAYITHLSIADISLL
FCIFILSIEYIAGFGFAYGFYYYITTTLSIVFLLGYNTGLYLLTAISIERCLSIVYPIWYRCHRSQHQSAIVCAILWTLSF
LMTIAEYLTCKDDSTKEQFDDANHCQAMLIFIWILTFLIFIPLMILSSLILVIRIHRNSLRPHSSKLYIIIVATVVVFLIF
AMPMRLLYLLNYHHWSSLLSQQNHVTIVLSTVNSSINPLVYFFVGSSKNKRFKESLKVVLSRALADGLRPRSQEVGMSLDI
AETIF
>ggMRGn3
MSASTTPFFLLDANSQAQGANHSSAAGRPEPSYAVLKFMESFCLISAACGMVGNGIVLWYLGFCIRRNHFTVYILNLAAAD
FGYLLCIAIETVQYLMQFNVGVQFGIFLFLDLFTYGTGLYLLTAISIERCLSVLCPIWCRSHRPKHLSAIISGLLWCLSLL

LNTVGYILCSIHPSGSCRMLLITIGVLDFLVCTPLMLFFSLTLFLRVKCSSQKFRTGRLFTVIMLTVLFFLIFAVPLSVLI
LFDFLGYKFLYSPEIGFVLSCVNSSLNPVIYFLVGSYRDRRIKFTLRLAFQRVFEDSADDKEERDTRDTITMSS
>ggMRGn5
MADTTSAVFVLNFSEYMDSFENTTSECPTLNYKEKVTLSVCVGISFCGLVDNAIVMRFLFFHVKKNPFTVYTLNLAVADFS
LLLLLSLLMLLILTLPICSLFDGILLSYVKIYIAVGYLCHFFDLSSLGFLTAISVERCLSVLFPIWYRCRRPKRLAGIVSG
LLWAFSGSLVSFMYLTFNFIDEHMKTFGAVAFATCLTFSSMMLVSNLSLFIKIQRGFRIRHPGKLYIAVLINVIFFFVFAI
PFSVEVFVNLADRLELFPEDTSLLLVMLNSSINPVIYFLVGCCRQRRSRCSVTAALRRVFEEKATSSAPEDKVDETTV
>ggMRGn2
YVLNLAVADFTFLLFMLTYTLLNLLKELSCLTLPISVKHLVALLLISLFAHNMGMYLLAAISIERCVSILCPLWYRCRRPQ
HLSAVVCALLWVLSIAVITMVTFLCLSHQHEQCRLALITMYVLNSLIFAPSMVISNVILLVKVLCRSRQRQHRRLYIVIFL
TVLFFLLFGVPLSVWNFMQHFISDPGYHQVVFLLACINSSINPFIYLLVGGGRRHCSLASLQVAFQRVFEETGDTVISA
>ggLGR5
MATSRADRALLLLLLLLLCLPGLRRGALGGGQRPRGCLAPCRCEQEGMLLRADCSDLGLTAVPANLSAFTSYLRLAGNGLTY
IPKGAFAGLFSLKVLMLQNNQLRQVPTEALQNLRSLQSLRLDANHINYVPPNCFNGLVSLRHLWLDDNSLTEIPIQAFRSL
PALQAMTLALNKIHYIPDYAFGNLSSLVVLHLHNNRIYSLGKKCFDGLHSLETLDLNYNSLDEFPTAIRTLTNLKELGFHS
NNIKSIPERAFVGNPSLITIHFYDNPIQLVGKSAFQNLPELRTLTLNGASQITEFPDLTGTTSLESLTLTGAQITSLPRSA
CDQLPNLQVLDLAWNKIKIIHPNAFSSLPSLIKLDVSSNLLSSFPVTGLHGLTHLKLTGNHALQSLISSDNFPELKVMEMP
YAYQCCAFGACESHYKISSQWNKDENSSTDDFPRKDIGLLQIQDERDFEDFLLDFEEDLKAHHSVQCSPSPGPFKPCDHLF
GSWLIRIGVWTIVGLTFVCNALVTATVFRSPLYMSSIKLLIGLIGIVNALMGLSSGVLASVDASTFGSFAQYGAQWESGIG
CQMTGLLSIFASEASIFLLTLAALERAFSIKHATKFETKSSIASVKIAIFFCFILALVIAVIPLLTGSEYGVSPLCLPLPF
GEPTAMGYMVALVLLNSLCFLVMTIAYTKLYCSLEKGELDNIWDCSMVKHIALLLFTNCILYCPVAFLSFSSLLNLTFISP
EVIKSILLVIVPLPACLNPLLYILFNPHFKEDLGSLRKQTLLWRRSKHASLISVNSEDIEKQSCDSTQALVTFTSASISYD
MPTSNSLMPSSYQMTESCNISSVAFVPCR
>ggLGR7
DLSLNGLDELPPSLFHGLAALQQLNISYNPLKEICPSQFESLPQLLSLSLEGLEIPNIHNVTFRGLANLSHIYFKKFQYCS
SAPHVRSCKPNTDGISSFEHLLANIILRVFVWVIACITCLGNLCVICMRSCVVTESSAHTMAIKSLCCADGLMGIYLFVIG
AFDLKYSGEYNRHAQSWMASVPCQLVGSLAMLSSEVSVLLLTYMTLEKYLSIVFPFSYRRAGRKQTASVLAAIWLLGLSLS
IVPLCCKESFGNYYGRNGVCFPLQSELGEQPSARGFSTTIYLGLNLAAFITIVFAYTGMFYSIRITTCRTAESNVCPRDVA
VAKRFFFIVFTDALCWIPIFLLKLLSLLQVEIPGTVTSWVVIFILPINSALNPILYTITTAPFQEKVKGCLHTRRPELSSR
QSFMLVVL
>ggLGRn1
PVSRGGAACPLGYFPCGNITKCLPQQLHCNGEDDCGNHADEDNCGMXXYLSNNKITNLKPRVFEDLHKLEWLYFKKFQYCG
YAPHVRSCKPNTDGISSLENLLASIIQRVFVWVVSAITCFGNIFVICMRPYIRSENKLHAISIMSLCCADCLMGIYLFVIG
AFDLKYRGEYNKHAQLWMDSIHCQLVGSLAILSTEVSVLLLTYLTLEKYICIVYPFRCLKPRKCRTISILVLIWVIGFAVA
FIPLSNKEFFRNYYGTNGVCFPLHSEQSESSGSQIYSVVIFLGVNLAAFLIIVFSYGSMFYSVHQTAIMATEIQNHIKKEM
ILAKRFFFIVFTDALCWIPIFILKLLSLLQVEIP
>ggP2Y8
MVKNGSHLDAETLAMLQNKAISITLPVVYTMVAIISIPGNFFSLWVLCWHIKPKTPSVIFMINLSITDLLLACCFPFQIFY
HIQRNHWIFGKTLCSLVTVMFYSNMYSSILTMTCISIERYMGVVYPMKLIKWRRKRYALGACVIMWIFLLLAFYPLESTDL
TYEVKELGIITCFDVLKWEMLPNFAAWVAFLLTLFVVLFLIPFIVTVGCYIGTIRKLIQTSSRYGNKQKTRSIYLAIIVLS
VFITCFAPNNFILLAHMIVRLFYEGSLYPAYKLTLCLSCLNNCIDPFIYYFASKEFYQKFMQVFRPKVPLSDSLETRRESL
FSGRTMSVRSMSSGPMDGLEGVKICLQRQESVF
>ggP2Y1
MTEALISAALNGTQPELLAGGWAAGNATTKCSLTKTGFQFYYLPTVYILVFITGFLGNSVAIWMFVFHMRPWSGISVYMFN
LALADFLYVLTLPALIFYYFNKTDWIFGDVMCKLQRFIFHVNLYGSILFLTCISVHRYTGVVHPLKSLGRLKKKNAVYVSS
LVWALVVAVIAPILFYSGTGVRRNKTITCYDTTADEYLRSYFVYSMCTTVFMFCIPFIVILGCYGLIVKALIYKDLDNSPL
RRKSIYLVIIVLTVFAVSYLPFHVMKTLNLRARLDFQTPQMCAFNDKVYATYQVTRGLASLNSCVDPILYFLAGDTFRRRL
SRATRKSSRRSEPNVQSKSEEMTLNILTEYKQNGDTSL
>ggP2Y4
MDAPVRMFSLAPWTPTPTPWLGGNTTATAEAKCVFNEEFKFILLPISYGIVFVVGLPLNSWAMWIFVSRMRPWNATTTYMF
NLAISDTLYVFSLPTLVYYYADRNNWPFGEVLCKIVRFLFYANLYSSILFLTCISVHRYMGICHPIRSLKWVKTKHARLIC
VGVWLVVTICLIPNLIFVTTSSKDNSTLCHDTTKPEEFDHYVHYSSSVMALLFGIPFLVIVVCYCLMAKRLCKPSFPSPSP
RVPSYKKRSIKMIIIVLTVFAICFVPFHITRTLYYTSRYFQADCQTLNIINFTYKITRPLASINSCLDPILYFMAGDKYRG
RLRRGAAQRPRPVPTSLLSLVAPSVDSSVVGSCCNSESRGTGTVWSRGGQ
>ggP2Y10a
MTRTPPPVPNAPSSPIMMHSNWTCSDPNVTYKSTLYAATYTIIFIPGLLANSAALWVLCRFISKKNKAIIFMINLAVADLA
HVLSLPLRIYYYINRTWPFGRFLCLLCFYLKYLNMYASICFLTCISIQRYFFLYQPFKAKNWKRRYDIAISAAVWLFVGAA
CSPFPIMRGFRLQNNSKTCFADLEVRQIESKVATVAMTATAELFGFIGPLIVIVFCTWKTKISLRGFRIPLQNNSEGRKAS
RMVSTCAIVFFVCFAPYHINFFFYMMVKENVITDCLLRRITLHTQPFCLTLASLDCCLDPILYFFMTSEFQDQISRHSSMA
IRSRLMSKESASSVKE
>ggP2Y10b
MESNMSSGNCSQPQMSFQSTLYAATYTIIFIPGLLANSAALWVLCRFISKKSKAVIFMINLAVADLAHVLSLPLRMYYYIN
HTWPFGTFLWQLCFYLKYLNIXXXXXXXXXXXIQRYLFLLLPFKATAWKRRYDVGLSAAVWLFVGAACLPLPIVRSPALSN
STTTCFSDLAVKQLTPGASIALVTVAELFGFVIPFGIIAWCTWKMRQSLQEGGTQLQSTSEKQKALRMVLMCAAVFFICFT
PYHINFPFFMMVIENIIKDCTVHSNTLRFHPISLCLASLNCCLDPVLYYFMTSEFQDQLLRHGCAALWARLARSRRSLTMS
NVGHNIRTKRRNLPRFRFWSLPKFFGRINSMEIPPMQPDELLLESIS
>ggP2Y12
MQTKATSNFSYSINGSNCTSDNRIRQVIFPLLYTLLFLVGLTMNGLAMWVFFKISSKSNFIIFLKNTVISDILMILTFPFK

ILSDAQLVPWVLRGFVCQVSQVVFYFTMYISILFLGLITIDRYQKATSPFRTSTTRSLLGAKILSTAIWILMFILSLPNMI
LTNKKKTPTNVKKCALLKSEFGLVWHEAVNYICQLIFWVNLIVIVVCYILISKELYKSYQRTRCTGRPSRKSVNLKVFIII
AVFFICFVPFHFTRIPYTLSQTRNVFDCSAVNTLFYLKESTLWLTSLNACLDPFIYFFLCKSFRKSLLGMLCKHTASPELQ
AQIAVQNERDDTEDTPV
>ggP2Y15a
MNATSGDLADPTTWQDIGGHFPNCTDVEVSLKTLYLPVMYSLIFLVGFPGNVIAICVYSFKMRPWKSSTIIMLNLALTDLL
YLTSLPFLIHYYASGEHWVFGGFLCKFIRFGFHFNLYSSILFLTCFSAFRYMVIVHPMKFFHVQRRRWTAVACAAIWVISL
AAVSPINFLISSKEEQNRSLCLDFTSSENLGAIRWYNWLLTSLAFFLPLVTVTLCYTLIICALATGPQTRASYKQKARRLA
IVLLVVFYVCFLPFHLFRVARIELQLCPISCHVEKQIHTIYIISRPLAALNTCGNLLLYVVMGDNFQQAILSLSWCKWSKC
IQPPVSSNYVNKSEITLRI
>ggF2RL3
MGTSRSGQLAPRLALCCAVWVLCWAAPDYDDYSQNSTSEQITTAEITPCPRAIPGEKATIDNVTYLLLHEAARSQLSSGVT
VLLIPSLYSLVFLLGLPSNGLALWVLATRTEKLTSTIFLMNLAAADLLLISVLPLKIFYYFLGNNWPFGEGLCRVTTAFFY
GNMYCSVLLLTCISVDRYLAVVHPFFSRSFRTPTFAAWTCAAVWLCAAGITLPLTLQQQSYPLYRADITLCHDVLPRQEDD
GYYFYYFVCLIACAFLAPLAVMLFSYCSVLQALLGSGKRYFHSMKLTALVLVMLTAFYTPSNVLLLVHYSSYQCKLSGSLY
ISYMVSLAVSTCNSCADPFVYYYISEDFREKVRSTFFNRSKTPSLRTSKETLPQKSSKESLV
>ggF2RL1
MAGRRGLCVLLLLSALLAAASSSKGRSFAGQKVPDTSNASEESYKVDDFAAKVLTGKLTTVFLPTVYVIVFIIGLPSNAMA
IWVFFFRTKKKHPAVIYMVNLALADLLFVVWFPLKIAYHLNGNNWLFGEGLCKVLVGFFYGNMYCSILFMTCLSVQRYWVV
VNPIVHSRRKSEIALGISLAIWILILLGTIPLYLVNQTVYISDLNITTCHDVLPENILAHDMFKYFLSLAIGLFLIPAVIT
AVAYILMIKTLNASISDISTGKKRKRAIKLIIAVLSMYLICFTPSNVLLVVHYVLLKNYNQSSLYVSYITALCLSTLNSCI
DPFIYYYISKDFRDNLKNALICRSVRTTRRMQVSLSSGRYPRKSNSYSSNSNGTTKSTY
>ggF2R
MGLPALLCALAALSCSLSPPVAVAASRAGESWGRGSALPRLGVWVGERACSRRRPDHTYNNSSARARTFLIPDRGEPIPLE
DIESSAENDSEVGRGPTNQTGSPQHEQRTVSAETKRYLTSPWLTRFVPSVYTVVVVLSLPLNITAIFVFLKKMKIEKPAVV
YMLNLALADVLFVSVLPFKIAYHFSGNNWVFGPRMCRFVTAAFYCNMYCSIMLMTSISFDRFLAVVYPMQALGWRTLTRAS
VICFIIWFVAISGVVPFLVREQAMEIPKLNITTCHDVLRESELHSYYVYFFSVFSSVFFVVPFIITTICYVCIIRCLSSST
IVARQNKKTRALLLCVAVFSVFVICFGPTNVLLLIHYIHFSYNNSLEYLYFAYVLCVCISSVSCCIDPFIYYYASSQYQRQ
LFSLLSCKETLDPNSSNSSGQSMSSTSRRGTCSTNANNSVYSKLLVIQ
>ggFSHR
MSLGLTCLLILLASCSGCQHHTCLCEGRIFICQEIKVVQLPRDIPTNATELRFVLTKMRVIPKGAFTGLHDLEKIEISQND
ALEIIEGNVFSSLPKLHEIRIEKANKLMKIDQDAFQHLPSLRYLLISNTGLSFLPVVHKVHSFQKVLLDVQDNIHIRTIER
NTFMGLSSESVILRLNKNGIQEIKDHAFNGTCLDELNLSDNYNLEKLPEKVFQGAIGPVVLDISRTRISFLPSHGLEFIKK
LRARSTYKLKKLPDVNKFRSLIEANFTYPSHCCAFTNRKTQNTEFYPICSMSPAKQDLGEQTGKRKHRRSAAEDYISHYGT
RFGPVENEFDYGLCNEVVDFVCSPKPDAFNPCEDIMGYNVLRVLIWFINILAITGNTTVLIILISSQYKLTVPRFLMCNLA
FADLCIGIYLLFIASVDIQTKSRYYNYAIDWQTGAGCNAAGFFTVFASELSVYTLTVITLERWHTITYAMQLNRKVRLRHA
VIIMVFGWMFAFTVALLPIFGISSYMKVSICLPMHIETPFSQAYVIFLLVLNVLAFVIICICYICIYFTVRNPNVISSNSD
TKIAKRMAILIFTDFLCMAPISFFAISASLRVPLITVSKSKILLVLFYPINSCANPFLYAIFTKTFRRDFFILLSKFGCCE
MQAQIYRTETSSSAHNFHTRNGHYPTASKNSDGTIYSLVPLNHLN
>ggLHCGR
MLPALLPLLLPALLPGAGGGRCPQRCACTQPALRCPTPPPGARPAPARASFTHLPVKVIPSHAFEGLRDAFIIEISQSDSL
ERIEASAFDSLPALSEILILNTKNLLHIEDGAFRNLPRLKYLSICNTGIIEFPDLTQIFSSEAHFILELCDNLRMTTIPQN
AFQGMSNESLTLKLYKNGFEDIHSHAFNGTKLNQLILKDNKNLRRIHNDALRGATGPDVLDISSTALESLPSYGLEAIQVL
NAMSSYSLKRLPPLDKFSSLLEAVLTYPSHCCAFQNLRTEKQNSLLSIFDNFSKQCESTMRKPASEVFYRDASSNTSLWPA
EKHMYPLETGEEAFPYSYSTVFYEDEMTGFDFEYDFCQPKILTCTPEPDAFNPCEDILGYSFLRVLIWFINILALAGNFIV
LLVLITSHYKLTVPRFLMCNLSFADFCMGLYLLLIASVDAQTSGQYYNHAIDWQTGSGCSTAGFFTVFASELSVYTLTVIT
IERWHTITYAMQLDRKLRLRHAVPIMLGGWVFSILIAVLPLLGVSSYMKVSICLPMDIETGLSQAYILLILMLNVIAFLVI
CACYIKIYVAVQNPELVAANKDTKIAKRMAILIFTDFTCMAPISFFAISAAIKVPLITVTNSKILLVLFYPVNSCANPFLY
AIFTKAFQRDFFLLMSKLGCCKSRAELYRVNYFSAYTPNCKNGSSAPGPSKASQALLLLSASEKLCKTRRSTKKSQPECQ
>ggGPR82
MRNSTCVLHPSIATTVALPVIYSLLFITGSFGNILTAWVFSRQASTKRTQYIYLVNLVTANLLVCSTMPFLAGYFAKGHEW
TYSSDVCRAAYHLGTTVMHVCMYVTITILCTIALSQYATLKKNNNARHSPAVTKSLYKRGLQKFRQPKFAKYFCIFIWLIV
LCATITAIAFDAQARREQIFHTCYNTKVEIGEFTAKTAASFATACFFLFFLTVLLSYYSLTRHLSKIQKNTHFGQNHMIYR
TVKRNILVIQIILTVCFLPYHIFKPIFYVLLPEEDCSRINYLVEIKNFLTCLCVSRSSIDPVMTLLIDKTFKKSLYSLFKK
SIPDHHQRRADIFTEEIPPM
>ggG2A
MGDHNVCATIGKDNNSTICSNIPYEDTKILVIVVYSIVFAIGLPANCLTSMLALVQIRRNNAIAIYIFSLSLCDLMYLSTL
PLWIIYAQQNHQWTMGEQVCKLTGFVFFCNIYISILLLCCISLDRYVALVYALESRGRRGRKKAIIIVCFLVAAVAVIHSP
VFHMNSIHNSNCFETLPLDKTLASFSFARFLFGFVIPFTVLIFTNYKIFQRTKTSDSVTSHQKAKVKYLAIAIIVIFLMCF
APYHVVLLIRAIYFMFHQDCSCPFERSIYSVFTAFLCL
>ggH963
MPNNASQCYIKTELEPFTYFYYLIFLTGFLGSCFALWAFTQKGQKQKCMSIYLTNLLTADFLLTLALPVKIIVDLGVAPWK
LQIFHCQVTACLIYLNMYLSIIFLGFVSMDRYLQLMHSSKIYRIQEPGFAKILSAVVWTMVLLITVPNMAIPIKTIEERPN
ARCIDFKTKFGRDWHVFTNFVCTAIFLNFSAVILISNFLVVRQLYQNKYSESYTNVKKALVSILLLTAAYLLCFLPYHVVR
IPYTLSQSNAITSCSLKQTLFHAKESTLLFAVSNLCFDPILYYRLSNVFRLKVTEAFMASKQVKVSTDEEAQHRSEQWVQR
Y
>ggP2Y14
MFNSSTNSSGSSCSNSTVITRAVIPLIYCLIFVVGLLLNGVAAWIFLCISSKKSFIVYLKNIVVADLLMSLTFPFKILADS

EIAPPQLNTFVCRYSAVVFYANMYIGISFFGLIGFDRYYKIVKPLFTSFVHTVHYSKVISAIIWLLITFITLPNMILTNEI
SKANDSRTCIGLKSELGKQWHEVSSYICTGIFWIVFLLLIIFYTSISKKIYSSYKKFRRNSDSAKRKTSRNIFTIMFVFVL
CFVPYHFCRIPYTLSQTSPQFNCRSQRALFLAKEFTLLLSAANVCLDPIIYFFLCQPFRERLYQKLHLQLKTSGEAEITKS
RRSNTLQESVNIL
>ggTSHR
MLWLPVAFQLVLVLCSQGTERCPSAFCECSDWDDYKITCRDIHFIPSLPEDTQTLXRFMETHLRTIPSEAFSNLPNISRIY
ISIDETLQSLEAHSFNSLSKVTHIEIRNLRNLDYIDPDAFKNLPLLKYLGIFNTGLKVFPDLTKIYSSDVNFLLEIADNPF
MTSVPANAFHGLCNESLTLASKAKVLMGQIWMLCNIQGKGFNGTNLDAIYLHKNKYLKVINEDAFLGVHSGPTLLDISRTV
IVNLPVKGLESLKELMARNTWTLKKLPAVKIFLQLIRADLSYPSHCCAFKNWKKNSGILEYLMCNQSGSHSSRNRRSFRAI
RGPFYKDYAEEYTDRTDTVYDKNTKFTNFHENSQYYIFLEEHGEGNVGFGQELKNPQTEDMQAFDSHYDYLVCGGSEDVIC
TPEPDEFNPCEDIMGYQFLRIVVWFVNLLAIIGNVFVLFILLTSHYKLTVPRFLMCNLAFADFCMGLYLLLIASVDLYTRS
EYYNHAIEWQTGPGCNTAGFFTVFASELSVYTLTVITLERWYAITFAMRPNRKIRLRHALVIMLGGWLSCFLLALLPLVGV
SSYSKVSICLPMDTETPVAEAYVVFVLICNIIAFVIICACYIKIYITVRNPQYKSGDKDTKIAKRMAVLIFTDFLCMAPIS
FHALSAIMNKPLITVTNSKILLVLFYPLNSCANPFLYAIFTKAFRRDVFILLSKFGICEHQAQVYRGQTISVKNSSGSYGQ
RISQGIGQILTSIQDPVNDYLPAMTMQNQILAEECKQTEL
>ggP2Y2
MANLSTPAAWTRVTHSSFVADNADNNTYRCVFDEDFKYVLLPVSYGIVCVVGLFLNLLALYGFIFRIKTWNASTTYMFNLA
VSDTLYVVSLPLLVYYYAMGDNWPFGVGLCKIVRFLFYTNLYCSILFLLCISIHRFLGICYPLKSLQWGHVRYARRVSVIV
WVVTVVCQLPVLFFVTTSMRRGTITCHDTSSKDLFGQFVIYSSVMMVLLFCIPFLVIIVCYCLMARRLLQPTRGMSRLSRS
KKKSVKMIIIVLVVFSVCFLPFHVTRSLYYSFRSWDLSCQTLNAINLAYKVTRPLASTNSCLDPVLYFLAGQRFMKLTGNK
IPWKLQNEMAMGTVPNGPLGTSNDTDTTSKDVKS
>ggP2Y15b
MAPEHPDNVTALSDPSTNCKDENFLQVKSYLSAFYGLIFLVCFPGNIVTIFVYFVKMRPWKGSTIIMLNLAVTDLLYVATL
PFFIHYSANGNNWIFGNFMCKFIHFGFYFNMYSGIIFLSCFSIFRFLVVVHPVRCFFIQKKRWAVVTCTVIWMISLATISP
LGTLISIKHSQNRTICPDLSSAEDLGATRWYNWLLTIFAFFLPLVTVTLCYALIIHALATGPHTQACYKQKARRLAIILLV
VFYVCFLPFHVFQVIRIELRVRVASCNLKNTILFIFILTKPLAALNTFGNLLLYVVAGDSFQHAIFSLLKFWTHKNLK
>ggP2Y5
MVSSNCSTEDSFKYTLYGCVFSMVFVLGLIANCVAIYIFTFTLKVRNETTTYMLNLAISDLLFVFTLPFRIYYFVVRNWPF
GDVLCKISVTLFYTNMYGSILFLTCISVDRFLAIVHPFRSKTLRTKRNARIVCVAVWITVLAGSTPASFFQSTNRQNNTEQ
RTCFENFPESTWKTYLSRIVIFIEIVGFFIPLILNVTCSTMVLRTLNKPLTLSRNKLSKKKVLKMIFVHLVIFCFCFVPYN
ITLILYSLMRTQTWINCSVVTAVRTMYPVTLCIAVSNCCFDPIVYYFTSDTIQNSIKNNRFTRTRDNRFSETPLSENFLQH
SFQTIKMKIFDTDSPR
>ggGPR92
MSAANVSQPCKDYSFYHKLLLPGYILIFITGLTLNVMALWIFVRYLRLKSVVMIYMFNLAVSDLTFTLPLPLRLYYYFNHE
WPFGNTLCQVSGSVFQINMYGSCIFLMCINLDRYIAIVHPLRWRHLRRPQVARLLCLVVWVVILMGSIPTAIVHKQNHCES
QNQTTYLCFESFSDNMWQKNLFPLVILAEILGFLLPLSAVTYCSIQIFRELCQTRQPKTLRQQKTVRLLLVNLVIFVIFFV
PYNTTLAVYGLIKARVIDVEKQMQASVREVLIVTMMLASMNCTLDPLIYYFSTEGFRNTFKKLRRGQTWHSDTGVIRNQVV
EQKSTRNHAASTAKQLPSENFHCPGEAVPALPTTVFLNGPIEDSEI
>ggF2RL3n1
HEEAECPDASLESFLNSTTTTILLPTLYSVVLLVGLPANALACWVLAKNFRRCSSSLFLLNLAAADLLFILLLPFKISYHL
LGNHWLFGDYLCRTMVALFYGNMYTSILFLTCIGVERYVSVAHPFLWKGSMGTWGRAGICVAIWLLVGLGVSPLLFYPQTE
HISKLNITTCHDVQGKDNFFQHYFLCLVGLGFGVPFVLMIISHGCILARLLGKQNNYRHVVRVLVVVLLAFLLCFTPSNVL
LLMHYLRQDTGCNNITYSWYALALAFSAFNNCFDPFLYFYVSKDFRGWIRDAGCC
>ggLGR4
MIRLTVWFIFLVALFFNLLVMLTVFASCAPLPSSKLFIGLISVSNLFMGVYTGILTFLDAISWGRFAEFGIWWETGSGCRV
AGFLAVFSSESAIFFLMLAAVERSFSAKEIIKKGKSNHQKQFQIAAVLAFLCAVVAGCLPLFHKAEYSASPLCLPFPTGET
PSLGFTVTLVLLNSLAFLLMAIIYTKLYCNLEKEDLSENSQSCMIKHVAWLIFTNCIFFCPVAFFSFAPLITAISISPEIM
KSVTLIFFPLPACLNPVLYVFFNPKFKEDWKLLRRQVTRKNGVVAISISAQGGCATQDFYYDCGMYTHLQGNVAMCECCES
LLLSKPVPCKHLIKSHSCPTLAVVPCQRPDGYWSDCGTQSAHSDYADEDDSFVSDSSDQVQACGRACFYQSRGFPLVRYAY
NINIPRIKD
>ggP2Y1n1
MAPGRLSAAVWLWVMAQVGPDFAFSKMDPKGTQCHDTTGAESLGAYVPYTAAVTVTGFIIPFLIIVGCYCHVVLVLCRSDT
VEPGLRRSSIRLVVLVTVLFSVCFLPYHIFRNLNLLSRAWQRQGSCTQAMKDIYVSYQVTRGLASFNSALNPLLYVMTSKD
CMSRMRNIYQRSKETLGSIFTWESNNQAHETRSSIVFEEQEASEQL
>ggCRHR1
MVPGPRPALLLLLFLLQAFLLWDSPVAASIQEQYCESLLPTTNHTGPQCNASVDLIGTCWPRSAVGQLVARPCPEYFYGVR
YNTTNNGYRECLANGSWAARVNYSQCQEILSEEKRSKLHYHIAVIINYLGHCVSLGTLLVAFVLFMRLRSIRCLRNIIHWN
LITAFILRNATWFVVQLTMNPEVHESNVVWCRLVTAAYNYFHVTNFFWMFGEGCYLHTAIVLTYSTDKLRKWMFICIGWCI
PFPIIVAWAIGKLYYDNEKCWFGKRAGVYTDYIYQGPMILVLLINFIFLFNIVRILMTKLRASTTSETIQYRKAVKATLVL
LSLLGITYMLFFVNPGEDEISRIVFIYFNSFLESFQGFFVSVFYCFLNSEVRSAVRKRWHRWQDKHSIRARVARAMSIPTS
PTRVSFHSIKQSSAV
>ggCRHR2
YCNATTDQIGTCWPRASAGKLVERPCPEFFNGIKYNTTKNAYRECLGNGTWASKINYSQCEPILDDKRKYAHHYKIALIIN
YLGHCISVGALIVAFMLFLCLRSIRCLRNVIHWNLITTFILRNVMWFLLQMIDHNIHESNEPWCRCITTVYNYFVVTNFFW
MFVEGCYLHTAIVMTYSTDKLRKWVFLFIGWCIPCPIIVAWAIGKLYYENEQCWFGKEPGKYIDYIYQGPVILVLLINFVF
LFNIVRILMTKLRASTTSETIQYRKAVKATLVLLPLLGITYMLFFVNPGEDDISQIVFIYFNSFLQSVLLPGKDGTAGRII
TLSECVWHVPCPSPRPQHGSASIASNRPQLCDTPCTYMHHLVAFRVLSPVSPDPFLQTFFLYSALRFFATRCPSYFCGLPS
PVFMMLL

>ggPTHR2
VDADDVITKEEQIFLLLKAKAKCERHLKAKVPKVHDGFCLPEWDGIVCWPEGVPGKVVAMPCPEYIYDFNHKGHAYRRCDL
NGSWELVPGNNRTWANYSECAKFLTNETREREVFDRLYLIYTVGYSISLGSLTVAVLILGYFRRLHCTRNYIHMHLFVSFM
LRAVSIFVKDAVLYSGSALEEMERISEEDLKSITEAPPADKSQFVGCKVAVTFFLYFLATNYYWILVEGLYLHSLIFMAFF
SEKKYLWGFTLFGWGNLLVFATNGFLMGCQSLLRCWDLSAGNLKWIIQVPILAAIVVNFILFINIIRVLATKLRETNAGRC
DSRQQKLLKSTLVLMPLFGVHYIVFMAMPYTDVSGILWQVQMHYEMLFNSFQVQAEIKKSWSRWTLALDFKRKARSGSTTY
SYGPMVSHTSITNVATRGALALHLNTRLIPGTLNGHRNLPGYVKNGSISENSMPSSGPEQYNKDEEYLNGSGLYDGDRPTV
LVEEERETVM
>ggVIPR2
VNGVHPECRFHLEIHKEEAKCMELLKHAKPVDYKGCVGVWDNITCWRPAKIGETVTVPCPKIFSLVSGKPGNISKNCTSNG
WSDIFPDIFSTCGYNDYEDDYKVNFYVRVKAIYTLGHSVSLIALTTGSIILCLFRKLHCTRNYIHLNLFLSFILRAISVLV
KDDILYSSSNTAHCPEDQTSWVGCKAILVFFQYGVMANFYWLLVEGLYLHILLVLIFSPNRHFTVYLLIGWGIPTIFIITW
TVTRIILEDTGCWDTNEHGGPWWVIRIPILISIIVNFILFISIIRILLQKLRSPDVGGNDQSQYKRLAKSTLLLIPLFGVH
YTVFALFPDRSSNNYKIIFELCLGSFQGLIVAVLYCFLNSEVQGELKRKWRSLCWKQTAGRDYRLHSSISRNGSESVSQLH
RNSRAQSFMQTETTMI
>ggVIPR1
IMLEIEEERSQCLAEITEDNQTSGCRRQWDNITCWPEAQVGAVVVKPCPKYFRFLTTFLGNVSRNCTSQGWTDVYPAPYAV
ACGYDSNSTPGEEQTAFYGTVKTGYTIGHTLSLIALTAAMIIFCLFRKLHCTRNYIHMHLFMSFIMRAIAVFIKDVIVFES
GEPEHCFVSSVGCKAMMVFFQYCVMANFFWLLVEGLYLHTLLVISFFSERKYFWWYILIGWGAPSVFITAWTVVRIYFFNV
GCWEEIIETPIWWIIKTPILVSILVNFILFICIIRILVQKLHSPDVGHNETSQYSRLAKSTLLLIPLFGIHYIMFALFPDN
FKAEVKLVFELVVGSFQGFVVAVLYCFLNGEVQAELKRKWRRWHLERFLGSDMKYHHPSSGSNGTNFSTQISMLTKCSPKT
RRCSSFQAEFSLV
>ggGHRHR
MGCPWLNPNSCVLLLLLALPMLQVSCRTAWDGVSCWPAMPVGQSHAVACPDVPHIFKKSKRGYFITMKVLYTCGYSTSLAA
LFLAIGIFSCFRKLHCTRNSIHIHFFTSFILRGAAVLIKDAVLFSDESVDHCTMSTVKCKASIAFVQYSVLANFYWLLVEG
MYLQTLLLLTFTSDRSYIWWYILIGWGVPMLTVCVWVVTRLQYDDHGCWDDYSSPYWWVIKAPILLAIFVNFLIFLNVTRM
LAQKIRSPDISKNYKQQYMLTKSTLLLIPLFGVHYVVFAFFPEHIGVDARLYFELVLGSYQGFLVALLYCFLNGEVGIQHT
WDPPRWHPSPSWSSAAPTPWVPQLPAPILVSLQVQSEIKRNWGKWQSTMESNVFNMATQDFTA
>ggPTHR1
MGSVGRGGIVAALLCCCLLGSARALVDPDDVLTKEEQIYLLVEAREKCQRDIRAQLEKXXXXXXXXXXXXXXXXXXXXXXX
XXXXXXXXXXXXXXXXXXXXXXXXTSCLPEWDGII.RPKGSPSQEVAVPKRDYIYDFNHKXAYGRWEVTLSLNKTWANYTE
CAVLFSSESRSREKVFDRLHLMYTIGYSISLASLIVAVCILSYFKRLHCTRNYIHVHLFTSFICRAASIFLKDAVLYSGTL
GSEAKLREEELGAELGAELGPSPGQRSHLVGCKVVVTLFLYFLATNHYWILVEGLYLHSLIFMAFLSNKNYLWVLIIIGWG
LPAVFVSIWASVRASLADTQCWDLSAGNMKWIYQVPILAAVVVNFFLFLNIVRVLASKLWETNTGKPDPRQQKLLKSTLVL
MPLFGVHYVVFMAMPYTEVSGVLWQIQMHYEMLFNSSQGFFVAFIYCFCNGEVQAEIKKAHFRRSLALDFKQKARASSAAG
SCCYGGLMSHGTTNFSVSLTGRGPGGTQPRGLLLPARGSLPGYTPSSCAADLLPHLTQEMSQKTCGENTVGSKDPDESHPN
PNKELETML
>ggSCTR
IRAVPPVCDLLNVLKKEEENCAEILSLEARNRTPADDLLSSGRCAGMWDNMSCWPSSAVGYTVSAHCPKFFQMLTGKQGFV
YRNCTSEGWSDPYPRPDIACGYNVNDTTNEARRSYFMTLKTMYTIGYCTSLVTLTIALECYFIGMLLHRSSLCYRRLRCTR
NYIHMHLFTSFILRASSNFIKDNVLFSSEDTNYCDAYTAGCKLTMVFFQYCIMSNYSWLLVEGLYLHSLLVISFFSERKFL
WWFIALGWGAPTVFVAAWATARQLSENVGCWDVNTNANTWWIIRGPVVASIFVNFILFVDILRILMRKLSSPEGRSSDFNQ
YKRLAKSTLLLIPLFGVHYIIFAFFPEDASSGTMEIQLFFELALGSFQGFVVAILYCFLNGEVQLEVQRKWRQWHLSKHLR
QQLSTSASNGGSGVTQEMQVTKASPPQDRKATPQRSSIL
>ggCALCRL
XDSGQLTVTRNKIMTAQYECYQKIMQDPIHRKEGPYCNRTWDGWLCWSDVAAGTVSVQHCPDYFQDFNPSEKVTKICDSSG
NWFRHPESNRTWTNYTQCNIYTHEKVKTALNLYYLAIIGHGLSITSLLISLGIFFYFKSLSCQRITLHKNLFFSFVCNSVV
TIISLTAIANNQKLVAANPISCKVAMFIYQYLMGCNYFWMLCEGIHLHTLIVVAVFADKQHLMWYYLLGWGFPLIPASIHA
VARSLYYNDNCWISSDTHLLYIIHGPICAALLVNLFFLLNIVRVLITKLKDTHKAESNLYMKAVRATLILVPLLGIEFVLF
PWRPEGRITEEVYEYVMHILMHYQGLLVATIFCFFNGEVQAVLRRHWNQYKIQFEHSFSHSDAMRTVSYTVSTISDGPGYN
YNHDCTSEHLNGKGYHDMTV
>ggCALCRn1
XMVPSLTATVNYTDPTLEPVVTENSVIRQKIIDSQFKCYERMNRAPPYRKKGLFCNRTWDGWLCWDDTPAGRITAQNCPDY
FPDFDPTERASKYCDETGNWFRHPESNRTWSNYTLCNSFTSEKLKMAFILYYMAIVGHALSITSLLISLAIFFYFKSLSCQ
RITLHKNLFFSYVLNSMFTIAHLIIVVPNPGLVKRDPVSCKVLQFFHQYMLGCNYFWMLCEGIYLHTLIVVAVFAEEQRLH
WYYLLGWGFPLVPASIHAVARAKYFNDNCWMSVDTHLLYIVHGPVMAALLVNFFFLLNIVRVLVTKLRDTHRAESNMYMKA
VRATLILVPLLGIQFVIIPWRPENRLAGEIYDYIMHILMHYQVCYIXVQGALKRQWTQYKTQWGQRRREHCSTRSTSYTAT
SITEVPVYLYHHDSNNEQLNGRYVDDSELVALKSGETSA
>ggPACAP
MKFEHILPFRLCSAVQLLKTGLTDERHKLYEYVVTATHSNCIIKKEQETCLEKIRRAAALNPLNDSTPGCPGMWDNITCWK
PASVGEIVFVKCPALFRFIISEDGWEVDNLGQTHAGDYDLLESTAETPLGDISRNCTEDGWSEPFPHYYDACGFDENETES
DNQDYYYLSVKALYTVGYSTSLVSLTTAMVILCRFRKLHCTRNFIHMNLFVSFILRAISVFIKDGVLYAEQDGNHCFISTV
ECKAVMVFFHYCVMSNYFWLFIEGLYLFTLLVETFFPERRYFYWYTIIGWGTPTICVTVWAVLRLHFDDTGCWDMNDNTAL
WWVIKGPVVGSIMINFVLFIGIIVILVQKLQSPDIGGNESSIYLRLARSTLLLIPLFGIHYTVFAFSPENVSKRERLVFEL
GLGSFQGFVVAVLYCFLNGEVQSEIKRKWRSWKVNRYFAVDFKHRHPSLASSGVNGGTQLSILSKSSSQIRMSSINAENLA
T
>ggGIPR
AVAGCRLAQALTQYCVGANYGWLLAEGLFLHKLLVLATFSGDRCLPAFLLLGW

>ggGLP1R
STDGSLSGVVQKWKEYQLQCLKYLYEAPPIAAGKFCNRTFDNYACWPDGLPGTYVNVSCPWYLPWANTVLHGQVYRFCTSE
GTWLLKENSTLPWRNLTECEASDQVRDAPEEQLLNLSIIYTIGYALSFSALVIATAILLGFRHLHCTRNYIHLNLFTSFIL
RAISVFIKDSVVKWMYSTATQEHQWEGLISFQESLSCRLVFVMMQYCVAANYYWLLVEGMYLYTLLVLSVFSEQRIFRLYL
CIGWGVPMLFVILWGTVKYLYEDEGCWSRNYNMNYWLIIRLPILIAIGVNFLIFIRVICIIISKLQANLMCKTDIKCRLAK
STLTLIPLLGTHEVIFAFITDEHARGMLRFVKLFTELSFASFQGLMVAILYCFINNEVQMEFRKSWERWRLEHLYVQRDSS
MKPLKCPANSISSGGTVGSSVYAATCQATFS
>ggGCGR
CPQGPSAQITNFLFESWKAYSEECHRNMSRMPAPTELVCNRTFDKFSCWPDALPNTTVNVSCPWFLPWYQKVKHRYVFKTC
GPDGQWVTGPKGQSLRDATQCQIDAEDLEAH
>ggTAS2Rn2
MLPPVLIVSMCIVAIEVVVGFMGNGFITAVNSINWIKSKRISAADMILIFLSTSRFILQVTVMMYIHSLYFTDVFKLASVY
KTFGAIWMFVNHASLWFSTWLYVLYCVKIINVTQWLLLQIKHRITAMIPWLLLGSLLISSVTSFPLLWITPNTYLCRSTGN
CGDNSTAHISGWDSSHLYLLLLYFTGCFFPLVLSMITSGLLINSLWKHTKKMQCYVDSFRDPLIDVHLIAIKSIISFLILY
ISSFVAQILLTLSTSQSKDFVKVAVSLVVTGAYPSIHSIILIIVNSKLKLALRMLCQHFMCHLENVNL
>ggTAS2Rn3
MSSLFSSFCLVIAIFESVVGLLGNGTIVAVSSTSCIRSKILSSYDVIVIFLSLSRFFLQLWMILDFLLIFFCQPSYYEENL
FVTFKTVFIFLNSYSFWFAAWLSVFYCVKVASFTQSFLSWLKQRIASLIPWMLITSSLFSFATSLPFFWDSYNAHSNFTTP
LTMTNSSKRITTRKTNLIFLILLCNVGIALPSIMLVFSSILLIRSLWRHTRQMQNNATGFRDPSLEALIGAIKTVFSFLLL
YITNFIALILILSDTFVPLSTEEAICVVVVAACPAGQSMVLIWSNPRFRELLSSILHYVNSCVRARCS
>ggTAS2Rn1
MAEQHNTTSSSSAFVVIFAFQALAGMGINAFIVAASCMAWFKKKGMNSNEKILLFLGCFRFWYLCATWIYLIISVLFPQNL
LDTGISLTFAIFMCFLSSSNLWTSTCLYAFYLMKIANFRHHFFIYLKARIDRIVPWLLLSSVVLSLLNCSPFLKVIDEENR
TSPNFTTQGIFWKTNEEIRKHFNSIISICTCGFSMAFILVTLFAFFLLFSLCRHKHKMQTSSTRSLSMDAHIKAMKSLLSF
FFTFSIHYILLITTVYYSSKENFLVLLLLVLQYSFPVIHSLILIFSNPRLERIALRILPCAKCKECARQPTETPMLCS
>ggGPR97
TLVAVANGGCGVAVAFSIFTFAFYIFLRCSYKQFRSEDTLRINLGLHMNLVSSLFLLNLAFLLNSGLSSRASMGLCSALGG
LTHYCLLCCFTWTVLEGCHLYLLFVKVLGTYIHHYLLKLCIVGW
>ggGPR128
PLEYISYVGVGLSIAGLTITILFQIFTRKTRKSSITWMLVSLCFSMLIFNIIFISGIENHNARKDSSDTSGNNQQFSTYTT
TNTILTSDVVDPPADAWCMVVAVLLHYFLLATFMWTALNSAQLYLLLLRTMKPFPGHFIVTMSAIGWGVPAAVVAITLGAT
YGEGKALNYRQEEFCWLAALDQNQNFSMKKPMFWSFLLPVAFILLFNIIIFVEIIVSVIWKENKNLTRNKKDSLVKKMTAT
ISIVVVLGITWTIGYLMLISHEETNLFFSYAFCIFNATQGLQICILYTFR
>ggGPR114
IQAHDLSESELQSLTYISTVGCSISAAAALCTLLLCCVFRCRPRDDTTRIHMNLLAALLLLNVSFLLSEPLAAGTTGGCQA
AAALLHGSLLCALGWMGAEGFHLYLLLIKVYNIYVRHYLLKLCLCTWGECWLTSPSAHYVTLCYAGLILLFNVLVLVAVVA
VLRRTRQQQQQRARKDWMTVLGLTCLLGTTWGTAFCAFGVLLVPQLYLFNVLNSLQRQQP
>ggGPR144
QRTTKDELTLQTLTFIGCGVSFCALVVTFILFLVVGVPKSERTTVHKNLIFALAAAEALLMFSELAKTNQVLCFLVTAFLH
LSFMAAFSWMLVEGLLLWSKVVAVNMSEDRRMKFYYATGWGLPAITVGLTLATSFNNYVADNYCWLNVQTNVIWAFVGPVL
FILGVNTFVLFRVVMVTVSSARRRAKMLTLNSSLENQIGIQIWATAKPILVLLPVLGLTWVCGILVHLSIIWAYIFIMLNS
LQGLYIFLVYAIYNSE
>ggGPR133
ALSSITYIGCALSIFCLTITLVTFAVLSVCDTIRNQRYHIHANLSFAVLVAQILLVTSFQFSPGTVPCKILAILLHFFFLS
AFAWMLVEGFHLYSMVIKVFGSEESKHLYYYGIGWGCPLVICVISATSSLDSYGESDNCWLSLENGAIWAFVAPALFVILV
VNIGILIAVTRVISRISADNYKVHGDANAFKLTAKAVAVLLPILGSSWIFGILAINAHTLIFQYMFAVFNSLQGFFMFLFH
CLLNSE
>ggCELSR1
PNIGRLGIPHGPSGEKVAVVTVDDCDTAVAVRFGSLIGNYTCAAQGTQTGSKKSLDLTGPLLLGGVPNLPEDFPVHNRQFI
GCMRNLSIDSKPIDMASFIANNGTLPGCAAQRNYCEANWCQNGGTCVNKWSTYICECPVQYGGKNCEQVMPSPQRFSGESI
IIWSELDITISVPWYIGLMFRTRKVNGMLMQANAGTASKINIQILNNYVQFEVYSGMTQVASLKMTQSRVSDGEWHHLLIE
LKSAKDGKDIKYLAVMSLDYGMYQSTVQIGNQLPGLKMKSIIVGGVSGDQVSVQQGFYCXXYFGRDCVDVCNLNPCEHVST
CVHKPSSSHGYTCECGQSYYGQYCESKIDLPCPRGWWGNPICGPCNCETSKGFDPDCNKTNGECHCKANYYRPQSSDTCYP
CDCFPSGSHTRSCDMETGQCPCKPGVIGRQCNRCDNPFAEVTARGCEVIYNGCPKAFEAGIWWPQTKFGQPAAVPCPKGSV
GNAVRHCNIEKGWLPPELFNCTTNTFVDLKVMNEKLHRNETILDGDKTIQIVRVLQNATKYTHSMYGNDVRTAYQMMIRVL
RYESQQQGFDLAATRDVEFNENIIKVGSALLDPNNKEQWEQIQRTEGGTAHLLRHYEDYFNNVAQNMKKTYMRPFVIVAAN
MIIAVDIFDKSNFTGARIPRFHEIKEDYPKDLESSVIFPDTLFRPSDRKAVPTMRPSNQKLSSKVNSDARSPESMFTKRRK
RHPDDSTHHTVAMVIIYRSLGHLLPENYDPDRRSLRLPNRPIINTPVVSTAVHSDGDLPPNLVEKPIIVEYTMLETEERTK
PVCVFWNHSIMIGGTGAWSSRGCELFSRNQSHIACQCNHLTSFAVLMDISKRENGEVLPLKIVTYTTVSISLVALLITFIL
LVLIRTLRSNLHSIHKNLVAALFFSELVFLIGINQTENPFVCTVIAILLHYFYMSTFAWMFVEQLHIYRMLTEVRNINFGH
MRFNNVVGGGIPAIITGLAVGLDPQGYGNPDFCWLSVHDTLIWSFAGPIVIVVVINTVIFILAMKASCRRRQRSFEKTGVI
SVLRTAFLLLLLISATWLLGLMAVNSDVMTFHYLFAIFSCLQGLFIFFFHCVFNKEVRKHLKNTLTGKKPLPDDSTATRAT
LLTRSLNCNNTYIEEPNMYRTTIGESTVSLESTVXXHDSDSDSELSLDEHSSSYASSHSSDSEEDGLEPEKKWNTSTAKNN
ERGPLHSTPKVDTLPNHVKPYWPTECITASDSEDPSGKQKLKVETKVNVELHRENQVNHSNEAPQDKENDGQQKENRPLSH
QNNQQPEQRKGILKNKVTYPPPLIDKNMKNRLREKLSDYNQTTISSRTTSLGTNDGVRSPSDSGVTVKNARREQSRDQLNG
MAMNVHVGTGHAETSDSE
>ggCELSR3
XAALPRPRRGSRAAPNRHPLFPQYNYQAEVAENQPAGTAVVAVAAQDPDGGEAGRLVYSMDALMNSRSLELFSIDHHAGLI

TTTQALDRESMELHYFRVTAADHGAPRLSATTMVAITVADRNDHDPVFEQGEYRETIRENVEEGYPILQLRATDVDSPPNA
NIRYRFVNERAAHAVFEIDPRSGLITTSGPVDREKMERYSLVVEANDQGREPGPRSATVRVYITVLDENDNTPQFSEKRYI
VQVREDIRPHTEILRVTATDLDKDNNALVHYNIISGNSRGQFSIDSVTGEIQVVAPLDFEVEREYALRIRAQDAGRPPLSN
NTGMASIQVVDINDHAPIFVSTPFQISVLENAPLGHSVIHIQAVDADYGENARLEYKLTGVSADTPFVVNSATGWITVSGP
LDRELVEHYFFGVEARDHGSPSLSASASVTITVMDVNDNRPEFTQKEYFIRLNEDAAVGTSVLSVTAIDRDVNSAITYQIT
GGNTRNRFAISTQGGVGIITLSLPLDYKQERRYVLTVTASDRTLRDNCHVHINITDANTHRPVFQSAHYSVSINEDRPVGS
TVVVISATDDDVGENARITYYLEDNVPQFRIDPDSGAITLQAELDYEDQVTYTLAITAKDNGIPQKADTTYVEIMVNDVND
NAPQFVSTHYQGVISEDAPPFTSVLQISATDRDAHTNGRVQYTFQNGEDGDGDFTIEPTSGIIRTVRRLDRENVPVYELTA
YAVDRGIPAQRTPVHIQVTIQDVNDNAPVFPAEEFEVLVKENSIVGSVVAQITAIDPDEGPNAQIMYQIVEGNIPEIFQMD
IFSGELTALIDLDYETKPEYVIVVQATSAPLVSRATVHIKLIDQNDNSPVLKNFQILFNNYVSNKSNTFPSGVIGKVPAYD
PDVSDRLFYTFERGNELHLLIVNQTSGELRLSRKLDNNRPLVASMLVTVTDGIHSVTAQCVLRVIIITEDMLANSITVRLE
NMWQERFLSPLLATFLEGVATVLATPKEDVFIFNIQNDTDVGGTVLNVSFSALAPRGGHYFSSEELQEQLYMKRMALTGAS
MLEVLPFDDNVCLREPCQNYMKCISVLKFDSSAPFIASPSTLFRPIHPITGLRCRCPQGFTGDYCETEINLCYSNPCLHGG
TCTRKEGGYTCVCRQHFSGENCEVDSRSGRCQPGVCRNGGTCTNGADGGFRCQCPAGGFETPFCELSTRSFPPRSFVMFRG
LRQRFHLTLSLSFSTVEPGGLLLYNGRLNERHDFLAVEIIQGQVQLKYSTGESSTVVSPYLPGGVSDGQWHTLQLRYYNKP
KVSALGVVQGPSKDKVAILTVDECDASVALQFGSEIGNYSCAAEGVQTSSKKSLDLTGPLLLGGVPNLPENFPVSHRDFVG
CMRDLYIDNKRIDLASYIANNGTTAGCHAKHSFCDSSPCKNGGTCSVSWGTYSCLCPVGFGGKDCRHAMHHAHYFQGNSVL
SWDFKADMKISVPWYLGLAFRTRQMDGVLLQAHAGQYTTLLCQLSGGLLSFMVSRGSGRSTSLVLDQLQLNDGRWHDLQLE
LRDVRSGRDSRYVITIMLDFGLYQDTVVVGNELHGLKVKHLHVGGVLGSGEVQNGLRGCIQGVRLGDSVTGTVLPKPSHAL
RVEAGCSVPSPCDSNPCPANSICKDEWQSYSCVCQPGYYGGECVDACHLNPCKNKSVCRRKPGSPLGYVCECGGNFFGQYC
EHRMDQQCPKGWWGNPSCGPCNCDVSKGFDPDCNKTNGQCHCKDFHYRPKGSDTCLPCDCYPVGSSSRSCNKETGRCHCRP
GVIGRQCNSCDSPFAEVTPSGCKVLYDGCPKSLKAGVWWPQTKFGFSAVVLCPKGSLGAAVRHCDEEKGWLEPDLFNCTSP
AFKELSMLLEGLERNETELNTIEAKKLAHRLRAVTDHMDHYFGNDVHITYRLLSRLMAFESRQHGFGLTATQDAHFNEXTN
PSGFWTARDCDLVYRNTTHVHCQCSQFGTFGVLMDSSHREQLEGDLETLAIVTYSLVSLSLVALLLTFSFLTCLKGLKSNT
RGIHSNISVTLFFSELLFLLGINRTENQFLCTVIAILLHCFFLSTFAWLFVQGLHIYRMQTEARNVNFGAMRFYYAIGWGV
PAIITGLAVGLDPEGYGNPDFCWISVHDKLVWSFAGPITVVIVMNGVMFLLVAKMSCSPGQKETKKKSVLMTLRSSFVLLL
VISATWLFGLLAVNNSVLAFHYLYTVLCSLQGLAVLVLFCVLNEEVQEAWKLACLGKKGQSEEAARSTQGPNTYNNTALFE
ESGLIRITLGASTISSVSSVRSARTHSSQRGYLRDNMTARQGSALDHSLLGHAGPTDIDVAMFHRDAGGDQDSDSDSDLSL
DEERSLSIPSSESEENVRLRGRFPRQFKRAAHSERLLTNPTNTAPKGKFHVDGNDLMSYWPALGECEVHPCSLQKWGSERK
LGFDINKDAANNNQPDLALTSGDENSLTQTQRQRKGILKNRLQYPPALQGLPAVGRMTNELSWYKTSTLGHRAVPAASYGR
IYSGAGSLSQPASRYSSREQLDMLMRRQMSREQLSRNNSGECLETVPSRHGSREELDTIPSRHGSTEHLENLPSRHGSREN
LDLLAPRPSQRDHGNTLPRRQGSRDHLETLPCRFGSREQLDCGLVREVSREWLNTLPSRQGSRDRIDRLPSRDTSREQLDL
LSRRQPSRDQLASSRQTSREHLDFLSRKSNSREPLGTVPSRQPSQENLGSLSRRQLSRESLEPLSRRQPSRENLEAIPSRH
PSTEQLDILSSILASFNTSVLSSVQSSSTPSGPQTTANPSGMHTSTPSAMCPSTPHSSTSHSISELSPDSE.
>ggLEC2
MVSSGCRMQSFWFLLIISFLQCTEGFSRAALPFGLVRRELSCEGYSIDLRCPGSDVIMIESANYGRTDDKICDADPFQMEN
TECFLPDAYKIMTQRCNNRTQCIVVTGSDVFPDPCPGTYKYLEVQYECVPYIFVCPGTLKAIVDSPYLYEAEQKAGAWCKD
PLQAADKIYFMPWTPYRTDTLIEYASLEDFQNGRQQTTYKLPNRVDGTGFVVYDGAVFFNKERTRNIVKFDLRTRIKSGEA
IINYANYHDTSPYRWGGKTDIDLAVDENGLWVIYATEQNNGMIVISQLNPYTLRFEATWETTYDKRAASNAFMICGVLYVV
RSVYQDNESETGRNAIDYIYNTRLSRGEHVDVPFPNQYQYIAAVDYNPRDNQLYVWNNNFILRYSLEFGPPDPAQVPTTAV
TITSSAELYKTTASTTSTTSQKGPVSTTLAGGTKEGSRGPKPPPPISTTKIPPVTSFFPLPERFCEPTEARGISWPQTQRG
MMVERPCPKGTRGTASYLCVVLTGTWNPKGPDLSNCTSHWVNQLAQKIRSGENAASLANELAKHTKGPVFAGDVSSSVRLM
EQLVDILDAQLQELRPSEKDSAGRSYNKAIVDTVDNLLRPEALESWKDMNSSEQAHAATMLLDTLEEGAFVLADNLLEPTR
VSMPTENIVLDVAVLITEGQVQDLKFPQGSKGGNSIQLSASTVKQNSRNGLAKLVFIIYKSLGRFLSTENATIKLGSDFAG
RNSTIAVNSHVIAASINKESSRVYLTDPVYFTLEHIDPDNYFNANCSFWNYSERTMMGYWSTQGCKLVDTNKTHTTCACSH
LTNFAILMAHREIYKDGVHELLLTVITWVGIVISLVCLAICIFTFCFFRGLQSDRNTIHKNLCINLFIAEFIFLIGIDKTE
YKIACPIFAGLLHFFFLAAFAWMCLEGVQLYLMLVQVFESEYSRKKYYYVAGYLFPATVVGVSAAIDYKSYGTEKACWLHV
DNYFIWSFIGPVTFIILLNLIFLVITLCKMVKHSNTLKPDSSRLENIKSWVLGAFALLCLLGLTWSFGLLFINEGTVVMTY
LFTVFNAFQGMFIFIFHCALQKKVRKEYGKCFRHSYCCGGLPTESPHSSVKASTTRTSARYSSGTQSRIRRMWNDTVRKQS
ESSFISGDINSTSTLNQGHSLNNARDTSAMDTLPLNGNFNNSYSLRNGDYNDSVQVVDCGLSLNDTAFEKMIISELVHNNL
RGSSKNHNLERTLPAKAVIGGSSSEDDAIVADASSLMHSDTPGLELHHKELEAPLIPQRTHSLLYQPQKKVKTEGTDSYVS
QLTAEADDHLQSPNRDSLYTSMPNLRDSPYPESSPDVEEDLSPSRRSENEDIYYKSMPNLGAGHQLQMYYQISRGNSDGYI
IPINKEGCIPEGDVREGQMQLVTSL
>ggLEC3
ILAAFSRAPVPMAVVRRELSCESYPIELRCPGTDVIMIESANYGRTDDKICDSDPAQMENIRCYLPDAYKIMTQRCSNRTQ
CAVVAGPDVFPDPCPGTYKYLEVQYECVPYIFLCPGLLKGVYQSEHLFESDHQSGAWCKDPLQASDKIYYMPWTPYRTDTL
TEYSSKDDFIAGRPTTTYKLPHRVDGTGFVVYDGALFFNKERTRNIVKFDLRTRIKSGEAIIANANYHDTSPYRWGGKSDI
DLAVDENGLWVIYATEQNNGKIVISQLNPYTLRIEGTWDTAYDKRSASNAFMICGILYVVKSVYEDDDNEATGNKIDYIYN
TDQSKDSMVDVPFFPNSYQYIAAVDYNPRDNLLYVWNNYHVVKYSLDFGPLDSRAGQAHHGQVSYIAPPIHLDSDLERPPSK
GVSTSGALGPVSTTTSTTPRMATSSTGRPTTTSALGRRNRSTSMPSPMADVPDEMTTHLPPASSQLPPAGAESCDPMEARD
IMWSRTRQGQVAKQPCPMGSIGXMKSGEMAANIARELAEHTRNHLYAGDITYSVSAMVQLVNLLDVQLRNLTPGGKDSAAR
SLNKLQKRERSCRAYVQAMVETVNNLLQPQALNAWRDLNASEQQRAATKLLDTVEDSAFVLADNLLKTDIVRENTDNIQLE
VARLSTDGNLEDLKFPQNTGHGSAIQLSANTLKQNGRNGEIRVAFVLYNNLGTYLSTENASMKLGTEAMSTNHSVIVNSPV
ITAAINKEFSNKVYLADPVVFTVKHIKQSEENFNPNCSFWSYAKRTMTGYWSTQGCRLLTTNKTHTTCSCNHLTNFAVLMA
HVEVKHSDAVHDLLLDFITWVGILLSLVCLLICIFTFCFFRGLQSDRNTIHKNLCISLFVAELLFLIGINRTDQPIACAVF
AALLHFFFLAAFTWMFLEGVQLYIMLVEVFESEHSRRKYFYLVGYGMPALIVAVSAAVDYRSYGTDKVCWLRLDTYFIWSF
IGPATLIIMLNVIFLGIALYKMFHHTAILKPESGCLDNINYEDNRPFIKSWVIGAIALLCLLGLTWAFGLMYINESTVIMA
YLFTIFNSLQGMFIFIFHCVLQKKVRKEYGKCLRTHCCSGKSTESSIGSGKTSGSRTPGRYSTGSQSRIRRMWNDTVRKQS

ESSFITGDINSSASLNREPYRETSMGVKLNIAYQIGASEQCQGYKCHGYSTTEW.
>ggVLGR1
MFTTFFFNYYYFYCVLESGFDFTIKENGLQVDQPPIIGNISTIRVTIAKSDNAEGIIEFDPQYLLLQVEEDAGVITIPVVR
KRGTYGYVTADFLSRSISALPNGVDYVIHNNSVTFHHGQNQTFIYISIIDDEESEFAEQFEIQLTGATGGAVLGLHLVSQI
TIAKSDSPLGNVRFLNKSRIILPNPXXPPXVSLLKERTGRLLGDTQINWDILGPNSEEILSPLNSDIGDPVNGSFYFGEGE
GGLRTIHLQIYPHEDIEVQEIFVIRLSLVKGETEIDPKSNNITLIIQKFGDPNGIVQFAPESLFERNFTEPSAVEGPQNIT
LLCMFLTLCVILFSFAFLNSFQVHWKLYSEFDITSDFLTTEGSVVIADQQSTGEIIIYLLPDDVPELDENYIVQLISVEGG
ADLDEDKRISKINVFANDDPHGVFALYSDHQLILVEKDLDRYVQINVTRHAGTFGEVVIEYHIFSHQEEELIAPENERGYF
TIKDGASFAVKRVPISSQAFLLLDLNFTLELINVSLVRESAKYVPKILEKAKSAVVLVPAEAANSQVGFESVILQLTNIVA
GTGQAVITRKGVYGSVTVNWISGYPPELTTDFVQPGNITPSFGIVLFSSGEQSKVITFRATPNINKRESFAISLTEVHSNV
SGGAQLRSGFTIAEIEPMGIFQFAFNSTFISVEEDVESVTLHVQKLFGFRSNVTKLTYQTTPGSAKPLEDFRPIHNGELIF
AHFQTNAMIEISIIDDTLSEIEEYFFVNLTAVEILDFQPAKLDWSPRLNPAFSVATINILANDILHGILSLGPEFIYVEED
ANNNTPNTVIINIRRTQGFTGDIEVTIKTFGGISAQNGVGLYPFENVYGKSNFTWAMEGEDFEEQSVSLTLLDGERESKVS
IKIFDDDEPEGQEFFYVFLSDPKHGAQIVKGKDEYGFAAFATVIIAGSDLQNGILGFIPEVQSGLILDEDSENREVLLVVA
RQPNRAFEDVKVFWRVTFNETSAVLLKDGVNLENELVSVLGVTTCMAGQTMCNISIEIKPDNVPEFETYFFVELYDVSAGA
ALNSSARFAFVTVLESDAPHGLVYFAVGSRFAVAHKKTTLISLQVLRDSSTSVTTKVSYNMQELQKPETVGRTFISPAVAG
QDFARSEGLLTFEPGQRNKFLDVTLTPETASLNPFPKRFQVVLSNPPGGARVDDVYGVANVTIVSDLGSLPFWMLIDQLHQ
PLDDMILQRVLPNLNVKVATETTEEQLTAVMHVIDKVTDVGEKHLLTGKSRSLFYEILCALASPKRKDTRGYSLLAEVTEK
FAFSLLTEIMCGSPGERGKNILDTCPYIAIMAHNWYPQQINGHRFDGRDGDSIQVPEHLLKVSLVLSPPQEENCESIQFTE
YSSQQWFLTGDKELALKNKVFSMSLKNQSSHPLKDSNEVIYRIYATGSRIVPQKSLCLLWNQAAESWLSDSGFCRVIDDTS
DYVECACSHMSIYAVYAQTDDLSSYNEAFFSSGFICISGFTLAIISHLFCTRCAMFAAKLLTHMMVACLGTQISFLASAYL
SQHLSEESCSALASITHYLYLCQFSWMLIQAVNFWYVLVMNDEHTERRYLLYFLLSWGLPAFIVILLLIILRGIYHHSTAQ
IYGLVYSDLCFIPNAYAALFTAALMPLMCLVVVFVVFVHAYQVTPQWKAYDDIFRGRTNAAEIPLVLYLFALISITWLWGG
LHMAYRHLWMLVFFIIFNSLQGLYVFVVYFILHNQLCCPVKASYTVDMNGHSTPGSAFFTHGSGLPAAGGEISKSTQNLIS
AMEEVTVPADWERASLQPASQASSAFKQSPQNGNVFHPSGGFSNSSLVADEESQEFDDLIFALKTGDVSDNESCHGSQDGG
SMANSQIVELRRIPIADTHL
>ggGPR112
XLLDCPESISQTKYKGSYSWPQTSPAATVEVSCGKNPLQSAWRKCAINIDLEQSFWGKPNMTECKLLEELPSNILDLHNVI
ITEENAQDVVQHILYLLPDATLDMEELEVIGNKISDIVKFLDLSMTLAEAILTILDYILLQEMEDQNIRKITNRILKTTEE
IGYKLAHTERNTSVITTSFALLVMRPDPSVFEGVAFGVTSYDQGRDIKDDSLQKERLNTYVVSASVENSLIQNLNEPVNVT
LQHINKNTGNAAVHCVFWDFLKNDGLGGWNTTGCETEYTDMNYTICFCNHLTHFGVLLDLSRAEIDATNDRVLTLISYAGC
GASSLFLAITLVTYLALEKLRRDNPAKILLNLCAALLMLNVIFLVNPWLSSYNLPAVCIAVAALLHYFLLAAFTWMCMESV
HLYLALVKVFNVYIPKYILKCCIAGWGIPAIVTIIVLIINKDFYGNGSQSENSPFSNFCWIQDNVVFYVSVVAYIFLVFLT
NTAMFITVLLQIHSVKSRTQMRSRFWKRFFLQDLKSAFSLMFLLGLTWSFAFFAWGAVRIFFLYLFSIFNTLQ
>ggGPR115
XLIPCEDSGTLCIQACSPSFHGETSFVCKDRTWRMFTDACANLDVQSLFQVMISKREPLPSSEGHIAGGHFPFVRPGKPMH
GKPGDGAKYFGADDRGKSNCQADFSCIIPDILSSPAIPGNIADIVELLKMISLMLSEDVNRGKMQSYSKIANHILNSSVIS
NWAFVKDRNASSTLLDSVNLFAGKLLLRNGSEHIQEHFISTKGFSIHRNASGKSFDFSMEVNKTNYVSGRVVIPEEELQKL
PNTSKAISIAFPTLGAIIEASRPDPISVNGMVLSVSLPEELQHILLTFEKVNKLESVGAQCVGWHSTERRWDNKACQMKSD
NISSVVCMCKHRRRTFRSFSILMSPTLLRNTVLDYITCVGLGLSIFSLILCLTIEMAVWHHVTKTEITYMRHFCLVNIATS
LLIADILFIVAAVVHNTALNYRLCVAATFFLHLFYLALFFWMFTLGLLILYGLLLVFFKITRSVFIVTAFVIGYGCPLVIS
ILTVAITEPKNGYLRSGACWLNWYETKALLAFAVPALSIVAVNLAVVVVVVVKTPRPSIGESCKSRDLSNMIRISKNVALL
TPLLGLTWGFGLATVVDSHSLAFHVTFALLNAFQGFFILLFGTLLDRKVL
>ggGPR116
QVGREVGEESFHPELQRQKRHVILFDLPPLEYAIDIEVSLTNSSFLAPIKEYFRNLHLPVSINISDVEMTVSSINITTVCV
PSENKSCCSCESGYAWPSTGCSDLISCPALSRAPNQPCGYTRERPFNGPYCEPQNGDSCGMGEPTVMNMSVRLDTAFHDDL
RNHSSQLYRKYKADLDKAFKASYGCLPGFVSATTTGFRFSAWKAELVQVHLHKAVWDLWLMQMVEEDPVGKCSICYQRQFP
AAKAEYRTNFTVSPAHIFEGDTIQMVCEINTTSENMTWYRDSQIILNSSRHSVRTDNNTAMSQAFLTITNVTQEDSGTYKC
TFANRYRSLNLIYEGTKEIQVSPLRIISYSNDYKVHCNSPEIQANSPVLFCCIDGQLPLLTGDWKVNGAISITGVPSFSSN
CTEYKLNMTDSLCLAEHSGTVTTYTCELQTGHGARSSRDIRVMYFRAAQVRISSSVNTTVSVGYGFSLKCESDTSNYDSIS
WEIHSGDRIQAVECAACIRTNKSMATSMLVVNTATQDWNGTYICTLSQDSLNSSASVTINVIPLPLKQHILIDPAGAPIMC
GTPQALTCCISANSMGDYDVKFVVQEKEFQAEKEEQGNFFCYSYNYTETECREKKLEAYCKFVNSNEQEVSSNSINLSLIP
DKDLISCSENNTFGKEGDILKKSCSVLNTTFIRGSNIYKCFNSSWKMEKNDCVSEQINNLLLMAESLVNSPSAKVGLPNFL
QNLRNQTELLQNDNSSANLAAIVTILYNISSIPVEAKQSTISDYFTTVDIIVTDFKKELWADLNEGQVPISSLLLNAVERF
SENLQPVNNTIPHLSTETLELQGMVVTEHSHADYNKNFSRLGNLTANVLIDNIETLPPNSTIVSVAYSALGHILPVKENMS
VNSLVISTALSCERNQNFHINMTFQKENISLKMPLCVFWDFKLNESRGGWNDRGCTQTEEGSNVICSCNHLTPFSILMSPD
EPAHNSVEDYITHIGLAISILSLVVCIVIESLVWKTVTNNTTSYMRHVCILNTATSLLIADIWFIVTASINVQKRNICIAA
TFFIHLFYLCGFFWMLSLGLILFYRLVFILHNTSKTSQKAVVFCLGYGCPFVFAVTTIAVTLPWNTYTRKDACWLNWKDSK
AVLAFVIPALAIVAMNLFITAVVIIKILRPNIGDRTNKQERKTFFQICKSLVILTPLLGLTWGFGLATIIRNSHQTFHILF
ALLNALQGLFILVFGTLWDNK
>ggGPR123
DFKTVLSFPQYPGEFLHPVVYACTAVMLLCLFASIITYIVHHSTIRISRKGWHMLLNFCFHTALTFAVFAGGINRIKYPII
CQAVGIVLHYSTLSTMLWIGVTARNIYKQVTKKTQSCQNGDQPSYPKQPLLRFYLISGGVPFIICGITAATNINNYGIEGN
APYCWMAWEPSLGAFYGPIAFIVLVTCIYFLCTYIQLKRHPERKYELKERTEDPQRMPSTEVGHSHITDAASIPQAACSMI
SSSLLENEHSFKAQLRAAAFTLFLFTATWTFGALAVSQGHFLDMIFSCLYGAFCVTLGLFILIHHCAKRDDVWHCWWSCCP
SRRNTYSVQVNVRPKVNVNGDTQVHAPCLQESPCPNKSAVFNHPAASHCKLTNLQAAQNHVNCLSPVTPCCAKMHCDQLLD
DEAHIHVHNEGSFRPNMHIHRCLKSRTKPRYFSRHRSAGEREYAYHIPSSIDGSIHSSHTDSPHSTHDSQSGHRRACCSKS
DPYPTVNQPESSDASTVIYSCTKMPESDTVHHPAHFEMHPRTQSLPFNTTNHNGILKGNVHEAMIYSSDSTGNIKTGPWKN

ETTV
>ggGPR124
XLLSNNKITVLQNGSFFGLRVLEKLDLKNNLISTVQPGAFLGLPELKRLDLSNNRIGCLSASVFQGLTNLLRLNMSGNIFS
SLPPGVFDELPSLKVVDFATEYLTCDCNLRWVLPWARNYSVQISERTLCVYPRHLHTTPLRSAQESQLQCAGTPELHTHHL
IPSLRQVVFQGDRLPFQCTATYLDNSTQIHWYHNRQRVQEDERAGVIVEESLIHDCTFITSELILSNIHVSANGEWECAVS
TSQGNVSKKVEIVVLETSASYCPAERVTNNRGDFRWPRTLAGITAFQSCLQYPFASGPASGGPAAEKQASRRCDRAGRWEE
GDYSHCLYTNDITRVLYTFVLMPINASNALTLAHQLRVYTAEAANFSDMVDVLYVAQMIEKFIGYVDQIKQLTDVIVEMAS
NIMLVDDHILWMSQKEEKACTSIVRSLERIAAHTLSSNSQHMAVVSPCTTIHITRKCWHMLLNLCFHMAMTSAVFAGGITL
TGYLVVCQAVGIILHYSSLSTLLWMVVKARVLYKEATWKAPRQPDGDSAPPAPRPMLRFYLIAGGIPLIICGITAAVNIHN
YHDNNPYCWLVWRPSLGAFYVPVAFILLVTWLYLLCAGLSLQCHAPRRKEPPELPGTLPRLGANGELLTDSGSISVTLHSG
THCPEVDGVYSLQAQLWALLAVHAMYVALWTFGAMAVSQRGDLNVVFSCLYGVTAVALGLFIFIHHCVRRRDVLGSWFSCC
PSYRSALPMQPYSHPGTAAVEDGSQMFIGCDPEAARSATSSSSPSSAGSAAGRCKLTNLRAAQSQAEGPEHGDGRSAGAGR
HSNNHRRNHKSRTKQHREGKHHRLKVLRGPSSEQSESGSVHNSHSESYHSSRNSPMNNCAAPRAAGPQDGEVAPGQSEGSD
GFVEARRRSRDNVRQAEREAKRRSYPLNVGSHNGGLKGSKYDVNLAGADSGAGMRSGLWKSETTV
>ggGPR125
XPPLELPSFYMTPSHRQVVFEGDSLPFQCMASYIDQDMQVLWYQDGKIVETDESQGIFVEKNMIHNCSLIASAPSKSNIQA
GSTGNWGCHVQTRRGNNSRTVDIVVLESSAQYCPPERVVNNKGDFRWPRTLAGITAYLLCTRYSAGSGIYPGNSQDERRAW
RRCDRGGYWAEEDYSRCQYANDVTRVLYMFNQMPLNLTNALATARQLLAYTVEAANFSDKMDVIFVAEMIEKFGRFAEKYK
ELGDVMVDIASNIMLADERVLWMAQRESKACTRIVQCLQKIAMYRLANGAQVYSTYSPNIALEAYVIKAAGFTGMTCTVFQ
KVATSDRTGLSDYGRRDPDGNLDKQLSFKCNVSNTLSSLALKQKREIRPADESVYKLQLIAFRNGKLFPATGNSTNLADDG
KRRTVVTPVILTKIDGLNPASHHVPINVTLRRIAHGADAVAAQWDFDLLNGQGGWKSDGCRILLSDENITTIQCYSLSNYA
VLMDLTEAELYTQPADLLHPVIYATAMVLLMCLLMIIVSYIYHHSVIRISVKSWHMLVNLSFHIFLTCVMFIGGITQTRNA
SICQAVGIILHYSTLATVLWLGVTSRNIYKQVTKKAKRCQDTDEPPPPPRPMLRFYLIGGGIPVIVCGITAAANIRNYGSR
PNAPYCWMAWEPSLGAFYGPASFIIFINCMYFLSIFIQLKRHPERKYELKEPAEEQQRLATNEHGELTHQDSLSVSLVSTS
ALENEHSFQAQLMGVSLTLLLYIALWIFGALSVSLYYPMDLIFSCFFGATCLSLSAFLMVHHCVNREDVRHAWITTCCPGR
STYSVQVNVQPSSSSGTNGETPKCTNSSAESSCTNKSAASLKNSSQGCKLTNLQAAAAQCHSTSLPLNTGPQLDNSLTEHS
VDNDIKMHVAPLEVQFRTNGHPSRHHKNRSKGHRASRLAVLREYAYDVPTSVEGSVQNGVPKSRPSNSEGHSRSRRAYLAY
RERQYNQSQQDSSDACSTLPKSSRNTEKAIAANNKKEILRKPIVVELENQQKSYGLNLAIQNGPLKSSGQDGPLLTADSTG
NIRTGLWKHETTV
>ggGPR126
IDFDKDQTNAIASVILPPSLLKNLSQDDFEVISRAQFTFFNKNGLFQDAETPGNLTSFVVACSIGNITIRDLQEYVKITIK
HTKIQEDPKPTCVFWDMAKNGGNGGWNPTGCKVQADSNENETVCLCNHLTHFGVLMDLQGTATEIDAQNTKILTFITYIGC
GISAIFSAATLLTYIAFEKLRRDYPSKILMNLSTALLFLNLTFLLDGWIASFDIDGLCIAIAALLHYFLLATFTWMGLEAV
HMYIALVKVFNTYIRRYILKFCIIGWGLPALVIAIVLASTNKNASRVYGKEFCWIKNKVVFYVTCAGYFGIMFLLNVAMFV
VVMVQICGRNGKRTNRTLKEEILRNLRSVVSLTFLLGMTWGFAFFAWGPLTLPFLYLFSIFNSLQGLFIFVFHCAMKENVQ
KQWRRHLCCGRFRLADNSDWSKTATNIIKKSSDNLGKSLSSSSIGSNSTYLTSKSKSTTNTYCKRSSHTENIFVDKPPSKF
AQEDGEQTSIIPVHQVIDKVKGYCNSHSDNFYKNIIMSDTFSHSTKF
>ggBAI1
MRSIGSALNLLLVSPLYFVWTVVALDLAQTPCTTLVQGKFFGYFSSFAVFPLNSSLCSWIIQNPDPRRYTLYMKILKPTGI
CDHQHIRTYQFDSFLESTRTYLGMESFDDVLKLCDGSTRYAFLQSNKQFLQMKQTAPPGVENGPVRWKPIKAQERDFSVEY
LVVGNRNPSKAACQMLCKWLDACLAISSSSHPCGIMQTPCSCSGGEVLDQASEILGFPKKKEDCYKDGIYLENCLSSPKDS
SGVEFLGGWNTWSAWGDCTKDCGGGLQTRMRTCKPLPNEGLACEGVLEEGRLCNRKACNTNGRYNSRSQSLRSTDNRKRED
TDELQHYGYPAPQIGDPAAEEWSPWSVCSTTCGEGWQTRTRFCVSSSYSTQCSGPLREQRQCNNSAICPVHGTWDEWSPWS
LCSSTCGRGYRDRTRTCKPPQFGGNPCEGPEKQTKFCNIALCPVDGNWNEWSSWSSCSASCSNGTQQRTRECNGPSYGGAE
CQGHWVETRDCFLRQCPVDGKWQAWGVWGSCTTTCGGGTQRRDRVCYGPFFGGETCQGPKEEYRQCNDRKCPEPHEICDEE
SLASVVWKETPAGDAAAVRCPRNATGLILRRCTLDEEGIAYWEPPTYVKCVSIDYRNIQMMTREHLSKAQRGLMGDGLSEV
LQNLVEISQDGTSYSGDLLSTIDVLRNMTEIFRRAYHSPTSGDVQNFVQIISNILSEENRDKWEEAQLIGPNAKELFRLVE
DFIDVIGFRMKDFRDSYQVTDNLVLGIHKLPANAATDITFPMKGWRGMVDWAKNSEDKVTVSKSILSSGLTETDDSSVFVV
GTVLYRNVGNILSLQRNSTVLNSRVISVTVKPFPRSLLTPLEIEFSHLYNGTTNQTCILWDETDXVLVPSVTLIVGCGVSS
LTLLLLIIIYVSVWRYIRSERSVILINFCLSIISSNALILIGQTQTRNKVICTLVAAFLHFFFLSSFCWVLTEAWQSYMAV
TGRLRNRIIRKRFLCLGWGLPALVVAISVGFTKAKGYGTVNYCWLSLEGGLLYAFVGPAAAVVLVNMVIGILVFNKLVSKD
GITDKKLKERAGASLWSSCVVLPLLALTWMSAVLAITDRRSALFQILFAVFDSLEGFVIVMVHCILRREVQDAVKCRVVDR
QEEGNGDSGGSFQNGHAQLMTDFEKDVDMACRSVLNKDITACRTSTITGTLKRPSLQDEEKLKLNHQKGSSNFNSLPANVS
KLHLQGSPHYLGAINLNEFSNHSLTLKKDKNQPPKSIYICDGDIFKKLDSELSRAQEQTLDTSYVILPTNTSTLRAKPPKD
ESKYSINIDQMPQTRLIHLSMATDPGFVVKSPPREPVTMTCSEVQGSPMIQQQQQLPPQNIPSESQIPNSLCDTGDSGNSG
VVSKSETVSTLSMSSLERRKSRYAELDFEKIMHTRKRHQDMFQDLNRKLQHAEKEKESPTTDSKQEKQQTPNKRPWEGIRK
VQSPPSWVKKDIEPVAQSPLELKTVEWEKTGATIPLVGQDIIDLQTEV
>ggBAI2
ALIILLNFCVSILASNVLILVGQSQMLSKGVCTMTAAFLHFFFLSSFCWVLTEAWQSYLAVIGRIRTRLVRKRFLCLGWGL
PALVVAVSVGFTRTKGYGTASXXASLWSSCVVLPLLALTWMSAVLAMTDRRSILFQVLFAVFNSVQGFVIITVHGFLRREV
QDVVKCQIGVCRSEENENSPDSCKNGQVQILTDFEKDVDLACQTVLFKEVNTCNPATITGTLSRISLDGDEDPKLNTSSEG
NLGFSSLPGNIPTASILVQVPKMTPNVVAGLELGEPPAQLEAQGPVYLCTESSLRPLEYSWLRAPEPPRESDYMVLPHRSL
QPFQREEGPSTEEGTPRPTGAGEGDSYPGFVSVEHVNVNLSQPYGTVKHPYGLQFKQHPTVRQILASELSERSRTMPRTVP
GSAMKVGSLE
>ggBAI3
MKAVRNLLIYIFSTYLLVMFGFNAAQDFWCSTLVKGVIYGSYSVSEMFPKNFTNCTWTLENPDPTKYSIYLKFSKKDFSCS
NFSLLAYQFDHFSHEKIKDLLKNNHSIMQLCDSKNAFVFLQYDKNFIQIRRVYPIDFLGLHKKEEDQKSFFEFLVLNKVSP
SQFGCHVLCTWLESCLKSENGRTESCGIMYTKCTCPQHLGEWGIDDQSLVLLNNVVLPLNEQTEGCLTQELQTTQVCNLTR

EAKRPPKEEFGMMGDHTIKSQRPRSVHEKRVPQEQADAAKFMAQTGESGVEEWAQWSTCSVTCGQGSQVRTRTCVSPYGTH
CSGPLRESRVCNNTALCPVHGVWEEWSPWSLCSFTCGRGQRTRTRSCIPPQYGGRSCDGPETQHKPCNIALCPVDGQWQEW
SPWSQCSVTCSNGTQQRSRQCTAAAHGGSECRGPWAESRECYNPECTANGEWNQWGHWSGCSKSCDGGWERRIRICQGAAV
TGQQCEGTGEEVRRCSEQRCPAPYEICPEDYLMSMVWKRTPAGDMAFNRCPLNATGTTSRRCSLSVHGVAFWEHPSFARCI
SNEYRHLQHSIKEHLAKGQRMLAGDGMSQVTKTLLDLTQRKNFYAGDLLISVEILRNVTDTFKRASYIPASDGVQNFFQII
SNLLDEENKEKWEDAQQXASIQKLPAASVLTDINFPMKGRKGMVDWARNSEDRVVIPKNIFTPMSTELDESTVFVLGAVLY
KNLELILPTLRNFTVVNSKIIVVTIRPEPKTTDSFLEIELAHLSNGTLNPYCVLWDDSRTNDSLGTWSTQGCKTVLTDASH
TKCLCDRLSTFAILAQQPREIIMESSGTPSVTLIVGSGLSCLALVTLAVVYAALWRYIRSERSIILINFCLSIISSNILIL
VGQTQTHNKGICTTTTAFLHFFFLASFCWVLTEAWQSYMAVTGKIRTRLIRKRFLCLGWGLPALVVAISIGFTKTKGYGTA
HYCWLSLEGGLLYAFVGPAAAVVLVNMVIGILVFNKLVSRDGILDKKLKHRAGQMSEPHSGLTLKCAKCGVVSTTALSATT
ASNAMASLWSSCVVLPLLALTWMSAVLAMTDKRSILFQILFAVFDSLQGFVIVMVHCILRREVQDAFRCRLRNCQDPINAD
SSSSFPNGHAQIMXLHKDIGPCRAATITGTLSRISLNDEEEEKGPNPEGMSYSTLPGNIISKVIIQQPTGLHMPMSMSELA
NQCLKKDNSELRRTVYLCTDDNLRGADMDIVHPQERMIESDYIVMPRSSVNTQPSLKDESKISIGMDTLPHERLLHYKVNP
EFNMNPSVMDQFNINLDQHLPTQEHMQSLPFEPRTAVKNFIASELDDNAGLSRSETGSTISMSSLERRKSRYSDLDFEKVM
HTRKRHMELFQELNQKFQTLDRFRDVPKSGSMENSAPNKVPWDNFKTTGEYQHYTTINVLDTEAKDTLELRPAEWEKCLNL
PLDVQEGDFQTEV
>ggETL
MCAPGFKSSNGQKTFVPNDGTSCVENPEAKCVLYKDCVTEHVNKTLAEIRRLKTPLEMLQEINRNTLGPLLPVDVISYIEA
LSYSSLNTMQYSVPDNEALRNTTINVLVSTVNNFLQKDKITIWEELPVDNQRQSLTKLLHTAEQATLLMSQNFKKTTQLDA
NASDIALKVFAFDSHHMKHIHPHVYTEGDYVKISPKKKEEYQPNGTVAVVFLRYSNIGSLLSSPKNRSSKDSSEQRETVSS
SVIAVAISSNPPTLYELEKITFTLKYTKTADKDIKCAFWNYSADTMNGNWATEGCELTHSNSTHISCKCNHLTHFAVLMSS
GGSVGVSNYNILTRITQLGIIISLICLFMCIFTFWFFSEIQSTRTTIHKNLCCSLFLAELIFLIGINMNNNKLICSITAGL
LHYFLLAAFAWMCIEGIHLYLIVVGVIYNKGFLHKNFYVFGYVSPAVVVGISAALGYKYYGTTEVCWLSTKNNFIWSFIGP
ACLIILVNLLAFGVIIYKVFRHTAMLKPEVSCYENIRSCARGALALLFLLGATWIFGVLHVVQGSVVTAYLFTVFNAFQGM
FIFIFLCVLSRKIQEEYYRLFKNVPCCFGCLR
>ggHE6
XIIKVVDYIGLKLNFSAMSADFSSPSLALAVVKTNSIRSNQMSFAVQDSSDLQISLGSNAIHDLNNLGSITLPSSLLKNLP
PEELDLASRIIFNFFKKTTVFQDLSLKNASLISSVISSSVANLTISNLKANVTVTLQNIRPNQDNSTVRCVFWDFNKNGGH
GGWSYEGCIVKESRVNETVCSCNHLTSFAVLMXKIRRDYPSKILIQLCAALLLLNLVFLLDSWIALYNTRGLCIAVAVFLH
YFLLVSFTWMGLEAFHMYLALVKVFNTYVRKYILKFCIVGWGLPAVVVAIVLAVSPDNYGLISTGRVSKTRPDEFCWIKNR
IVFYITAVGYFCVIFLINISMFIVVLIQLCRIKKKKQLGAQRKTSIQDLRSVAGLTFLLGITWGFAFFTXFFIFIFYCVTK
ENVRKQWRRYLCCGKFRLAENSDWSRTATNGLKKQTVNQGVSSSSNSLQSNSNSTNSTTVLMNNDYSVHANGNGIVRRMVF
PSVCRMVMCVFKTFQANTLYFKKRMTLAAKIAVSHLEGLQRGE
>ggGPR146
MWSCETLNSTDNSEDQHLCQDFHFVLSIFSLLYLIICFPIGLCYNGLLVLVNLYNKATMTMPDVYFVNMAIAGLIINALAP
MYLLGLANTKWAIWNSNNEVYITLLILFNVSSLVTMYSTTLLSLDYYIERALPRTYMSSVYNTKHVCGFIWGGAMLTSFSS
LLFYVCNHVSXXXXXXXXXXXXXXXXXXXXXXXXXXXXXXXXXXXXXXVDSYTFTLWTPYYVTPLEGTFTYARGRPAPLN
YSRILHFTKILSKFLAFSSSFVMPLLYRYINKNFPNKLRQLLKKIHCGNQGCSHERTVVQQVMT
>ggGPR141a
MPNSSLTQQNHSFIRTVPDTSDTLRSILIILYSIDLAGGTLGVIVMSHQLFQRRSQSAMIVIIINLLVLHTFLLLSIPFRL
SYYILKEWKFGAFTCKLVSATIYLHMYITFTFYVAIIIIRLFRLEFNKCYTTIWVTAVWMLGALVIAPVFLSYYGTSRIYP
SSQCFQFQQEMEETPMVIVNYCLVGVLLAVCGLLTIIQLSVMCRLTVKYWPDINSHVEFRAQAKSFFFTLVTVVCFMPHHV
FRVYYIQNFHLDKDHELLTYNEIFLALTAMCCLDMLCFIAGIAH
>ggGPR141b
MYKENFRNMTEENIASNGSFSSFAYTNTTRAILITVYSVAFAGGGIGSTAMSFVLVKMNSLSVTTTAIINLVVVHGLLLLT
VPFRLHYYVNKEWIFHIPFCKMVSAMVHIHMYLTFLFYVITLVIRWLVFFQWKDKVEFYRKLHAIAASVAVWVIVIIFMVP
VFCIQYGRSGTYDDKACFKFHKELKQDSVKILNYLIIAAVALITSVLLGLQIFILVKVSRKLSTSLWSHQEFWAQVKNLIF
ICVIIICFLPYHLFRAYYIKYVSDSEHLESYNEVFLSLTSLSCLDLLFFVLSGSRLFKQKFGMLRSRLSCC
>ggTM7SF1
MEAPEWDPLKNDTLPPTLTPAVPPYVKLGLTIVYTVFYSLLFVFIYVQLWLVLHYRHKRFSYQTVFLFLCLFWASLRTVLF
SFYFKDFVTANSLSPFIFWLLYCFPVCLQFFTLTLMNLYFTQVIFKAKSKYSPELLKYRLPLYLASLFISLLFLLVNLTCA
VLVRTQNSERKIIVSVRVAINDTLFVLCAVSLSICLYKISKMSLANIYLESKGSSVCQVTSIGVTVILLYTSRACYNLFIL
SFSQTKKVNSFDYDWYNVSDQADLKCQLGDAGYIVFGVILFVWELLPTSLVVYFFRVRNPTKDLANAGMVPSHGFNPRSYF
FDNPRRYDSDDDLAWNIAPQGAQGSFSPDYYDWGHQNNSFMAYIGSLQQDPTLDTDRPSPI
>ggTM7SF1b
VIFKVKCAADFNKYKVLLYLGSILTSLLFLVVNLTCAMLIHSEVPENQLRRTVLARALVNDSLFILCAISLACCMCKLGKM
SSANVYLESKGTSVCQAILVGSVVALLYSSRACYNLVAVAISPDSVPGPFNYGWDNLSDKVHVEVSSEEYVVFGVVLFLWE
LVPTTFVVLFFRAQRLSQNLTPAGMVNSHSYSSRAYFFDNPRRYDSDDDLSRLGAREGGYTAGPPPSGTATDSAPLLSAAG
GSELSNHHSSYPAAQN
>ggPGR10
MSVMPSNLSLNGTSFFAENHSIMDKPNEQRTLNVFLFCSTFIIAFTVLLGSIYSLVSLLKLQNKSTISMIVTSLSIDDLIS
IVPVIIFMLTQWSSDALPQPLCTTSALIYLFQGISSNLKGSLIVSYNFYSINKTETMNCSASKRRVSMVWAILSIWIVSLL
ICILPLCGWGKYIPTTWGCFTDHASSYILFLFIVYSLCFCLLTVLSVPLTYQLLCSDEQQLLHVNYQEISRGYITPGTPAG
CSTATPCLSPVDPVDKTLKHFQNACLSSKAVFRKGVAESSGLEPRCMNNAKSRSFTVGFAQKRFSLILALTKVILWLPMMI
QMVVQHITGYQSFSFETLSFLLTLLAATVTPVFVLSEHWIHLPCGCIINCRRNSYAVSSEELKTKHRGFEFNLSFQHGYGI
YRISPESHHHDGDGKSTSCHNLLVCEKPYEPPRGGGGGAALAELSTTDSARPGPAGLSATDSARPGPAGFSTTDSARPSAA
GVRGEAASGLGRTVEGPERRLSHEEGHKPELTDWEWCRSKSERTPRQRSGGALAIPLCAFQGTVSLQAPTGKTLSLSTYEV
STEGQKITPTSKKIEVYRSKSVGHDPNPEECPNTFADTSVKIHLEVLEICDNEEALDTVSIISNISQSSTQVRSPSLRYSR

KENRFVSCDLGETASYSLFIPSNNPDSDINITIPDTVEAHRQNSKKQHMERGGYQEEIQMLNKAYRKREEDGNSN
>ggGPR88
MPNASSWSASSPLLLLWEDSSGTRIFLSLLYAVLAISGTLSNVVVIYLVFSFKKLQTTSNAFIVNGCAADLSVCALWMPQE
AVLGLLPPNSSSLRSAEYRLLREGLLGLGLTVSMASHLLVAFNRYVLITKLPSVYQALYQRRHTGCMIGLSWALALLLLPL
LPGLWTTGPAPQPPPRQTDGGSRYPALFLALAVLGQTALLLHCYLGIVRRVRGSAKRVSVLNFHLLHQLPFPAAPPPPRRA
QRRLSSVSVLLLCCVFLLGTQPLVWVSLLGFFLRPAPPALQAASWLLLCSLSALNPLLYTWRSEEFRRAARSVLPRAEGSS
AAPRHAAAAGHGAAAPPCLQLPRRRSTAG
>ggGPR142
PVNILTAVALSRLAPKTKKSSYWYLLALTTSDILTQVFIIFVGFILQTAILARAVPRAFFHIDNVLEFTANHASIWVTVLL
TVDRYVALCHPLRYRTVSYPRRTRRIIAAVFAVALATGIPFNWFPAPWRDANAFFALDRVLKWVHCVTIYFLPCSIFLAAH
SVIIRKLKQRGRSGGRRPHRGKTTALLLAVTAVFIVLWAPRTAVMMCHLYVASVKRDWRVHLALDVANMAAMLNTTLNFFL
YCFVSDTFRRAVSEVLRAHAWHGLRHGGARLVSPTLSPSQLLGTACSARGCRHAGVSVLTQHPVGPCPRDAAPLCPQSCGA
GGGGSSQPLSSSNSAAKPPPGLGPGELRSPGEEAM
>ggGPCR150
MAALLCENCTSRYHQPQLSRPLEISCMLLLIMLGKVGLDFFMLQVKHKKVKVSFMGYFCVSLALLDFILLLNIAVILYLED
FALWGVRFTKYHICLFTQIISLTYGTLHYPVYLVAGLDYYMTVAQTSQFPCRGRRLFYICVVAFIWISGFFCILKVPAIYE
ELEIRNHFSPYQCPVYVSMQSYSVSVAMLVLLGMALMVCWKEVITMLLAVRVASFAGQHVLMFSYVPNNNSTCFKQQLLTR
LLICFLGTWAPFVLFQFIILFLGAWIPAYMEMNVPWLYFINSFLIGVAYWCRCHDVELTEEMWSTDPFVSWKFCFMPFNNE
NTEPAEKPGTVIVIC
>ggNOVEL7
MPQPLPPTQLYAAERAAVLTSCVLSSLGSGLLVGTHALWPELRTRPRELLLYLSLADLLSALSYFYGVLKDFDRTSWDCVL
QGALSTFSNTSSFFWTVAVALYLYVTIVKGSPTGTGLLCCFHIVSWGVPLAITIAAVALKKIGYDASNVSVGWCWVNLDAE
DRVLWMLLTGKVWEILAYVTLPVLYILIKRHINRAXXXXXXXVSSHSLRAPAPSSWTSTADKKLILIFVIFIILRIWSTVR
FILTLCNSPAVQNSVLVVLHGIGNTFQGGANCIMFVLCTRVVRARLLSSICCCHHDDMGWPWRSSSSSRQHPDPAESEDVP
DPERTKPLLSST
>ggOA1
MASPRLETYCCPNRDAATQLVMNFQPQVFCGVCIGSASASLLLTILQLLPKKGQSLRKMPKASSSSTILLLISVCDILGGS
GVIFRSSVWLGFPSFIANISVANGTDIWPSAFCVGSAMWIQLLYSAGFWWLFCYAVDSYLVVRRSAGRSTIVLYHMMAWGL
AVLLCMEGVMLLYYPSLSSCERGLEHAIPHYITTYAPLLLVLVVNPVLFRRTVTAVASLLKGRQGIYTENERRLGTEIQMR
FFKIMLVFTVCWSSNIINESLLFYLEMQPDINETPLKNIRSAALITWIIMGVLNPMQGFLFTLPFYGWTGWKVDLKWQKRE
IPWESMSSSTVGDNDYPSPVNYQSNVHDSKKISTTDSQQTDEAISMLSE
>ggIEDA
MSVMPSNLSLNGTSFFAENHSIMDKPNEQRTLNVFLFCSTFIIAFTVLLGSIYSLVSLLKLQNKSTISMIVTSLSIDDLIS
IVPVIIFMLTQWSSDALPQPLCTTSALIYLFQGISSNLKGSLIVSYNFYSINKTETMNCSASKRRVSMVWAILSIWIVSLL
ICILPLCGWGKYIPTTWGCFTDHASSYILFLFIVYSLCFCLLTVLSVPLTYQLLCSDEQQLLHVNYQEISRGYITPGTPAG
CSTATPCLSPVDPVDKTLKHFQNACLSSKAVFRKGVAESSGLEPRCMNNAKSRSFTVGFAQKRFSLILALTKVILWLPMMI
QMVVQHITGYQSFSFETLSFLLTLLAATVTPVFVLSEHWIHLPCGCIINCRRNSYAVSSEELKTKHRGFEFNLSFQHGYGI
YRISPESHHHDGDGKSTSCHNLLVCEKPYEPPRGGGGGAALAELSTTDSARPGPAGLSATDSARPGPAGFSTTDSARPSAA
GVRGEAASGLGRTVEGPERRLSHEEGHKPELTDWEWCRSKSERTPRQRSGGALAIPLCAFQGTVSLQAPTGKTLSLSTYEV
STEGQKITPTSKKIEVYRSKSVGHDPNPEECPNTFADTSVKIHLEVLEICDNEEALDTVSIISNISQSSTQVRSPSLRYSR
KENRFVSCDLGETASYSLFIPSNNPDSDINITIPDTVEAHRQNSKKQHMERGGYQEEIQMLNKAYRKREEDGNSN
>ggPERVAR1
XXXXXXXXSVDCTSSVTFLPFPFQLQAQYLTTFFIGEGLSGLIPALIALGQGSGISSCANVSQMVNITSDNETMEGTIFRM
ETRYLPARFSTLIFFLLMTAMMVACLVAFFFLTRQPKVWELSQQQLCPSTIILNSFDQILEDDGGTGRGEGYSCPKSTKRP
METHPEKVSYSVAKLAFIYLLITWVSSLTNGVLPSVQSYSCLPYGNTAYHLSATLSSMANPLACIVAMVLPSRSLALLGTL
SLAGTGFGAYNMAIAVMSPCPLLQHSQWGDAIIILSWVLFTGTLSYVKVMAGVILRSRSHSALVWYGALEQLGSLLGALLM
FPLVNIYGFFQSADYCSLQCPV
>ggNOVEL26
MFPPGPACLPGSHLVLRWPGSHLVLWLPSCPPGSHVSPCSRRLPGSHLVLRWPGSHLAPPAAMFAPAPAGCPAAILSFGRH
LAPPAAMFPPGPAGCPAAILSLGGPAAILPPRQPSCPPGSHVAPPAAMFAPGPAGCPAAGAVGDDAEGGAGGDEDGGGDGA
GLPGVLGAIHGLRAVGGDAPRAQLRGGIGLHPLRLLQVGQNLQPRHLRPHEQATPFVAMGAGGHPCVDSTTVARYQIAXXX
XXXXXXXXXXXXXXXXXXXXXXXXXXXXXXXXXXXXXXXXXXXXXXXXXXXXXXXXXXXXXXXXXXXXXXXXXXXXXXYGS
RCFLGHSTLWGTQLYGAQSSMGPMGHGVLWGTAPYGAQHPMGPMGQGVLWGTELYGSQCSMGHRALWVLWVMVFYGAQNSM
GHSVLWVLWGTAPYGAHSSMGPMGHSTLWVTAPYGAVWCPMGGSAMGLTPWF
>ggTRHR
MENGTGDEQNHTGLLLSSQEFVTAEYQVVTILLVLLICGLGIVGNIMVVLVVLRTKHMRTPTNCYLVSLAVADLMVLVAAG
LPNITESLYKSWVYGYVGCLCITYLQYLGINASSFSITAFTIERYIAICHPIKAQFLCTFSRAKKIIIFVWSFASVYCMLW
FFLLDLNIAVYKDTTVVSCGYKVSRSYYSPIYMMDFGIFYVLPMVLATVLYGLIARILFLNPIPSDPKENSNTWKNDMAQQ
NKTVNSKMTNKSFNSTIASRRQVTKMLAVVVVLFAFLWMPYRTLVVVNSFLSSPFQENWFLLFCRICIYLNSAINPVIYNL
MSQKFRAAFRKLCNCHLKRDKKPANYSVALNYNVIKESDHFSSEIEDITVTNTYLSSAKTSIGDTCLSSEA
>ggTRHR2
MENGSASPGATLGNQTLGRMPRQPLELQVVTILLVLLVCGVGIAGNAMVVLVVLRTKHMVTPTNCYLVSLAVADLLVLLVA
GLPNISEVVASWVYGYAGCLCITYLQYLGINISAWSITAFTVERYIAICHAIRAQLLCTVARAKRIISSLWIFTSVYCLVW
FFLVDTSQVTFSDGEQVSCGYRVSRSLYMPIYFLDFAVFYVIPLGLAAVLYGLIARILYMSPLPAAPQHALGSAHRGGSLQ
QSCQGSRGTLSSRKQVWDSGGLQALFPGEWCFGNNLTSPTTATLHALLGKAVRFVSVKKQHWSPLTAQHYEEEEEEEPVGI
QGYCSREVTRCKMLSHTALLAARESLDGAQP
>ggGPR139
MEHNHLHLHNGSLVPHHRYGCGLGYAPVVYYSLLLCLGLPGNILTVIILSQLVARRQKSSYNYLLALAAADILVLFFIVFV

DFLMEDFILNKQMPQVLDKIIEVLEFSSIHTSIWITVPLTIDRYIAVCHPLKYHTVSYPARTRKVIVSVYITCFLTSIPYY
WWPNIWIEDYISTSMHHVLIWIHCFTVYLVPCSIFFILNSIIVYKLRRKSNFRLRGYSTGKTTAILFTITSIFAILWAPRI
IMILYHLYVSPINNSWVVHIVSDIANMLALLNTAINFFLYCFISKRFRTMAAATLKAFFKCQKQPVQFYTNHNFSITSSPW
ISPANSHCIKMLVYQYDKNGKPIKISP
>ggGPR120
LVLNLFCADLLFITAIPFIAIVRWTETWVLGDVICHMLFYVMTLSGTVVIVSLSAVSLERVISIARLHHTAFRRRKLLAAA
LLIWGFAAIVTLPLCCFFTVVQLPSVTGEVRVKAGWPPSTCAFLCEIHICTLDWPSHAGEIVWDVTYAVAVFLIPGLITVI
SYSKILQITKASRRSLNAGLAYSENHQIRVSQQDYKLFRALIVLMISFFIMWSPIIIIIFLILIRNYKQDLNILPSVFFWI
MLFTFANSAVNPVLYN
>ggGPR135
SAALHAVLGITATLTMALLSFDRYCAIVRQPRHKMGRRRAAQLLAAVWLAALGLAGPWYGLAGEGRREARHGAYRCVYVLP
WGSSRLGPPYGAALIVLCYLLPFALMCFCHYNICRAVRLAESRVRPLATYGNLLRAYGEMRTATTVLIMIVSIICCWGPYC
VLGLAAAAGRLPFSPTMDAVASGMAWANGAINPLIYAARNPNISVLLRRSREGGYRTRNNVAAYLSAPGRRPEPRSRAERV
RERYVNRHSGPPGSAPSSSSPASGGEVAMWACKNPAVLFCRDGQPDTASEAALQGRVDTVNTSL
>ggGRM2
MHLAPCLAAGHGSTHGKKEISTEGDLVIGGLFPVHEKGVGGEDCGKINEHRGIQRLEAMLFAPDEINKDPSILPGVRLGAH
ILGTCSKDTYALVQSLDFVRALLTRVDGANLLRLFQIPQISYASTSAKLSDKSRYDYFARTVPPDFYQAKAMSEILRFFNW
TYVSTVASEGDYGETGIEAFEQEARMRNICIATSEKVGRSMNKKTYDGVVRALLQKPNARVVVLFTRSEDARELLAAAHRA
NVSFVWVASDGWGALESVVAGSEAAAEGAITIELAAYPIQEFAAYFLNLHPYNNSRNPWFREFWEQKFKCSLHTQDCSRYS
LKTGKFEPESKITFVVNAVYAMAHSLHNMHRALCPNTTKLCDSMKPVNGKRFYKDFMLNVNFDGAPFRPADTESVVRFDRY
GDGIGRYNIFNYHRMDGRYRYQKVGYWAEGLILNTSLIPWAETSIPVSQCSDPCKKNEIKSMQPGDICCWICIPCQPYEYL
LDEFTCMDCGLGYWPNETLNGCYELPQEYIRWKDVWAIGPVTISCLGFISTLFVFGVFMKNNDTPIVKASGRELCYILLTG
VLMCYSMTFIFIAKPSTEVCTLRRLGLGTSFAVCYSALLTKTNRIARIFSGVKEGVQRPRFISPTSQVVICMALISCQLII
VIIWLLVEAPGTGKETEPDKRYIVSLKCNNRDSNMLISLTYNVLLIVLCTVYAFKTRKCPENFNEAKFIGFTMYTTCIIWL
AFLPIFYVTSSDYRVQTTTMCISVSLSGTVVLGCLFTPKLHIILFQPQKNVASHRVGTTRFSMAAASSSQSHGSASQYVPT
VCNGREAVDSTTSSL
