# Supplementary material for: The G Protein–Coupled Receptor Subset of the Chicken Genome
Source: PLoS Comput Biol. 2006 Jun 2;2(6):e54. doi: 10.1371/journal.pcbi.0020054 (PMC1472694; doi:10.1371/journal.pcbi.0020054)
Supplement: Supplementary file 3 [file pcbi.0020054.sd004.pdf]

Gene	Accesion number	Comment
NPY2R	gi|1000751	
PRLHR	gi|1002739	
OR09.01.12	gi|10092669	
V1RL1	gi|10190668	
GABBR1	gi|10835015	
HTR2A	gi|10835175	
HTR1F	gi|10835197	
HTR7	gi|10880131	
ADRA2C	gi|11055421	
EDG4	gi|11066254	
OR17.03.02	gi|11386153	
OR17.05.01	gi|11415034	
NPFF1	gi|11545887	
C3AR	gi|1199578	
T2R9	gi|12965172	
T2R8	gi|12965174	
T2R7	gi|12965176	
T2R13	gi|12965178	
T2R10	gi|12965180	
T2R14	gi|12965182	
GPR61	gi|13122461	99% Identical to the sequence used
HTR5	gi|13236497	
CELSR2	gi|13325064	
CELSR3	gi|13325066	
FKSG46	gi|13384175	
HRH2	gi|13435405	
EDG8	gi|13540517	
OR11.06.02	gi|13540539	
GPR63	gi|13540557	
OR17.01.03	gi|13562104	
OR19.04.07	gi|13624325	
OR06.05.01	gi|13624329	
OR06.04.01	gi|13624331	
OR06.11.01	gi|13624335	
OR19.04.10	gi|13775162	
OR07.01.01	gi|13929212	
CCBP2	gi|13929467	
FZD8	gi|13994190	
FKSG83	gi|14042951	
CCR9	gi|14043044	
LEC3	gi|14149677	
FKSG79	gi|14211849	
FKSG80	gi|14211851	
EMR3	gi|14211883	
GPR128	gi|14249454	
HRH4	gi|14251205	
C5R1	gi|14290436	
OR11.04.01	gi|14423840	
CHRM4	gi|14573543	97% Identical to the sequence used
GPR18	gi|15029528	
OR11.04.02	gi|15042965	
OPN4	gi|15150803	
MRGX1	gi|15546062	
MRGX3	gi|15546066	99% Identical to the sequence used
VIPR1	gi|15619006	

MRG	gi|16418463	
GPR78	gi|16566319	98% Identical to the sequence used
GPR103	gi|16566347	
TAR5	gi|16751917	
GPR101	gi|16876435	
MRGX2	gi|16876451	
MRGF	gi|16877435	
EDG2	gi|16950638	
CMKLR1	gi|1732343	
FZD5	gi|17433018	99% Identical to the sequence used
GPR124	gi|17511205	
ADRA2A	gi|178192	
ADRA2B	gi|178198	
H963	gi|17901945	
CD97	gi|17978491	
DRD2	gi|17986270	
GPR82	gi|18201870	
GHRHR	gi|183173	
LGR8	gi|18677729	
NPY1R	gi|189154	
VLGR1	gi|19882213	
GPR146	gi|19923975	
TAS1R3	gi|20142349	
V1RL2	gi|20152278	
GPCR29	gi|20152286	
T2R47	gi|20336523	
GPR73	gi|20373179	
BDKRB1	gi|20544172	
TAR1	gi|21264324	
RGR	gi|21361329	
GPR73L1	gi|21426829	
LGR4	gi|21542532	
C11ORF4	gi|21706644	
NT010306	gi|21706644	
GPCR43	gi|21748554	
OR11.23.01	gi|21928291	
OR11.20.01	gi|21928365	
OR11.28.01	gi|21928463	
OXER1	gi|22507376	
GPRC6A	gi|22507380	
FZD4	gi|22547161	
TAS1R2	gi|22726203	
OR11.06.01	gi|22748911	
DRD3	gi|227503	
AGTR2	gi|23238240	
GABABL	gi|23308585	
EMR2	gi|23397681	
P2Y11	gi|23463303	
GPR26	gi|23592220	
OR11.29.02	gi|23592222	
OR11.29.01	gi|23592230	
HE6	gi|23953678	
GPR112	gi|24285980	
GPR116	gi|24286029	98%% Identical to the sequence used
GPR113	gi|24475867	
GPR114	gi|24475871	

GPCR19	gi|24850109	
EDG1	gi|2668608	99% Identical to the sequence used
PERVAR1	gi|27447045	
PERVAR2	gi|27447047	
OPRD1	gi|27545517	
TAR4	gi|28173558	
GPR125	gi|28195599	
GNRHRII	gi|28380053	99% Identical to the sequence used
PTGDR	gi|28466969	
OR11.38.10	gi|28570170	
T2R51/50	gi|28603626	
T2R52	gi|28603628	
T2R53/44	gi|28603630	
T2R54/46	gi|28603632	
T2R61/38	gi|28603640	
OR06.03.03	gi|28626523	
NMU1R	gi|2865470	
P2Y2	gi|28872720	
CCKBR	gi|28875799	
T2R39	gi|28882026	
T2R40	gi|28882033	
T2R48	gi|28882035	
T2R60/56	gi|28973795	
P2Y12	gi|29029605	
P2Y6	gi|29029612	
T2R45	gi|29126245	
HM74a	gi|29171311	
P2Y13	gi|29171721	
PACAPR	gi|29171759	
TAS1R1	gi|29294615	
GPR123	gi|29789277	
MC3R	gi|299419	
P2Y8	gi|30023826	
OR11.42.04	gi|30039688	
F2R	gi|30354672	
GPR119	gi|30425400	
GPR8	gi|30581164	
GPR133	gi|30844176	99% Identical to the sequence used
GPR45	gi|31083315	
RDC1	gi|31083344	
T2R55	gi|31088852	
ETBRLP2	gi|31377793	
BLTR	gi|31881792	
OR06.08.02	gi|32060562	99% Identical to the sequence used
GPR120	gi|32165518	
GPR136	gi|32165522	
GPR135	gi|32261309	
OXTR	gi|32307152	
CRHR2	gi|32307159	
CHRM1	gi|32318	99% Identical to the sequence used
GPR142	gi|32401433	
GPR141	gi|32401435	
GPR54	gi|32454260	
DRD4	gi|32483397	
RLN3R2	gi|32490567	
GPR144	gi|32699042	

OPRL1	gi|33286424	
GPR72	gi|33354257	
GPR35	gi|33695097	
GPR65	gi|33695104	
GPR55	gi|33695107	
P2Y5	gi|33695113	
P2Y3L	gi|34495184	
F2RL1	gi|34577052	
GPR88	gi|34596208	
FZD6	gi|34734079	
MC1R	gi|34791	99% Identical to the sequence used
GPR62	gi|34878896	
CCR6	gi|37187860	
OR17.01.01	gi|37538297	99% Identical to the sequence used
CASR	gi|37577159	
OR07.03.01	gi|3766131	
CCR10	gi|38016879	
FSHR	gi|38016881	
NTSR1	gi|38016885	
GPR25	gi|38194224	
P2Y10	gi|38327548	
OR06.06.01	gi|38372635	98% Identical to the sequence used
OR06.12.03	gi|38372658	
OR14.02.01	gi|38372666	
OR11.15.06	gi|38372705	
OR11.48.08	gi|38372737|	
OR11.49.09	gi|38372738	
OR11.30.01	gi|38372828	
GHSR/GHRELI	gi|38455410	
FPRL2	gi|38455413	
PTGER3	gi|38505190	
SCTR	gi|38609719	
OR17.01.04	gi|38679898	
EDG3	gi|38788193	
OPRK1	gi|39725940	
OR19.04.08	gi|39812355	
OGR1	gi|40217829	
GPRC5C	gi|40217831	
HTR2B	gi|40254431	
GABBR2	gi|40255245	
GPR150	gi|40385873	
GPR97	gi|40538804	
P2Y14	gi|40789054	
SREB2	gi|40796189	
CRTH2	gi|40846396	
EDG5	gi|4090956	
PTGER1	gi|409488	
HTR4	gi|41282074	
GPR56	gi|41584200	
TBXA2R	gi|42518082	
MRGD	gi|42794265	
OR01.12.01	gi|43916899	
OR01.09.01	gi|43916920	99% Identical to the sequence used
OR11.38.08	gi|43917017	
OR11.38.07	gi|43917029	99% Identical to the sequence used
OR19.04.01	gi|43917316	

OR17.04.01	gi|43917468	
OR11.20.03	gi|43917640	
OR11.13.04	gi|43917664	99% Identical to the sequence used
OR12.04.02	gi|43917709	
OR06.07.01	gi|43917858	
OR11.07.01	gi|43917941	99% Identical to the sequence used
OR11.11.04	gi|43918031	
OR11.01.04	gi|43918253	
OR11.50.03	gi|43918301	
OR11.48.06	gi|43918331	
OR11.43.01	gi|43918460	99% Identical to the sequence used
OR11.32.04	gi|43918524	
OR11.32.05	gi|43918537	
OR11.32.03	gi|43918585	99% Identical to the sequence used
OR11.39.02	gi|43918749	
OR17.03.01	gi|43918829	
OR14.07.06	gi|43918888	
OR14.06.01	gi|43918901	
OR14.06.03	gi|43918913	99% Identical to the sequence used
OR06.02.04	gi|43918926	99% Identical to the sequence used
OR01.07.01	gi|43919110	
OR11.49.10	gi|43919137	
OR11.01.03	gi|43919220	
OR11.12.01	gi|43919413	
OR16.01.02	gi|43919515	
OR07.01.05	gi|43919698	99% Identical to the sequence used
OR07.01.03	gi|43919714	
OR11.48.07	gi|43919750	
OR11.47.03	gi|43919779	99% Identical to the sequence used
OR06.02.01	gi|43919871	
OR09.05.01	gi|43920086	99% Identical to the sequence used
OR01.13.01	gi|43920195	99% Identical to the sequence used
OR15.02.02	gi|43920429	
OR15.02.01	gi|43920442	
OR11.43.03	gi|43920554	
OR11.49.03	gi|43920737	
OR11.24.02	gi|43920887	
GPR127	gi|44887876	
ADORA1	gi|4501947	
ADORA2B	gi|4501951	
ADORA3	gi|4501953	
ADRA1D	gi|4501957	
ADRA1B	gi|4501959	
ADRB2	gi|4501969	
AGTR1	gi|4501997	
AVPR1A	gi|4502331	
AVPR1B	gi|4502333	
BAI1	gi|4502355	
BAI2	gi|4502357	
BAI3	gi|4502359	
OPN1SW	gi|4502387	
CXCR5	gi|4502415	
BRS3	gi|4502455	
CALCR	gi|4502547	
CCKAR	gi|4502607	
CCR1	gi|4502631	

CCR2	gi|4502633	
CCR3	gi|4502637	
CCR5	gi|4502639	
CCR7	gi|4502641	
CHRM3	gi|4502819	
CXC3R1	gi|4503171	
CXCR4	gi|4503175	
DRD1	gi|4503383	
DRD5	gi|4503391	
EDG6	gi|4503459	
EDNRA	gi|4503465	
FPR1	gi|4503779	
FZD1	gi|4503825	
FZD2	gi|4503827	
FZD7	gi|4503833	
FZD9	gi|4503835	
GALR1	gi|4503903	
GALR2	gi|4503905	
GALR3	gi|4503907	
GCGR	gi|4503947	
OPN1MW	gi|4503965	
GIPR	gi|4503999	
GNRHR	gi|4504059	
CMKLR2	gi|4504091	
GPR32	gi|4504093	
GHRELIN	gi|4504095	99% Identical to the sequence used
GPR39	gi|4504097	
CXCR3	gi|4504099	
GRM1	gi|4504135	
GRM4	gi|4504141	
GRM6	gi|4504145	
GRM7	gi|4504147	
LGR5	gi|4504379	
HRH1	gi|4504491	
HTR1B	gi|4504533	
HTR1D	gi|4504535	
HTR1E	gi|4504537	
HTR2C	gi|4504541	
HTR6	gi|4504545	
CXCR1	gi|4504681	
CXCR2	gi|4504683	
MAS	gi|4505105	
NMBR	gi|4505407	
OR17.04.03	gi|4505517	
OR17.01.02	gi|4505519	
P2Y1	gi|4505557	
P2Y4	gi|4505561	
PNR	gi|4505925	
PTGER4	gi|4506259	
PTGFR	gi|4506261	
PTGIR	gi|4506263	
PTHR1	gi|4506271	
GPRC5A	gi|4506403	
RHO	gi|4506527	
TACR1	gi|4507343	
TACR2	gi|4507345	

TM7SF1	gi|4507545	
TRHR	gi|4507681	
GPR52	gi|4545134	
OR09.01.02	gi|45504346	
OR06.02.03	gi|45504386	
ADRB1	gi|4557265	
ADRB3	gi|4557267	
AVPR2	gi|4557345	
BDKRB2	gi|4557359	
EDNRB	gi|4557547	
HCRTR1	gi|4557637	
HCRTR2	gi|4557639	
LHCGR	gi|4557717	
OA1	gi|4557807	
SSTR1	gi|4557857	
SSTR2	gi|4557859	
SSTR3	gi|4557861	
SSTR5	gi|4557865	
OR06.08.01	gi|45594310	
NMU2R	gi|45709228	
GPR1	gi|45709641	
OR05.01.01	gi|46047429	
OR09.06.01	gi|46048143	
CRHR1	gi|461836	
GPR152	gi|46243671	
GRM3	gi|46358417	
OR11.42.05	gi|46395479	
GPR148	gi|46409360	
GPR153	gi|46409372	
CNR2	gi|46854759	97% Identical to the sequence used
GPR158	gi|47059125	
SUCNR1	gi|47271392	
F2RL2	gi|4758326	
GLP2R	gi|4758438	
GPR50	gi|4758468	
NPFF2	gi|4758820	
GRM8	gi|47717935	
EBI2	gi|4826706	
PTHR2	gi|4826954	
OR07.04.03	gi|48717231	
OR07.04.02	gi|48717234	
OR07.04.01	gi|48717323	
AGTRL1	gi|4885057	
CCR8	gi|4885121	
GPR12	gi|4885295	
GPR15	gi|4885299	
GPR17	gi|4885301	
GPR20	gi|4885305	
GPR21	gi|4885307	
P2Y9	gi|4885311	
GPR34	gi|4885319	
ETBRLP1	gi|4885323	
GPR3	gi|4885325	
GPR40	gi|4885327	
GPR41	gi|4885329	
GPR43	gi|4885333	

GPR4	gi|4885335	
CCXCR1	gi|4885339	
GPR6	gi|4885341	
GRPR	gi|4885361	
OR01.04.01	gi|49227741	
OR01.04.06	gi|49227791	
OR01.04.07	gi|49227791	99% Identical to the sequence used
TAR3	gi|49574495	
OR09.04.01	gi|50052934	
OR11.22.01	gi|50053006	
OR14.05.01	gi|50054442	
OR01.06.01	gi|50054449	
OR11.15.01	gi|50054459	
OR11.17.04	gi|50054463	
OR11.01.05	gi|50054466	
OR01.01.01	gi|50054470	
OR01.06.02	gi|50054473	
OR11.36.02	gi|50054477	
OR11.17.01	gi|50054481	
OR11.48.03	gi|50080193	
OR11.26.03	gi|50080195	
OR09.01.07	gi|50080197	
OR19.04.04	gi|50080201	
OR09.01.09	gi|50233846	
OR11.50.01	gi|50233848	
OR14.02.02	gi|50233850	
OR01.01.05	gi|50233854	
OR11.37.02	gi|50233856	
OR01.04.09	gi|50233858	
OR01.02.01	gi|50233864	
CALCRL	gi|5031621	
CCR4	gi|5031627	
SMOH	gi|5032099	
OR17.06.01	gi|50897264	
OR11.39.05	gi|50897270	
OR11.38.02	gi|50897282	
OR11.38.03	gi|50897292	
F2RL3	gi|50960696	
OR11.34.01	gi|50979290	
OR11.31.02	gi|51230656	
Etl	gi|51458584	
OR07.02.01	gi|51466441	
HM74	gi|5174461	
MC4R	gi|5174533	
MC5R	gi|5174535	
MTNR1A	gi|5174593	
MTNR1B	gi|5174595	
OR11.49.01	gi|51783971	
OR11.39.07	gi|51783977	
OR14.07.03	gi|51854211	
OR11.49.02	gi|51871367	
OR07.01.04	gi|51921273	
OR11.19.02	gi|51921275	
OR12.01.01	gi|51921277	
OR01.04.08	gi|51921381	
GPR158L	gi|51972246	

OR09.05.02	gi|52218826	
OR19.02.01	gi|52218828	
OR11.48.02	gi|52218830	
OR09.04.04	gi|52218832	
OR19.05.05	gi|52218834	
OR11.42.02	gi|52218836	
OR01.09.02	gi|52218838	
OR11.48.01	gi|52218840	
OR01.09.06	gi|52218842	
OR19.05.02	gi|52218844	
OR01.09.07	gi|52218846	
OR11.43.02	gi|52218848	
OR14.03.01	gi|52218850	
OR01.09.04	gi|52218852	
OR09.01.05	gi|52218854	
OR09.01.03	gi|52218858	
OR14.03.02	gi|52218860	
OR09.01.01	gi|52218862	
OR09.01.10	gi|52218864	
OR0X.01.01	gi|52218868	
OR11.21.01	gi|52218872	
OR09.03.02	gi|52219202	
OR14.06.02	gi|52317094	
OR11.49.07	gi|52317096	
OR11.26.02	gi|52317100	
OR16.03.01	gi|52317102	
OR11.37.01	gi|52317104	
OR11.49.06	gi|52317106	
OR11.33.01	gi|52317108	
OR11.26.01	gi|52317111	99% Identical to the sequence used
OR03.01.01	gi|52317114	
OR01.01.03	gi|52317117	
OR11.32.01	gi|52317120	
OR11.40.03	gi|52317124	
OR03.01.02	gi|52317126	
OR11.40.06	gi|52317128	
OR11.32.02	gi|52317130	
OR11.35.03	gi|52317132	
OR11.40.07	gi|52317134	
OR11.35.02	gi|52317136	
OR11.39.04	gi|52317138	
OR11.11.05	gi|52317140	
OR11.35.01	gi|52317142	
OR11.11.02	gi|52317144	
OR11.11.07	gi|52317146	
OR11.06.03	gi|52317150	
OR11.03.01	gi|52317154	
OR19.03.01	gi|52317162	
OR09.06.02	gi|52317166	
OR11.41.02	gi|52317168	
OR09.04.02	gi|52317170	
OR11.48.04	gi|52317172	
OR11.41.01	gi|52317174	
OR01.09.05	gi|52317176	
OR11.42.03	gi|52317178	
OR09.01.06	gi|52317180	

OR11.48.05	gi|52317182	
OR09.01.04	gi|52317186	
OR11.22.02	gi|52317194	
OR07.03.02	gi|52317196	
OR01.03.01	gi|52317200	
OR01.03.02	gi|52317209	
OR01.03.05	gi|52317213	
OR01.04.02	gi|52317215	
OR01.03.04	gi|52317219	
OR01.04.03	gi|52317221	
OR01.04.04	gi|52317223	
OR19.01.01	gi|52317227	
OR11.49.04	gi|52317230	
OR16.03.02	gi|52317235	
OR11.49.14	gi|52317237	
OR11.50.04	gi|52317239	
OR11.50.05	gi|52317241	
OR17.06.02	gi|52317243	
OR11.50.02	gi|52317245	
OR14.07.05	gi|52317249	
OR14.07.02	gi|52317251	
OR14.07.04	gi|52317255	
OR14.01.01	gi|52317257	
OR14.07.01	gi|52317259	
OR11.40.02	gi|52317261	
OR11.09.01	gi|52317265	
OR11.10.01	gi|52317269	
OR11.08.01	gi|52317271	
OR11.05.01	gi|52317273	
OR11.13.03	gi|52353247	99% Identical to the sequence used
OR11.15.07	gi|52353250	
OR11.15.04	gi|52353252	
OR11.15.05	gi|52353254	96% Identical to the sequence used
OR11.15.03	gi|52353256	
OR11.18.02	gi|52353258	
OR11.20.02	gi|52353260	
OR11.17.02	gi|52353262	
OR11.18.01	gi|52353264	99% Identical to the sequence used
OR11.01.01	gi|52353266	
OR11.17.03	gi|52353268	
OR11.46.01	gi|52353270	
OR11.44.01	gi|52353274	
OR01.10.01	gi|52353276	
OR11.45.02	gi|52353278	
OR01.12.02	gi|52353280	
OR19.04.05	gi|52353282	
OR19.04.03	gi|52353284	
OR11.38.06	gi|52353286	
OR11.38.01	gi|52353288	
OR11.31.03	gi|52353290	
OR11.31.04	gi|52353292	
OR11.31.05	gi|52353294	
OR15.01.01	gi|52353296	
OR11.39.01	gi|52353298	
OR11.39.06	gi|52353300	
OR11.39.03	gi|52353304	

OR11.24.03	gi|52353311	
OR11.15.02	gi|52353326	
OR11.02.02	gi|52353330	
OR11.14.01	gi|52353332	
OR11.02.01	gi|52353334	
OR11.19.01	gi|52353336	
OR11.01.06	gi|52353338	
OR11.01.02	gi|52353340	
OR01.10.05	gi|52353342	
OR19.04.11	gi|52353344	
OR19.04.02	gi|52353346	
OR11.38.04	gi|52353348	
OR11.38.09	gi|52353350	
OR11.38.05	gi|52353354	
OR11.11.01	gi|52353941	
OR06.12.01	gi|52421345	
OR06.02.02	gi|52421347	
OR06.12.02	gi|52421349	
DUFFY	gi|52426505	
P2Y15	gi|52426789	
OR09.04.06	gi|52546685	
OR09.04.03	gi|52546687	
OR11.11.03	gi|52546689	
OR22.01.01	gi|52546691	
OR09.04.05	gi|52546735	
OR12.02.01	gi|52546737	
OR19.06.01	gi|52546739	
OR11.40.01	gi|52546741	
OR11.49.08	gi|52627147	
OR01.10.03	gi|52627155	
OR01.10.02	gi|52627158	
OR12.03.01	gi|52627160	
OR11.40.05	gi|52627162	
OR01.11.01	gi|52627164	
OR11.13.02	gi|52627166	
OR11.49.11	gi|52627199	
OR11.25.01	gi|52627201	
OR11.25.02	gi|52627203	
OR11.09.02	gi|52627205	
OR11.49.12	gi|52627207	
OR11.24.01	gi|52627211	
OR07.01.02	gi|52627213	
OR11.47.01	gi|52627215	
OR01.08.01	gi|52693915	
OR01.03.03	gi|52693917	
OR11.45.01	gi|52693923	
OR11.11.06	gi|52693925	99% Identical to the sequence used
OR06.12.05	gi|52693931	
OR11.30.02	gi|52693941	
OR01.10.04	gi|52693943	
OR01.04.05	gi|53793655	
OR11.42.01	gi|53793658	
OR14.04.01	gi|53793663	
OR14.04.02	gi|53793669	
OR11.33.03	gi|53793671	
OR06.09.01	gi|53793673	

OR11.49.05	gi|53793679	
OR09.05.03	gi|53828661	
OR12.03.02	gi|53828666	
OR05.02.01	gi|53828670	
OR06.12.04	gi|53828672	
OR09.01.08	gi|53828674	
OR09.02.01	gi|53828676	
OR11.49.13	gi|53828680	
OR11.13.01	gi|53828683	
OR03.01.03	gi|53828688	
OR14.07.09	gi|53828692	
OR11.33.02	gi|53828696	
OR11.42.06	gi|53828700	
OR11.28.02	gi|53828705	
OR12.04.01	gi|53828708	
OR11.32.06	gi|53828710	
OR14.07.07	gi|53828714	
OR19.04.06	gi|53828729	
OR03.01.04	gi|53828732	
OR11.31.01	gi|53828734	
OR14.07.08	gi|53828736	
OR14.03.03	gi|53828744	
GPR7	gi|53828924	
OR06.01.01	gi|53850574	
OR09.03.01	gi|53850576	
OR11.33.04	gi|53933201	
FPRL1	gi|54112388	
GPR19	gi|5453666	
NPY5R	gi|5453796	
CHRM2	gi|54792109	
PTGER2	gi|5524235	
OR09.01.11	gi|55633303	98% Identical to the sequence used
OR11.40.04	gi|55636071	97% Identical to the sequence used
OR01.10.06	gi|55665059	98% Identical to the sequence used
OR10.01.01	gi|55665314	
HTR1A	gi|55956923	
GPR111	gi|55958806	
OR01.05.01	gi|55961958	
OR01.01.02	gi|55961963	
OR06.03.02	gi|55961976	99% Identical to the sequence used
PPYR1	gi|55975655	
CNR1	gi|56122284	
OPRM1	gi|56549097	
SSTR4	gi|5689779	99% Identical to the sequence used
GPR87	gi|57157102	
LEC1	gi|57165355	
OR06.04.02	gi|57209701	99% Identical to the sequence used
CYSLTR1	gi|5729798	
OR11.36.01	gi|5729960	
RRH	gi|5730019	
CXCR6	gi|5730106	
GPCR-2037	gi|57977305	
GPR75	gi|5803025	
ADORA2A	gi|5921992	
ADMR	gi|6005705	
FZD10	gi|6005762	

GPR	gi|6005772	
HRH3	gi|6005782	
OR06.03.01	gi|601919	
GPR110	gi|61743940	
GPR22	gi|62088120	
GPR154	gi|62123293	
GRM5	gi|625527	
LGR6	gi|62912472	
TSHR	gi|64085121	
MCHR2	gi|66267373	
RE2	gi|6677701	
GPCR23	gi|68163574	
EDG7	gi|6912348	
LEC2	gi|6912464	
NTSR2	gi|6912538	
OR01.09.03	gi|6912550	
OR17.02.02	gi|6912552	
OR16.02.01	gi|6912554	
OR16.01.01	gi|6912556	
OR11.16.01	gi|6912560	
G2A	gi|7019387	
CHRM5	gi|7108336	
VIPR2	gi|712837	
V1RL4	gi|71361647	
ADRA1A	gi|71565156	
GLP1R	gi|717034	99% Identical to the sequence used
OPN3	gi|71999131	
T2R59	gi|72533524	
T2R56/49	gi|72533586	
OR19.05.03	gi|7330337	
OR19.05.04	gi|7330339	
OR19.05.01	gi|7363439	
OR06.10.01	gi|7363441	
GPR126	gi|74048422	
OR16.03.03	gi|74760242	
MC2R	gi|75516485	99% Identical to the sequence used
OR11.47.02	gi|75516944	
OR06.11.02	gi|75517871	
CELSR1	gi|7656967	
GPR160	gi|7657136	
OR17.02.01	gi|7657421	
OR17.04.02	gi|7657423	
TACR3	gi|7669548	
MRGX4	gi|76828028	
RLN3R1	gi|7706103	
GPRC5B	gi|7706451	
CCR11	gi|7706769	
EMR1	gi|784994	
FZD3	gi|8393378	
T2R16	gi|8394395	
T2R3	gi|8394398	
T2R4	gi|8394402	
IEDA	gi|84662753	
GPR115	gi|84781719	
MRGE	gi|85986587	
LGR7	gi|85986601	

MCHR1	gi|88758590	
CCRL2	gi|88967526	
GPRC5D	gi|8923705	
C5L2	gi|8923873	
GPR31	gi|9453792	99% Identical to the sequence used
UTRII	gi|9506745	
SREB1	gi|9506747	
OR19.04.09	gi|9506799	
SREB3	gi|9507143	99% Identical to the sequence used
T2R5	gi|9507173	
T2R1	gi|9625043	
BLTR2	gi|9789897	
OPN1LW	gi|9910526	
GPR162	gi|9951913	
GPR84	gi|9966839	
CYSLTR2	gi|9966851	
GPR92	gi|9966879	
GRM2	gi|999416	
MRGG	NA	Obtained from human genome sequence, annotated in chimpanzee
OR01.01.04	NA	
OR07.01.06	NA	
OR11.27.01	NA	
OR11.40.08	NA	
