# Supplementary material for: The G Protein–Coupled Receptor Subset of the Chicken Genome
Source: PLoS Comput Biol. 2006 Jun 2;2(6):e54. doi: 10.1371/journal.pcbi.0020054 (PMC1472694; doi:10.1371/journal.pcbi.0020054)
Supplement: Supplementary file 4 [file pcbi.0020054.st001.pdf]

Supplementary table I.

Summary of the process of identification of chicken GPCRs and in which step receptors from the respective groups were identified.

	Known	Step 1	Step 2	Step 3	Step 4	Total
Adhesion	0	16	1	0	5	22
Frizzled	9	2	0	0	0	11
Glutamate	1	9	3	2	0	15
Rhodopsin (α)	25	51	7	6	3	92
Rhodopsin (β)	25	14	5	1	0	45
Rhodopsin (γ)	17	22	3	4	0	46
Rhodopsin (δ No *Olfactory*)	10	35	6	6	4	61
Rhodopsin (*Olfactory*)	8	221	0	0	0	229
Secretin	4	9	0	1	0	14
Taste 2	0	1	0	0	2	3
Other	3	10	0	2	4	19
Total	102	390	25	22	18	557
